# Supplementary material for: A Catalog of Coding Sequence Variations in Salivary Proteins’ Genes Occurring during Recent Human Evolution
Source: Int J Mol Sci. 2023 Oct 9;24(19):15010. doi: 10.3390/ijms241915010 (PMC10573131; doi:10.3390/ijms241915010)

PRB1: rs554211998

chr12:11506784 T/C

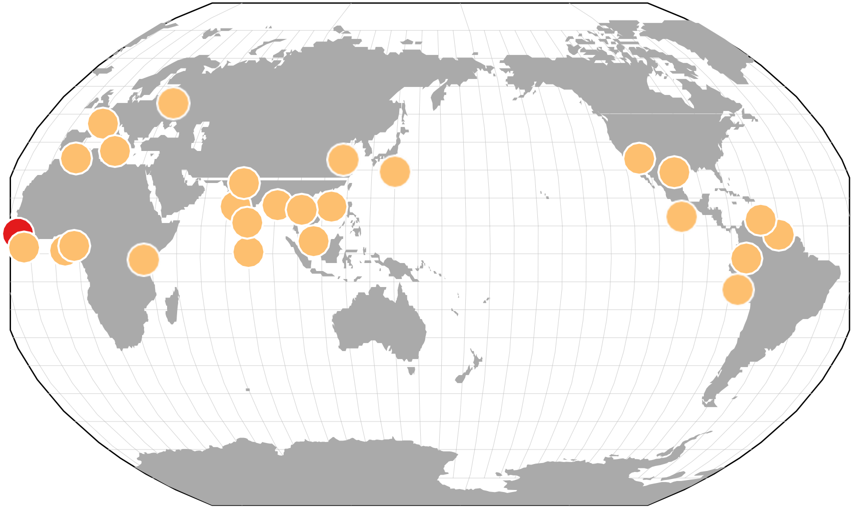

Frequency Scale = Proportion out of 0.01
The pie below represents a minor allele frequency of 0.0025

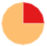

Sample sizes below 30 become increasingly transparent to represent uncertain frequencies, i.e.

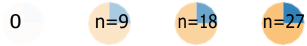

PRB1: rs534597111

chr12:11506723 A/C

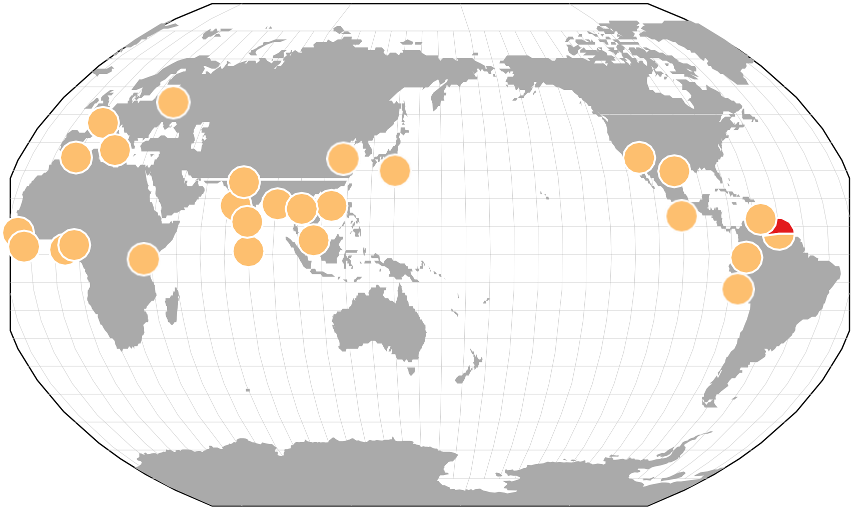

Frequency Scale = Proportion out of 0.01
The pie below represents a minor allele frequency of 0.0025

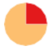

Sample sizes below 30 become increasingly transparent to represent uncertain frequencies, i.e.

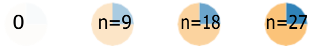

PRB1: rs369001998

chr12:11506369 A/G

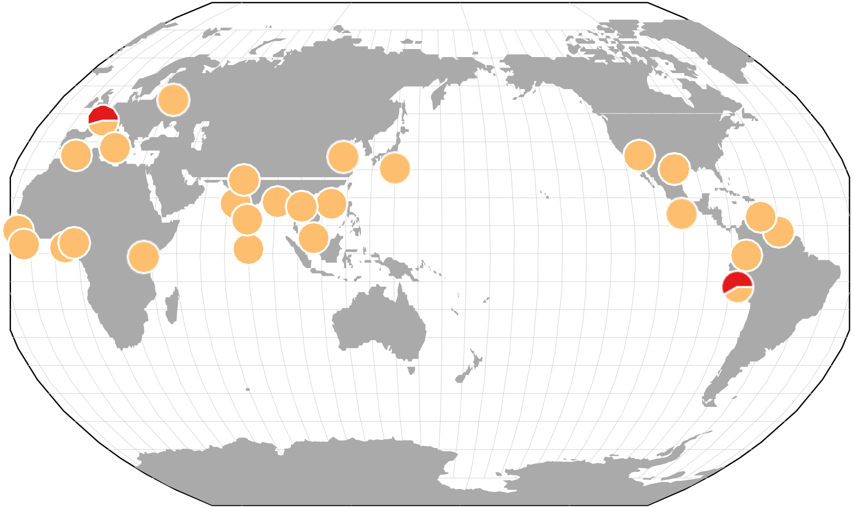

Frequency Scale = Proportion out of 0.01
The pie below represents a minor allele frequency of 0.0025

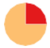

Sample sizes below 30 become increasingly transparent to represent uncertain frequencies, i.e.

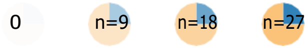

PRB2: rs188924826

chr12:11546899 T/C

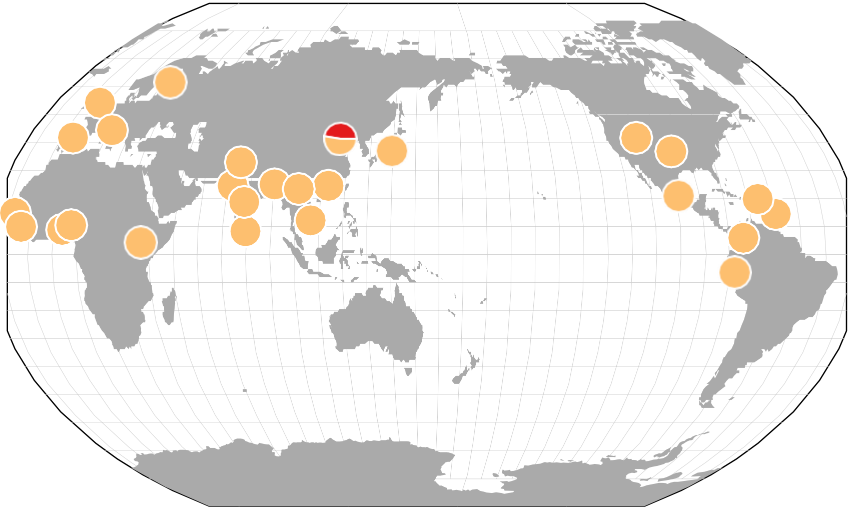

Frequency Scale = Proportion out of 0.01

The pie below represents a minor allele frequency of 0.0025

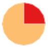

Sample sizes below 30 become increasingly transparent to represent uncertain frequencies, i.e.

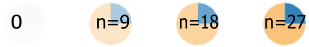

PRB2: rs201994479

chr12:11546764 T/G

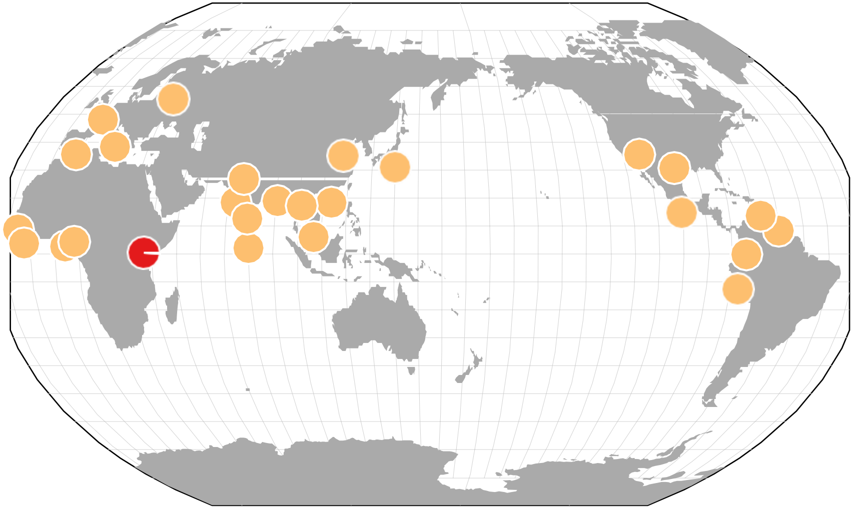

Frequency Scale = Proportion out of 0.01
The pie below represents a minor allele frequency of 0.0025

Sample sizes below 30 become increasingly transparent to represent uncertain frequencies, i.e.

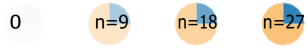

PRB2: rs201392419

chr12:11546462 T/G

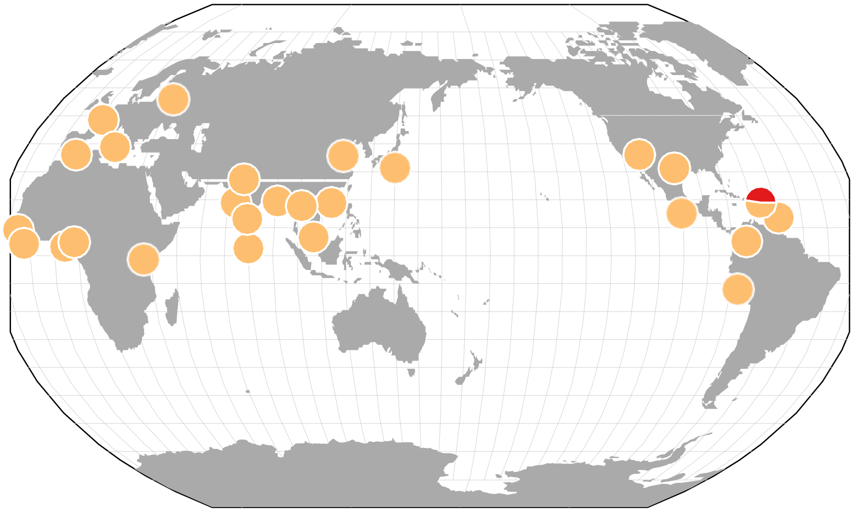

Frequency Scale = Proportion out of 0.01

The pie below represents a minor allele frequency of 0.0025

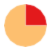

Sample sizes below 30 become increasingly transparent to represent uncertain frequencies, i.e.

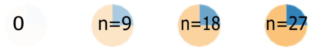

PRB2: rs11054277

chr12:11546395 T/G

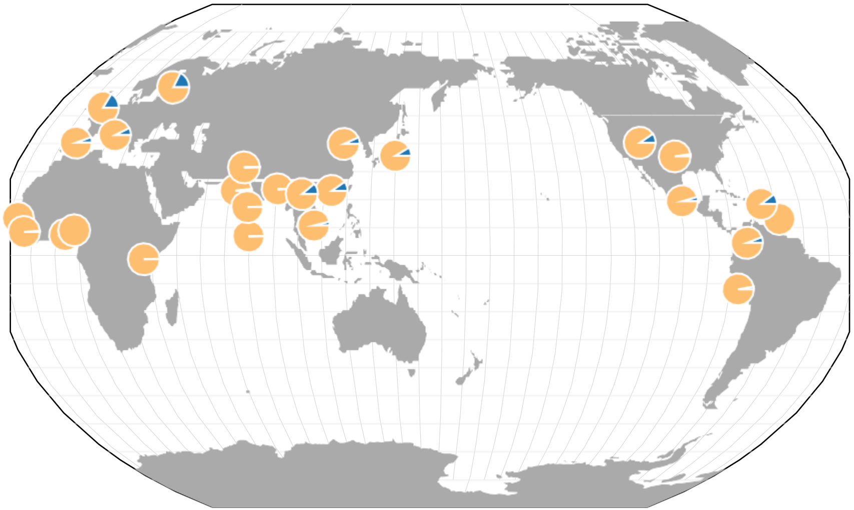

Frequency Scale = Proportion out of 1

The pie below represents a minor allele frequency of 0.25

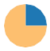

Sample sizes below 30 become increasingly transparent to represent uncertain frequencies, i.e.

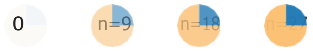

PRB2: rs34305575

chr12:11546314 C/T

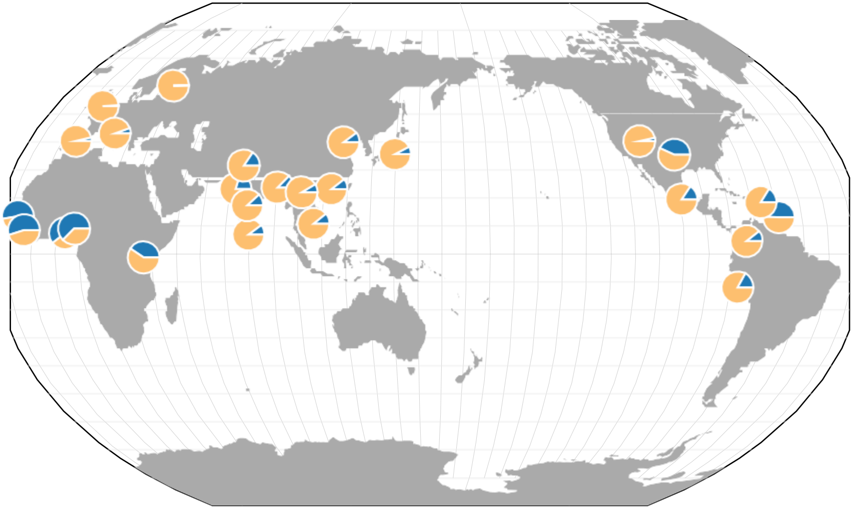

Frequency Scale = Proportion out of 1

The pie below represents a minor allele frequency of 0.25

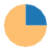

Sample sizes below 30 become increasingly transparent to represent uncertain frequencies, i.e.

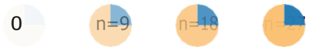

PRB2: rs201391404

chr12:11546023 T/G

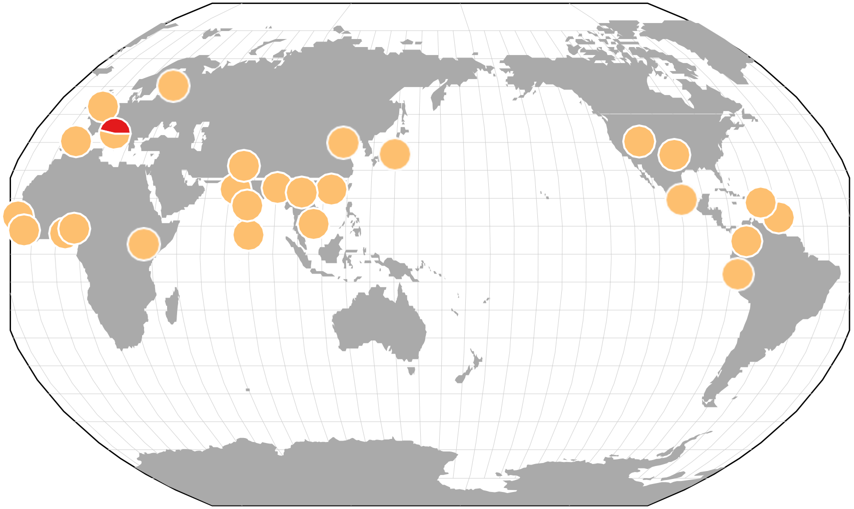

Frequency Scale = Proportion out of 0.01

The pie below represents a minor allele frequency of 0.0025

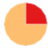

Sample sizes below 30 become increasingly transparent to represent uncertain frequencies, i.e.

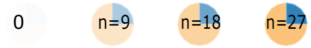

PRB3: rs533382585

chr12:11421002 A/G

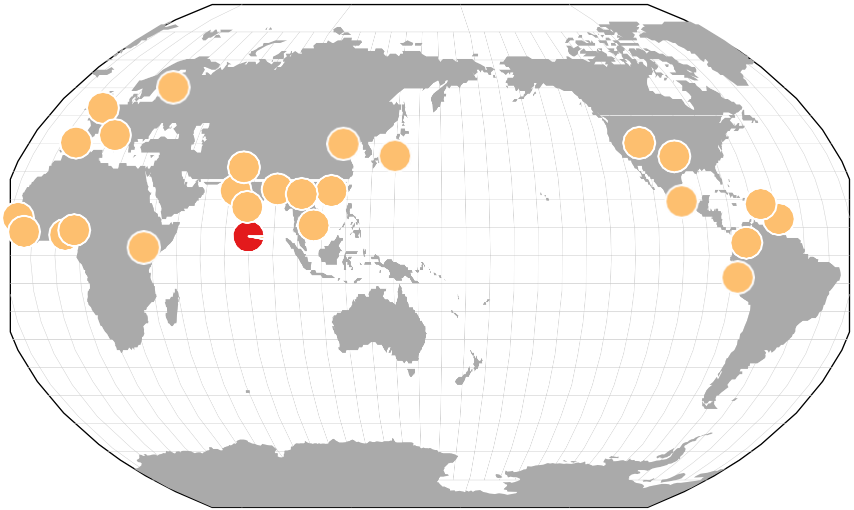

Frequency Scale = Proportion out of 0.01

The pie below represents a minor allele frequency of 0.0025

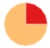

Sample sizes below 30 become increasingly transparent to represent uncertain frequencies, i.e.

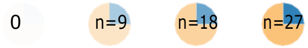

CST1: rs6076122

chr20:23731494 A/G

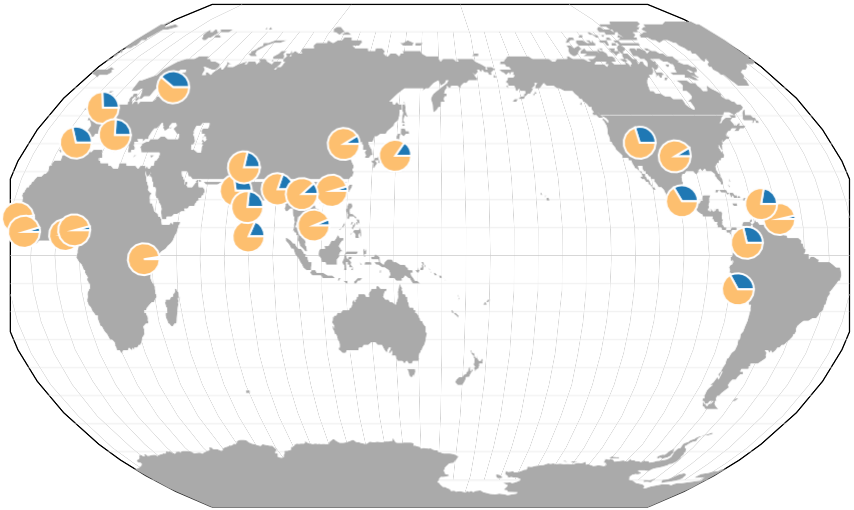

Frequency Scale = Proportion out of 1

The pie below represents a minor allele frequency of 0.25

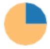

Sample sizes below 30 become increasingly transparent to represent uncertain frequencies, i.e.

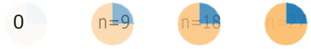

CST1: rs368203290

chr20:23731344 T/C

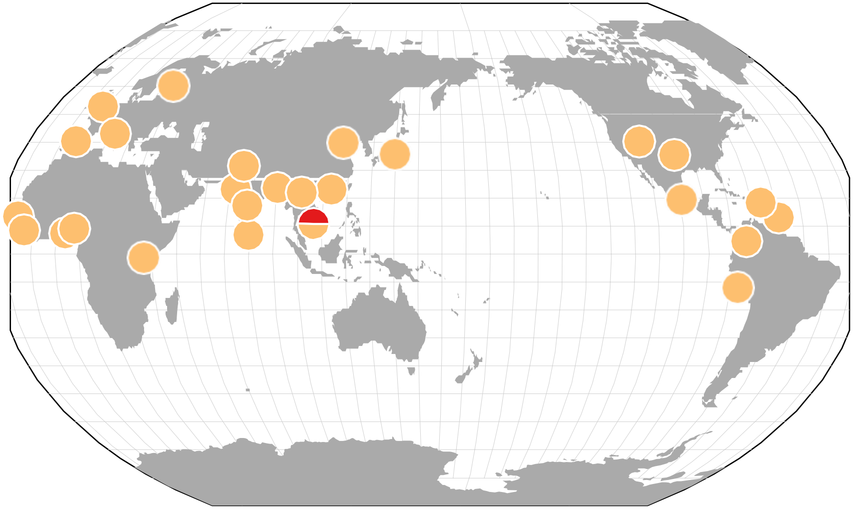

Frequency Scale = Proportion out of 0.01

The pie below represents a minor allele frequency of 0.0025

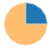

Sample sizes below 30 become increasingly transparent to represent uncertain frequencies, i.e.

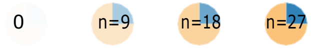

CST1: rs111349461

chr20:23807162 A/G

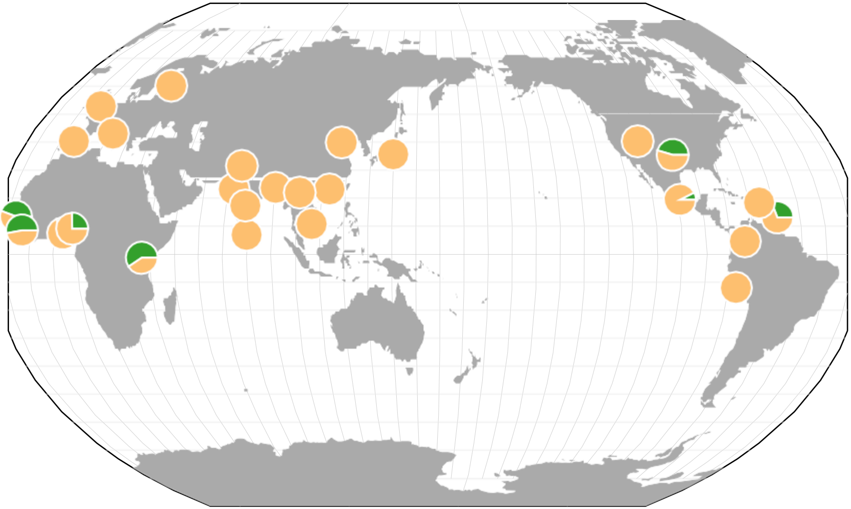

Frequency Scale = Proportion out of 0.1

The pie below represents a minor allele frequency of 0.025

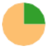

Sample sizes below 30 become increasingly transparent to represent uncertain frequencies, i.e.

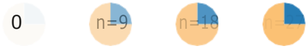

CST1: rs55860552

chr20:23807093 A/G

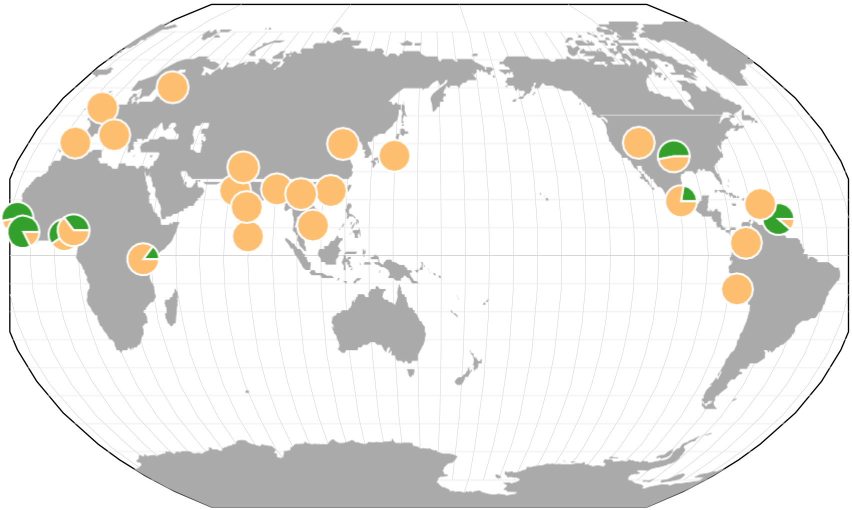

Frequency Scale = Proportion out of 0.1

The pie below represents a minor allele frequency of 0.025

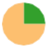

Sample sizes below 30 become increasingly transparent to represent uncertain frequencies, i.e.

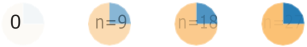

CST1: rs568411970

chr20:23807084 A/G

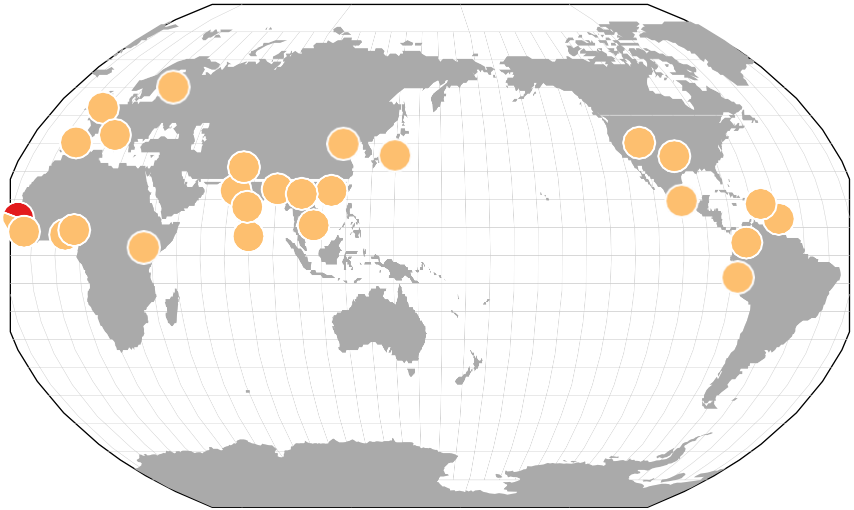

Frequency Scale = Proportion out of 0.01

The pie below represents a minor allele frequency of 0.0025

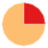

Sample sizes below 30 become increasingly transparent to represent uncertain frequencies, i.e.

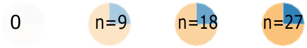

CST1: rs202150666

chr20:23804691 C/A

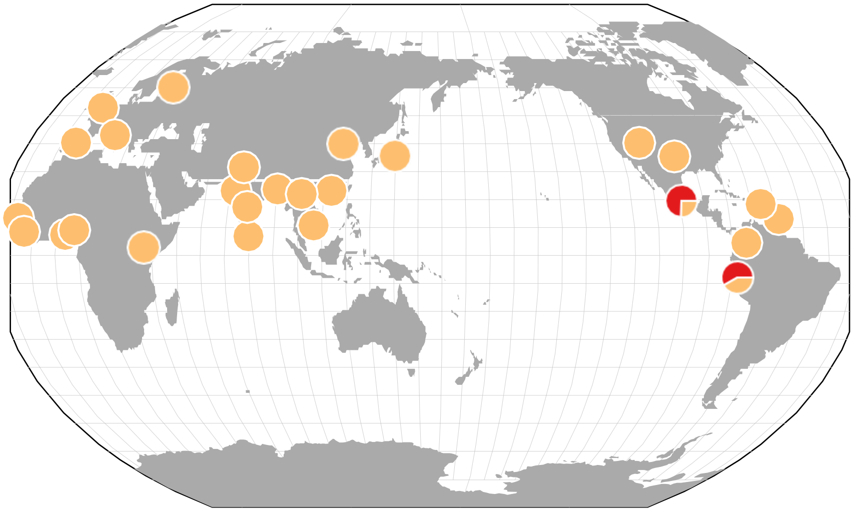

Frequency Scale = Proportion out of 0.01

The pie below represents a minor allele frequency of 0.0025

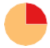

Sample sizes below 30 become increasingly transparent to represent uncertain frequencies, i.e.

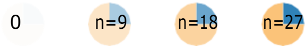

CST5: rs145031249

chr20:23860243 A/G

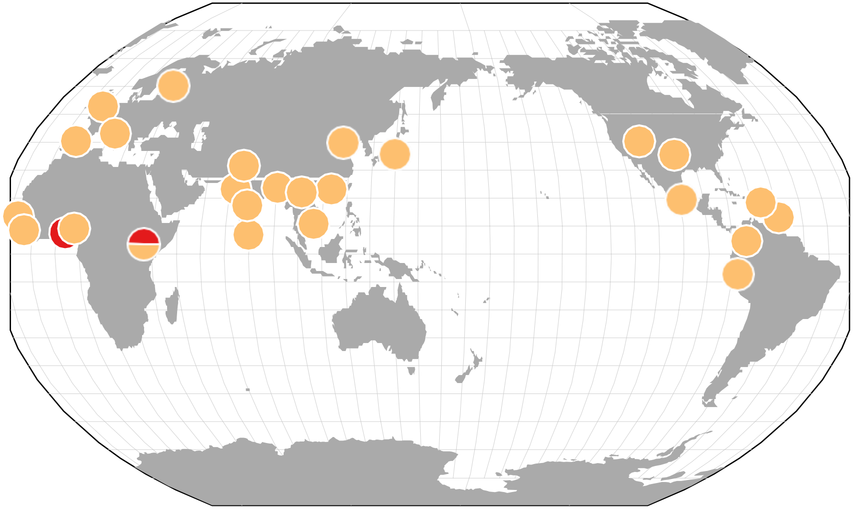

Frequency Scale = Proportion out of 0.01

The pie below represents a minor allele frequency of 0.0025

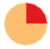

Sample sizes below 30 become increasingly transparent to represent uncertain frequencies, i.e.

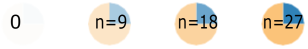

CST5: rs1799841

chr20:23860178 A/G

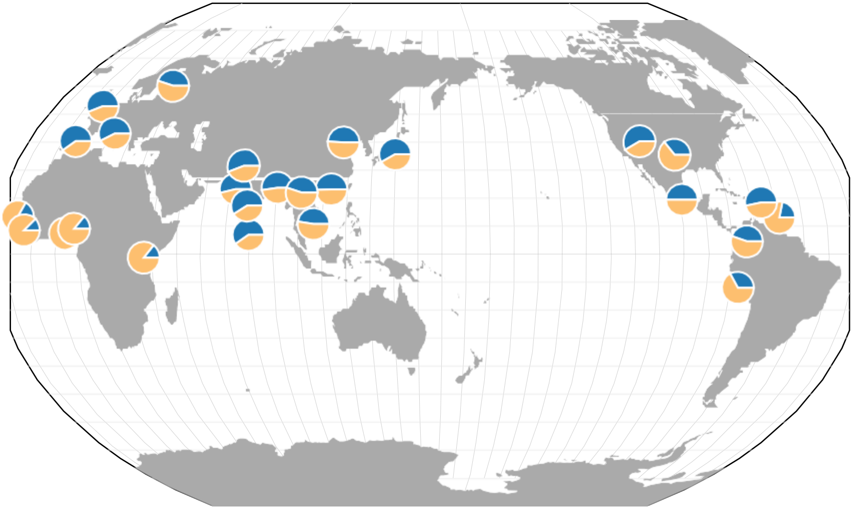

Frequency Scale = Proportion out of 1

The pie below represents a minor allele frequency of 0.25

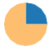

Sample sizes below 30 become increasingly transparent to represent uncertain frequencies, i.e.

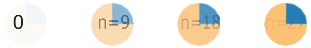

CST5: rs41282292

chr20:23858200 A/G

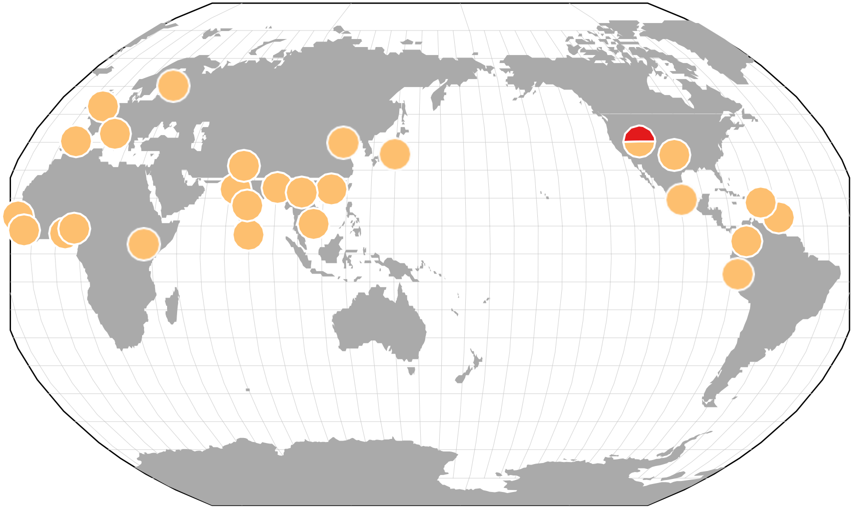

Frequency Scale = Proportion out of 0.01

The pie below represents a minor allele frequency of 0.0025

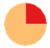

Sample sizes below 30 become increasingly transparent to represent uncertain frequencies, i.e.

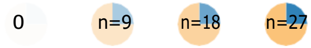

CSTB: rs559906825

chr21:45194562 T/C

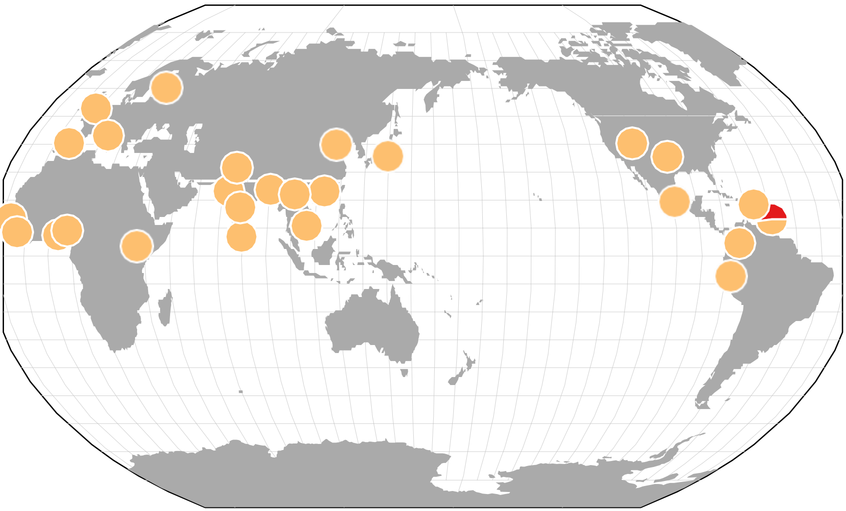

Frequency Scale = Proportion out of 0.01

The pie below represents a minor allele frequency of 0.0025

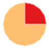

Sample sizes below 30 become increasingly transparent to represent uncertain frequencies, i.e.

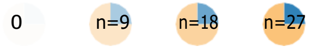

Supplement: Supplementary file 1 [file ijms-24-15010-s001.zip › File S1.pdf]
