# Supplementary material for: A Catalog of Coding Sequence Variations in Salivary Proteins’ Genes Occurring during Recent Human Evolution
Source: Int J Mol Sci. 2023 Oct 9;24(19):15010. doi: 10.3390/ijms241915010 (PMC10573131; doi:10.3390/ijms241915010)
Supplement: Supplementary file 1 [file ijms-24-15010-s001.zip › File S2 new.pdf]

PBS (Population Branch Statistics) plot of basic proline rich proteins and cysteine proteases. The dotted and continuous lines represent the empirical reference values at the 99.5th and 99.9th percentile respectively, so that the PBS values above these lines indicate significant values.

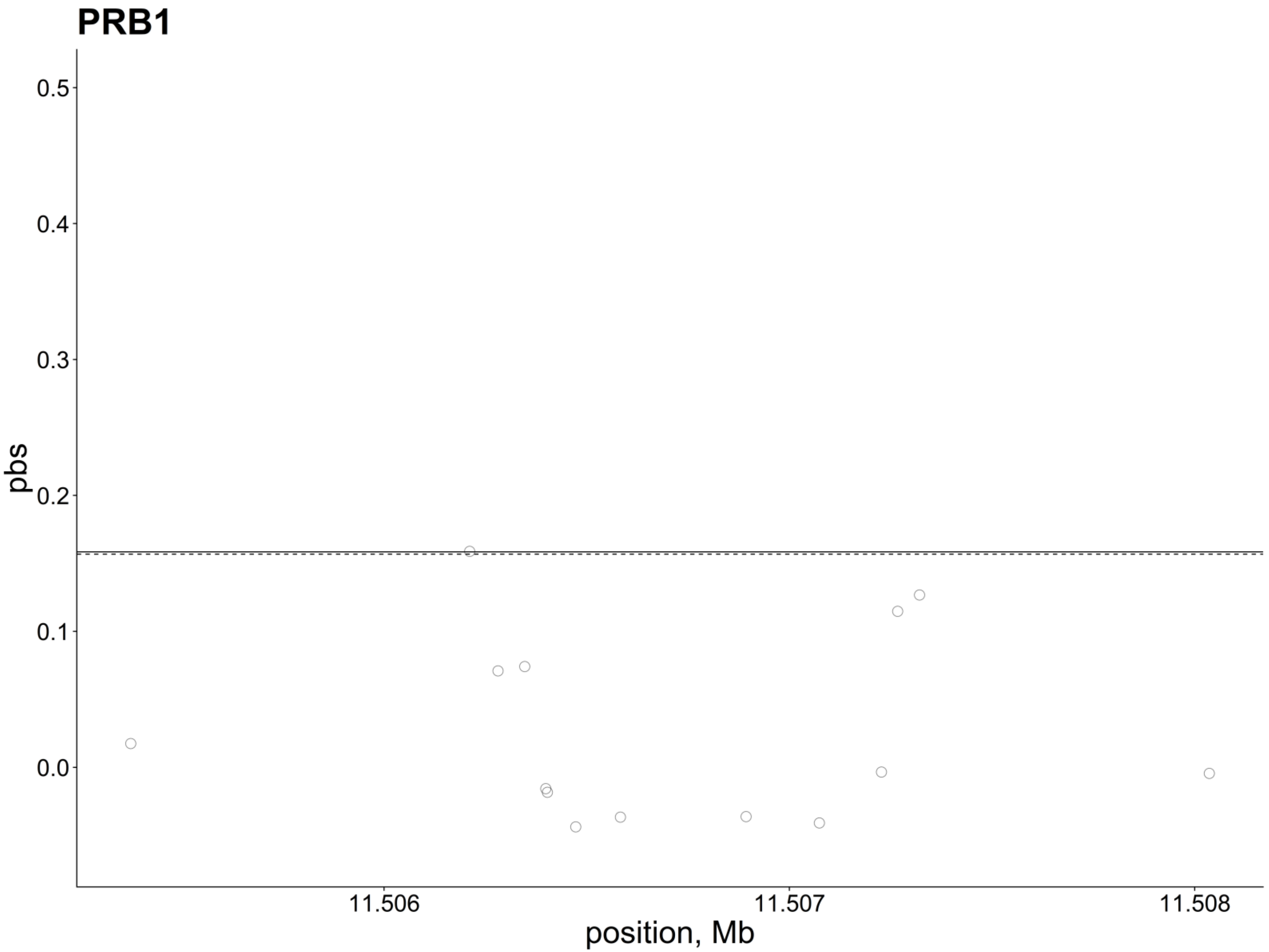

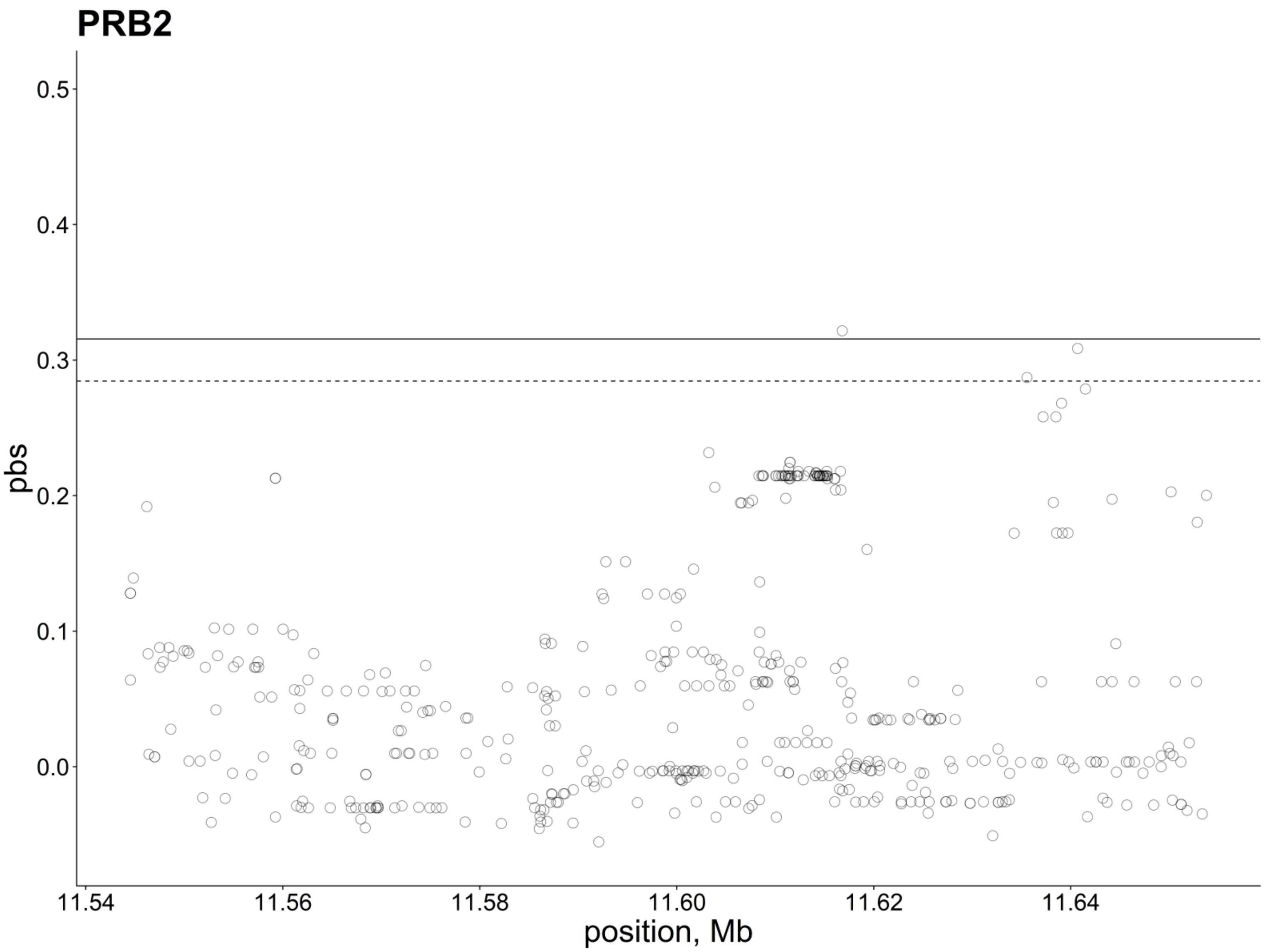
PRB2
0.5
0.4
0.3
0.2
0.1
0.0
pbs
11.54
11.56
11.58
11.60
11.62
11.64
position, Mb

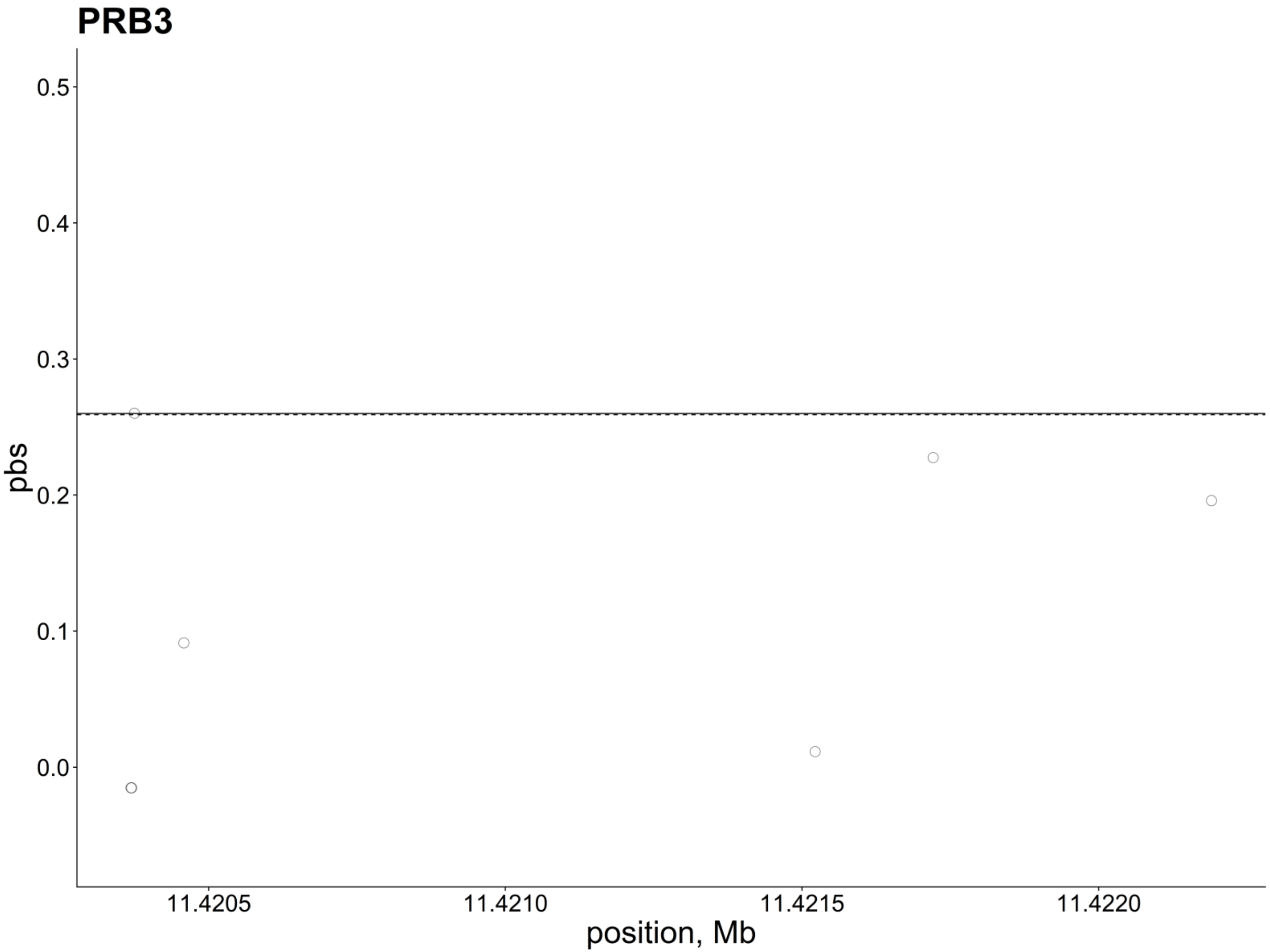
PRB3
0.5
0.4
0.3
0.2
0.1
0.0
pbs
11.4205
11.4210
11.4215
11.4220
position, Mb

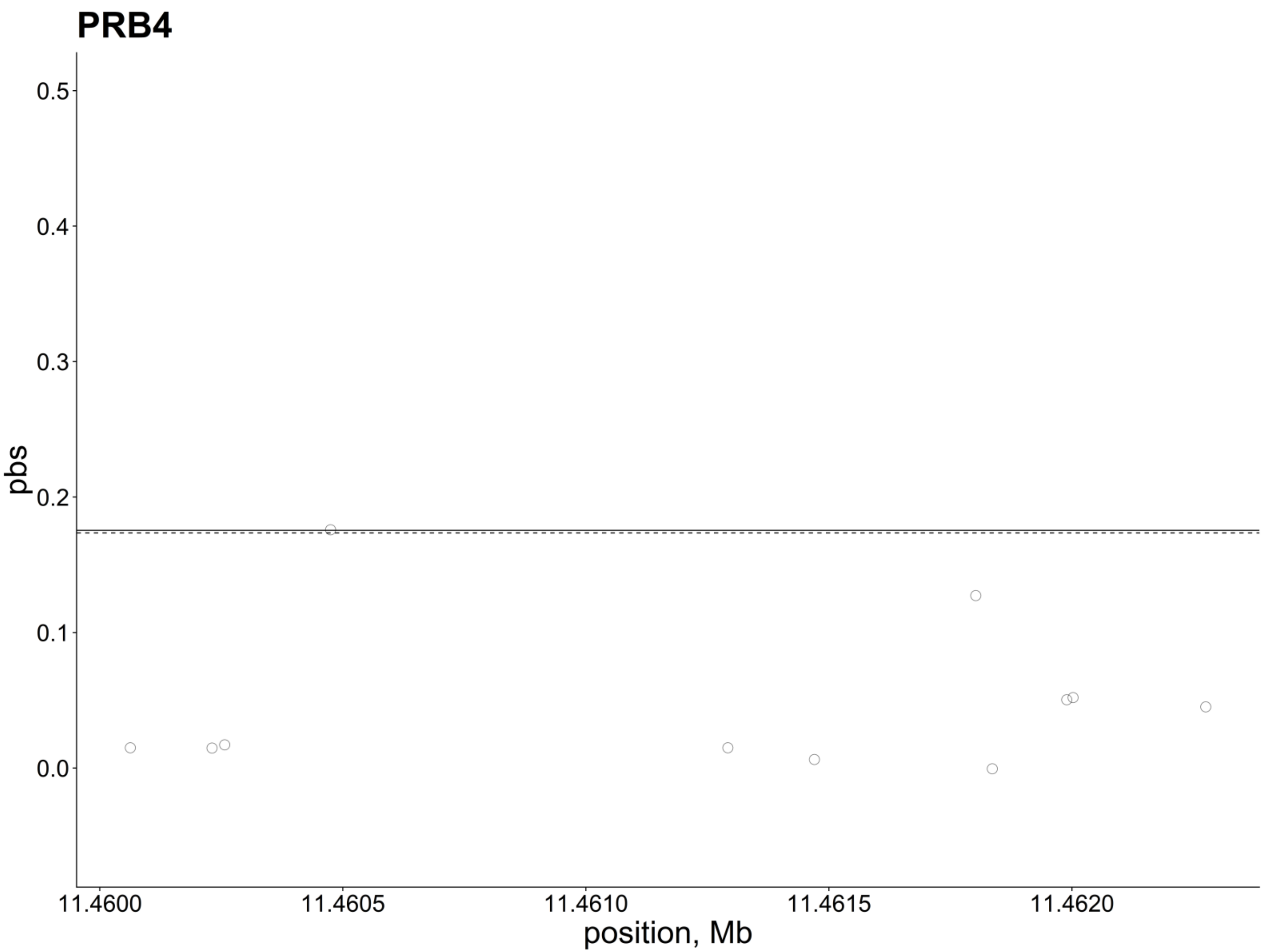
PRB4
pbs
0.5
0.4
0.3
0.2
0.1
0.0
11.4600
11.4605
11.4610
11.4615
11.4620
position, Mb

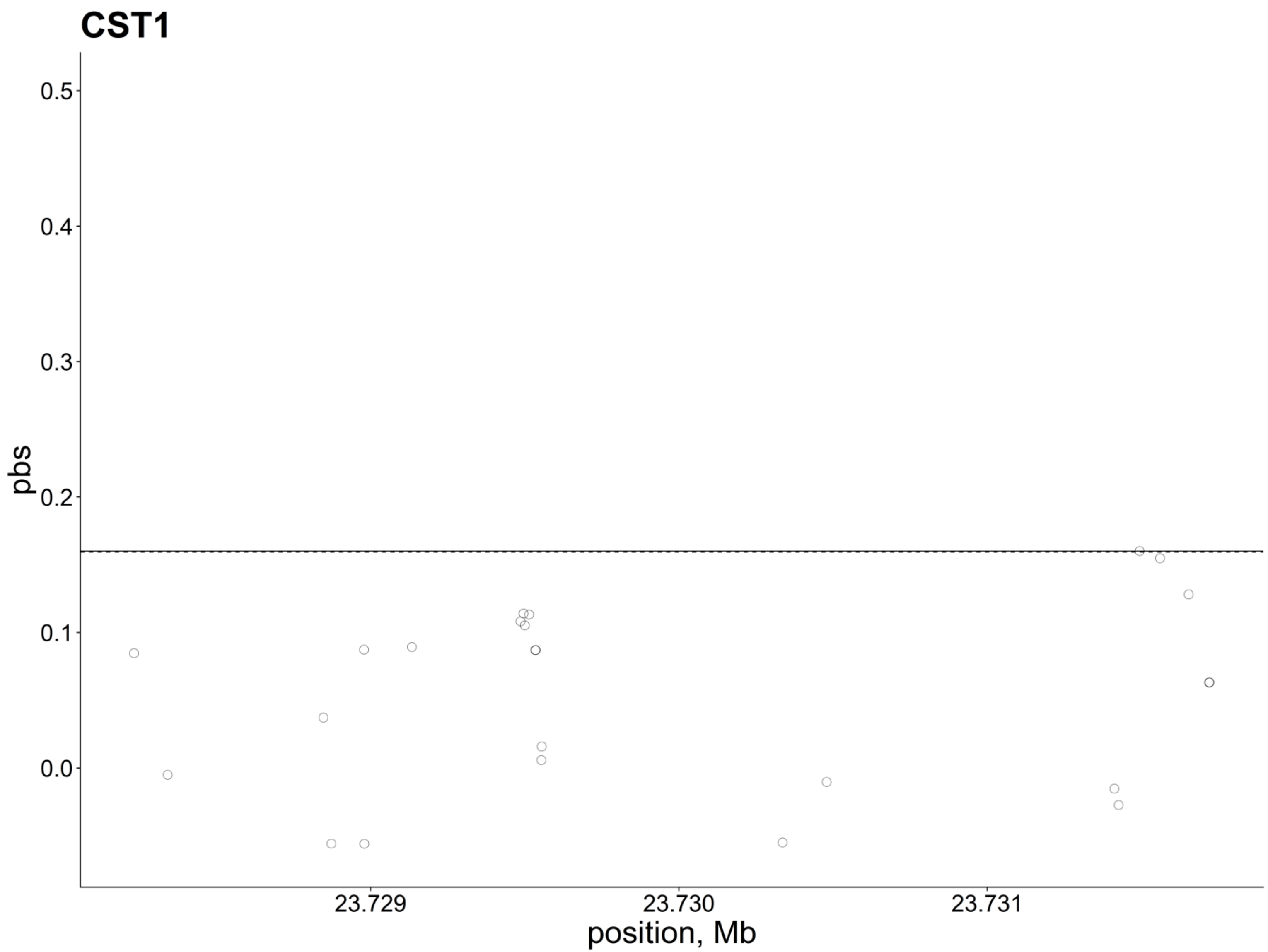

CST1
pbs
0.5
0.4
0.3
0.2
0.1
0.0
23.729
23.730
23.731
position, Mb

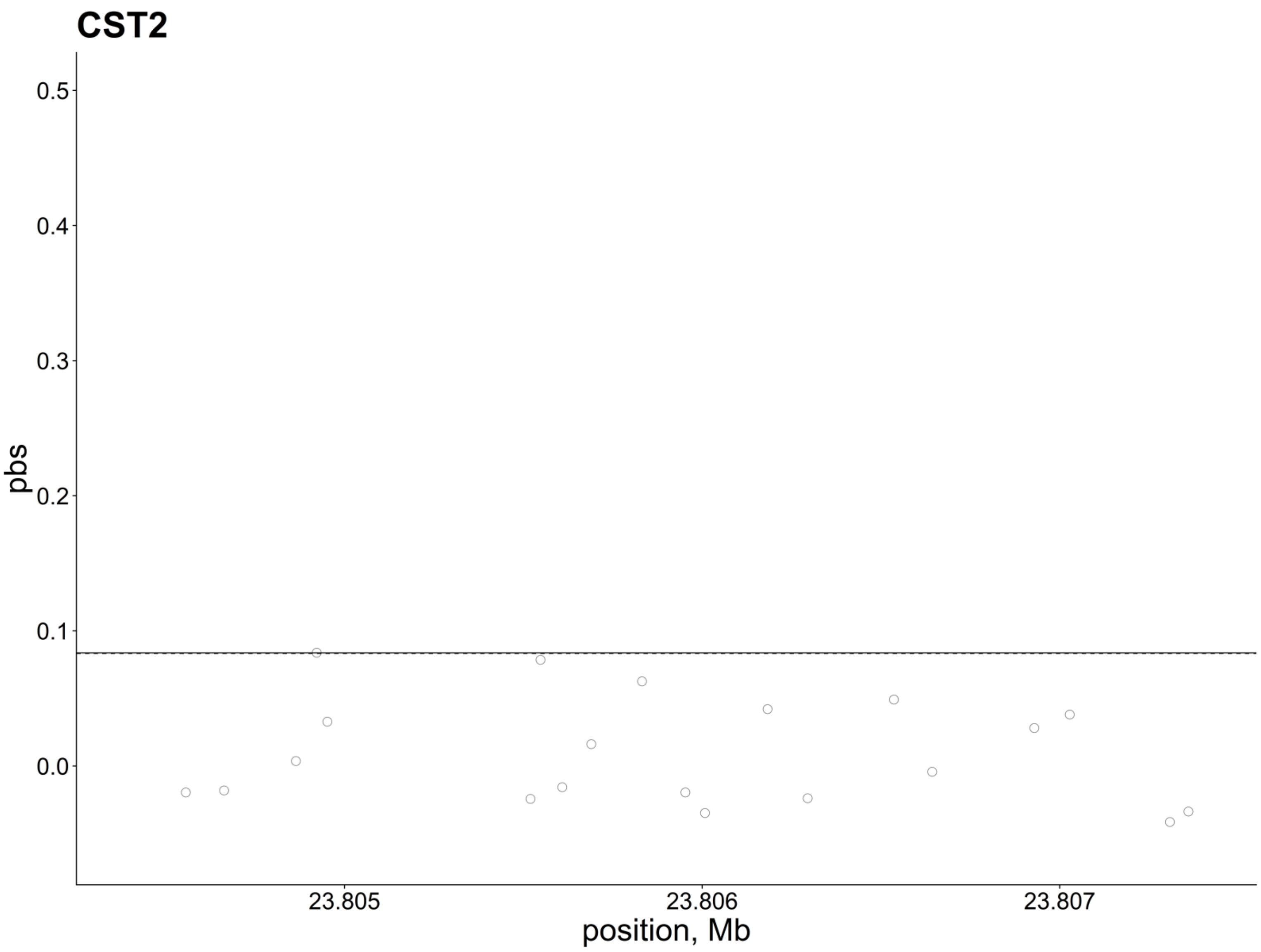

CST2
pbs
0.5
0.4
0.3
0.2
0.1
0.0
23.805
23.806
23.807
position, Mb

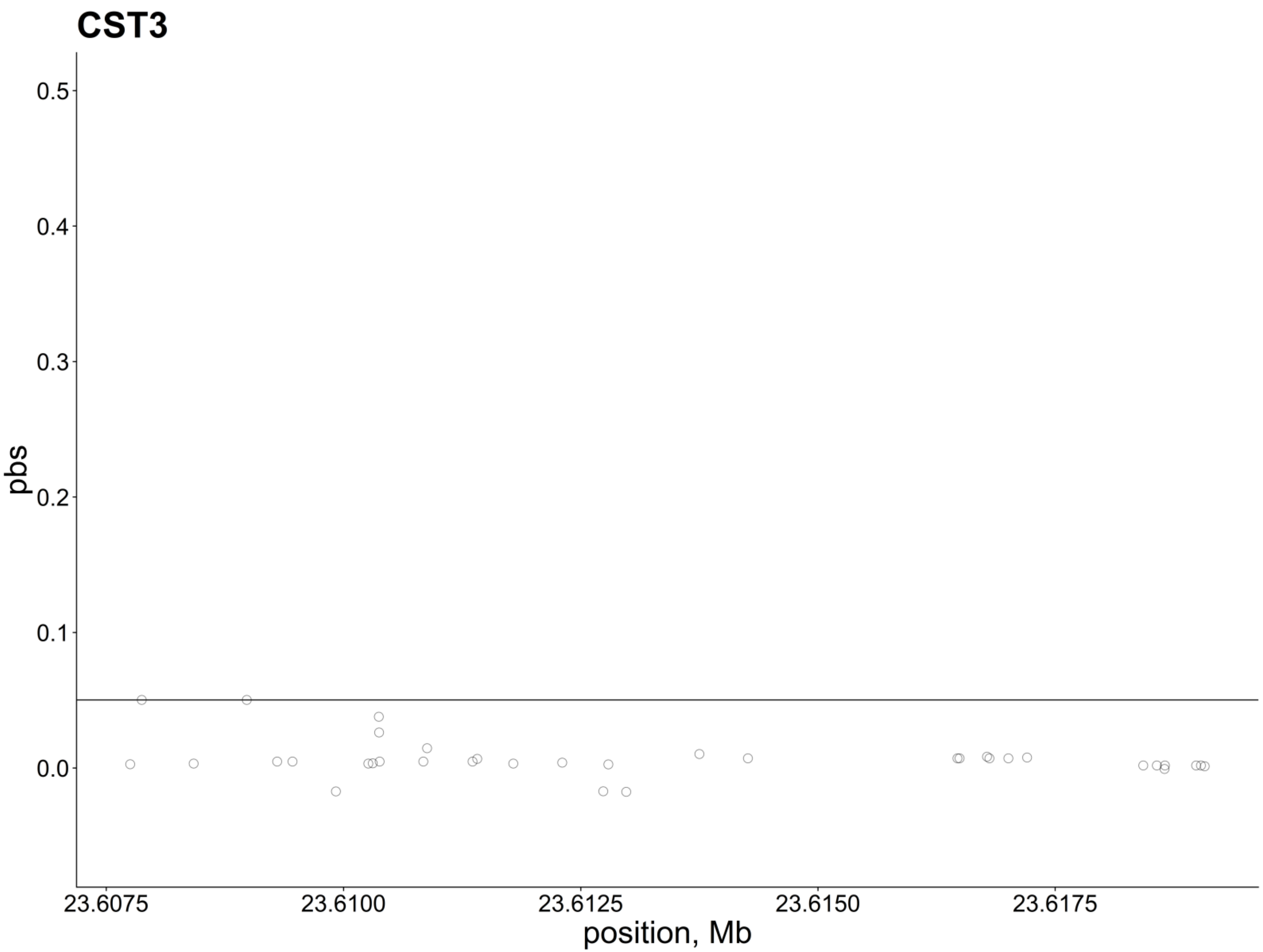
CST3
pbs
0.5
0.4
0.3
0.2
0.1
0.0
23.6075
23.6100
23.6125
23.6150
23.6175
position, Mb

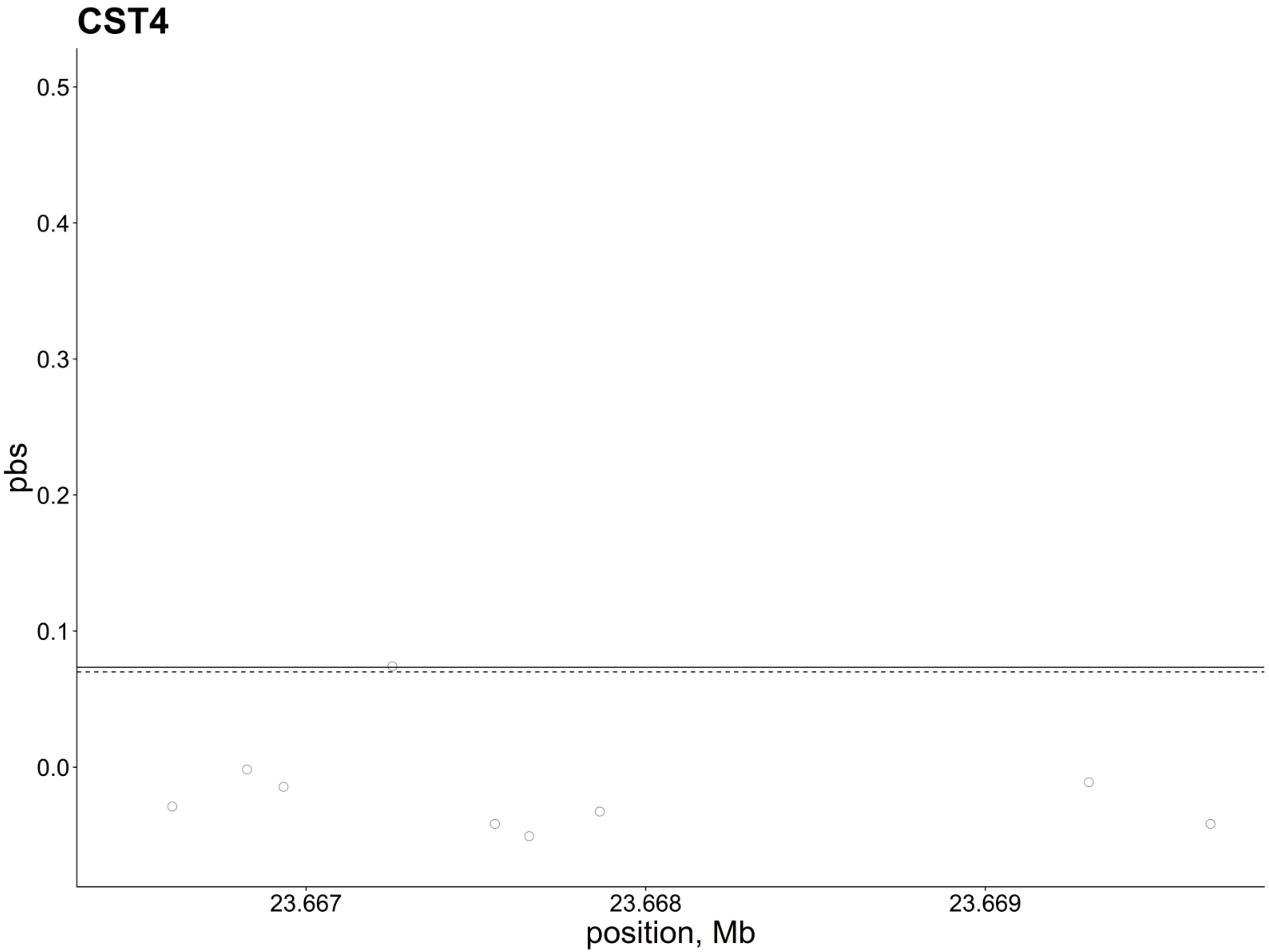
CST4
pbs
0.5
0.4
0.3
0.2
0.1
0.0
23.667
23.668
23.669
position, Mb

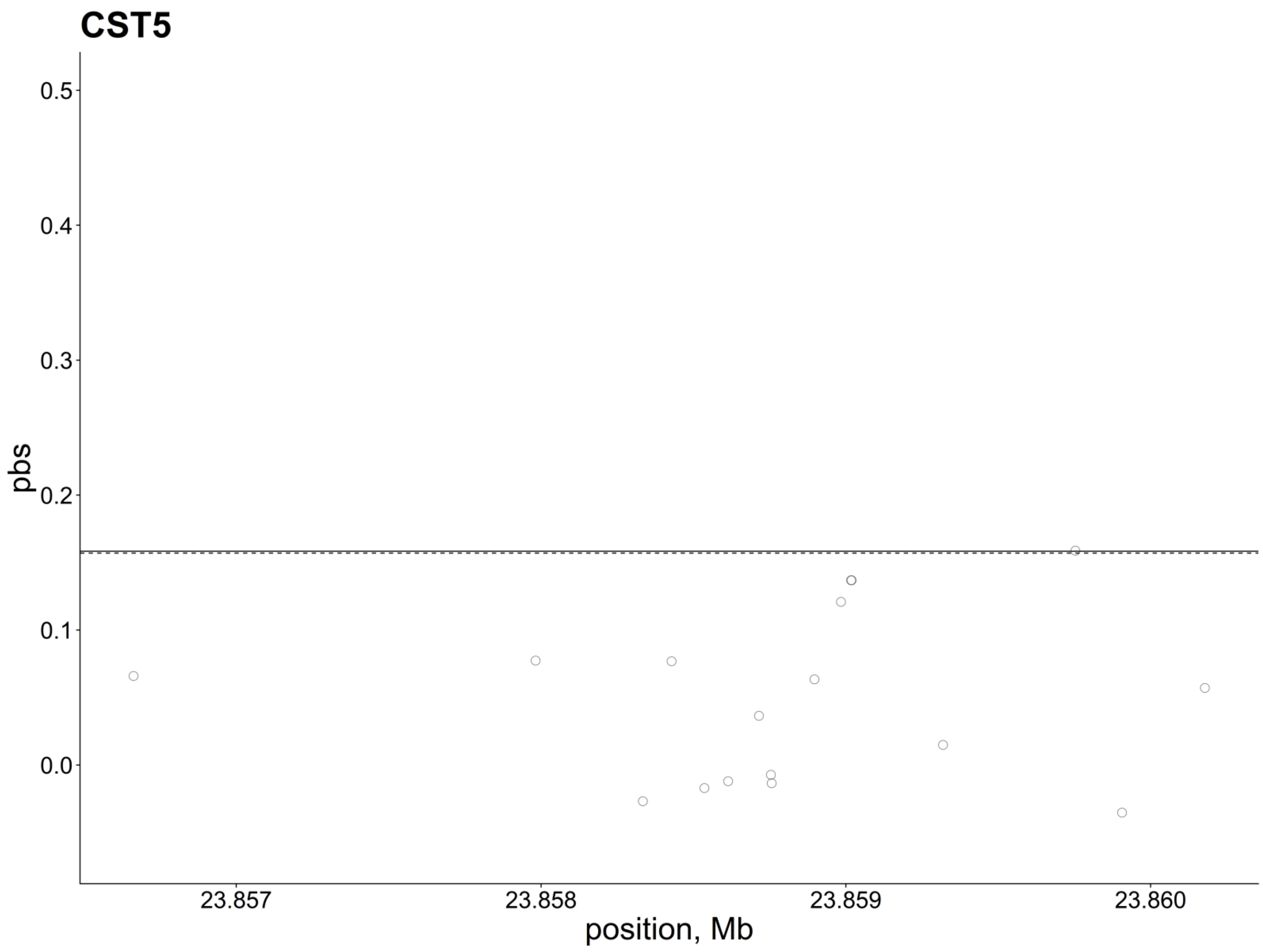
CST5
pbs
0.5
0.4
0.3
0.2
0.1
0.0
23.857
23.858
23.859
23.860
position, Mb

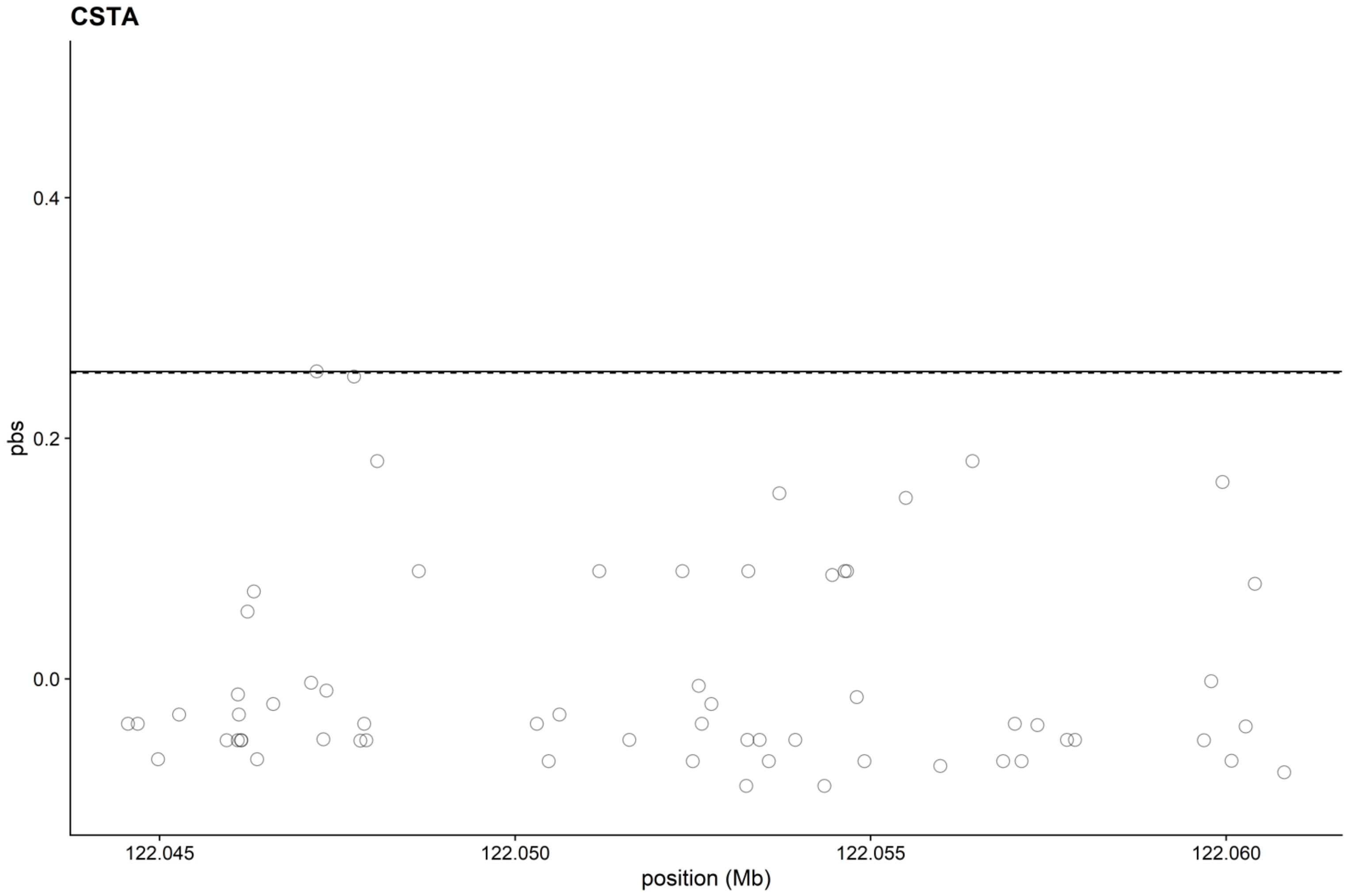
CSTA
pbs
0.4
0.2
0.0
122.045
122.050
122.055
122.060
position (Mb)

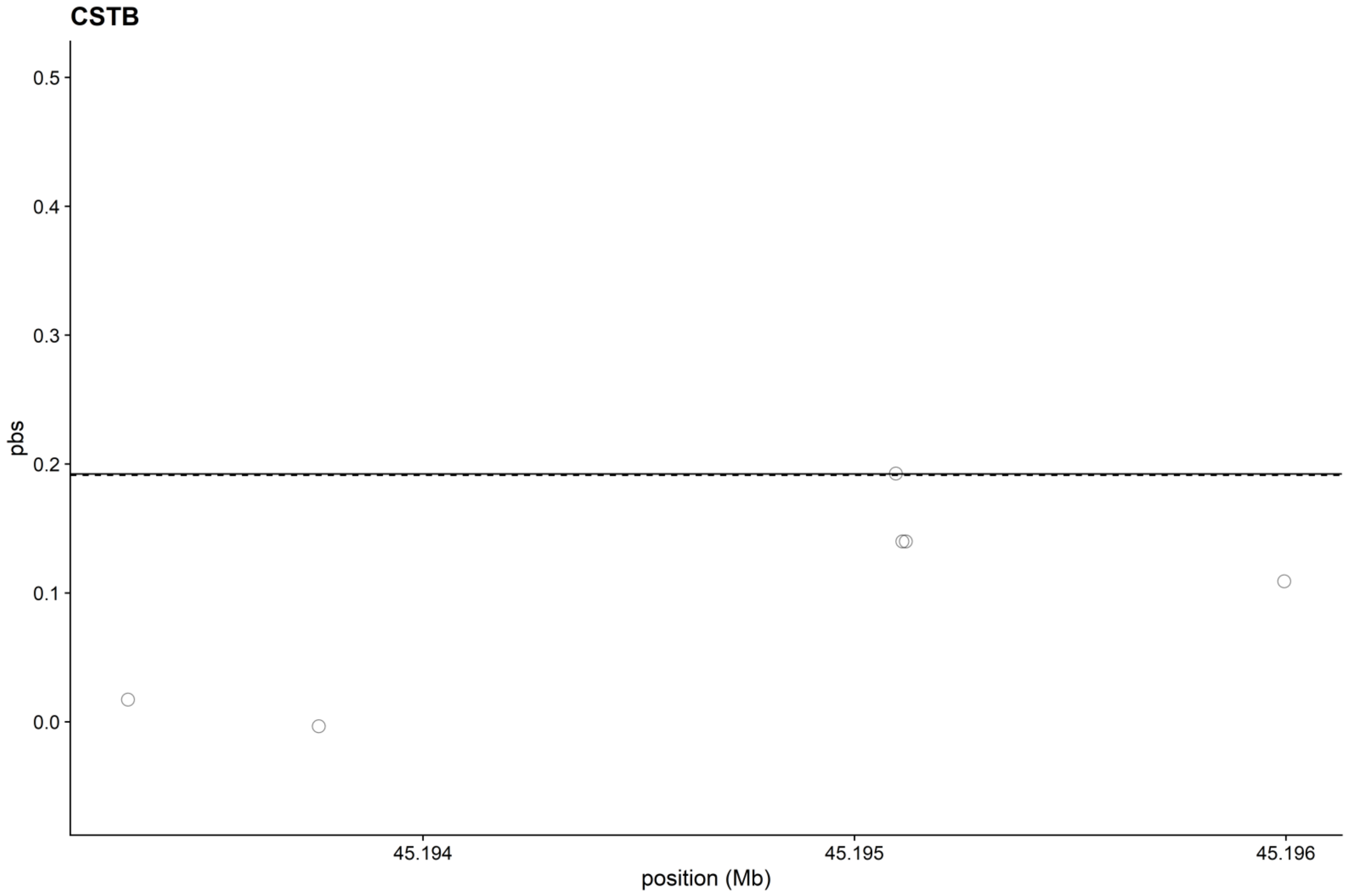

CSTB
pbs
0.5
0.4
0.3
0.2
0.1
0.0
45.194
45.195
45.196
position (Mb)
