# Supplementary material for: A Catalog of Coding Sequence Variations in Salivary Proteins’ Genes Occurring during Recent Human Evolution
Source: Int J Mol Sci. 2023 Oct 9;24(19):15010. doi: 10.3390/ijms241915010 (PMC10573131; doi:10.3390/ijms241915010)
Supplement: Supplementary file 1 [file ijms-24-15010-s001.zip › Table S1.pdf]

Table S1. Nucleotide Substitutions in *PRB1* gene in introns and regulatory regions

Genomic Coordinates (hg19)	Gene Region	Modern human	Altai Neanderthal (Variant Frequency)	Chagyrskaya Neanderthal (Variant Frequency)	Vindija Neanderthal (Variant Frequency)	Denisovan (Variant Frequency)	Codon → amino acid
**11,508,879**	Upstream regions	**G**	**C** (10%) *	**C** (15%)	**C** (26%)	**C** (26%)	
**11,508,854**	Upstream regions	**C**	**C** (100%)	**T** (11%)	**T** (3%) *	**C** (100%)	
**11,508,728**	Upstream regions	**G**	**A** (1%)*	**A** (13%)	**A** (7 %)*	**G** (100%)	
**11,508,703**	Upstream regions	**G**	**G** (100%)	**A** (11%)	**A** (6%)*	**G** (100%)	
**11,508,622**	Upstream regions	**C**	**C** (100%)	**T** (2%)*	**T** (13%)	**T** (2%)*	
**11,508,557**	Upstream regions	**G**	**G** (100%)	**G** (100%)	**A** (11%)	**G** (100%)	
**11,508,523**	5′ UTR	**G**	**G** (100%)	**G** (100%)	**A** (11%)	**G** (100%)	
**11,508,460**	Exon 1 (Signal)	**A**AC	**G**AC (27%)	**G**AC (17%)	**G**AC (34%)	**G**AC (31%)	UUG→$L_{9(sp)}$ CUG→$L_{9(sp)}$
**11,508,383**	Intron 1	**C**	**C** (100%)	**C** (100%)	**T** (11%)	**C** (100%)	
**11,508,382**	Intron 1	**C**	**C** (98%)	**T** (12%)	**T** (6%)*	**C** (100%)	
**11,508,345**	Intron 1	**C**	**C** (97%)	**T** (13%)	**C** (100%)	**C** (100%)	
**11,508,296**	Intron 1	**C**	**C** (100%)	**T** (13%)	**T** (4%)*	**C** (100%)	
**11,508,289**	Intron 1	**C**	**C** (100%)	**T** (14%)	**T** (5%)*	**C** (100%)	
**11,508,283**	Intron 1	**G**	**G** (100%)	**A** (11%)	**A** (12%)	**G** (100%)	
**11,508,217**	Intron 1	**G**	**G** (100%)	**G** (100%)	**A** (11%)	**G** (100%)	
**11,508,167**	Intron 1	**C**	**C** (98%)	**C** (100%)	**T** (11%)	**C** (100%)	
**11,508,026**	Intron 1	**C**	**C** (100%)	**C** (100%)	**T** (13%)	**C** (100%)	
**11,508,014**	Intron 1	**G**	**G** (100%)	**A** (11%)	**G** (100%)	**G** (100%)	
**11,507,986**	Intron 1	**G**	**G** (100%)	**A** (12%)	**A** (10%)*	**G** (100%)	
**11,507,916**	Intron 1	**G**	**G** (100%)	**A** (11%)	**A** (7%)*	**G** (100%)	
**11,507,833**	Intron 1	**C**	**C** (100%)	**C** (100%)	**T** (11%)	**C** (100%)	
**11,507,830**	Intron 1	**G**	**G** (98%)	**G** (100%)	**A** (11%)	**G** (100%)	
**11,507,811**	Intron 1	**C**	**C** (100%)	**C** (100%)	**T** (11%)	**C** (100%)	
**11,507,810**	Intron 1	**C**	**C** (100%)	**C** (100%)	**T** (11%)	**C** (100%)	
**11,507,801**	Intron 1	**C**	**C** (100%)	**C** (100%)	**T** (11%)	**C** (100%)	
**11,507,768**	Intron 1	**G**	**G** (100%)	**A** (15%)	**A** (5%)*	**G** (100%)	
**11,507,652**	Intron 1	**G**	**G** (100%)	**G** (100%)	**A** (14%)	**G** (100%)	
**11,507,607**	Intron 1	**G**	**G** (100%)	**G** (100%)	**A** (16%)	**G** (100%)	
**11,507,594**	Intron 1	**C**	**C** (100%)	**T** (11%)	**C** (100%)	**C** (100%)	
**11,507,592**	Intron 1	**C**	**C** (97%)	**T** (14%)	**C** (100%)	**C** (100%)	
**11,507,584**	Intron 1	**C**	**C** (100%)	**T** (11%)	**C** (100%)	**C** (100%)	

**11,507,578**	Intron 1	**G**	**G** (100%)	**G** (100%)	**A** (13%)	**G** (100%)	
**11,507,536**	Intron 1	**T**	**A** (16%)	**A** (27%)	**A** (18%)	**A** (20%)	
**11,507,532**	Intron 1	**C**	**C** (100%)	**T** (23%)	**T** (8%)*	**C** (100%)	
**11,507,236**	Intron 2	**C**	**C** (100%)	**T** (4%)*	**T** (11%)	**C** (100%)	
**11,507,234**	Intron 2	**C**	**C** (100%)	**T** (14%)	**T** (8%)*	**C** (100%)	
**11,507,230**	Intron 2	**C**	**T** (3%)*	**T** (13%)	**C** (100%)	**C** (100%)	
**11,507,214**	Intron 2	**C**	**T** (2%)*	**T** (15%)	**C** (100%)	**C** (100%)	
**11,507,202**	Intron 2	**G**	**T** (100%)	**T** (100%)	**T** (100%)	**T** (100%)	
**11,507,199**	Intron 2	**G**	**G** (100%)	**A** (13%)	**G** (100%)	**G** (100%)	
**11,507,196**	Intron 2	**C**	**C** (100%)	**T** (11%)	**T** (8%)*	**C** (100%)	
**11,507,180**	Intron 2	**C**	**C** (100%)	**T** (4%)*	**T** (14%)	**C** (100%)	
**11,507,074**	Intron 2	**A**	**C** (100%)	**C** (100%)	**C** (98%)	**C** (100%)	
**11,506,905**	Exon 3 (II-2)	CC**G**	CC**G** (100%)	CC**A** (11%)	CC**A** (4%)*	CC**G** (100%)	GGC→G_{28} GGU→G_{28}
**11,506,887**	Exon 3 (II-2)	GG**G**	GG**G** (100%)	GG**A** (11%)	GG**G** (100%)	GG**G** (100%)	CCC→P_{34} CCU→P_{34}
**11,506,794**	Exon 3 (II-2)	GG**G**	GG**G** (100%)	GG**A** (12%)	GG**G** (100%)	GG**G** (100%)	CCC→P_{65} CCU→P_{65}
**11,506,785**	Exon 3 (II-2)	CC**C**	CC**T** (4%)*	CC**T** (17%)	CC**C** (100%)	CC**T** (5%)*	GGG→G_{68} GGA→G_{68}
**11,506,749**	Exon 3 (P-E)	TT**T**	TT**C** (17%)	TT**C** (9%)*	TT**T** (100%)	TT**C** (10%)*	AAA→K_{5} AAG→K_{5}
**11,506,533**	Exon 3 (Ps-1)	GT**T**	GT**C** (5%)*	GT**C** (5%)*	GT**C** (4%)*	GT**C** (14%)	CAA→Q_{16} CAG→Q_{16}
**11,506,356**	Exon 3 (Ps-1)	CC**G**	CC**G** (100%)	CC**A** (17%)	CC**A** (17%)	CC**A** (12%)	GGC→G_{75} GGU→G_{75}
**11,506,260**	Exon 3 (Ps-1)	TT**C**	TT**C** (100%)**	TT**T** (18%)	TT**C** (100%)	TT**C** (100%)	AAG→K_{107} AAA→K_{107}
**11,506,245**	Exon 3 (Ps-1)	GG**G**	GG**G** (100%)**	GG**A** (18%)	GG**A** (6%)*	GG**G** (100%)	CCC→P_{112} CCU→P_{112}
**11,506,040**	3′ UTR	**G**	**G** (100%)	**A** (5%)*	**A** (13%)	**G** (100%)	
**11,505,908**	Intron 3	**C**	**C** (100%)	**T** (11%)	**T** (5%)*	**C** (100%)	
**11,505,736**	Intron 3	**G**	**G** (98%)	**A** (13%)	**G** (90%)	**G** (100%)	
**11,505,708**	Intron 3	**G**	**A** (1%)*	**A** (12%)	**A** (6%)*	**G** (100%)	
**11,505,389**	Intron 3	**G**	**G** (100%)	**A** (14%)	**G** (100%)	**G** (100%)	
**11,505,375**	Intron 3	**G**	**A** (65%)	**A** (59%)	**A** (59%)	**A** (47%)	
**11,505,362**	Intron 3	**G**	**G** (100%)	**G** (100%)	**A** (11%)	**G** (100%)	
**11,505,347**	Intron 3	**C**	**C** (100%)	**T** (11%)	**C** (100%)	**C** (100%)	
**11,505,277**	Intron 3	**C**	**C** (100%)	**T** (2%)*	**T** (14%)	**T** (2%)*	
**11,505,249**	Intron 3	**G**	**A** (2%)*	**A** (3%)*	**A** (17%)	**G** (100%)	
**11,505,246**	Intron 3	**G**	**A** (2%)*	**A** (8%)*	**A** (12%)	**G** (100%)	
**11,505,168**	Intron 3	**C**	**C** (100%)	**T** (13%)	**T** (5%)*	**C** (100%)	
**11,505,069**	Intron 3	**G**	**A** (58%)	**A** (46%)	**A** (51%)	**A** (49%)	

**11,504,999**	Intron 3	**C**	**C** (100%)	**T** (4%)*	**T** (21%)	**C** (100%)
**11,504,988**	Intron 3	**C**	**C** (99%)	**T** (11%)	**C** (100%)	**C** (100%)
**11,504,987**	Intron 3	**C**	**T** (1%)*	**T** (4%)*	**T** (17%)	**C** (100%)
**11,504,980**	Intron 3	**C**	**C** (100%)	**T** (12%)	**C** (100%)	**C** (100%)
**11,504,970**	Intron 3	**C**	**C** (100%)	**T** (12%)	**C** (100%)	**C** (100%)
**11,504,952**	Intron 3	**C**	**C** (100%)	**C** (100%)	**T** (15%)	**C** (100%)
**11,504,942**	Intron 3	**C**	**C** (100%)	**C** (100%)	**T** (13%)	**C** (100%)
**11,504,691**	Downstream regions	**C**	**C** (100%)	**T** (13%)	**T** (7%)*	**C** (100%)
**11,504,589**	Downstream regions	**G**	**G** (100%)	**A** (12%)	**A** (5%)*	**G** (100%)
**11,504,563**	Downstream regions	**G**	**G** (100%)	**A** (13%)	**A** (13%)	**G** (100%)
**11,504,561**	Downstream regions	**G**	**G** (100%)	**G** (100%)	**A** (17%)	**G** (100%)
**11,504,548**	Downstream regions	**C**	**C** (100%)	**C** (100%)	**T** (12%)	**C** (100%)
**11,504,493**	Downstream regions	**G**	**G** (100%)	**A** (11%)	**A** (4%)*	**G** (100%)
**11,504,491**	Downstream regions	**G**	**G** (100%)	**A** (12%)	**A** (4%)*	**G** (100%)
**11,504,470**	Downstream regions	**G**	**G** (100%)	**A** (13%)	**G** (100%)	**G** (100%)
**11,504,425**	Downstream regions	**A**	**G** (100%)	**G** (96%)	**G** (91%)	**G** (100%)
**11,504,388**	Downstream regions	**C**	**T** (3%)*	**T** (5%)*	**T** (13%)	**C** (100%)
**11,504,360**	Downstream regions	**G**	**G** (98%)	**A** (17%)	**G** (100%)	**G** (100%)
**11,504,295**	Downstream regions	**C**	**C** (100%)	**C** (100%)	**T** (14%)	**C** (100%)
**11,504,272**	Downstream regions	**G**	**G** (100%)	**G** (100%)	**A** (23%)	**G** (100%)

n.a.: not available; the variant frequency is referred to the percentage of the highlighted base in the sequenced ancient hominine genome, with* frequency≤10% and ** counts<10. In light orange are underlined the variants fixed at 100% in modern human compared to ancient hominines.
