# Supplementary material for: A Catalog of Coding Sequence Variations in Salivary Proteins’ Genes Occurring during Recent Human Evolution
Source: Int J Mol Sci. 2023 Oct 9;24(19):15010. doi: 10.3390/ijms241915010 (PMC10573131; doi:10.3390/ijms241915010)
Supplement: Supplementary file 1 [file ijms-24-15010-s001.zip › Table S10 .pdf]

Table S10. Nucleotide Substitutions in *SMR3B* gene in introns and regulatory regions

Genomic Coordinates (hg19)	Gene Region			Chagyrskaya (Variant Frequency)	Vindija (Variant Frequency)	Denisovan (Variant Frequency)	Codon → amino acid
**71,248,560**	Upstream region	**G**	**G** (98%)	**G** (100%)	**A** (13%)	**G** (100%)	
**71,248,568**	Upstream region	**G**	**G** (100%)	**A** (11%)	**A** (8%)*	**G** (100%)	
**71,248,590**	Upstream region	**C**	**C** (100%)	**T** (11%)	**T** (7%)*	**C** (100%)	
**71,248,861**	5′ UTR	**G**	**G** (100%)	**G** (100%)	**A** (12%)	**G** (100%)	
**71,248,913**	5′ UTR	**C**	**C** (98%)	**C** (100%)	**T** (13%)	**C** (100%)	
**71,248,914**	5′ UTR	**A**	**G** (98%)	**G** (100%)	**G** (96%)	**G** (100%)	
**71,248,933**	Intron 1	**G**	**G** (100%)	**A** (6%)*	**A** (13%)	**G** (100%)	
**71,248,937**	Intron 1	**C**	**C** (100%)	**T** (3%)*	**T** (13%)	**C** (100%)	
**71,249,018**	Intron 1	**G**	**G** (100%)	**A** (11%)	**G** (100%)	**G** (100%)	
**71,249,052**	Intron 1	**C**	**C** (100%)	**C** (100%)	**C** (100%)	**T** (100%)	
**71,249,061**	Intron 1	**C**	**C** (100%)	**C** (100%)	**T** (14%)	**C** (100%)	
**71,249,065**	Intron 1	**C**	**C** (98%)	**T** (3%)*	**T** (16%)	**C** (100%)	
**71,249,140**	Intron 1	**G**	**G** (100%)	**G** (100%)	**A** (11%)	**G** (100%)	
**71,249,145**	Intron 1	**G**	**G** (100%)	**A** (11%)	**A** (5%)*	**G** (100%)	
**71,249,242**	Intron 1	**G**	**G** (100%)	**A** (18%)	**G** (100%)	**G** (100%)	
**71,249,289**	Intron 1	**G**	**G** (99%)	**A** (11%)	**A** (7%)*	**G** (100%)	
**71,249,361**	Intron 1	**C**	**C** (100%)	**C** (100%)	**T** (12%)	**C** (100%)	
**71,249,365**	Intron 1	**C**	**C** (100%)	**T** (11%)	**T** (8%)*	**C** (100%)	
**71,249,426**	Intron 1	**G**	**G** (100%)	**G** (100%)	**A** (12%)	**G** (100%)	
**71,249,434**	Intron 1	**C**	**C** (100%)	**T** (8%)*	**T** (14%)	**C** (100%)	
**71,249,441**	Intron 1	**T**	**T** (100%)	**T** (100%)	**T** (100%)	**A** (100%)	
**71,249,472**	Intron 1	**G**	**A** (3%)*	**A** (7%)*	**A** (11%)	**G** (100%)	
**71,249,476**	Intron 1	**A**	**A** (100%)	**A** (100%)	**A** (100%)	**G** (100%)	
**71,249,484**	Intron 1	**C**	**C** (100%)	**C** (100%)	**T** (17%)	**C** (100%)	
**71,249,487**	Intron 1	**G**	**G** (100%)	**A** (10%)*	**A** (11%)	**G** (100%)	
**71,249,488**	Intron 1	**G**	**G** (100%)	**A** (4%)*	**A** (11%)	**G** (100%)	
**71,249,593**	Intron 1	**C**	**C** (95%)	**T** (3%)*	**T** (16%)	**C** (100%)	
**71,249,604**	Intron 1	**G**	**G** (100%)	**G** (100%)	**A** (11%)	**G** (100%)	
**71,249,629**	Intron 1	**T**	**T** (100%)	**T** (100%)	**T** (100%)	**C** (100%)	
**71,249,671**	Intron 1	**G**	**G** (100%)	**G** (100%)	**A** (12%)	**G** (100%)	

**71,249,733**	Intron 1		**InsT** (78%)	**InsT** (74%)	**InsT** (78%)	**InsT** (7%)*
**71,249,743**	Intron 1	**C**	**C** (100%)	**T** (11%)	**T** (3%)*	**T** (3%)*
**71,249,744**	Intron 1	**C**	**C** (100%)	**T** (11%)	**C** (100%)	**C** (100%)
**71,249,756**	Intron 1	**C**	**C** (100%)	**T** (15%)	**C** (100%)	**C** (100%)
**71,249,836**	Intron 1	**G**	**G** (98%)	**G** (100%)	**A** (11%)	**G** (100%)
**71,249,839**	Intron 1	**T**	**C** (98%)	**C** (100%)	**C** (91%)	**T** (100%)
**71,249,901**	Intron 1	**C**	**C** (100%)	**C** (100%)	**T** (12%)	**C** (100%)
**71,249,908**	Intron 1	**G**	**G** (100%)	**A** (10%)*	**A** (15%)	**G** (100%)
**71,249,921**	Intron 1	**G**	**G** (100%)	**A** (12%)	**A** (3%)*	**G** (100%)
**71,249,966**	Intron 1	**T**	**T** (100%)	**T** (100%)	**C** (57%)	**T** (100%)
**71,250,060**	Intron 1	**C**	**C** (100%)	**G** (4%)*	**G** (43%)	**C** (100%)
**71,250,203**	Intron 1	**C**	**C** (100%)	**T** (7%)*	**T** (15%)	**C** (100%)
**71,250,286**	Intron 2	**G**	**G** (100%)	**A** (13%)	**G** (100%)	**G** (100%)
**71,250,287**	Intron 2	**T**	**T** (100%)	**T** (100%)	**C** (15%)	**T** (100%)
**71,250,308**	Intron 2	**T**	**A** (100%)	**A** (100%)	**A** (100%)	**A** (100%)
**71,250,329**	Intron 2	**C**	**C** (100%)	**T** (5%)*	**T** (12%)	**C** (100%)
**71,250,399**	Intron 2	**A**	**G** (98%)	**G** (100%)	**G** (100%)	**G** (100%)
**71,250,415**	Intron 2	**G**	**G** (100%)	**A** (12%)	**A** (9%)*	**G** (100%)
**71,250,521**	Intron 2	**G**	**A** (2%)*	**G** (100%)	**A** (20%)	**A** (4%)*
**71,250,550**	Intron 2	**C**	**C** (100%)	**T** (11%)	**T** (5%)*	**C** (100%)
**71,250,564**	Intron 2	**G**	**G** (100%)	**A** (7%)*	**A** (13%)	**G** (100%)
**71,250,570**	Intron 2	**C**	**C** (100%)	**T** (13%)	**C** (97%)	**C** (100%)
**71,250,583**	Intron 2	**G**	**G** (99%)	**A** (14%)	**G** (100%)	**G** (100%)
**71,250,657**	Intron 2	**T**	**C** (100%)	**C** (100%)	**C** (88%)	**T** (100%)
**71,250,711**	Intron 2	**G**	**G** (100%)	**A** (13%)	**G** (100%)	**G** (100%)
**71,250,716**	Intron 2	**G**	**A** (100%)	**A** (100%)	**A** (100%)	**G** (100%)
**71,250,743**	Intron 2	**T**	**T** (100%)	**T** (100%)	**C** (11%)	**T** (100%)
**71,250,769**	Intron 2	**G**	**A** (100%)	**A** (100%)	**A** (100%)	**A** (100%)
**71,250,835**	Intron 2	**G**	**G** (100%)	**G** (100%)	**A** (13%)	**G** (100%)
**71,250,977**	Intron 2	**A**	**A** (100%)	**A** (100%)	**A** (100%)	**T** (100%)
**71,251,066**	Intron 2	**C**	**C** (100%)	**T** (11%)	**C** (100%)	**C** (100%)
**71,251,115**	Intron 2	**G**	**G** (100%)	**T** (20%)	**T** (7%)*	**G** (100%)
**71,251,116**	Intron 2	**T**	**delT** (14%)	**T** (100%)	**delT** (32%)	**delT** (46%)
**71,251,117**	Intron 2	**T**	**delT** (12%)	**T** (100%)**	**delT** (11%)	**delT** (43%)
**71,251,137**	Intron 2	**T**	**G** (4%)*	**T** (100%)**	**G** (12%)	**T** (100%)

**71,251,138**	Intron 2	**T**	**A** (4%)*	**T** (100%)**	**A** (12%)	**T** (92%)
**71,251,299**	Intron 2	**T**	**T** (100%)	**T** (100%)	**T** (100%)	**A** (15%)
**71,251,313**	Intron 2	**G**	**G** (100%)	**A** (5%)*	**G** (100%)	**A** (10%)*
**71,251,367**	Intron 2	**A**	**C** (13%)	**C** (18%)	**C** (4%)*	**C** (11%)
**71,251,375**	Intron 2	**C**	**C** (100%)	**T** (11%)	**C** (100%)	**C** (100%)
**71,251,469**	Intron 2		-	-	**InsA** (11%)	-
**71,251,514**	Intron 2	**C**	**C** (100%)	**T** (11%)	**C** (100%)	**C** (100%)
**71,251,609**	Intron 2	**C**	**C** (99%)	**T** (12%)	**T** (3%)*	**C** (100%)
**71,251,669**	Intron 2	**T**	**A** (98%)	**A** (97%)	**A** (100%)	**T** (100%)
**71,251,716**	Intron 2	**C**	**C** (100%)	**T** (11%)	**C** (100%)	**C** (100%)
**71,251,718**	Intron 2	**C**	**C** (100%)	**T** (4%)*	**T** (13%)	**C** (100%)
**71,251,804**	Intron 2	**A**	**G** (95%)	**G** (95%)	**G** (100%)	**A** (100%)
**71,251,815**	Intron 2	**C**	**A** (96%)	**A** (98%)	**A** (100%)	**C** (100%)
**71,251,848**	Intron 2	**G**	**G** (98%)	**A** (12%)	**G** (100%)	**G** (100%)
**71,251,989**	Intron 2		**InsA** (16%)	-	-	**InsA** (20%)
**71,251,990**	Intron 2		**InsA** (16%)	-	**InsA** (29%)	**InsA** (20%)
**71,252,145**	Intron 2	**G**	**G** (100%)	**A** (4%)*	**A** (11%)	**G** (100%)
**71,252,158**	Intron 2		-	-	-	**InsAC** (77%)
**71,252,168**	Intron 2	**C**	**C** (100%)	**C** (100%)	**T** (11%)	**C** (100%)
**71,252,239**	Intron 2	**C**	**C** (100%)	**C** (100%)	**T** (17%)	**C** (100%)
**71,252,240**	Intron 2	**C**	**C** (100%)	**C** (100%)	**T** (17%)	**C** (100%)
**71,252,241**	Intron 2	**G**	**A** (100%)	**A** (100%)	**A** (100%)	**G** (97%)
**71,252,261**	Intron 2	**C**	**C** (100%)	**T** (12%)	**T** (7%)*	**C** (100%)
**71,252,298**	Intron 2	**G**	**G** (100%)	**G** (100%)	**A** (18%)	**G** (100%)
**71,252,310**	Intron 2	**C**	**C** (98%)	**C** (100%)	**C** (100%)	**A** (100%)
**71,252,311**	Intron 2	**G**	**A** (100%)	**A** (100%)	**A** (100%)	**G** (100%)
**71,252,314**	Intron 2	**G**	**G** (98%)	**A** (11%)	**A** (3%)*	**G** (100%)
**71,252,332**	Intron 2	**G**	**G** (100%)	**G** (100%)	**A** (11%)	**G** (100%)
**71,252,376**	Intron 2	**G**	**A** (3%)*	**G** (97%)	**A** (11%)	**G** (100%)
**71,252,448**	Intron 2	**C**	**T** (100%)	**T** (100%)	**T** (100%)	**C** (100%)
**71,252,470**	Intron 2	**G**	**A** (4%)*	**A** (3%)*	**A** (12%)	**G** (100%)
**71,252,537**	Intron 2	**A**	**G** (98%)	**G** (97%)	**G** (98%)	**A** (100%)
**71,252,694**	Intron 2	**C**	**C** (98%)	**T** (3%)*	**T** (11%)	**C** (100%)
**71,252,882**	Intron 2	**C**	**C** (100%)	**T** (12%)	**T** (5%)*	**C** (100%)
**71,252,947**	Intron 2	**T**	**T** (100%)	**T** (100%)	**T** (100%)	**C** (100%)

**71,252,977**	Intron 2	G	**G** (100%)	**A** (5%)*	**A** (13%)	**G** (100%)
**71,252,994**	Intron 2	G	**G** (100%)	**A** (11%)	**G** (100%)	**A** (3%)*
**71,253,001**	Intron 2	G	**G** (98%)	**A** (11%)	**G** (100%)	**G** (100%)
**71,253,017**	Intron 2	G	**G** (100%)	**A** (3%)*	**A** (12%)	**G** (100%)
**71,253,024**	Intron 2	C	**T** (2%)*	**C** (97%)	**T** (11%)	**C** (100%)
**71,253,074**	Intron 2	T	**A** (100%)	**A** (100%)	**A** (100%)	**T** (100%)
**71,253,085**	Intron 2	G	**G** (100%)	**A** (14%)	**G** (100%)	**G** (100%)
**71,253,153**	Intron 2	G	**A** (98%)	**A** (100%)	**A** (100%)	**G** (100%)
**71,253,270**	Intron 2	G	**G** (100%)	**A** (11%)	**A** (8%)*	**G** (100%)
**71,253,281**	Intron 2	G	**A** (2%)*	**G** (100%)	**A** (17%)	**G** (100%)
**71,253,283**	Intron 2	G	**G** (100%)	**G** (100%)	**A** (11%)	**G** (100%)
**71,253,291**	Intron 2	C	**C** (100%)	**C** (100%)	**T** (13%)	**C** (100%)
**71,253,481**	Intron 2	A	**T** (97%)	**T** (96%)	**T** (98%)	**A** (100%)
**71,253,518**	Intron 2	G	**G** (98%)	**A** (13%)	**A** (7%)*	**G** (100%)
**71,253,561**	Intron 2	C	**C** (100%)	**T** (3%)*	**T** (12%)	**C** (100%)
**71,253,609**	Intron 2	C	**C** (100%)	**T** (3%)*	**T** (13%)	**C** (100%)
**71,253,619**	Intron 2	G	**G** (100%)	**A** (11%)	**G** (100%)	**G** (100%)
**71,253,709**	Intron 2	G	**G** (100%)	**G** (100%)	**A** (52%)	**G** (100%)
**71,253,747**	Intron 2	C	**C** (100%)	**T** (11%)	**T** (10%)*	**C** (100%)
**71,253,752**	Intron 2	G	**A** (100%)	**A** (100%)	**A** (100%)	**G** (100%)
**71,253,816**	Intron 2	C	**C** (100%)	**T** (13%)	**C** (100%)	**C** (100%)
**71,253,817**	Intron 2	C	**C** (100%)	**T** (12%)	**C** (100%)	**C** (100%)
**71,253,830**	Intron 2	G	**G** (100%)	**A** (7%)*	**A** (11%)	**G** (100%)
**71,253,893**	Intron 2	G	**A** (2%)*	**G** (100%)	**A** (11%)	**G** (100%)
**71,253,934**	Intron 2	C	**C** (100%)	**T** (13%)	**T** (3%)*	**C** (100%)
**71,253,991**	Intron 2		**InsT** (80%)	**InsT** (47%)	**InsT** (73%)	**InsTT** (92%)
**71,254,001**	Intron 2	A	**T** (2%)*	**T** (19%)	**A** (100%)	**A** (97%)
**71,254,078**	Intron 2	C	**C** (100%)	**T** (11%)	**T** (3%)*	**C** (100%)
**71,254,160**	Intron 2	C	**T** (100%)	**T** (100%)	**T** (100%)	**C** (100%)
**71,254,169**	Intron 2	C	**C** (100%)	**T** (3%)*	**T** (11%)	**C** (100%)
**71,254,246**	Intron 2	G	**G** (100%)	**A** (4%)*	**A** (15%)	**G** (100%)
**71,254,247**	Intron 2	G	**A** (2%)*	**A** (13%)	**A** (12%)	**G** (100%)
**71,254,378**	Intron 2	C	**C** (100%)	**T** (4%)*	**T** (13%)	**C** (100%)
**71,254,420**	Intron 2	T	**C** (98%)	**C** (100%)	**C** (96%)	**T** (100%)
**71,254,472**	Intron 2	C	**C** (100%)	**C** (100%)	**T** (12%)	**C** (100%)

**71,254,486**	Intron 2	**C**	**C** (100%)	**T** (13%)	**T** (4%)*	**C** (100%)	
**71,254,512**	Intron 2	**G**	**G** (98%)	**G** (100%)	**A** (14%)	**G** (100%)	
**71,254,543**	Intron 2	**G**	**G** (100%)	**G** (93%)	**G** (97%)	**T** (100%)	
**71,254,578**	Intron 2	**G**	**G** (100%)	**A** (12%)	**G** (100%)	**G** (100%)	
**71,254,609**	Intron 2	**G**	**G** (100%)	**A** (16%)	**A** (4%)*	**G** (100%)	
**71,254,610**	Intron 2	**G**	**G** (100%)	**A** (11%)	**G** (100%)	**G** (97%)	
**71,254,612**	Intron 2	**C**	**C** (100%)	**C** (100%)	**T** (13%)	**C** (100%)	
**71,254,658**	Intron 2	**G**	**G** (100%)	**A** (14%)	**G** (100%)	**G** (100%)	
**71,254,797**	Intron 2	**G**	**A** (2%)*	**A** (5%)*	**A** (13%)	**G** (100%)	
**71,254,910**	Intron 2	**G**	**G** (100%)	**A** (6%)*	**A** (13%)	**G** (100%)	
**71,254,963**	Intron 2	**C**	**C** (100%)	**C** (100%)	**T** (15%)	**T** (3%)*	
**71,254,997**	Intron 2	**G**	**G** (100%)	**G** (100%)	**A** (16%)	**G** (100%)	
**71,255,001**	Intron 2	**G**	**A** (2%)*	**G** (100%)	**A** (11%)	**G** (100%)	
**71,255,043**	Intron 2	**C**	**C** (98%)	**T** (16%)	**C** (100%)	**C** (100%)	
**71,255,051**	Intron 2	**C**	**T** (100%)	**T** (100%)	**T** (100%)	**C** (100%)	
**71,255,071**	Intron 2	**G**	**G** (100%)	**A** (11%)	**A** (9%)*	**G** (100%)	
**71,255,079**	Intron 2	**C**	**C** (100%)	**T** (5%)*	**T** (13%)	**C** (100%)	
**71,255,081**	Intron 2	**C**	**C** (100%)	**T** (11%)	**T** (9%)*	**C** (100%)	
**71,255,093**	Intron 2	**C**	**C** (100%)	**T** (6%)*	**T** (11%)	**C** (100%)	
**71,255,108**	Intron 2	**C**	**C** (100%)	**T** (13%)	**T** (8%)*	**C** (100%)	
**71,255,118**	Intron 2	**G**	**A** (97%)	**A** (100%)	**A** (90%)	**G** (100%)	
**71,255,119**	Intron 2	**C**	**C** (100%)	**T** (15%)	**T** (11%)**	**C** (100%)	
**71,255,211**	Intron 2	**G**	**G** (100%)	**G** (100%)	**A** (14%)	**G** (100%)	
**71,255,267**	Intron 2	**G**	**A** (2%)*	**G** (97%)	**A** (11%)	**G** (100%)	
**71,255,322**	Intron 2	**C**	**T** (2%)*	**T** (15%)	**C** (100%)	**C** (100%)	
**71,255,370**	Intron 2	**A**	**T** (99%)	**T** (100%)	**T** (100%)	**A** (100%)	
**71,255,427**	Exon 3	**CCG**	CC**A** (6%)*	CC**G** (100%)	CC**A** (11%)	CC**G** (100%)	CCG→P_{12}<br>CCA→P_{12}
**71,255,496**	Exon 3	**GGG**	GG**G** (100%)	GG**A** (11%)	GG**A** (8%)*	GG**G** (100%)	GGG→G_{35}<br>GGA→G_{35}
**71,255,593**	3′ UTR	**G**	**A** (100%)	**A** (100%)	**A** (100%)	**G** (100%)	
**71,255,614**	3′ UTR	**C**	**C** (100%)	**C** (100%)	**T** (19%)	**C** (100%)	
**71,255,623**	3′ UTR	**C**	**C** (100%)	**T** (13%)	**T** (5%)*	**C** (100%)	
**71,255,675**	3′ UTR	**C**	**C** (100%)	**T** (4%)*	**T** (16%)	**C** (100%)	
**71,255,677**	3′ UTR	**C**	**T** (2%)*	**C** (100%)	**T** (15%)	**C** (100%)	
**71,255,679**	3′ UTR	**C**	**C** (100%)	**T** (4%)*	**T** (16%)	**C** (100%)	

**71,255,680**	3′ UTR	**C**	**C** (100%)	**T** (4%)*	**T** (11%)	**C** (100%)
**71,255,719**	3′ UTR	**G**	**G** (100%)	**A** (12%)	**G** (100%)	**G** (100%)
**71,255,739**	3′ UTR	**C**	**C** (100%)	**T** (5%)*	**T** (11%)	**C** (100%)
**71,255,767**	3′ UTR	**A**	**G** (100%)	**G** (97%)	**G** (100%)	**A** (100%)
**71,255,989**	Downstream region	**G**	**G** (100%)	**A** (13%)	**A** (9%)*	**G** (100%)
**71,256,001**	Downstream region	**C**	**C** (100%)	**C** (100%)	**T** (13%)	**C** (100%)
**71,256,002**	Downstream region	**C**	**C** (100%)	**C** (100%)	**T** (13%)	**C** (100%)
**71,256,003**	Downstream region	**C**	**C** (100%)	**T** (4%)*	**T** (13%)	**C** (100%)
**71,256,123**	Downstream region	**G**	**A** (2%)*	**G** (100%)	**A** (11%)	**G** (100%)
**71,256,203**	Downstream region	**C**	**C** (100%)	**T** (4%)*	**T** (17%)	**C** (100%)
**71,256,204**	Downstream region	**C**	**C** (100%)	**T** (4%)*	**T** (17%)	**C** (100%)
**71,256,209**	Downstream region	**G**	**G** (100%)	**G** (100%)	**A** (11%)	**G** (100%)
**71,256,210**	Downstream region	**G**	**G** (100%)	**A** (10%)*	**A** (12%)	**G** (100%)
**71,256,289**	Downstream region	**C**	**C** (100%)	**T** (14%)	**C** (100%)	**C** (100%)
**71,256,397**	Downstream region	**C**	**C** (100%)	**T** (11%)	**T** (6%)*	**C** (100%)

n.a.: not available; the variant frequency is referred to the percentage of the highlighted base in the sequenced ancient hominine genome, with* frequency≤10% and ** counts<10. In light orange are underlined the variants fixed at 100% in modern human compared to ancient hominines.
