# Supplementary material for: A Catalog of Coding Sequence Variations in Salivary Proteins’ Genes Occurring during Recent Human Evolution
Source: Int J Mol Sci. 2023 Oct 9;24(19):15010. doi: 10.3390/ijms241915010 (PMC10573131; doi:10.3390/ijms241915010)
Supplement: Supplementary file 1 [file ijms-24-15010-s001.zip › Table S11.pdf]

Table S11. Nucleotide Substitutions in *CST1* gene in introns and regulatory regions

Genomic Coordinates (hg19)	Gene Region	Modern human	Altai Neanderthal (Variant Frequency)	Chagyrskaya Neanderthal (Variant Frequency)	Vindija Neanderthal (Variant Frequency)	Denisovan (Variant Frequency)	Codon → amino acid
**23,731,947**	Upstream regions	**G**	**A** (2%)*	**G** (100%)	**A** (14%)	**G** (100%)	
**23,731,940**	Upstream regions	**C**	**C** (100%)	**T** (12%)	**T** (7%)*	**C** (100%)	
**23,731,864**	Upstream regions	**G**	**G** (100%)	**G** (100%)	**A** (15%)	**G** (100%)	
**23,731,815**	Upstream regions	**C**	**C** (100%)	**C** (100%)	**T** (13%)	**C** (100%)	
**23,731,810**	Upstream regions	**C**	**C** (100%)	**C** (100%)	**T** (13%)	**C** (100%)	
**23,731,800**	Upstream regions	**C**	**C** (100%)	**T** (13%)	**C** (100%)	**C** (100%)	
**23,731,770**	Upstream regions	**C**	**C** (100%)	**C** (100%)	**T** (17%)	**C** (100%)	
**23,731,769**	Upstream regions	**C**	**T** (2%)*	**T** (8%)*	**T** (17%)	**C** (100%)	
**23,731,741**	Upstream regions	**C**	**C** (100%)	**T** (30%)	**C** (100%)	**C** (100%)	
**23,731,738**	Upstream regions	**C**	**T** (3%)*	**T** (20%)	**C** (100%)	**C** (100%)	
**23,731,721**	Upstream regions	**C**	**T** (87%)	**T** (100%)	**T** (71%)	**T** (95%)	
**23,731,719**	Upstream regions	**C**	**T** (90%)	**T** (100%)	**T** (79%)	**T** (96%)	
**23,731,705**	Upstream regions	**C**	**C** (100%)	**C** (100%)	**T** (13%)	**C** (100%)	
**23,731,665**	Upstream regions	**T**	**G** (100%)	**G** (89%)	**G** (88%)**	**G** (94%)	
**23,731,653**	Upstream regions	**C**	**T** (95%)	**T** (100%)	**T** (100%)**	**T** (100%)	
**23,731,624**	Upstream regions	**C**	**C** (100%)	**T** (9%)*	**T** (17%)	**C** (100%)	
**23,731,620**	Upstream regions	**C**	**C** (100%)	**T** (7%)*	**T** (17%)	**C** (100%)	
**23,731,619**	Upstream regions	**C**	**C** (100%)	**T** (7%)*	**T** (17%)	**C** (100%)	
**23,731,589**	Upstream regions	**G**	**C** (58%)	**C** (60%)	**C** (54%)	**C** (36%)	
**23,731,571**	5′UTR	**G**	**G** (100%)	**G** (100%)	**A** (13%)	**G** (100%)	
**23,731,563**	5′UTR	**G**	**G** (100%)	**A** (11%)	**A** (5%)*	**G** (100%)	
**23,731,560**	5′UTR	**C**	**T** (69%)	**T** (47%)	**T** (42%)	**T** (56%)	
**23,731,557**	5′UTR	**G**	**G** (100%)	**G** (100%)	**A** (11%)	**G** (100%)	
**23,731,551**	5′UTR	**G**	**G** (100%)	**A** (19%)	**A** (5%)*	**G** (100%)	
**23,731,550**	5′UTR	**G**	**G** (100%)	**A** (13%)	**A** (5%)*	**G** (100%)	
**23,731,527**	5′UTR	**A**	**G** (34%)	**G** (21%)	**G** (43%)	**G** (21%)	
**23,731,479**	Exon 1 (Signal)	**GAC**	GAC (100%)	AAC (11%)	AAC (4%)*	GAC (100%)	CUG→$L_{8(sp)}$ UUG→$L_{8(sp)}$
**23,731,453**	Exon 1 (Signal)	**CAC**	CAC (98%)	CAC (100%)	CAT (11%)	CAC (100%)	GUG→$V_{16(sp)}$ GUA→$V_{16(sp)}$

**23,731,363**	Exon 1	**CGG**	CG**G** (100%)	CG**A** (12%)	CG**G** (100%)	CG**G** (100%)	GCC→A_{27}<br>GCU→A_{27}
**23,731,354**	Exon 1	**AAG**	AA**A** (5%)*	AA**A** (13%)	AA**G** (10%)	AA**G** (10%)	UUC→F_{30}<br>UUU→F_{30}
**23,731,294**	Exon 1	**CAT**	CA**A** (57%)	CA**T** (100%)	CA**A** (52%)	CA**T** (100%)	GUA→V_{50}<br>GUU→V_{50}
**23,731,282**	Exon 1	**TCC**	TC**C** (100%)	TC**C** (100%)	TC**T** (13%)	TC**C** (100%)	AGG→R_{54}<br>AGA→R_{54}
**23,731,254**	Intron 1	**C**	**C** (100%)	**C** (100%)	**T** (11%)	**C** (100%)	
**23,731,251**	Intron 1	**C**	**C** (100%)	**T** (6%)*	**T** (13%)	**C** (100%)	
**23,731,248**	Intron 1	**G**	**G** (100%)	**G** (100%)	**A** (11%)	**G** (100%)	
**23,731,123**	Intron 1	**C**	**G** (2%)*	**T** (14%)	**C** (100%)	**C** (100%)	
**23,731,102**	Intron 1	**C**	**C** (100%)	**T** (13%)	**T** (15%)	**C** (100%)	
**23,731,048**	Intron 1	**G**	**G** (100%)	**A** (3%)*	**A** (11%)	**G** (100%)	
**23,731,032**	Intron 1	**C**	**C** (100%)	**C** (100%)	**T** (13%)	**C** (100%)	
**23,731,017**	Intron 1	**C**	**C** (100%)	**C** (100%)	**T** (14%)	**T** (3%)*	
**23,731,007**	Intron 1	**C**	**C** (100%)	**T** (4%)*	**T** (11%)	**C** (100%)	
**23,731,005**	Intron 1	**C**	**C** (100%)	**C** (96%)	**T** (14%)	**C** (100%)	
**23,730,890**	Intron 1	**C**	**C** (100%)	**C** (100%)	**T** (14%)	**C** (100%)	
**23,730,888**	Intron 1	**C**	**C** (100%)	**T** (4%)*	**T** (11%)	**C** (100%)	
**23,730,886**	Intron 1	**G**	**G** (100%)	**A** (22%)	**A** (11%)	**G** (100%)	
**23,730,885**	Intron 1	**G**	**G** (100%)	**A** (5%)*	**A** (11%)	**G** (100%)	
**23,730,873**	Intron 1	**C**	**C** (100%)	**C** (100%)	**T** (15%)	**C** (100%)	
**23,730,867**	Intron 1	**G**	**G** (100%)	**A** (7%)*	**A** (25%)	**G** (100%)	
**23,730,841**	Intron 1	**C**	**C** (100%)	**C** (100%)	**T** (18%)	**C** (100%)	
**23,730,803**	Intron 1	**C**	**C** (100%)	**C** (100%)	**T** (14%)	**C** (100%)	
**23,730,798**	Intron 1	**C**	**C** (100%)	**T** (11%)	**C** (100%)	**C** (100%)	
**23,730,797**	Intron 1	**C**	**C** (100%)	**T** (6%)*	**T** (13%)	**C** (100%)	
**23,730,777**	Intron 1	**C**	**C** (100%)	**T** (12%)	**T** (5%)*	**C** (100%)	
**23,730,775**	Intron 1	**C**	**C** (100%)	**T** (12%)	**T** (11%)	**C** (100%)	
**23,730,772**	Intron 1	**C**	**C** (100%)	**T** (13%)	**C** (100%)	**C** (100%)	
**23,730,761**	Intron 1	**G**	**G** (100%)	**A** (19%)	**A** (8%)*	**G** (100%)	
**23,730,760**	Intron 1	**G**	**G** (100%)	**A** (13%)	**A** (18%)	**G** (100%)	
**23,730,754**	Intron 1	**G**	**G** (100%)	**A** (11%)	**G** (100%)	**G** (100%)	
**23,730,747**	Intron 1	**G**	**G** (100%)	**G** (100%)	**A** (14%)	**G** (100%)	
**23,730,692**	Intron 1	**C**	**C** (100%)	**T** (13%)	**T** (11%)*	**C** (100%)	
**23,730,682**	Intron 1	**C**	**C** (100%)	**T** (13%)	**T** (14%)**	**T** (4%)*	
**23,730,677**	Intron 1	**C**	**C** (97%)	**T** (13%)	**C** (100%)	**C** (100%)	

**23,730,674**	Intron 1	**C**	**C** (100%)	**T** (13%)	**C** (100%)	**C** (100%)	
**23,730,629**	Intron 1	**C**	**C** (100%)	**T** (6%)*	**T** (13%)	**C** (100%)	
**23,730,625**	Intron 1	**G**	**G** (100%)	**G** (100%)	**C** (4%)*	**C** (32%)	
**23,730,607**	Intron 1	**G**	**G** (100%)	**A** (5%)*	**A** (13%)	**G** (100%)	
**23,730,549**	Intron 1	**C**	**C** (98%)	**T** (16%)	**T** (6%)*	**C** (97%)	
**23,730,536**	Intron 1	**G**	**G** (100%)	**A** (11%)	**A** (3%)*	**G** (100%)	
**23,730,522**	Intron 1	**C**	**C** (100%)	**T** (17%)	**T** (3%)*	**C** (100%)	
**23,730,494**	Intron 1	**G**	**G** (98%)	**A** (13%)	**A** (4%)*	**G** (100%)	
**23,730,475**	Intron 1	**C**	**C** (100%)	**T** (7%)*	**T** (11%)	**C** (100%)	
**23,730,436**	Intron 1	**C**	**C** (100%)	**C** (94%)	**T** (20%)	**C** (100%)	
**23,730,434**	Intron 1	**C**	**C** (100%)	**C** (100%)	**T** (18%)	**C** (100%)	
**23,730,234**	Intron 1	**G**	**A** (2%)*	**G** (100%)	**A** (18%)	**G** (100%)	
**23,730,227**	Intron 1	**C**	**C** (100%)	**C** (100%)	**T** (14%)	**C** (100%)	
**23,730,034**	Intron 1	**G**	**G** (100%)	**G** (100%)	**A** (11%)	**G** (100%)	
**23,729,973**	Intron 1	**A**	**A** (100%)	**G** (41%)	**G** (55%)	**A** (100%)	
**23,729,898**	Intron 1	**C**	**C** (100%)	**T** (13%)	**C** (100%)	**T** (5%)*	
**23,729,871**	Intron 1	**C**	**T** (2%)*	**T** (4%)*	**T** (11%)	**C** (100%)	
**23,729,870**	Intron 1	**C**	**T** (2%)*	**T** (11%)	**C** (100%)	**C** (100%)	
**23,729,858**	Intron 1	**C**	**C** (100%)	**T** (12%)	**T** (5%)*	**C** (100%)	
**23,729,857**	Intron 1	**G**	**G** (97%)	**G** (100%)	**A** (11%)	**G** (100%)	
**23,729,837**	Intron 1	**G**	**G** (100%)	**G** (100%)	**G** (100%)	**A** (47%)	
**23,729,814**	Intron 1	**C**	**C** (100%)	**T** (3%)*	**T** (11%)	**C** (100%)	
**23,729,812**	Intron 1	**C**	**C** (100%)	**C** (100%)	**T** (14%)	**C** (100%)	
**23,729,793**	Intron 1	**G**	**A** (4%)*	**A** (4%)*	**A** (14%)	**A** (4%)*	
**23,729,764**	Exon 2	**TGG**	TGG (98%)	TGA (14%)	TGA (4%)*	TGG (100%)	ACC→T_{57}<br>ACG→T_{57}
**23,729,737**	Exon 2	**CTG**	CTG (100%)	CTA (13%)	CTA (9%)*	CTA (3%)*	GAC→D_{66}<br>GAU→D_{66}
**23,729,722**	Exon 2	**GCG**	GCT (48%)	GCT (45%)	GCT (54%)	GCT (44%)	CGC→R_{71}<br>CGA→R_{71}
**23,729,689**	Exon 2	**CTG**	CTG (100%)	CTA (3%)*	CTA (13%)	CTG (100%)	GAC→D_{82}<br>GAU→D_{82}
**23,729,635**	Intron 2	**C**	**C** (100%)	**T** (4%)*	**T** (13%)	**C** (100%)	
**23,729,616**	Intron 2	**G**	**G** (100%)	**A** (11%)	**A** (3%)*	**G** (100%)	
**23,729,609**	Intron 2	**C**	**C** (100%)	**C** (100%)	**T** (13%)	**C** (100%)	
**23,729,602**	Intron 2	**C**	**T** (3%)*	**C** (100%)	**T** (13%)	**C** (100%)	
**23,729,584**	Intron 2	**G**	**G** (100%)	**G** (100%)	**G** (100%)	**A** (87%)	
**23,729,579**	Intron 2	**T**	**G** (10%)*	**T** (100%)	**G** (16%)	**T** (100%)	

**23,729,567**	Intron 2	**C**	**C** (100%)	**C** (100%)	**T** (20%)	**C** (100%)
**23,729,556**	Intron 2		**InsAC** (50%)	**InsAC** (40%)**	**InsAC** (23%)	**InsAC** (56%)**
**23,729,550**	Intron 2	**C**	**C** (100%)	**C** (100%)	**A** (20%)	**C** (100%)
**23,729,549**	Intron 2	**C**	**C** (100%)	**C** (100%)	**A** (20%)	**C** (100%)
**23,729,544**	Intron 2	**C**	**T** (26%)	**C** (100%)**	**T** (33%)*	**C** (100%)**
**23,729,514**	Intron 2	**C**	**T** (31%)	**T** (14%)**	**T** (17%)**	**T** (25%)**
**23,729,500**	Intron 2	**T**	**C** (42%)	**C** (45%)	**C** (56%)**	**C** (30%)
**23,729,496**	Intron 2	**C**	**T** (48%)	**T** (55%)	**T** (50%)**	**T** (45%)
**23,729,486**	Intron 2	**T**	**G** (63%)	**G** (54%)	**G** (63%)**	**G** (62%)
**23,729,484**	Intron 2	**C**	**C** (100%)	**T** (13%)	**T** (9%)*	**C** (100%)
**23,729,481**	Intron 2	**C**	**C** (100%)	**T** (7%)*	**T** (17%)	**C** (100%)
**23,729,464**	Intron 2	**C**	**C** (100%)	**T** (12%)	**C** (100%)	**T** (5%)*
**23,729,449**	Intron 2	**G**	**G** (100%)	**A** (13%)	**G** (100%)	**G** (100%)
**23,729,373**	Intron 2	**G**	**G** (100%)	**G** (92%)	**T** (60%)	**G** (100%)
**23,729,337**	Intron 2	**A**	**A** (100%)	**G** (70%)	**G** (26%)	**A** (100%)
**23,729,302**	Intron 2	**C**	**C** (100%)	**T** (13%)	**T** (5%)*	**C** (97%)
**23,729,301**	Intron 2	**C**	**C** (100%)	**T** (13%)	**C** (100%)	**C** (96%)
**23,729,299**	Intron 2	**C**	**C** (100%)	**T** (13%)	**C** (100%)	**T** (4%)*
**23,729,298**	Intron 2	**C**	**C** (100%)	**T** (13%)	**T** (5%)*	**C** (100%)
**23,729,296**	Intron 2	**C**	**C** (100%)	**T** (13%)	**T** (11%)	**C** (97%)
**23,729,287**	Intron 2	**C**	**C** (100%)	**T** (6%)*	**T** (11%)	**C** (100%)
**23,729,286**	Intron 2	**C**	**C** (100%)	**T** (6%)*	**T** (11%)	**C** (100%)
**23,729,264**	Intron 2	**C**	**C** (100%)	**C** (100%)	**T** (17%)	**C** (100%)
**23,729,253**	Intron 2	**C**	**C** (100%)	**T** (3%)*	**T** (17%)	**C** (100%)
**23,729,232**	Intron 2	**C**	**C** (100%)	**T** (5%)*	**T** (13%)	**C** (100%)
**23,729,187**	Intron 2	**C**	**A** (100%)	**A** (100%)**	**A** (100%)**	**A** (100%)**
**23,729,184**	Intron 2	**C**	**G** (100%)	**G** (100%)**	**G** (100%)**	**G** (100%)**
**23,729,134**	Intron 2	**G**	**C** (100%)	**C** (93%)	**C** (90%)	**C** (100%)
**23,729,117**	Intron 2	**G**	**G** (100%)	**A** (11%)	**A** (4%)*	**G** (100%)
**23,729,090**	Intron 2	**A**	**A** (100%)	**A** (100%)	**A** (100%)	**G** (76%)
**23,729,056**	Intron 2	**C**	**C** (100%)	**C** (100%)	**T** (12%)	**C** (100%)
**23,729,045**	Intron 2	**G**	**G** (100%)	**A** (11%)	**G** (100%)	**G** (100%)
**23,729,041**	Intron 2	**G**	**G** (100%)	**A** (6%)*	**A** (13%)	**G** (100%)
**23,729,029**	Intron 2	**G**	**G** (98%)	**G** (96%)	**A** (14%)	**G** (100%)
**23,728,986**	Intron 2	**C**	**T** (87%)	**T** (74%)	**T** (64%)	**C** (100%)

**23,728,979**	Intron 2	**T**	**C** (6%)*	**C** (43%)	**C** (9%)*	**T** (100%)	
**23,728,951**	Intron 2	**G**	**G** (100%)	**A** (13%)	**G** (100%)	**G** (100%)	
**23,728,934**	Intron 2	**C**	**C** (100%)	**T** (4%)*	**T** (15%)	**C** (100%)	
**23,728,927**	Intron 2	**G**	**G** (98%)	**G** (100%)	**A** (12%)	**G** (100%)	
**23,728,925**	Intron 2	**G**	**G** (100%)	**A** (13%)	**G** (100%)	**G** (100%)	
**23,728,910**	Intron 2	**G**	**G** (100%)	**A** (14%)	**A** (4%)*	**G** (100%)	
**23,728,909**	Intron 2	**G**	**G** (100%)	**A** (11%)	**A** (9%)*	**G** (100%)	
**23,728,893**	Intron 2	**C**	**C** (100%)	**T** (13%)	**T** (11%)	**C** (100%)	
**23,728,890**	Intron 2	**G**	**G** (100%)	**A** (12%)	**G** (100%)	**G** (100%)	
**23,728,882**	Intron 2	**C**	**T** (87%)	**T** (65%)	**T** (68%)	**C** (100%)	
**23,728,881**	Intron 2	**C**	**C** (100%)	**T** (6%)*	**T** (11%)	**C** (100%)	
**23,728,878**	Intron 2	**G**	**G** (100%)	**G** (100%)	**A** (11%)	**G** (100%)	
**23,728,873**	Intron 2	**C**	**T** (2%)*	**C** (100%)	**T** (11%)	**C** (100%)	
**23,728,872**	Intron 2	**C**	**C** (100%)	**T** (5%)*	**T** (13%)	**C** (100%)	
**23,728,805**	Intron 2	**G**	**G** (100%)	**A** (13%)	**A** (11%)	**G** (100%)	
**23,728,797**	Intron 2	**T**	**C** (11%)	**T** (100%)	**C** (25%)	**T** (100%)	
**23,728,751**	Intron 2	**C**	**C** (100%)	**T** (4%)*	**T** (11%)	**C** (100%)	
**23,728,749**	Intron 2	**C**	**C** (98%)	**T** (11%)	**C** (100%)	**C** (100%)	
**23,728,719**	Intron 2	**A**	**A** (95%)	**A** (96%)	**A** (100%)	**delA** (21%)	
**23,728,687**	Intron 2	**G**	**G** (98%)	**A** (5%)*	**A** (14%)	**G** (100%)	
**23,728,661**	Intron 2	**C**	**C** (100%)	**T** (11%)	**C** (100%)	**C** (100%)	
**23,728,658**	Intron 2	**C**	**C** (100%)	**T** (6%)*	**T** (20%)	**C** (100%)	
**23,728,656**	Intron 2	**C**	**C** (100%)	**T** (6%)*	**T** (13%)	**C** (100%)	
**23,728,649**	Intron 2	**C**	**C** (100%)	**C** (100%)	**T** (11%)	**C** (100%)	
**23,728,648**	Intron 2	**C**	**C** (100%)	**T** (9%)*	**T** (18%)	**C** (100%)	
**23,728,641**	Intron 2	**C**	**T** (8%)*	**T** (5%)*	**T** (14%)	**C** (100%)	
**23,728,639**	Intron 2	**G**	**G** (100%)	**G** (100%)	**A** (13%)	**G** (100%)	
**23,728,635**	Intron 2	**G**	**G** (100%)	**A** (12%)	**A** (15%)	**G** (100%)	
**23,728,557**	Intron 2	**G**	**G** (100%)	**A** (11%)	**A** (9%)*	**G** (100%)	
**23,728,556**	Intron 2	**G**	**G** (100%)	**A** (11%)	**A** (9%)*	**G** (100%)	
**23,728,550**	Intron 2	**G**	**G** (100%)	**G** (100%)	**A** (15%)	**G** (100%)	
**23,728,547**	Intron 2	**G**	**G** (100%)	**A** (11%)	**A** (4%)*	**G** (100%)	
**23,728,546**	Intron 2	**G**	**G** (100%)	**A** (11%)	**G** (100%)	**G** (100%)	
**23,728,486**	Exon 3	**TCC**	TCC (100%)	TCT (4%)*	TCT (17%)	TCC (100%)	AGG→R_{111}<br>AGA→R_{111}

**23,728,419**	3′UTR	**G**	**G** (100%)	**G** (100%)	**A** (20%)	**G** (100%)
**23,728,418**	3′UTR	**G**	**G** (100%)	**A** (10%)*	**A** (20%)	**G** (100%)
**23,728,417**	3′UTR	**G**	**G** (100%)	**A** (27%)	**G** (100%)	**G** (100%)
**23,728,359**	3′UTR	**C**	**A** (15%)	**A** (22%)**	**A** (13%)	**C** (100%)
**23,728,341**	3′UTR	**C**	**C** (100%)	**C** (100%)	**T** (11%)	**C** (95%)
**23,728,332**	3′UTR	**G**	**G** (100%)	**G** (100%)	**A** (11%)	**G** (100%)
**23,728,314**	3′UTR	**C**	**C** (100%)	**C** (100%)	**T** (13%)	**C** (100%)
**23,728,301**	3′UTR	**C**	**C** (100%)	**T** (15%)	**C** (100%)	**C** (100%)
**23,728,291**	3′UTR	**C**	**C** (100%)	**T** (12%)	**C** (100%)	**C** (100%)
**23,728,238**	3′UTR	**G**	**G** (100%)	**G** (100%)	**A** (14%)	**G** (100%)
**23,728,233**	3′UTR	**G**	**T** (100%)	**T** (89%)**	**T** (90%)	**T** (100%)**
**23,728,222**	3′UTR	**G**	**G** (100%)	**G** (100%)	**A** (14%)	**G** (100%)
**23,728,157**	Downstream regions	**C**	**T** (100%)	**T** (100%)**	**T** (100%)	**T** (100%)
**23,728,128**	Downstream regions	**G**	**G** (96%)	**A** (14%)	**G** (100%)	**G** (100%)
**23,728,103**	Downstream regions	**G**	**C** (25%)	**C** (35%)	**C** (42%)	**G** (100%)
**23,728,101**	Downstream regions	**C**	**C** (100%)	**C** (100%)	**T** (13%)	**C** (100%)
**23,728,094**	Downstream regions	**G**	**G** (100%)	**A** (3%)*	**A** (14%)	**G** (100%)
**23,728,083**	Downstream regions	**G**	**A** (23%)	**A** (25%)	**A** (43%)	**A** (5%)*
**23,728,058**	Downstream regions	**C**	**T** (3%)*	**T** (38%)	**T** (60%)	**C** (100%)
**23,728,056**	Downstream regions	**C**	**C** (100%)	**C** (100%)	**T** (15%)	**C** (100%)
**23,728,055**	Downstream regions	**G**	**G** (100%)	**G** (100%)	**A** (15%)	**G** (94%)
**23,727,966**	Downstream regions	**C**	**T** (3%)*	**T** (11%)	**C** (100%)	**C** (100%)
**23,727,961**	Downstream regions	**C**	**T** (8%)*	**C** (100%)	**T** (13%)	**C** (100%)
**23,727,953**	Downstream regions	**C**	**C** (100%)	**T** (10%)*	**T** (14%)	**C** (100%)
**23,727,940**	Downstream regions	**G**	**G** (100%)	**A** (12%)	**A** (5%)*	**G** (100%)
**23,727,928**	Downstream regions	**G**	**G** (100%)	**A** (26%)	**G** (100%)	**G** (100%)
**23,727,927**	Downstream regions	**G**	**G** (100%)	**A** (11%)	**A** (4%)*	**G** (100%)
**23,727,917**	Downstream regions	**T**	**C** (100%)	**C** (100%)	**C** (100%)	**C** (100%)**
**23,727,909**	Downstream regions	**C**	**C** (100%)	**C** (100%)	**T** (13%)	**C** (100%)
**23,727,891**	Downstream regions	**C**	**C** (100%)	**C** (100%)	**T** (20%)	**C** (100%)
**23,727,890**	Downstream regions	**C**	**C** (100%)	**C** (100%)	**T** (13%)	**C** (100%)
**23,727,884**	Downstream regions	**G**	**G** (100%)	**A** (13%)	**G** (100%)	**G** (100%)
**23,727,875**	Downstream regions	**C**	**C** (100%)	**T** (5%)*	**T** (13%)	**C** (100%)

**23,727,874**	Downstream regions	**C**	**C** (100%)	**C** (100%)	**T** (25%)	**C** (100%)
**23,727,849**	Downstream regions	**C**	**C** (97%)	**T** (12%)	**C** (100%)	**C** (100%)
**23,727,828**	Downstream regions	**C**	**G** (96%)	**G** (91%)	**G** (90%)	**G** (95%)
**23,727,761**	Downstream regions	**T**	**T** (100%)	**C** (13%)	**T** (100%)	**T** (100%)
**23,727,757**	Downstream regions	**G**	**G** (100%)	**A** (4%)*	**A** (12%)	**G** (100%)
**23,727,756**	Downstream regions	**G**	**G** (100%)	**A** (4%)*	**A** (16%)	**G** (100%)
**23,727,735**	Downstream regions	**G**	**A** (100%)	**A** (100%)	**A** (100%)	**G** (100%)
**23,727,707**	Downstream regions	**C**	**C** (100%)	**T** (12%)	**C** (100%)	**C** (100%)
**23,727,697**	Downstream regions	**T**	**G** (100%)	**G** (86%) **A** (14%)	**G** (92%)	**G** (100%)
**23,727,695**	Downstream regions	**G**	**G** (100%)	**A** (14%)	**A** (4%)*	**G** (100%)
**23,727,628**	Downstream regions	**C**	**C** (100%)	**T** (4%)*	**T** (11%)	**C** (100%)

n.a.: not available; the variant frequency is referred to the percentage of the highlighted base in the sequenced ancient hominine genome, with* frequency≤10% and ** counts<10. In light orange are underlined the variants fixed at 100% in modern human compared to ancient hominines.
