# Supplementary material for: A Catalog of Coding Sequence Variations in Salivary Proteins’ Genes Occurring during Recent Human Evolution
Source: Int J Mol Sci. 2023 Oct 9;24(19):15010. doi: 10.3390/ijms241915010 (PMC10573131; doi:10.3390/ijms241915010)
Supplement: Supplementary file 1 [file ijms-24-15010-s001.zip › Table S12 .pdf]

Table S12. Nucleotide Substitutions in *CST2* gene in introns and regulatory regions

Genomic Coordinates (hg19)	Gene Region	Modern human	Altai Neanderthal (Variant Frequency)	Chagyrskaya Neanderthal (Variant Frequency)	Vindija Neanderthal (Variant Frequency)	Denisovan (Variant Frequency)	Codon → amino acid
**23,807,611**	Upstream region	**C**	**C** (100%)	**T** (15%)	**T** (3%)*	**C** (100%)	
**23,807,587**	Upstream region	**C**	**C** (100%)	**C** (100%)	**T** (11%)	**C** (100%)	
**23,807,544**	Upstream region	**G**	**G** (100%)	**G** (100%)	**A** (11%)	**G** (100%)	
**23,807,531**	Upstream region	**C**	**C** (100%)	**T** (11%)	**T** (6%)*	**C** (100%)	
**23,807,528**	Upstream region	**C**	**C** (100%)	**T** (12%)	**C** (100%)	**C** (100%)	
**23,807,515**	Upstream region	**C**	**C** (100%)	**C** (100%)	**T** (14%)	**C** (100%)	
**23,807,512**	Upstream region	**C**	**T** (2%)*	**T** (14%)	**T** (5%)*	**C** (100%)	
**23,807,491**	Upstream region	**C**	**C** (100%)	**C** (100%)	**T** (14%)	**C** (100%)	
**23,807,480**	Upstream region	**C**	**C** (100%)	**T** (4%)*	**T** (14%)	**C** (100%)	
**23,807,447**	Upstream region	**C**	**C** (100%)	**C** (100%)	**T** (17%)	**C** (100%)	
**23,807,423**	Upstream region	**C**	**C** (100%)	**C** (100%)	**T** (17%)	**C** (100%)	
**23,807,422**	Upstream region	**C**	**C** (100%)	**T** (5%)*	**T** (18%)	**C** (100%)	
**23,807,416**	Upstream region	**C**	**C** (100%)	**C** (100%)	**T** (11%)	**C** (100%)	
**23,807,363**	Upstream region	**G**	**G** (100%)	**A** (11%)	**G** (100%)	**G** (100%)	
**23,807,360**	Upstream region	**G**	**G** (98%)	**A** (13%)	**G** (100%)	**G** (100%)	
**23,807,259**	Exon 1 (Signal)	CG**G**	CG**G** (100%)	CG**A** (8%)*	CG**A** (21%)	CG**G** (100%)	GCC→$A_{12(sp)}$ GCU→ $A_{12(sp)}$
**23,807,241**	Exon 1 (Signal)	GA**C**	GA**G** (93%)	GA**G** (100%)	GA**G** (82%)	GA**C** (100%)	CUG→$L_{18(sp)}$ CUC→$L_{18(sp}$
**23,807,151**	Exon 1	GT**G**	GT**G** (100%)	GT**A** (13%)	GT**G** (100%)	GT**G** (100%)	CAC→H_{29} CAU→H_{29}
**23,807,145**	Exon 1	CA**G**	CA**G** (100%)	CA**A** (13%)	CA**A** (7%)*	CA**G** (100%)	GUC→V_{31} GUU→V_{31}
**23,807,100**	Exon 1	GC**G**	GC**G** (100%)	GC**A** (4%)*	GC**A** (12%)	GC**G** (100%)	CGC→R_{46} CGU→R_{46}
**23,807,076**	Exon 1	TC**C**	TC**C** (100%)	TC**C** (100%)	TC**T** (13%)	TC**C** (100%)	AGG→R_{54} AGA→R_{54}
**23,807,038**	Intron 1	**C**	**C** (100%)	**T** (15%)	**C** (100%)	**C** (100%)	
**23,807,028**	Intron 1	**A**	**C** (100%)	**C** (100%)	**C** (95%)	**C** (97%)	
**23,807,019**	Intron 1	**G**	**G** (100%)	**A** (14%)	**A** (4%)*	**G** (100%)	
**23,807,018**	Intron 1	**G**	**G** (100%)	**A** (14%)	**A** (4%)*	**G** (100%)	
**23,806,929**	Intron 1	**A**	**A** (100%)	**A** (95%)	**A** (100%)	**C** (100%)	
**23,806,880**	Intron 1	**G**	**G** (100%)	**G** (100%)	**A** (12%)	**G** (100%)	
**23,806,827**	Intron 1	**G**	**G** (100%)	**A** (15%)	**A** (5%)*	**G** (100%)	

**23,806,821**	Intron 1	**G**	**G** (100%)	**G** (100%)	**A** (14%)	**G** (100%)
**23,806,811**	Intron 1	**G**	**G** (100%)	**G** (100%)	**A** (12%)	**G** (100%)
**23,806,715**	Intron 1	**G**	**G** (100%)	**G** (100%)	**A** (14%)	**G** (100%)
**23,806,695**	Intron 1	**G**	**G** (100%)	**A** (12%)	**G** (100%)	**G** (100%)
**23,806,690**	Intron 1	**G**	**A** (3%)*	**G** (100%)	**A** (18%)	**G** (100%)
**23,806,685**	Intron 1	**G**	**G** (100%)	**A** (12%)	**A** (17%)	**G** (100%)
**23,806,655**	Intron 1	**C**	**C** (100%)	**C** (100%)	**T** (15%)	**C** (100%)
**23,806,653**	Intron 1	**C**	**C** (100%)	**C** (100%)	**T** (12%)	**C** (100%)
**23,806,536**	Intron 1	**T**	**T** (100%)	**T** (100%)	**T** (100%)	**C** (100%)
**23,806,513**	Intron 1	**G**	**G** (100%)	**A** (13%)	**G** (100%)	**G** (100%)
**23,806,507**	Intron 1	**A**	**G** (100%)	**G** (100%)	**A** (13%)	**G** (100%)
**23,806,506**	Intron 1	**G**	**A** (3%)*	**G** (100%)	**A** (17%)	**G** (100%)
**23,806,468**	Intron 1	**G**	**G** (100%)	**A** (17%)	**A** (9%)*	**G** (100%)
**23,806,466**	Intron 1	**G**	**G** (100%)	**G** (100%)	**A** (13%)	**G** (100%)
**23,806,452**	Intron 1	**A**	**G** (97%)	**G** (100%)	**G** (89%)	**A** (96%)
**23,806,416**	Intron 1	**G**	**G** (100%)	**A** (11%)	**A** (7%)*	**G** (100%)
**23,806,387**	Intron 1	**G**	**G** (100%)	**A** (25%)	**A** (4%)*	**G** (100%)
**23,806,383**	Intron 1	**C**	**C** (100%)	**T** (17%)	**T** (9%)*	**C** (100%)
**23,806,310**	Intron 1	**C**	**C** (100%)	**C** (100%)	**T** (12%)	**T** (3%)*
**23,806,290**	Intron 1	**C**	**C** (100%)	**T** (11%)	**C** (100%)	**T** (4%)*
**23,806,278**	Intron 1	**C**	**C** (100%)	**C** (100%)	**T** (18%)	**C** (100%)
**23,806,274**	Intron 1	**C**	**C** (100%)	**T** (6%)*	**T** (11%)	**C** (100%)
**23,806,273**	Intron 1	**C**	**C** (100%)	**C** (100%)	**T** (11%)	**C** (100%)
**23,806,183**	Intron 1	**A**	**A** (100%)	**A** (100%)	**A** (100%)	**G** (100%)
**23,806,108**	Intron 1	**C**	**C** (100%)	**C** (100%)	**T** (15%)	**C** (100%)
**23,806,105**	Intron 1	**C**	**C** (100%)	**C** (100%)	**T** (18%)	**C** (100%)
**23,806,095**	Intron 1	**C**	**G** (76%)	**G** (22%)	**C** (100%)	**C** (100%)
**23,806,079**	Intron 1	**G**	**G** (98%)	**A** (11%)	**G** (100%)	**G** (100%)
**23,806,071**	Intron 1	**G**	**G** (100%)	**G** (97%)	**A** (12%)	**G** (100%)
**23,806,039**	Intron 1	**G**	**G** (100%)	**A** (4%)*	**G** (100%)	**A** (28%)
**23,805,961**	Intron 1	**C**	**T** (2%)*	**T** (14%)	**C** (100%)	**C** (100%)
**23,805,846**	Intron 2	**C**	**C** (100%)	**T** (6%)*	**T** (11%)	**C** (100%)
**23,805,832**	Intron 2	**G**	**A** (92%)	**A** (93%)	**A** (92%)	**A** (97%)
**23,805,800**	Intron 2	**C**	**C** (100%)	**T** (4%)*	**T** (11%)	**C** (100%)
**23,805,798**	Intron 2	**C**	**C** (100%)	**T** (11%)	**C** (94%)	**C** (100%)

**23,805,785**	Intron 2	**A**	**G** (14%)	**G** (3%)*	**A** (100%)	**G** (4%)*
**23,805,773**	Intron 2	**T**	**T** (100%)	**G** (11%)	**G** (6%)*	**G** (2%)*
**23,805,769**	Intron 2	**C**	**C** (100%)	**T** (11%)	**C** (100%)	**C** (100%)
**23,805,758**	Intron 2	**C**	**C** (100%)	**T** (3%)*	**T** (20%)	**C** (100%)
**23,805,754**	Intron 2	**C**	**C** (100%)	**C** (100%)	**T** (24%)	**C** (100%)
**23,805,750**	Intron 2		**InsAC** (25%)	**InsAC** (6%)*	**InsAC** (9%)*	**InsAC** (15%)
**23,805,746**	Intron 2	**C**	**C** (100%)	**T** (10%)*	**T** (15%)	**C** (100%)
**23,805,738**	Intron 2	**C**	**T** (44%)	**T** (13%)	**T** (26%)	**C** (100%)
**23,805,720**	Intron 2	**T**	**C** (49%)	**C** (67%)	**C** (48%)	**C** (59%)
**23,805,712**	Intron 2	**T**	**C** (46%)	**C** (31%)	**C** (45%)	**C** (36%)
**23,805,686**	Intron 2	**A**	**C** (24%)	**C** (12%)	**C** (27%)	**C** (10%)*
**23,805,680**	Intron 2	**T**	**G** (23%)	**G** (9%)*	**G** (24%)	**G** (7%)*
**23,805,674**	Intron 2	**T**	**C** (18%)	**C** (6%)*	**C** (23%)	**C** (3%)*
**23,805,632**	Intron 2	**C**	**C** (98%)	**C** (100%)	**T** (13%)	**C** (100%)
**23,805,630**	Intron 2	**C**	**C** (100%)	**T** (19%)	**T** (6%)*	**C** (100%)
**23,805,599**	Intron 2	**G**	**G** (98%)	**A** (15%)	**A** (3%)*	**G** (100%)
**23,805,548**	Intron 2	**C**	**C** (98%)	**C** (100%)	**C** (100%)	**G** (88%)
**23,805,532**	Intron 2	**C**	**T** (2%)*	**C** (100%)	**T** (11%)	**T** (5%)*
**23,805,507**	Intron 2	**C**	**C** (100%)	**C** (100%)	**T** (11%)	**C** (100%)
**23,805,506**	Intron 2	**C**	**C** (100%)	**C** (100%)	**T** (11%)	**C** (100%)
**23,805,505**	Intron 2	**C**	**C** (100%)	**T** (13%)	**T** (17%)	**C** (100%)
**23,805,497**	Intron 2	**C**	**C** (100%)	**C** (100%)	**T** (11%)	**C** (100%)
**23,805,494**	Intron 2	**C**	**C** (98%)	**T** (6%)*	**T** (13%)	**C** (100%)
**23,805,491**	Intron 2	**C**	**C** (100%)	**T** (6%)*	**T** (11%)	**C** (100%)
**23,805,490**	Intron 2	**C**	**C** (100%)	**T** (6%)*	**T** (11%)	**C** (100%)
**23,805,489**	Intron 2	**C**	**C** (100%)	**T** (12%)	**T** (11%)	**C** (100%)
**23,805,487**	Intron 2	**C**	**C** (100%)	**C** (100%)	**T** (11%)	**C** (100%)
**23,805,469**	Intron 2	**C**	**C** (100%)	**T** (17%)	**C** (100%)	**C** (100%)
**23,805,468**	Intron 2	**C**	**C** (100%)	**T** (11%)	**T** (4%)*	**C** (100%)
**23,805,465**	Intron 2	**C**	**C** (100%)	**T** (11%)	**C** (100%)	**C** (100%)
**23,805,464**	Intron 2	**G**	**G** (100%)	**A** (13%)	**G** (100%)	**G** (100%)
**23,805,409**	Intron 2	**G**	**G** (100%)	**A** (23%)	**A** (4%)*	**G** (100%)
**23,805,369**	Intron 2	**C**	**C** (100%)	**C** (100%)	**T** (13%)	**C** (100%)
**23,805,365**	Intron 2	**C**	**C** (100%)	**T** (14%)	**C** (100%)	**C** (100%)
**23,805,356**	Intron 2	**C**	**C** (100%)	**T** (10%)*	**T** (13%)	**C** (100%)

**23,805,329**	Intron 2	**G**	**G** (100%)	**A** (6%)*	**A** (15%)	**G** (100%)
**23,805,283**	Intron 2	**C**	**C** (100%)	**C** (100%)	**T** (20%)	**C** (100%)
**23,805,282**	Intron 2	**C**	**C** (100%)	**T** (7%)*	**T** (20%)	**C** (100%)
**23,805,280**	Intron 2	**G**	**G** (100%)	**A** (19%)	**G** (100%)	**G** (100%)
**23,805,269**	Intron 2	**C**	**T** (2%)*	**T** (17%)	**C** (100%)	**C** (100%)
**23,805,258**	Intron 2	**G**	**G** (100%)	**A** (15%)	**G** (100%)	**G** (100%)
**23,805,176**	Intron 2	**G**	**G** (100%)	**G** (100%)	**A** (20%)	**G** (100%)
**23,805,167**	Intron 2	**G**	**G** (98%)	**G** (100%)	**A** (11%)	**G** (100%)
**23,805,154**	Intron 2	**G**	**G** (98%)	**A** (14%)	**A** (8%)*	**G** (100%)
**23,805,057**	Intron 2	**C**	**G** (4%)*	**G** (6%)*	**T** (14%)<br>**G** (11%)	**C** (100%)
**23,805,032**	Intron 2	**G**	**G** (100%)	**A** (10%)*	**A** (11%)	**G** (100%)
**23,805,026**	Intron 2	**C**	**C** (100%)	**C** (100%)	**T** (11%)	**C** (100%)
**23,804,923**	Intron 2	**A**	**A** (100%)	**A** (100%)	**A** (100%)	**delA** (76%)
**23,804,918**	Intron 2	**C**	**T** (4%)*	**T** (12%)	**T** (10%)*	**C** (100%)
**23,804,864**	Intron 2	**C**	**T** (2%)*	**T** (11%)	**T** (5%)*	**C** (100%)
**23,804,863**	Intron 2	**C**	**C** (100%)	**T** (12%)	**T** (6%)*	**C** (100%)
**23,804,860**	Intron 2	**C**	**C** (100%)	**C** (100%)	**T** (21%)	**C** (100%)
**23,804,858**	Intron 2	**C**	**T** (5%)*	**T** (17%)	**C** (100%)	**C** (100%)
**23,804,856**	Intron 2	**C**	**T** (2%)*	**T** (15%)	**C** (100%)	**C** (100%)
**23,804,854**	Intron 2	**C**	**C** (100%)	**T** (15%)	**T** (14%)	**C** (100%)
**23,804,843**	Intron 2	**G**	**G** (100%)	**A** (11%)	**A** (10%)*	**G** (100%)
**23,804,839**	Intron 2	**G**	**G** (98%)	**A** (11%)	**A** (20%)	**G** (100%)
**23,804,838**	Intron 2	**G**	**G** (100%)	**A** (11%)	**A** (9%)*	**G** (100%)
**23,804,828**	Intron 2	**G**	**G** (100%)	**G** (100%)	**A** (13%)	**G** (100%)
**23,804,794**	Intron 2	**G**	**G** (100%)	**A** (12%)	**A** (6%)*	**G** (100%)
**23,804,750**	Intron 2	**G**	**G** (100%)	**A** (11%)	**A** (6%)*	**G** (100%)
**23,804,655**	3′UTR	**C**	**C** (100%)	**C** (100%)	**T** (13%)	**C** (100%)
**23,804,647**	3′UTR	**G**	**G** (100%)	**G** (100%)	**A** (17%)	**G** (100%)
**23,804,612**	3′UTR	**G**	**G** (100%)	**A** (15%)	**A** (10%)*	**G** (100%)
**23,804,575**	3′UTR	**G**	**A** (3%)*	**A** (18%)	**G** (100%)**	**G** (100%)
**23,804,515**	3′UTR	**G**	**G** (100%)	**A** (13%)	**G** (100%)	**G** (100%)
**23,804,501**	3′UTR	**G**	**G** (100%)	**A** (14%)	**G** (100%)	**G** (100%)
**23,804,465**	3′UTR	**G**	**G** (98%)	**G** (100%)	**A** (14%)	**G** (96%)
**23,804,408**	3′UTR	**T**	**C** (11%)	**C** (4%)*	**T** (100%)	**C** (5%)*

**23,804,370**	Downstream region	**T**	**C** (25%)	**C** (21%)	**C** (23%)	**C** (19%)
**23,804,355**	Downstream region	**C**	**C** (98%)	**T** (3%)*	**T** (4%)*	**T** (68%)
**23,804,286**	Downstream region	**C**	**C** (100%)	**C** (100%)	**T** (11%)	**C** (100%)
**23,804,245**	Downstream region	**C**	**C** (100%)	**C** (100%)	**T** (15%)	**C** (100%)
**23,804,180**	Downstream region	**C**	**C** (100%)	**T** (13%)	**T** (6%)*	**C** (100%)
**23,804,169**	Downstream region	**C**	**C** (100%)	**T** (20%)	**C** (100%)	**C** (100%)
**23,804,167**	Downstream region	**C**	**C** (100%)	**C** (100%)	**T** (15%)	**C** (100%)
**23,804,157**	Downstream region	**C**	**C** (100%)	**C** (100%)	**T** (18%)	**C** (100%)
**23,804,155**	Downstream region	**C**	**C** (100%)	**T** (8%)*	**T** (20%)	**C** (100%)
**23,804,147**	Downstream region	**G**	**G** (100%)	**A** (6%)*	**A** (18%)	**G** (100%)
**23,804,131**	Downstream region	**C**	**C** (100%)	**T** (11%)	**T** (5%)*	**C** (100%)
**23,804,128**	Downstream region	**C**	**C** (100%)	**T** (15%)	**C** (100%)	**C** (100%)
**23,804,089**	Downstream region	**C**	**C** (100%)	**C** (100%)	**T** (17%)	**C** (100%)
**23,804,080**	Downstream region	**C**	**C** (100%)	**T** (11%)	**C** (100%)	**C** (100%)
**23,804,991**	Downstream region	**G**	**G** (100%)	**A** (3%)*	**A** (12%)	**G** (100%)
**23,804,983**	Downstream region	**C**	**T** (2%)*	**T** (11%)	**T** (3%)*	**C** (98%)
**23,804,931**	Downstream region	**C**	**C** (98%)	**T** (4%)*	**T** (13%)	**C** (100%)

n.a.: not available; the variant frequency is referred to the percentage of the highlighted base in the sequenced ancient hominine genome, with* frequency≤10% and ** counts<10. In light orange are underlined the variants fixed at 100% in modern human compared to ancient hominines.
