# Supplementary material for: A Catalog of Coding Sequence Variations in Salivary Proteins’ Genes Occurring during Recent Human Evolution
Source: Int J Mol Sci. 2023 Oct 9;24(19):15010. doi: 10.3390/ijms241915010 (PMC10573131; doi:10.3390/ijms241915010)
Supplement: Supplementary file 1 [file ijms-24-15010-s001.zip › Table S13 .pdf]

Table S13. Nucleotide Substitutions in *CST3* gene in introns and regulatory regions

Genomic Coordinates (hg19)	Gene Region	Modern human	Altai Neanderthal (Variant Frequency)	Chagyrskaya Neanderthal (Variant Frequency)	Vindija Neanderthal (Variant Frequency)	Denisovan (Variant Frequency)	Codon → amino acid
**23,619,036**	Upstream region	**C**	**C** (100%)	**C** (100%)	**T** (17%)	**C** (100%)	
**23,619,022**	Upstream region	**C**	**C** (100%)	**T** (11%)**	**T** (22%)	**C** (100%)	
**23,619,003**	Upstream region	**C**	**C** (100%)	**T** (14%)	**C** (100%)	**C** (100%)	
**23,619,002**	Upstream region	**C**	**C** (100%)	**T** (14%)	**C** (100%)	**C** (100%)	
**23,618,976**	Upstream region	**C**	**C** (100%)	**T** (13%)	**T** (56%)**	**C** (100%)	
**23,618,938**	Upstream region	**C**	**C** (100%)	**T** (8%)*	**T** (18%)	**C** (100%)	
**23,618,934**	Upstream region	**C**	**C** (100%)	**C** (100%)	**T** (13%)	**C** (100%)	
**23,618,932**	Upstream region	**C**	**C** (100%)	**T** (20%)	**T** (20%)	**C** (100%)	
**23,618,846**	Upstream region	**C**	**C** (100%)	**C** (100%)	**T** (30%)	**C** (100%)**	
**23,618,833**	Upstream region	**C**	**T** (4%)*	**T** (20%)	**C** (100%)	**C** (100%)**	
**23,618,820**	Upstream region	**C**	**C** (100%)	**T** (17%)	**T** (8%)*	**C** (100%)	
**23,618,815**	Upstream region	**C**	**C** (100%)	**T** (20%)	**T** (20%)	**C** (100%)	
**23,618,803**	Upstream region	**C**	**C** (100%)	**T** (13%)	**C** (100%)	**C** (100%)	
**23,618,783**	Upstream region	**C**	**C** (100%)	**C** (100%)	**T** (13%)	**C** (100%)**	
**23,618,782**	Upstream region	**C**	**C** (100%)	**C** (100%)	**T** (13%)	**C** (100%)**	
**23,618,781**	Upstream region	**C**	**C** (100%)	**C** (100%)	**T** (13%)	**C** (100%)**	
**23,618,779**	Upstream region	**C**	**C** (100%)	**C** (100%)	**T** (21%)	**C** (100%)**	
**23,618,778**	Upstream region	**C**	**C** (100%)	**C** (100%)	**T** (15%)	**C** (100%)**	
**23,618,659**	5′ UTR	**C**	**C** (100%)	**T** (18%)	**C** (100%)	**C** (100%)	
**23,618,656**	5′ UTR	**C**	**C** (100%)	**T** (17%)	**C** (100%)	**C** (100%)	
**23,618,645**	5′ UTR	**C**	**C** (100%)	**C** (100%)	**T** (12%)	**C** (100%)	
**23,618,644**	5′ UTR	**C**	**C** (100%)	**C** (100%)	**T** (12%)	**C** (100%)	
**23,618,641**	5′ UTR	**C**	**C** (100%)	**T** (15%)	**C** (100%)	**C** (100%)	
**23,618,633**	5′ UTR	**G**	**G** (100%)	**G** (100%)	**A** (17%)	**G** (100%)	
**23,618,578**	5′ UTR	**C**	**C** (100%)	**T** (13%)	**T** (18%)	**C** (93%)	
**23,618,532**	5′ UTR	**G**	**G** (100%)	**A** (6%)*	**A** (15%)	**G** (100%)	
**23,618,516**	5′ UTR	**G**	**G** (100%)	**A** (8%)*	**A** (14%)	**G** (100%)	
**23,618,491**	Exon 1 (Signal)	CCC	CCC (100%)	CCT (14%)**	CCT (18%)	CCC (100%)	GGG→$G_{2(sp)}$ GGA→$G_{2(sp)}$
**23,618,422**	Exon 1 (Signal)	CCG	CCG (100%)	CCA (11%)	CCA (11%)	CCG (100%)**	GGC→$G_{25(sp)}$ GGU→$G_{25(sp)}$

**23,618,407**	Exon 1	TT**C**	TT**C** (100%)	TT**T** (12%)	TT**C** (100%)	TT**C** (100%)**	AAG→K_5<br>AAA→K_5
**23,618,398**	Exon 1	GC**G**	GC**G** (100%)	GC**G** (100%)	GC**A** (13%)	GC**G** (100%)**	CGC→R_8<br>CGU→R_8
**23,618,397**	Exon 1	**G**AT	**G**AT (100%)	**G**AT (100%)	**A**AT (13%)	**G**AT (100%)**	CUA→L_9<br>UUA→L_9
**23,618,395**	Exon 1	GA**T**	GA**C** (100%)	GA**C** (95%)	GA**C** (11%)	GA**C** (100%)**	CUA→L_9<br>CUG→L_9
**23,618,296**	Exon 1	AT**G**	AT**G** (100%)	AT**G** (100%)	AT**A** (13%)	AT**G** (100%)	UAC→Y_{42}<br>UAU→Y_{42}
**23,618,253**	Intron 1	**G**	**G** (100%)	**A** (18%)	**G** (100%)	**G** (100%)**	
**23,618,231**	Intron 1	**C**	**C** (100%)	**C** (100%)	**T** (30%)	**C** (100%)**	
**23,618,206**	Intron 1	**G**	**G** (100%)	**G** (100%)	**A** (18%)	**G** (100%)**	
**23,618,193**	Intron 1	**C**	**C** (100%)	**T** (8%)*	**T** (13%)	**C** (100%)	
**23,618,162**	Intron 1	**C**	**C** (100%)	**T** (17%)	**C** (100%)	**C** (100%)	
**23,618,108**	Intron 1	**G**	**G** (100%)	**G** (100%)	**A** (11%)	**G** (100%)	
**23,618,092**	Intron 1	**C**	**C** (100%)	**C** (100%)	**T** (13%)	**C** (100%)	
**23,618,086**	Intron 1	**C**	**C** (100%)	**T** (17%)	**T** (13%)	**C** (100%)	
**23,618,081**	Intron 1	**C**	**C** (100%)	**C** (100%)	**T** (18%)	**C** (100%)	
**23,618,079**	Intron 1	**C**	**T** (3%)*	**C** (100%)	**T** (14%)	**C** (100%)	
**23,618,040**	Intron 1	**C**	**C** (100%)	**T** (14%)**	**T** (14%)	**C** (100%)	
**23,617,987**	Intron 1	**C**	**T** (4%)*	**C** (100%)	**T** (11%)	**C** (100%)	
**23,617,982**	Intron 1	**C**	**C** (100%)	**T** (15%)	**C** (100%)	**C** (100%)	
**23,617,972**	Intron 1	**C**	**T** (3%)*	**T** (7%)*	**T** (11%)	**C** (100%)	
**23,617,948**	Intron 1	**C**	**C** (100%)	**T** (12%)	**C** (96%)	**C** (100%)	
**23,617,907**	Intron 1	**C**	**C** (100%)	**T** (18%)	**T** (3%)*	**C** (100%)	
**23,617,897**	Intron 1	**C**	**C** (100%)	**T** (11%)	**C** (100%)	**C** (100%)	
**23,617,878**	Intron 1	**G**	**G** (100%)	**A** (13%)	**G** (97%)	**G** (100%)	
**23,617,864**	Intron 1	**C**	**C** (100%)	**T** (9%)*	**T** (15%)	**C** (100%)	
**23,617,647**	Intron 1	**C**	**T** (2%)*	**C** (100%)	**T** (12%)	**C** (100%)	
**23,617,599**	Intron 1	**C**	**C** (100%)	**T** (5%)*	**T** (20%)	**C** (100%)	
**23,617,463**	Intron 1	**G**	**G** (100%)	**G** (100%)	**A** (15%)	**G** (100%)	
**23,617,438**	Intron 1	**G**	**A** (4%)*	**A** (13%)	**A** (6%)*	**A** (6%)*	
**23,617,435**	Intron 1	**G**	**C** (100%)	**A** (14%)	**G** (100%)	**C** (100%)	
**23,617,426**	Intron 1	**C**	**C** (100%)	**C** (100%)	**T** (14%)	**C** (100%)	
**23,617,413**	Intron 1	**G**	**G** (100%)	**G** (100%)	**A** (17%)	**G** (100%)	
**23,617,341**	Intron 1	**C**	**C** (100%)	**T** (12%)	**T** (10%)*	**C** (100%)	
**23,617,322**	Intron 1	**C**	**T** (2%)*	**T** (14%)	**T** (4%)*	**C** (100%)	
**23,617,261**	Intron 1	**G**	**G** (100%)	**A** (12%)	**A** (7%)*	**G** (100%)	

**23,617,256**	Intron 1	**G**	**A** (5%)*	**A** (6%)*	**A** (12%)	**G** (100%)
**23,617,236**	Intron 1	**G**	**G** (100%)	**G** (100%)	**A** (11%)	**G** (100%)
**23,617,197**	Intron 1	**C**	**C** (100%)	**T** (17%)	**T** (4%)*	**C** (100%)
**23,617,191**	Intron 1	**G**	**G** (100%)	**A** (8%)*	**A** (12%)	**A** (7%)*
**23,617,190**	Intron 1	**G**	**G** (100%)	**G** (90%)	**A** (13%)	**G** (100%)
**23,617,189**	Intron 1	**G**	**G** (100%)	**G** (100%)	**A** (13%)	**A** (7%)*
**23,617,188**	Intron 1	**G**	**G** (100%)	**G** (100%)	**A** (13%)	**A** (7%)*
**23,617,160**	Intron 1	**G**	**G** (100%)	**G** (100%)	**A** (20%)	**G** (100%)
**23,617,143**	Intron 1	**G**	**G** (100%)	**G** (100%)	**A** (17%)	**G** (100%)
**23,617,138**	Intron 1	**G**	**G** (100%)	**G** (100%)	**A** (18%)	**G** (100%)
**23,617,121**	Intron 1	**C**	**C** (100%)	**C** (100%)	**T** (15%)	**C** (100%)
**23,617,094**	Intron 1	**G**	**G** (100%)	**A** (13%)	**G** (100%)	**G** (100%)
**23,617,084**	Intron 1	**G**	**G** (100%)	**A** (13%)	**G** (100%)	**G** (100%)
**23,617,037**	Intron 1	**G**	**A** (2%)*	**G** (100%)	**A** (11%)	**G** (100%)
**23,617,007**	Intron 1	**G**	**G** (100%)	**G** (100%)	**A** (10%)*	**A** (100%)
**23,617,002**	Intron 1	**C**	**C** (100%)	**T** (14%)	**A** (9%)*	**C** (100%)
**23,617,001**	Intron 1	**C**	**C** (100%)	**T** (7%)*	**T** (18%)	**C** (100%)
**23,616,980**	Intron 1	**C**	**C** (100%)	**C** (100%)	**T** (13%)	**C** (100%)
**23,616,978**	Intron 1	**G**	**G** (100%)	**G** (100%)	**A** (12%)	**G** (100%)
**23,616,970**	Intron 1	**C**	**T** (3%)*	**C** (100%)	**T** (12%)	**C** (100%)
**23,616,940**	Intron 1	**C**	**T** (2%)*	**T** (4%)*	**T** (13%)	**C** (100%)
**23,616,914**	Intron 1	**G**	**G** (100%)	**A** (13%)	**G** (100%)	**G** (100%)
**23,616,876**	Intron 1	**C**	**C** (100%)	**C** (100%)	**T** (11%)	**C** (100%)
**23,616,862**	Intron 1	**G**	**A** (3%)*	**A** (12%)	**G** (100%)	**G** (100%)
**23,616,844**	Intron 1	**G**	**G** (100%)	**A** (14%)	**G** (100%)	**G** (100%)
**23,616,835**	Intron 1	**C**	**C** (100%)	**C** (100%)	**T** (13%)	**C** (100%)
**23,616,822**	Intron 1	**G**	**A** (100%)	**A** (100%)	**A** (100%)	**G** (100%)
**23,616,807**	Intron 1	**G**	**G** (100%)	**G** (100%)	**A** (5%)*	**A** (100%)
**23,616,712**	Intron 1	**G**	**G** (100%)	**G** (100%)	**A** (11%)	**G** (100%)
**23,616,645**	Intron 1	**C**	**C** (97%)	**T** (12%)	**C** (100%)	**C** (100%)
**23,616,631**	Intron 1	**C**	**C** (100%)	**T** (4%)*	**T** (15%)	**C** (100%)
**23,616,630**	Intron 1	**C**	**C** (100%)	**T** (9%)*	**T** (14%)	**C** (100%)
**23,616,618**	Intron 1	**G**	**A** (24%)	**A** (12%)	**A** (52%)	**G** (100%)
**23,616,587**	Intron 1	**A**	**G** (40%)	**G** (34%)	**G** (48%)	**G** (39%)
**23,616,585**	Intron 1	**A**	**C** (41%)	**C** (28%)	**C** (46%)	**C** (39%)

**23,616,581**	Intron 1	**C**	**C** (100%)	**C** (100%)	**T** (14%)	**C** (100%)	
**23,616,577**	Intron 1	**G**	**G** (98%)	**A** (11%)	**G** (100%)	**G** (100%)	
**23,616,552**	Intron 1	**G**	**T** (37%)	**T** (37%)	**T** (46%)	**T** (41%)	
**23,616,492**	Intron 1	**A**	**A** (100%)	**A** (100%)	**A** (100%)	**C** (83%)	
**23,616,469**	Intron 1	**T**	**T** (98%)	**T** (100%)	**T** (100%)	**C** (97%)	
**23,616,425**	Intron 1	**C**	**C** (100%)	**T** (16%)	**T** (5%)*	**C** (100%)	
**23,616,372**	Intron 1	**C**	**T** (1%)*	**T** (4%)*	**T** (11%)	**C** (100%)	
**23,616,348**	Intron 1	**G**	**G** (100%)	**G** (97%)	**A** (17%)	**G** (100%)	
**23,616,277**	Intron 1	**C**	**C** (100%)	**T** (5%)*	**T** (16%)	**C** (100%)	
**23,616,270**	Intron 1	**G**	**G** (100%)	**A** (28%)	**G** (100%)	**G** (100%)	
**23,616,256**	Intron 1	**C**	**C** (100%)	**C** (100%)	**T** (13%)	**C** (100%)	
**23,616,250**	Intron 1	**C**	**C** (100%)	**T** (11%)	**C** (100%)	**C** (100%)	
**23,616,206**	Intron 1	**G**	**A** (100%)	**A** (100%)	**A** (100%)	**A** (100%)	
**23,616,167**	Intron 1	**C**	**T** (2%)*	**T** (5%)*	**T** (14%)	**C** (100%)	
**23,616,150**	Intron 1	**C**	**T** (5%)*	**T** (14%)	**T** (8%)*	**C** (100%)	
**23,616,144**	Intron 1	**G**	**G** (100%)	**A** (14%)	**G** (100%)	**G** (100%)	
**23,616,083**	Intron 1	**G**	**A** (2%)*	**G** (100%)	**A** (15%)	**A** (4%)*	
**23,616,051**	Intron 1	**T**	**T** (100%)	**T** (100%)	**T** (100%)	**C** (100%)	
**23,616,008**	Intron 1	**C**	**C** (100%)	**C** (100%)	**T** (11%)	**C** (100%)	
**23,615,936**	Exon 2	GG<u>**G**</u>	GG<u>**G**</u> (100%)	GG<u>**A**</u> (5%)*	GG<u>**A**</u> (17%)	GG<u>**G**</u> (95%)	CCC→P_{78}<br>CCU→P_{78}
**23,615,886**	Intron 2	**C**	**T** (3%)*	**C** (100%)	**T** (15%)	**C** (100%)	
**23,615,883**	Intron 2	**G**	**G** (100%)	**A** (10%)*	**A** (13%)	**G** (100%)	
**23,615,882**	Intron 2	**G**	**G** (100%)	**G** (100%)	**A** (13%)	**G** (100%)	
**23,615,849**	Intron 2	**C**	**C** (100%)	**C** (86%)	**G** (11%)	**C** (100%)	
**23,615,846**	Intron 2	**C**	**C** (100%)	**C** (100%)	**T** (18%)	**C** (100%)	
**23,615,843**	Intron 2	**A**	**A** (100%)	**A** (100%)	**C** (21%)	**A** (100%)	
**23,615,820**	Intron 2	**CA**	**InsCACA** (35%)	**-**	**-**	**delCA** (40%)	
**23,615,775**	Intron 2	**C**	**C** (100%)	**T** (4%)*	**T** (13%)	**C** (100%)	
**23,615,772**	Intron 2	**G**	**G** (100%)	**A** (11%)	**G** (96%)	**G** (100%)	
**23,615,739**	Intron 2	**C**	**C** (100%)	**T** (13%)	**C** (100%)	**C** (100%)	
**23,615,728**	Intron 2	**C**	**T** (2%)*	**C** (100%)	**T** (13%)	**C** (100%)	
**23,615,723**	Intron 2	**C**	**C** (100%)	**C** (100%)	**T** (20%)	**C** (100%)	
**23,615,711**	Intron 2	**C**	**T** (2%)*	**T** (4%)*	**T** (14%)	**C** (100%)	
**23,615,709**	Intron 2	**C**	**C** (100%)	**C** (100%)	**T** (13%)	**C** (100%)	

**23,615,703**	Intron 2	**C**	**C** (100%)	**C** (100%)	**T** (13%)	**C** (100%)
**23,615,697**	Intron 2	**C**	**C** (100%)	**C** (100%)	**T** (13%)	**C** (100%)
**23,615,676**	Intron 2	**C**	**T** (2%)*	**T** (13%)	**T** (17%)	**C** (100%)
**23,615,674**	Intron 2	**C**	**C** (100%)	**T** (7%)*	**T** (18%)	**C** (100%)
**23,615,673**	Intron 2	**C**	**C** (100%)	**T** (13%)	**C** (100%)	**C** (100%)
**23,615,672**	Intron 2	**C**	**C** (100%)	**T** (13%)	**T** (18%)	**C** (100%)
**23,615,650**	Intron 2	**C**	**C** (100%)	**T** (18%)	**C** (100%)	**C** (100%)
**23,615,618**	Intron 2	**C**	**C** (100%)	**T** (10%)*	**T** (15%)	**C** (100%)
**23,615,617**	Intron 2	**C**	**C** (100%)	**T** (20%)	**T** (13%)	**C** (95%)
**23,615,615**	Intron 2	**C**	**C** (100%)	**T** (30%)	**T** (15%)	**C** (100%)
**23,615,614**	Intron 2	**C**	**C** (100%)	**T** (18%)	**T** (5%)*	**C** (100%)
**23,615,606**	Intron 2	**C**	**C** (100%)	**T** (9%)*	**T** (13%)	**C** (100%)
**23,615,521**	Intron 2	**C**	**C** (100%)	**T** (13%)	**T** (7%)*	**C** (100%)
**23,615,519**	Intron 2	**C**	**C** (100%)	**T** (6%)*	**T** (14%)	**C** (100%)
**23,615,517**	Intron 2	**C**	**C** (100%)	**C** (100%)	**T** (13%)	**C** (100%)
**23,615,510**	Intron 2	**C**	**T** (3%)*	**T** (15%)	**T** (5%)*	**C** (100%)
**23,615,473**	Intron 2	**C**	**C** (100%)	**C** (100%)	**T** (13%)	**C** (100%)
**23,615,467**	Intron 2	**C**	**C** (100%)	**T** (12%)	**T** (5%)*	**C** (100%)
**23,615,456**	Intron 2	**C**	**C** (100%)	**T** (19%)	**C** (100%)	**C** (100%)
**23,615,455**	Intron 2	**C**	**C** (100%)	**T** (27%)	**C** (100%)	**C** (100%)
**23,615,427**	Intron 2	**C**	**C** (100%)	**T** (4%)*	**T** (13%)	**C** (100%)
**23,615,423**	Intron 2	**C**	**C** (100%)	**C** (100%)	**T** (14%)	**T** (4%)*
**23,615,415**	Intron 2	**C**	**T** (3%)*	**C** (100%)	**T** (13%)	**C** (100%)
**23,615,352**	Intron 2	**G**	**G** (100%)	**G** (100%)	**A** (16%)	**G** (100%)
**23,615,274**	Intron 2	**C**	**C** (100%)	**C** (100%)	**T** (12%)	**C** (100%)
**23,615,241**	Intron 2	**C**	**C** (98%)	**T** (11%)	**C** (100%)	**C** (100%)
**23,615,234**	Intron 2	**G**	**G** (100%)	**A** (21%)	**G** (100%)	**G** (100%)
**23,615,227**	Intron 2	**G**	**G** (100%)	**G** (100%)	**A** (11%)	**G** (100%)
**23,615,226**	Intron 2	**G**	**G** (100%)	**G** (100%)	**A** (11%)	**G** (100%)
**23,615,221**	Intron 2	**G**	**G** (100%)	**A** (13%)	**G** (100%)	**G** (100%)
**23,615,201**	Intron 2	**C**	**C** (100%)	**C** (100%)	**T** (12%)	**C** (100%)
**23,615,200**	Intron 2	**C**	**C** (100%)	**T** (7%)*	**T** (13%)	**C** (100%)
**23,615,145**	Intron 2	**G**	**G** (100%)	**A** (17%)	**G** (100%)	**G** (100%)
**23,615,129**	Intron 2	**G**	**G** (96%)	**A** (11%)	**G** (100%)	**G** (100%)
**23,615,089**	Intron 2	**G**	**G** (100%)	**G** (100%)	**A** (12%)	**G** (100%)

**23,615,087**	Intron 2	**G**	**G** (100%)	**G** (100%)	**A** (13%)	**G** (100%)	
**23,615,085**	Intron 2	**G**	**G** (100%)	**A** (11%)	**A** (4%)*	**G** (100%)	
**23,615,083**	Intron 2	**G**	**G** (100%)	**A** (15%)	**A** (4%)*	**G** (100%)	
**23,615,082**	Intron 2	**G**	**G** (98%)	**A** (15%)	**G** (100%)	**G** (100%)	
**23,615,064**	Intron 2	**G**	**G** (100%)	**A** (11%)	**G** (100%)	**A** (4%)*	
**23,615,027**	Intron 2	**C**	**C** (100%)	**C** (100%)	**T** (12%)	**C** (100%)	
**23,615,019**	Intron 2	**G**	**G** (100%)	**A** (11%)	**G** (100%)	**G** (100%)	
**23,615,013**	Intron 2	**G**	**G** (100%)	**A** (13%)	**A** (7%)*	**G** (100%)	
**23,614,997**	Intron 2	**G**	**G** (100%)	**G** (100%)	**A** (13%)	**G** (100%)	
**23,614,987**	Intron 2	**C**	**C** (100%)	**T** (11%)	**C** (100%)	**C** (100%)	
**23,614,977**	Intron 2	**C**	**C** (100%)	**T** (13%)	**T** (7%)*	**C** (100%)	
**23,614,952**	Intron 2	**G**	**G** (100%)	**G** (100%)**	**A** (30%)	**G** (100%)	
**23,614,870**	Intron 2	**C**	**C** (98%)	**C** (100%)	**T** (11%)	**C** (100%)	
**23,614,825**	Intron 2	**C**	**C** (100%)	**T** (11%)	**C** (100%)	**C** (100%)	
**23,614,803**	Intron 2	**G**	**G** (100%)	**G** (100%)	**A** (11%)	**G** (100%)	
**23,614,786**	Intron 2	**G**	**G** (100%)	**A** (18%)	**G** (100%)	**G** (100%)	
**23,614,771**	Intron 2	**C**	**C** (100%)	**T** (9%)*	**T** (14%)	**C** (100%)**	
**23,614,767**	Intron 2	**C**	**C** (97%)	**C** (100%)	**T** (11%)	**C** (100%)**	
**23,614,764**	Intron 2	**C**	**C** (100%)	**C** (100%)	**T** (11%)	**C** (100%)**	
**23,614,725**	Intron 2	**G**	**G** (100%)	**A** (9%)*	**A** (15%)	**G** (100%)	
**23,614,722**	Intron 2	**G**	**G** (100%)	**A** (4%)*	**A** (11%)	**G** (100%)	
**23,614,718**	Intron 2	**G**	**G** (97%)	**A** (12%)	**G** (100%)	**G** (100%)	
**23,614,712**	Intron 2	**G**	**G** (100%)	**G** (100%)	**A** (11%)	**G** (100%)	
**23,614,652**	Intron 2	**G**	**G** (100%)	**G** (100%)	**A** (11%)	**G** (100%)	
**23,614,610**	Exon 3	AT**G**	AT**A** (3%)*	AT**G** (100%)	AT**A** (13%)	AT**A** (3%)*	UAC→Y_{102} UAU→Y_{102}
**23,614,550**	3’UTR	**C**	**C** (100%)	**T** (11%)	**T** (10%)*	**C** (100%)	
**23,614,531**	3’UTR	**C**	**C** (100%)	**T** (11%)	**T** (6%)*	**C** (100%)	
**23,614,520**	3’UTR	**G**	**G** (100%)	**A** (13%)	**G** (100%)	**G** (100%)	
**23,614,478**	3’UTR	**G**	**G** (100%)	**A** (11%)	**G** (100%)	**G** (100%)	
**23,614,437**	3’UTR	**G**	**G** (100%)	**G** (100%)	**A** (14%)	**G** (100%)	
**23,614,411**	3’UTR	**G**	**G** (100%)	**A** (11%)	**G** (100%)	**G** (100%)	
**23,614,366**	3’UTR	**G**	**G** (100%)	**A** (11%)	**G** (100%)	**G** (100%)	
**23,614,346**	3’UTR	**G**	**C** (98%)	**C** (100%)	**C** (100%)**	**G** (100%)	
**23,614,301**	3’UTR	**C**	**C** (100%)	**T** (11%)	**C** (100%)	**C** (100%)	

**23,614,292**	Intron 3	**G**	**G** (100%)	**G** (100%)	**A** (15%)	**G** (100%)
**23,614,278**	Intron 3	**C**	**C** (100%)	**T** (5%)*	**T** (11%)	**C** (100%)
**23,614,217**	Intron 3	**G**	**G** (100%)	**A** (15%)	**A** (3%)*	**G** (100%)
**23,614,214**	Intron 3	**C**	**C** (97%)	**C** (100%)	**T** (3%)*	**G** (100%)
**23,614,176**	Intron 3	**G**	**G** (100%)	**A** (5%)*	**G** (100%)	**A** (100%)
**23,614,150**	Intron 3	**G**	**G** (100%)	**G** (100%)	**A** (17%)	**G** (100%)
**23,614,145**	Intron 3	**C**	**C** (100%)	**A** (13%)	**C** (100%)	**C** (100%)
**23,614,138**	Intron 3	**C**	**C** (100%)	**C** (100%)	**T** (15%)	**C** (100%)
**23,614,104**	Intron 3	**C**	**C** (100%)	**T** (12%)	**C** (100%)	**C** (100%)
**23,614,087**	Intron 3	**T**	**C** (100%)	**C** (100%)	**C** (90%)	**C** (90%)
**23,614,052**	Intron 3	**C**	**T** (3%)*	**C** (100%)	**T** (13%)	**C** (100%)
**23,613,951**	Intron 3	**C**	**C** (100%)	**T** (4%)*	**T** (25%)	**C** (100%)
**23,613,950**	Intron 3	**C**	**C** (100%)	**C** (100%)	**T** (25%)	**C** (100%)
**23,613,948**	Intron 3	**C**	**C** (100%)	**T** (14%)	**T** (17%)	**C** (100%)
**23,613,947**	Intron 3	**G**	**G** (98%)	**G** (100%)	**A** (15%)	**G** (100%)
**23,613,924**	Intron 3	**C**	**C** (100%)	**T** (4%)*	**T** (11%)	**C** (100%)
**23,613,799**	Intron 3	**G**	**G** (100%)	**G** (100%)	**A** (11%)	**G** (100%)
**23,613,798**	Intron 3	**C**	**C** (98%)	**C** (100%)	**T** (11%)	**C** (100%)
**23,613,744**	Intron 3	**G**	**G** (100%)	**G** (100%)	**A** (13%)	**G** (100%)
**23,613,706**	Intron 3	**G**	**G** (100%)	**A** (14%)	**G** (100%)	**G** (100%)
**23,613,694**	Intron 3	**G**	**G** (100%)	**A** (11%)	**G** (100%)	**G** (100%)
**23,613,690**	Intron 3	**G**	**G** (100%)	**G** (100%)	**A** (12%)	**A** (100%)
**23,613,650**	Intron 3	**G**	**G** (100%)	**A** (13%)	**A** (3%)*	**G** (100%)
**23,613,611**	Intron 3	**C**	**C** (100%)	**C** (100%)	**T** (12%)	**C** (100%)
**23,613,595**	Intron 3	**C**	**C** (100%)	**T** (14%)	**C** (100%)	**C** (100%)
**23,613,470**	Intron 3	**G**	**G** (100%)	**G** (100%)	**A** (12%)	**G** (100%)
**23,613,467**	Intron 3	**G**	**G** (97%)	**G** (100%)	**A** (12%)	**G** (100%)
**23,613,418**	Intron 3	**G**	**A** (2%)*	**A** (5%)*	**A** (11%)	**G** (100%)
**23,613,416**	Intron 3	**G**	**G** (100%)	**G** (100%)	**A** (19%)	**G** (100%)
**23,613,397**	Intron 3	**C**	**C** (100%)	**C** (100%)	**T** (11%)	**C** (100%)
**23,613,395**	Intron 3	**C**	**T** (2%)*	**T** (13%)	**T** (5%)*	**C** (100%)
**23,613,362**	Intron 3	**C**	**C** (100%)	**T** (12%)	**C** (100%)	**C** (100%)
**23,613,361**	Intron 3	**G**	**G** (100%)	**A** (13%)	**G** (100%)	**G** (100%)
**23,613,358**	Intron 3	**G**	**G** (100%)	**G** (100%)	**A** (11%)	**G** (100%)
**23,613,357**	Intron 3	**G**	**G** (100%)	**A** (6%)*	**A** (16%)	**G** (100%)

**23,613,325**	Intron 3	**G**	**G** (100%)	**A** (5%)*	**A** (12%)	**G** (100%)
**23,613,324**	Intron 3	**G**	**G** (100%)	**A** (4%)*	**A** (16%)	**G** (100%)
**23,613,291**	Intron 3	**C**	**C** (100%)	**T** (15%)	**C** (100%)	**C** (100%)
**23,613,267**	Intron 3	**G**	**G** (100%)	**A** (4%)*	**A** (12%)	**G** (100%)
**23,613,240**	Intron 3	**G**	**G** (100%)	**G** (100%)	**A** (11%)	**G** (100%)
**23,613,232**	Intron 3	**G**	**G** (97%)	**A** (11%)	**A** (4%)*	**G** (100%)
**23,613,214**	Intron 3	**C**	**C** (100%)	**T** (11%)	**C** (100%)	**C** (100%)
**23,613,212**	Intron 3	**C**	**C** (97%)	**C** (100%)	**T** (13%)	**C** (100%)
**23,613,207**	Intron 3	**G**	**A** (3%)*	**A** (11%)	**A** (6%)*	**G** (100%)
**23,613,202**	Intron 3	**C**	**T** (3%)*	**C** (100%)	**T** (11%)	**C** (100%)
**23,613,200**	Intron 3	**C**	**C** (100%)	**T** (13%)	**C** (100%)	**C** (100%)
**23,613,191**	Intron 3	**C**	**C** (100%)	**C** (100%)	**T** (11%)	**C** (100%)
**23,613,187**	Intron 3	**C**	**C** (94%)	**C** (100%)	**C** (100%)	**T** (12%)
**23,613,182**	Intron 3	**G**	**G** (100%)	**A** (11%)	**A** (4%)*	**G** (100%)
**23,613,179**	Intron 3	**G**	**G** (100%)	**G** (100%)	**A** (13%)	**G** (100%)
**23,613,146**	Intron 3	**C**	**C** (100%)	**C** (100%)	**T** (13%)	**C** (100%)
**23,613,130**	Intron 3	**C**	**C** (100%)	**T** (13%)	**C** (100%)	**C** (100%)
**23,613,127**	Intron 3	**C**	**C** (100%)	**T** (13%)	**C** (100%)	**C** (100%)
**23,613,079**	Intron 3	**G**	**G** (100%)	**A** (12%)	**G** (100%)	**G** (100%)
**23,613,073**	Intron 3	**G**	**G** (100%)	**G** (100%)	**A** (11%)	**G** (100%)
**23,613,063**	Intron 3	**C**	**C** (100%)	**C** (100%)	**T** (13%)	**C** (100%)
**23,613,061**	Intron 3	**C**	**C** (100%)	**C** (100%)	**T** (19%)	**C** (100%)
**23,613,048**	Intron 3	**G**	**G** (100%)	**A** (8%)*	**A** (14%)	**G** (100%)
**23,613,046**	Intron 3	**G**	**G** (100%)	**G** (100%)	**A** (15%)	**G** (100%)
**23,613,028**	Intron 3	**C**	**C** (100%)	**T** (5%)*	**T** (17%)	**C** (93%)
**23,613,005**	Intron 3	**C**	**C** (100%)	**T** (4%)*	**T** (13%)	**C** (100%)
**23,613,002**	Intron 3	**C**	**C** (100%)	**T** (4%)*	**T** (21%)	**C** (100%)
**23,612,996**	Intron 3	**C**	**C** (100%)	**T** (9%)*	**T** (15%)	**C** (100%)
**23,612,995**	Intron 3	**C**	**T** (3%)*	**T** (4%)*	**T** (15%)	**C** (100%)
**23,612,989**	Intron 3	**C**	**C** (100%)	**T** (11%)	**T** (18%)	**T** (6%)*
**23,612,988**	Intron 3	**C**	**C** (100%)	**T** (11%)	**T** (8%)*	**C** (94%)
**23,612,982**	Intron 3	**C**	**C** (96%)	**T** (13%)	**T** (7%)*	**C** (100%)
**23,612,960**	Intron 3	**G**	**G** (100%)	**G** (100%)	**A** (11%)	**G** (100%)
**23,612,834**	Intron 3	**C**	**C** (100%)	**T** (10%)*	**T** (12%)	**C** (100%)
**23,612,833**	Intron 3	**G**	**G** (100%)	**G** (100%)	**A** (16%)	**G** (100%)

**23,612,820**	Intron 3	**C**	**C** (100%)	**T** (15%)	**C** (100%)	**C** (100%)
**23,612,818**	Intron 3	**C**	**C** (100%)	**T** (11%)	**C** (100%)	**C** (100%)
**23,612,796**	Intron 3	**G**	**G** (100%)	**A** (11%)	**A** (4%)*	**G** (100%)
**23,612,791**	Intron 3	**A**	**A** (100%)	**A** (100%)	**A** (100%)	**G** (100%)
**23,612,737**	Intron 3	**C**	**T** (100%)	**T** (100%)	**T** (100%)	**C** (100%)
**23,612,726**	Intron 3	**G**	**G** (97%)	**A** (11%)	**A** (3%)*	**G** (100%)
**23,612,710**	Intron 3	**C**	**C** (100%)	**T** (13%)	**T** (3%)*	**C** (100%)**
**23,612,676**	Intron 3	**G**	**G** (100%)	**A** (13%)	**G** (100%)	**G** (100%)
**23,612,675**	Intron 3	**G**	**G** (100%)	**A** (6%)*	**A** (14%)	**G** (100%)
**23,612,661**	Intron 3	**C**	**T** (3%)*	**C** (100%)	**T** (13%)	**C** (100%)
**23,612,642**	Intron 3	**G**	**G** (97%)	**A** (11%)	**G** (100%)	**G** (100%)
**23,612,641**	Intron 3	**G**	**G** (100%)	**A** (11%)	**G** (100%)	**G** (100%)
**23,612,633**	Intron 3	**G**	**G** (97%)	**A** (14%)	**A** (4%)*	**G** (100%)
**23,612,631**	Intron 3	**G**	**G** (97%)	**A** (13%)	**G** (100%)	**A** (4%)*
**23,612,600**	Intron 3	**G**	**A** (10%)*	**G** (100%)	**A** (10%)*	**G** (100%)
**23,612,540**	Intron 3	**C**	**C** (100%)	**T** (20%)	**T** (8%)*	**C** (100%)
**23,612,460**	Intron 3	**C**	**C** (100%)	**T** (4%)*	**T** (13%)	**C** (100%)
**23,612,404**	Intron 3	**G**	**G** (100%)	**A** (14%)	**A** (4%)*	**G** (100%)
**23,612,373**	Intron 3	**C**	**C** (100%)	**T** (7%)*	**T** (12%)	**C** (100%)
**23,612,372**	Intron 3	**C**	**C** (100%)	**T** (7%)*	**T** (11%)	**C** (100%)
**23,612,305**	Intron 3	**A**	**A** (100%)	**A** (100%)	**A** (100%)	**G** (100%)
**23,612,296**	Intron 3	**C**	**C** (100%)	**T** (11%)	**C** (100%)	**C** (100%)
**23,612,272**	Intron 3	**C**	**C** (100%)	**T** (7%)*	**T** (19%)	**C** (100%)
**23,612,258**	Intron 3	**G**	**G** (100%)	**A** (12%)	**G** (94%)	**G** (100%)
**23,612,239**	Intron 3	**C**	**C** (97%)	**C** (100%)	**T** (14%)	**C** (100%)
**23,612,229**	Intron 3	**G**	**G** (100%)	**G** (100%)	**A** (14%)	**G** (100%)
**23,612,210**	Intron 3	**C**	**C** (100%)	**T** (4%)*	**T** (14%)	**C** (100%)
**23,612,202**	Intron 3	**C**	**C** (100%)	**T** (11%)	**C** (100%)	**C** (100%)
**23,612,155**	Intron 3	**C**	**C** (100%)	**T** (13%)	**T** (7%)*	**C** (100%)
**23,612,144**	Intron 3	**G**	**G** (100%)	**A** (13%)	**G** (100%)	**G** (100%)
**23,612,137**	Intron 3	**G**	**G** (100%)	**G** (100%)	**A** (3%)*	**A** (100%)
**23,612,127**	Intron 3	**C**	**T** (2%)*	**C** (100%)	**T** (13%)	**T** (9%)*
**23,612,115**	Intron 3	**G**	**A** (2%)*	**G** (100%)	**A** (28%)	**G** (100%)
**23,612,110**	Intron 3	**C**	**C** (100%)	**T** (4%)*	**T** (13%)	**C** (100%)
**23,612,102**	Intron 3	**G**	**G** (100%)	**A** (11%)	**A** (12%)	**G** (100%)

**23,612,101**	Intron 3	**G**	**G** (100%)	**G** (100%)	**A** (11%)	**G** (100%)
**23,612,100**	Intron 3	**G**	**G** (100%)	**A** (8%)*	**A** (17%)	**G** (100%)
**23,612,094**	Intron 3	**G**	**G** (100%)	**A** (11%)	**A** (6%)*	**G** (100%)
**23,612,093**	Intron 3	**G**	**G** (100%)	**A** (4%)*	**A** (17%)	**G** (100%)
**23,612,092**	Intron 3	**G**	**G** (100%)	**G** (100%)	**A** (16%)	**G** (100%)
**23,612,091**	Intron 3	**G**	**G** (100%)	**G** (100%)	**A** (16%)	**G** (100%)
**23,612,032**	Intron 3	**G**	**A** (4%)*	**A** (13%)	**G** (97%)	**G** (100%)
**23,611,954**	Intron 3	**C**	**T** (3%)*	**C** (100%)	**T** (11%)	**C** (100%)
**23,611,931**	Intron 3	**C**	**T** (2%)*	**T** (5%)*	**T** (17%)	**C** (100%)
**23,611,930**	Intron 3	**C**	**C** (100%)	**T** (14%)	**T** (9%)*	**C** (100%)
**23,611,926**	Intron 3	**G**	**G** (100%)	**G** (100%)	**A** (20%)	**G** (100%)
**23,611,802**	Intron 3	**T**	**T** (100%)	**C** (33%)	**T** (100%)	**T** (100%)
**23,611,789**	Intron 3	**A**	**A** (100%)	**A** (96%)	**A** (100%)	**G** (100%)
**23,611,730**	Intron 3	**C**	**T** (2%)*	**T** (6%)*	**T** (11%)	**C** (100%)
**23,611,708**	Intron 3	**G**	**G** (99%)	**A** (13%)	**A** (4%)*	**G** (100%)
**23,611,687**	Intron 3	**C**	**C** (100%)	**T** (13%)	**T** (3%)*	**C** (100%)
**23,611,573**	Intron 3	**C**	**T** (2%)*	**T** (6%)*	**T** (13%)	**C** (100%)
**23,611,485**	Intron 3	**C**	**C** (100%)	**C** (100%)	**T** (13%)	**C** (100%)
**23,611,413**	Intron 3	**A**	**T** (2%)*	**T** (12%)	**A** (100%)	**A** (100%)
**23,611,412**	Intron 3	**A**	**T** (2%)*	**T** (17%)	**T** (3%)*	**T** (3%)*
**23,611,411**	Intron 3	**A**	**delA** (27%)	**delA** (25%)	**delA** (14%)	**delA** (3%)*
**23,611,410**	Intron 3	**A**	**delA** (68%)	**delA** (44%)<br>**T** (25%)	**delA** (50%)	**delA** (26%)
**23,611,378**	Intron 3	**G**	**G** (100%)	**A** (17%)	**G** (100%)	**G** (100%)
**23,611,354**	Intron 3	**G**	**G** (97%)	**A** (11%)	**G** (100%)	**G** (100%)
**23,611,345**	Intron 3	**A**	**G** (10%)*	**A** (100%)	**A** (100%)	**A** (100%)
**23,611,340**	Intron 3	**C**	**C** (100%)	**T** (5%)*	**T** (11%)	**C** (100%)
**23,611,338**	Intron 3	**C**	**C** (100%)	**T** (11%)	**C** (100%)	**C** (100%)
**23,611,329**	Intron 3	**C**	**C** (100%)	**C** (100%)	**T** (11%)	**C** (100%)
**23,611,302**	Intron 3	**C**	**C** (100%)	**T** (11%)	**C** (100%)	**C** (100%)
**23,611,218**	Intron 3	**G**	**A** (2%)*	**G** (100%)	**A** (12%)	**G** (100%)
**23,611,188**	Intron 3	**G**	**G** (98%)	**A** (15%)	**A** (7%)*	**G** (100%)
**23,611,181**	Intron 3	**G**	**G** (98%)	**A** (24%)	**A** (8%)*	**T** (100%)
**23,611,161**	Intron 3	**G**	**G** (100%)	**G** (100%)	**A** (13%)	**G** (100%)
**23,611,048**	Intron 3	**G**	**G** (100%)	**A** (9%)*	**A** (13%)	**G** (100%)

**23,610,943**	Intron 3	**C**	**C** (100%)	**T** (13%)	**C** (100%)	**C** (100%)
**23,610,937**	Intron 3	**G**	**G** (100%)	**A** (19%)	**G** (100%)	**G** (100%)
**23,610,918**	Intron 3	**G**	**G** (100%)	**A** (5%)*	**A** (19%)	**G** (100%)
**23,610,882**	Intron 3	**G**	**G** (100%)	**G** (100%)	**A** (14%)	**G** (100%)
**23,610,872**	Intron 3	**C**	**C** (100%)	**C** (100%)	**T** (21%)	**C** (100%)
**23,610,870**	Intron 3	**C**	**C** (100%)	**C** (100%)	**T** (15%)	**C** (100%)
**23,610,783**	Intron 3	**G**	**G** (98%)	**A** (4%)*	**A** (11%)	**G** (100%)
**23,610,680**	Intron 3	**C**	**C** (100%)	**C** (100%)	**T** (11%)	**C** (100%)
**23,610,513**	Intron 3	**C**	**C** (100%)	**C** (97%)	**T** (12%)	**C** (100%)
**23,610,497**	Intron 3	**G**	**G** (100%)	**A** (12%)	**A** (11%)	**G** (100%)
**23,610,425**	Intron 3	**G**	**G** (98%)	**G** (100%)	**A** (13%)	**G** (100%)
**23,610,411**	Intron 3	**C**	**C** (100%)	**T** (4%)*	**T** (13%)	**C** (100%)
**23,610,399**	Intron 3	**G**	**G** (100%)	**A** (11%)	**G** (100%)	**G** (100%)
**23,610,393**	Intron 3	**G**	**G** (100%)	**A** (15%)	**A** (6%)*	**G** (100%)
**23,610,389**	Intron 3	**G**	**G** (100%)	**A** (11%)	**G** (100%)	**G** (100%)
**23,610,388**	Intron 3	**G**	**G** (100%)	**A** (13%)	**A** (7%)*	**G** (100%)
**23,610,386**	Intron 3	**G**	**G** (100%)	**A** (7%)*	**A** (13%)	**G** (100%)
**23,610,383**	Intron 3	**G**	**A** (3%)*	**G** (100%)	**A** (13%)	**G** (100%)**
**23,610,377**	Intron 3	**G**	**G** (100%)	**A** (17%)	**A** (13%)	**delG** (90%)**
**23,610,374**	Intron 3	**G**	**G** (100%)	**G** (92%)	**A** (17%)	**G** (100%)**
**23,610,373**	Intron 3	**G**	**G** (100%)	**G** (100%)	**A** (16%)	**G** (100%)**
**23,610,352**	Intron 3	**C**	**C** (100%)	**T** (13%)	**C** (100%)	**C** (100%)
**23,610,348**	Intron 3	**G**	**G** (96%)	**A** (13%)	**A** (6%)*	**G** (100%)
**23,610,347**	Intron 3	**G**	**G** (100%)	**A** (21%)	**A** (6%)*	**G** (100%)
**23,610,262**	Intron 3	**C**	**C** (100%)	**C** (100%)	**C** (100%)	**T** (100%)
**23,610,236**	Intron 3	**C**	**C** (100%)	**T** (8%)*	**T** (11%)	**C** (100%)
**23,610,229**	Intron 3	**G**	**G** (100%)	**A** (4%)*	**A** (12%)	**G** (100%)
**23,610,226**	Intron 3	**G**	**A** (2%)*	**A** (13%)	**A** (13%)	**G** (100%)
**23,610,197**	Intron 3	**G**	**A** (2%)*	**A** (11%)	**A** (9%)*	**G** (100%)
**23,610,192**	Intron 3	**G**	**G** (100%)	**A** (5%)*	**A** (15%)	**G** (100%)
**23,610,189**	Intron 3	**C**	**C** (100%)	**C** (100%)	**T** (18%)	**C** (100%)
**23,610,182**	Intron 3	**C**	**C** (100%)	**C** (100%)	**T** (14%)	**C** (100%)
**23,610,176**	Intron 3	**G**	**C** (100%)	**A** (10%)*	**A** (20%)	**C** (100%)
**23,610,150**	Intron 3	**G**	**G** (100%)	**A** (4%)*	**A** (17%)	**G** (100%)
**23,610,035**	3’UTR	**C**	**C** (100%)	**T** (13%)	**T** (6%)*	**C** (100%)

**23,610,022**	3′UTR	**G**	**G** (100%)	**G** (100%)	**A** (12%)	**A** (5%)*
**23,609,992**	3′UTR	**G**	**G** (100%)	**G** (100%)	**A** (15%)	**G** (100%)
**23,609,989**	3′UTR	**G**	**G** (100%)	**G** (96%)	**A** (14%)	**G** (100%)
**23,609,987**	3′UTR	**G**	**A** (4%)*	**A** (8%)*	**A** (14%)	**G** (100%)
**23,609,861**	3′UTR	**G**	**G** (100%)	**A** (13%)	**A** (7%)*	**G** (100%)
**23,609,860**	3′UTR	**G**	**G** (93%)	**A** (20%)	**A** (14%)	**G** (100%)
**23,609,848**	3′UTR	**G**	**G** (100%)	**A** (17%)	**A** (11%)	**G** (100%)
**23,609,846**	3′UTR	**G**	**G** (100%)	**A** (11%)	**G** (100%)	**G** (100%)
**23,609,839**	3′UTR	**G**	**G** (100%)	**A** (18%)	**G** (100%)	**G** (100%)
**23,609,832**	3′UTR	**G**	**G** (100%)	**A** (11%)	**G** (100%)	**G** (100%)
**23,609,814**	3′UTR	**G**	**G** (100%)	**G** (100%)	**A** (11%)	**G** (100%)
**23,609,813**	3′UTR	**G**	**G** (100%)	**G** (100%)	**A** (15%)	**G** (100%)
**23,609,812**	3′UTR	**G**	**G** (100%)	**G** (100%)	**A** (11%)	**G** (100%)
**23,609,799**	3′UTR	**G**	**G** (100%)	**A** (14%)	**G** (100%)	**G** (100%)
**23,609,753**	3′UTR	**G**	**G** (100%)	**A** (5%)*	**A** (13%)	**G** (100%)
**23,609,750**	3′UTR	**G**	**G** (100%)	**A** (13%)	**G** (100%)	**G** (100%)
**23,609,749**	3′UTR	**G**	**G** (100%)	**A** (13%)	**A** (6%)*	**G** (100%)
**23,609,748**	3′UTR	**G**	**G** (100%)	**A** (4%)*	**A** (13%)	**G** (100%)
**23,609,600**	3′UTR	**C**	**C** (100%)	**C** (100%)	**T** (13%)	**C** (100%)
**23,609,599**	3′UTR	**C**	**C** (100%)	**T** (8%)*	**T** (19%)	**C** (100%)
**23,609,533**	3′UTR	**C**	**C** (100%)	**C** (100%)	**T** (17%)	**C** (100%)
**23,609,509**	3′UTR	**C**	**C** (100%)	**C** (100%)	**T** (12%)	**T** (6%)*
**23,609,480**	3′UTR	**G**	**A** (4%)*	**A** (4%)*	**A** (17%)	**G** (100%)
**23,609,477**	3′UTR	**C**	**T** (4%)*	**T** (12%)	**C** (100%)	**T** (4%)*
**23,609,462**	3′UTR	**C**	**C** (100%)	**T** (14%)	**T** (4%)*	**C** (100%)
**23,609,345**	3′UTR	**C**	**T** (2%)*	**T** (4%)*	**T** (12%)	**C** (100%)
**23,609,237**	3′UTR	**G**	**G** (100%)	**G** (100%)	**A** (13%)	**G** (100%)
**23,609,177**	3′UTR	**G**	**G** (100%)	**A** (4%)*	**A** (13%)	**G** (100%)
**23,609,104**	3′UTR	**G**	**G** (100%)	**A** (10%)*	**A** (13%)	**G** (100%)
**23,609,103**	3′UTR	**G**	**G** (100%)	**G** (100%)	**A** (17%)	**G** (100%)
**23,609,100**	3′UTR	**G**	**G** (100%)	**A** (8%)*	**A** (11%)	**G** (100%)
**23,609,099**	3′UTR	**G**	**G** (100%)	**A** (13%)	**A** (5%)*	**G** (100%)
**23,609,020**	3′UTR	**C**	**C** (100%)	**C** (100%)	**T** (12%)	**C** (100%)
**23,608,995**	3′UTR	**A**	**T** (56%)	**T** (52%)	**A** (100%)	**A** (100%)
**23,608,899**	3′UTR	**G**	**A** (2%)*	**A** (14%)	**G** (100%)	**G** (100%)

**23,608,807**	3′UTR	**G**	**G** (100%)	**A** (11%)	**G** (100%)	**G** (100%)
**23,608,721**	3′UTR	**G**	**G** (100%)	**A** (12%)	**G** (100%)	**G** (100%)
**23,608,570**	3′UTR	**T**	**T** (100%)	**A** (18%)	**T** (100%)	**T** (100%)
**23,608,564**	3′UTR	**T**	**delT** (92%)	**delT** (53%) **G** (14%)	**delT** (92%)	**T** (100%)
**23,608,562**	3′UTR	**G**	**G** (100%)	**T** (14%)	**A** (2%)*	**G** (100%)
**23,608,218**	Downstream region	**G**	**G** (100%)	**A** (14%)	**G** (100%)	**G** (100%)
**23,608,217**	Downstream region	**G**	**G** (100%)	**A** (11%)	**G** (100%)	**G** (100%)
**23,608,213**	Downstream region	**G**	**A** (2%)*	**A** (3%)*	**A** (13%)	**G** (100%)
**23,608,210**	Downstream region	**G**	**G** (100%)	**A** (11%)	**G** (100%)	**G** (100%)
**23,608,053**	Downstream region	**C**	**C** (98%)	**T** (11%)	**T** (7%)*	**C** (100%)
**23,608,036**	Downstream region	**G**	**G** (100%)	**A** (12%)	**A** (13%)	**G** (100%)
**23,608,022**	Downstream region	**G**	**A** (2%)*	**G** (100%)	**A** (4%)*	**A** (11%)
**23,607,996**	Downstream region	**G**	**A** (2%)*	**G** (100%)	**A** (13%)	**G** (100%)
**23,607,829**	Downstream region	**C**	**T** (2%)*	**T** (17%)	**C** (99%)	**C** (97%)
**23,607,793**	Downstream region	**G**	**G** (100%)	**G** (100%)	**A** (15%)	**G** (100%)
**23,607,713**	Downstream region	**C**	**C** (100%)	**C** (97%)	**T** (53%)	**C** (100%)
**23,607,620**	Downstream region	**C**	**C** (100%)	**C** (100%)	**T** (14%)	**C** (100%)
**23,607,585**	Downstream region	**T**	**C** (94%)	**C** (100%)	**C** (97%)	**C** (100%)
**23,607,554**	Downstream region	**G**	**A** (2%)*	**G** (100%)	**A** (15%)	**G** (100%)
**23,607,536**	Downstream region	**G**	**G** (100%)	**A** (10%)*	**A** (16%)	**G** (100%)
**23,607,463**	Downstream region	**G**	**G** (100%)	**G** (100%)	**A** (12%)	**G** (100%)
**23,607,456**	Downstream region	**G**	**G** (100%)	**A** (3%)*	**A** (13%)	**G** (100%)
**23,607,379**	Downstream region	**G**	**A** (100%)	**A** (100%)	**A** (100%)	**A** (100%)
**23,607,309**	Downstream region	**C**	**C** (100%)	**T** (5%)*	**T** (12%)	**C** (100%)
**23,607,244**	Downstream region	**C**	**C** (100%)	**C** (100%)	**G** (41%)	**C** (100%)
**23,607,207**	Downstream region	**C**	**C** (100%)	**C** (100%)	**T** (13%)	**C** (100%)
**23,607,078**	Downstream region	**C**	**C** (100%)	**C** (100%)	**T** (11%)	**C** (100%)
**23,607,034**	Downstream region	**C**	**C** (100%)	**T** (4%)*	**T** (19%)	**C** (100%)
**23,607,027**	Downstream region	**G**	**G** (100%)	**A** (4%)*	**A** (12%)	**G** (97%)
**23,606,942**	Downstream region	**A**	**A** (100%)	**G** (12%)	**A** (100%)	**A** (100%)
**23,606,941**	Downstream region		**InsG** (97%)	**InsG** (82%)	**InsG** (49%)	**InsG** (97%)
**23,606,918**	Downstream region	**G**	**G** (100%)	**A** (14%)	**A** (8%)*	**G** (100%)

**23,606,896**	Downstream region	**C**	**C** (100%)	**T** (20%)	**T** (7%)*	**C** (100%)
**23,606,816**	Downstream region	**G**	**A** (48%)	**A** (38%)	**G** (100%)	**G** (100%)

n.a.: not available; the variant frequency is referred to the percentage of the highlighted base in the sequenced ancient hominine genome, with* frequency≤10% and ** counts<10. In light orange are underlined the variants fixed at 100% in modern human compared to ancient hominines.
