# Supplementary material for: A Catalog of Coding Sequence Variations in Salivary Proteins’ Genes Occurring during Recent Human Evolution
Source: Int J Mol Sci. 2023 Oct 9;24(19):15010. doi: 10.3390/ijms241915010 (PMC10573131; doi:10.3390/ijms241915010)
Supplement: Supplementary file 1 [file ijms-24-15010-s001.zip › Table S14 .pdf]

Table S14. Nucleotide Substitutions in *CST4* gene in introns and regulatory regions

Genomic Coordinates (hg19)	Gene Region	Modern human	Altai Neanderthal (Variant Frequency)	Chagyrskaya Neanderthal (Variant Frequency)	Vindija Neanderthal (Variant Frequency)	Denisovan (Variant Frequency)	Codon → amino acid
**23,670,147**	Upstream regions	**C**	**C** (100%)	**T** (3%)*	**T** (13%)	**C** (100%)	
**23,670,111**	Upstream regions	**G**	**A** (3%)*	**A** (3%)*	**A** (11%)	**G** (100%)	
**23,670,022**	Upstream regions	**G**	**G** (100%)	**A** (10%)*	**A** (12%)	**G** (100%)	
**23,669,985**	Upstream regions	**C**	**C** (100%)	**T** (23%)	**T** (7%)*	**C** (100%)	
**23,669,978**	Upstream regions	**C**	**C** (100%)	**C** (100%)	**T** (11%)	**C** (100%)	
**23,669,971**	Upstream regions	**C**	**C** (100%)	**T** (21%)	**T** (4%)*	**C** (100%)	
**23,669,967**	Upstream regions	**G**	**G** (100%)	**A** (7%)*	**A** (13%)	**G** (100%)	
**23,669,962**	Upstream regions	**C**	**C** (100%)	**T** (11%)	**T** (12%)	**C** (100%)	
**23,669,960**	Upstream regions	**C**	**C** (100%)	**T** (11%)	**C** (100%)	**T** (2%)*	
**23,669,905**	Upstream regions	**C**	**C** (100%)	**T** (10%)*	**T** (11%)	**C** (100%)	
**23,669,881**	Upstream regions	**C**	**C** (100%)	**T** (6%)*	**T** (11%)	**C** (100%)	
**23,669,874**	Upstream regions	**C**	**C** (100%)	**T** (7%)*	**T** (14%)	**C** (100%)	
**23,669,872**	Upstream regions	**C**	**C** (100%)	**T** (8%)*	**T** (13%)	**C** (100%)	
**23,669,871**	Upstream regions	**C**	**C** (100%)	**T** (8%)*	**T** (11%)	**C** (100%)	
**23,669,870**	Upstream regions	**C**	**C** (100%)	**T** (8%)*	**T** (13%)	**C** (100%)	
**23,669,859**	Upstream regions	**C**	**C** (100%)	**C** (100%)	**T** (14%)	**C** (100%)	
**23,669,856**	Upstream regions	**C**	**C** (100%)	**T** (14%)	**C** (100%)	**C** (100%)	
**23,669,855**	Upstream regions	**C**	**C** (100%)	**T** (14%)	**T** (7%)*	**C** (100%)	
**23,669,851**	Upstream regions	**C**	**C** (100%)	**C** (100%)	**T** (13%)	**C** (100%)	
**23,669,850**	Upstream regions	**C**	**C** (100%)	**T** (7%)*	**T** (12%)	**C** (100%)	
**23,669,847**	Upstream regions	**C**	**C** (100%)	**T** (7%)*	**T** (12%)	**C** (100%)	
**23,669,839**	Upstream regions	**C**	**C** (100%)	**T** (13%)	**T** (5%)*	**C** (100%)	
**23,669,838**	Upstream regions	**C**	**C** (100%)	**T** (18%)	**T** (5%)*	**C** (100%)	
**23,669,829**	Upstream regions	**C**	**T** (3%)*	**C** (100%)	**T** (18%)	**C** (100%)	
**23,669,824**	Upstream regions	**C**	**T** (17%)	**T** (5%)*	**T** (27%)	**T** (12%)	
**23,669,822**	Upstream regions	**C**	**T** (15%)	**T** (15%)	**T** (20%)	**T** (12%)	
**23,669,808**	Upstream regions	**C**	**C** (100%)	**C** (100%)	**T** (18%)	**C** (100%)	
**23,669,775**	Upstream regions	**C**	**C** (100%)	**C** (100%)	**T** (11%)	**C** (100%)	

**23,669,762**	Upstream regions	**C**	**C** (100%)	**T** (3%)*	**T** (11%)	**C** (100%)	
**23,669,760**	Upstream regions	**C**	**C** (100%)	**T** (3%)*	**T** (14%)	**C** (100%)	
**23,669,756**	Upstream regions	**C**	**T** (5%)*	**T** (19%)	**T** (9%)*	**T** (29%)	
**23,669,745**	Upstream regions	**C**	**C** (100%)	**C** (100%)	**T** (18%)	**C** (100%)	
**23,669,741**	Upstream regions	**C**	**C** (100%)	**T** (8%)*	**T** (15%)	**C** (100%)	
**23,669,740**	Upstream regions	**C**	**C** (96%)	**T** (26%)	**T** (20%)	**C** (100%)	
**23,669,739**	Upstream regions	**C**	**C** (96%)	**T** (17%)	**T** (10%)*	**C** (100%)	
**23,669,731**	Upstream regions	**C**	**C** (100%)	**T** (14%)	**T** (10%)*	**C** (100%)	
**23,669,723**	Upstream regions	**C**	**C** (96%)	**T** (17%)	**T** (9%)*	**C** (100%)	
**23,669,692**	Upstream regions	**G**	**C** (31%)	**C** (27%)	**C** (44%)**	**C** (24%)	
**23,669,682**	Upstream regions	**C**	**C** (100%)	**C** (100%)	**T** (15%)	**C** (100%)	
**23,669,674**	Upstream regions	**G**	**A** (3%)*	**G** (100%)	**A** (13%)	**G** (100%)	
**23,669,672**	Upstream regions	**G**	**A** (3%)*	**A** (27%)	**G** (100%)	**G** (100%)	
**23,669,663**	Upstream regions	**C**	**T** (41%)	**T** (8%)*	**T** (43%)	**T** (29%)	
**23,669,650**	5′ UTR	**G**	**G** (100%)	**A** (12%)	**G** (100%)	**G** (100%)	
**23,669,630**	5′ UTR	**A**	**G** (74%)	**G** (89%)	**G** (52%)	**G** (79%)	
**23,669,620**	5′ UTR	**G**	**G** (100%)	**A** (13%)	**G** (100%)	**G** (100%)	
**23,669,617**	5′ UTR	**G**	**G** (100%)	**A** (11%)	**A** (5%)*	**G** (100%)	
**23,669,598**	Exon 1 (Signal)	GC**C**	GC**C** (100%)	GC**C** (100%)	GC**T** (5%)*	GC**T** (100%)	CGG→ $R_{2(sp)}$ CGA→ $R_{2(sp)}$
**23,669,577**	Exon 1 (Signal)	GA**G**	GA**G** (100%)	GA**A** (8%)*	GA**A** (11%)	GA**G** (100%)	CUC→$L_{9(sp)}$ CUU→$L_{9(sp)}$
**23,669,544**	Exon 1	AG**C**	AG**T** (5%)*	AG**C** (100%)	AG**T** (13%)	AG**T** (4%)*	UCG→S_1 UCA→S_1
**23,669,538**	Exon 1	AG**G**	AG**G** (100%)	AG**G** (100%)	AG**A** (13%)	AG**G** (100%)	UCC→S_3 UCU→S_3
**23,669,523**	Exon 1	TC**C**	TC**T** (2%)*	TC**C** (100%)	TC**T** (20%)	TC**C** (100%)	AGG→R_8 AGA→R_8
**23,669,493**	Exon 1	CT**G**	CT**G** (100%)	CT**A** (5%)*	CT**A** (20%)	CT**G** (100%)	GAC→D_{18} GAU→D_{18}
**23,669,405**	Exon 1	**G**AC	**G**AC (100%)	**A**AC (12%)	**G**AC (100%)	**G**AC (100%)	CUG→L_{48} UUG→L_{48}
**23,669,378**	Intron 1	**C**	**C** (100%)	**T** (16%)	**T** (6%)*	**C** (100%)	
**23,669,368**	Intron 1	**G**	**G** (100%)	**A** (11%)	**G** (100%)	**G** (100%)	
**23,669,367**	Intron 1	**G**	**G** (100%)	**A** (12%)	**G** (100%)	**G** (100%)	
**23,669,359**	Intron 1	**C**	**C** (100%)	**T** (5%)*	**T** (13%)	**T** (6%)*	
**23,669,356**	Intron 1	**C**	**C** (100%)	**C** (100%)	**T** (13%)	**C** (100%)	
**23,669,354**	Intron 1	**C**	**C** (100%)	**T** (6%)*	**T** (18%)	**C** (100%)	
**23,669,345**	Intron 1	**G**	**G** (100%)	**A** (11%)	**G** (100%)	**G** (100%)	
**23,669,278**	Intron 1	**G**	**G** (100%)	**A** (5%)*	**A** (15%)	**G** (100%)	

**23,669,208**	Intron 1	**A**	**C** (100%)	**C** (100%)	**C** (100%)	**A** (100%)
**23,669,024**	Intron 1	**G**	**G** (100%)	**G** (100%)	**A** (11%)	**G** (100%)
**23,668,993**	Intron 1	**G**	**G** (100%)	**G** (100%)	**A** (14%)	**G** (100%)
**23,668,992**	Intron 1	**G**	**G** (97%)	**G** (100%)	**A** (14%)	**G** (100%)
**23,668,988**	Intron 1	**G**	**G** (100%)	**A** (19%)	**G** (100%)	**G** (100%)
**23,668,971**	Intron 1	**G**	**G** (100%)	**A** (6%)*	**A** (19%)	**G** (100%)
**23,668,966**	Intron 1	**G**	**G** (100%)	**G** (100%)	**A** (11%)	**G** (100%)
**23,668,957**	Intron 1	**G**	**G** (97%)	**A** (17%)	**A** (6%)*	**G** (100%)
**23,668,956**	Intron 1	**G**	**G** (100%)	**A** (6%)*	**A** (11%)	**G** (100%)
**23,668,937**	Intron 1	**G**	**G** (100%)	**G** (100%)	**A** (14%)	**G** (100%)
**23,668,933**	Intron 1	**G**	**G** (100%)	**A** (18%)	**A** (7%)*	**G** (100%)
**23,668,932**	Intron 1	**G**	**G** (100%)	**A** (18%)	**A** (7%)*	**G** (100%)
**23,668,931**	Intron 1	**G**	**G** (100%)	**A** (18%)	**G** (100%)	**G** (100%)
**23,668,911**	Intron 1	**G**	**G** (100%)	**A** (15%)	**A** (17%)**	**G** (100%)
**23,668,891**	Intron 1	**G**	**G** (100%)	**A** (11%)	**G** (100%)**	**G** (100%)
**23,668,890**	Intron 1	**G**	**G** (100%)	**A** (16%)	**G** (100%)**	**G** (100%)
**23,668,848**	Intron 1	**G**	**G** (100%)	**A** (11%)**	**G** (100%)	**A** (13%)
**23,668,829**	Intron 1	**C**	**T** (97%)	**T** (100%)**	**T** (100%)	**C** (100%)
**23,668,801**	Intron 1	**C**	**C** (100%)	**C** (100%)	**T** (13%)	**C** (100%)
**23,668,798**	Intron 1	**C**	**C** (100%)	**C** (100%)	**T** (13%)	**C** (100%)
**23,668,796**	Intron 1	**C**	**C** (100%)	**C** (100%)	**T** (15%)	**C** (100%)
**23,668,765**	Intron 1	**C**	**C** (100%)	**C** (100%)	**T** (14%)	**C** (100%)
**23,668,750**	Intron 1	**C**	**T** (4%)*	**C** (100%)	**T** (13%)	**C** (100%)
**23,668,709**	Intron 1	**G**	**G** (100%)	**G** (100%)	**A** (11%)	**G** (100%)
**23,668,681**	Intron 1	**G**	**G** (100%)	**A** (10%)*	**G** (100%)	**A** (96%)
**23,668,664**	Intron 1	**G**	**G** (100%)	**A** (14%)	**A** (5%)*	**G** (100%)
**23,668,663**	Intron 1	**G**	**G** (98%)	**G** (100%)	**A** (14%)	**G** (100%)
**23,668,639**	Intron 1	**C**	**C** (100%)	**T** (12%)	**T** (9%)*	**C** (100%)
**23,668,636**	Intron 1	**G**	**G** (100%)	**G** (100%)	**A** (14%)	**G** (100%)
**23,668,596**	Intron 1	**G**	**G** (98%)	**G** (100%)	**A** (13%)	**G** (100%)
**23,668,581**	Intron 1	**C**	**C** (100%)	**C** (100%)	**T** (12%)	**C** (100%)
**23,668,573**	Intron 1	**G**	**G** (100%)	**G** (100%)	**A** (11%)	**G** (100%)
**23,668,572**	Intron 1	**G**	**G** (100%)	**A** (14%)	**A** (6%)*	**G** (100%)
**23,668,554**	Intron 1	**G**	**G** (100%)	**A** (12%)	**A** (11%)	**G** (100%)
**23,668,546**	Intron 1	**G**	**G** (100%)	**G** (100%)	**G** (100%)	**T** (100%)

**23,668,494**	Intron 1	**C**	**C** (100%)	**G** (96%)	**G** (100%)	**G** (100%)	
**23,668,492**	Intron 1	**G**	**G** (100%)	**A** (16%)	**A** (8%)*	**G** (100%)	
**23,668,475**	Intron 1	**G**	**G** (100%)	**G** (100%)	**A** (12%)	**G** (100%)	
**23,668,364**	Intron 1	**C**	**C** (100%)	**T** (8%)*	**T** (13%)	**C** (100%)	
**23,668,361**	Intron 1	**C**	**C** (100%)	**T** (12%)	**C** (100%)	**C** (100%)	
**23,668,345**	Intron 1	**C**	**C** (98%)	**C** (100%)	**T** (12%)	**C** (100%)	
**23,668,343**	Intron 1	**C**	**C** (100%)	**C** (100%)	**T** (17%)	**C** (100%)	
**23,668,214**	Intron 1	**C**	**C** (100%)	**T** (3%)*	**T** (11%)	**C** (100%)	
**23,668,111**	Intron 1	**C**	**C** (100%)	**T** (11%)	**T** (4%)*	**C** (100%)	
**23,668,091**	Intron 1	**G**	**G** (100%)	**A** (3%)*	**A** (11%)	**G** (100%)	
**23,668,033**	Intron 1	**G**	**A** (98%)	**A** (100%)	**A** (98%)	**G** (100%)	
**23,668,002**	Intron 1	**G**	**G** (100%)	**A** (6%)*	**A** (17%)	**G** (100%)	
**23,667,973**	Intron 1	**C**	**G** (14%)	**C** (90%)	**C** (95%)	**C** (100%)	
**23,667,957**	Intron 1	**G**	**G** (100%)	**G** (100%)	**A** (21%)	**G** (100%)	
**23,667,917**	Intron 1	**G**	**G** (100%)	**A** (4%)*	**A** (3%)*	**A** (23%)	
**23,667,866**	Intron 1	**G**	**A** (2%)*	**A** (7%)*	**A** (18%)	**G** (100%)	
**23,667,862**	Intron 1	**G**	**G** (100%)	**G** (100%)	**A** (13%)	**G** (100%)	
**23,667,797**	Exon 2	CC**G**	CC**G** (100%)	CC**G** (100%)	CC**A** (13%)	CC**G** (100%)	GGC→G_{70}<br>GGU→G_{70}
**23,667,794**	Exon 2	GC**G**	GC**T** (20%)	GC**T** (12%)	GC**T** (23%)	GC**T** (12%)	CGC→R_{71}<br>CGA→R_{71}
**23,667,782**	Exon 2	TG**G**	TG**A** (3%)	TG**G** (100%)	TG**A** (15%)	TG**G** (100%)	ACC→T_{75}<br>ACU→T_{75}
**23,667,767**	Exon 2	TT**G**	TT**G** (100%)	TT**G** (100%)	TT**A** (12%)	TT**G** (100%)	AAC→N_{80}<br>AAU→N_{80}
**23,667,720**	Intron 2	**C**	**T** (3%)*	**C** (100%)	**T** (15%)	**C** (100%)	
**23,667,675**	Intron 2	**G**	**G** (100%)	**G** (100%)	**A** (13%)	**G** (100%)	
**23,667,674**	Intron 2	**C**	**C** (100%)	**T** (6%)*	**T** (13%)	**C** (100%)	
**23,667,657**	Intron 2	**A**	**G** (100%)	**G** (100%)	**G** (94%)	**G** (100%)	
**23,667,612**	Intron 2	**C**	**C** (100%)	**T** (18%)	**T** (22%)**	**C** (100%)	
**23,667,606**	Intron 2	**C**	**C** (100%)	**T** (15%)	**T** (14%)**	**C** (100%)	
**23,667,572**	Intron 2	**T**	**C** (11%)	**C** (11%)	**C** (25%)**	**C** (9%)*	
**23,667,568**	Intron 2	**C**	**T** (11%)	**T** (18%)	**T** (33%)**	**T** (9%)*	
**23,667,558**	Intron 2	**T**	**G** (10%)*	**G** (16%)	**G** (30%)*	**G** (12%)	
**23,667,521**	Intron 2	**G**	**A** (100%)	**A** (95%)	**A** (100%)	**G** (96%)	
**23,667,514**	Intron 2	**C**	**C** (100%)	**C** (100%)	**T** (11%)	**C** (100%)	
**23,667,496**	Intron 2	**C**	**C** (97%)	**C** (100%)	**T** (12%)	**C** (100%)	
**23,667,472**	Intron 2	**C**	**C** (100%)	**T** (13%)	**T** (7%)*	**C** (100%)	

**23,667,466**	Intron 2	**C**	**C** (100%)	**T** (17%)	**T** (6%)*	**C** (100%)
**23,667,425**	Intron 2	**C**	**C** (100%)	**T** (5%)*	**T** (13%)	**C** (100%)
**23,667,392**	Intron 2	**G**	**G** (100%)	**G** (100%)	**A** (12%)	**G** (100%)
**23,667,376**	Intron 2	**C**	**C** (100%)	**C** (100%)	**T** (12%)	**C** (100%)
**23,667,375**	Intron 2	**C**	**C** (100%)	**C** (100%)	**T** (13%)	**C** (100%)
**23,667,374**	Intron 2	**C**	**C** (100%)	**T** (6%)*	**T** (13%)	**C** (100%)
**23,667,373**	Intron 2	**C**	**C** (100%)	**T** (6%)*	**T** (14%)	**C** (100%)
**23,667,368**	Intron 2	**C**	**C** (100%)	**C** (100%)	**T** (13%)	**C** (100%)
**23,667,361**	Intron 2	**C**	**C** (100%)	**T** (7%)*	**T** (13%)	**C** (100%)
**23,667,360**	Intron 2	**C**	**T** (3%)*	**T** (7%)*	**T** (13%)	**C** (100%)
**23,667,359**	Intron 2	**C**	**C** (100%)	**C** (100%)	**T** (20%)	**C** (100%)
**23,667,358**	Intron 2	**C**	**C** (100%)	**T** (21%)	**T** (25%)	**C** (100%)
**23,667,353**	Intron 2	**C**	**C** (100%)	**C** (100%)	**T** (11%)	**C** (100%)
**23,667,351**	Intron 2	**C**	**C** (100%)	**C** (100%)	**T** (18%)	**C** (100%)
**23,667,322**	Intron 2	**C**	**C** (100%)	**T** (17%)	**T** (3%)*	**C** (100%)
**23,667,312**	Intron 2	**G**	**G** (100%)	**G** (100%)	**A** (12%)	**G** (100%)
**23,667,306**	Intron 2	**C**	**C** (100%)	**T** (21%)	**T** (8%)*	**C** (100%)
**23,667,287**	Intron 2	**C**	**T** (11%)	**T** (22%)	**T** (23%)	**T** (20%)
**23,667,260**	Intron 2	**T**	**T** (100%)	**G** (21%)	**G** (26%)	**T** (100%)
**23,667,255**	Intron 2	**G**	**C** (26%)	**C** (26%)	**C** (42%)	**G** (98%)
**23,667,236**	Intron 2	**C**	**C** (100%)	**T** (13%)	**T** (4%)*	**C** (100%)
**23,667,225**	Intron 2	**T**	**G** (16%)	**G** (26%)	**G** (13%)	**G** (16%)
**23,667,209**	Intron 2	**G**	**C** (9%)*	**C** (23%)	**C** (8%)*	**C** (6%)*
**23,667,134**	Intron 2	**C**	**C** (100%)	**T** (18%)	**C** (100%)	**C** (100%)
**23,667,127**	Intron 2	**G**	**G** (100%)	**G** (100%)	**A** (14%)	**G** (100%)
**23,667,098**	Intron 2	**T**	**C** (100%)	**C** (100%)	**T** (100%)**	**C** (23%)
**23,667,054**	Intron 2	**G**	**A** (3%)*	**A** (11%)	**G** (100%)**	**G** (100%)
**23,667,052**	Intron 2	**G**	**G** (100%)	**A** (11%)	**G** (100%)**	**G** (100%)
**23,667,042**	Intron 2	**G**	**G** (100%)	**A** (21%)	**G** (100%)**	**G** (100%)
**23,667,036**	Intron 2	**T**	**G** (100%)	**G** (75%)<br>**G** (25%)	**G** (75%)**<br>**G** (25%)**	**G** (13%)
**23,667,012**	Intron 2	**C**	**C** (100%)	**C** (100%)	**T** (11%)	**C** (100%)
**23,667,011**	Intron 2	**C**	**C** (97%)	**T** (7%)*	**T** (11%)	**C** (100%)
**23,666,999**	Intron 2	**G**	**G** (100%)	**A** (24%)	**G** (100%)	**G** (100%)
**23,666,986**	Intron 2	**G**	**G** (100%)	**A** (13%)	**G** (100%)	**G** (100%)

**23,666,971**	Intron 2	**C**	**T** (2%)*	**T** (11%)	**T** (5%)*	**C** (100%)
**23,666,960**	Intron 2	**C**	**T** (14%)	**C** (100%)	**T** (18%)	**C** (100%)
**23,666,924**	Intron 2	**T**	**C** (3%)*	**T** (100%)	**T** (100%)	**C** (38%)
**23,666,923**	Intron 2	**G**	**G** (100%)	**A** (13%)	**G** (100%)	**G** (100%)
**23,666,920**	Intron 2	**G**	**G** (100%)	**A** (18%)	**G** (100%)	**G** (100%)
**23,666,905**	Intron 2	**G**	**G** (100%)	**A** (13%)	**G** (100%)	**G** (100%)
**23,666,888**	Intron 2	**G**	**G** (100%)	**A** (6%)*	**A** (20%)	**G** (100%)
**23,666,882**	Intron 2	**G**	**G** (97%)	**A** (13%)	**G** (100%)	**G** (100%)
**23,666,857**	Intron 2	**T**	**G** (100%)	**G** (100%)**	**G** (91%)	**T** (100%)
**23,666,851**	Intron 2	**G**	**G** (100%)	**G** (100%)**	**A** (17%)	**G** (100%)
**23,666,812**	Intron 2	**C**	**C** (100%)	**C** (100%)	**T** (7%)*	**T** (100%)
**23,666,804**	Intron 2	**C**	**C** (98%)	**T** (6%)*	**T** (20%)	**C** (100%)
**23,666,778**	Intron 2	**C**	**C** (100%)	**C** (100%)	**T** (13%)	**C** (100%)
**23,666,762**	Intron 2	**C**	**C** (100%)	**C** (100%)	**T** (12%)	**C** (100%)
**23,666,752**	Intron 2	**G**	**G** (100%)	**A** (13%)	**G** (100%)	**G** (100%)
**23,666,728**	Intron 2	**C**	**C** (100%)	**T** (12%)	**C** (100%)	**C** (100%)
**23,666,726**	Intron 2	**C**	**C** (100%)	**T** (12%)	**C** (100%)	**C** (100%)
**23,666,719**	Intron 2	**C**	**T** (100%)	**T** (100%)	**T** (100%)	**C** (100%)
**23,666,690**	Intron 2	**C**	**G** (94%)	**G** (100%)	**G** (100%)**	**G** (100%)**
**23,666,665**	Intron 2	**G**	**G** (100%)	**A** (15%)	**G** (100%)	**G** (100%)
**23,666,650**	Intron 2	**G**	**G** (100%)	**A** (13%)	**G** (100%)	**A** (6%)*
**23,666,644**	Intron 2	**G**	**G** (100%)	**G** (100%)	**A** (11%)	**G** (100%)
**23,666,642**	Intron 2	**C**	**C** (98%)	**C** (100%)	**T** (13%)	**C** (100%)
**23,666,528**	3′UTR	**C**	**C** (100%)	**T** (11%)	**C** (100%)	**C** (100%)
**23,666,526**	3′UTR	**G**	**G** (100%)	**A** (11%)	**G** (100%)	**G** (100%)
**23,666,524**	3′UTR	**C**	**C** (98%)	**C** (100%)	**T** (17%)	**C** (100%)
**23,666,496**	3′UTR	**G**	**G** (100%)	**A** (14%)	**A** (25%)**	**G** (100%)
**23,666,484**	3′UTR	**T**	**G** (96%)	**G** (100%)	**G** (67%)**	**T** (100%)
**23,666,442**	3′UTR	**C**	**C** (100%)	**T** (17%)	**T** (8%)*	**C** (100%)
**23,666,437**	3′UTR	**C**	**A** (85%)	**A** (100%)	**A** (100%)	**C** (100%)
**23,666,435**	3′UTR	**G**	**G** (100%)	**G** (100%)	**A** (14%)	**G** (100%)
**23,666,433**	3′UTR	**G**	**G** (100%)	**G** (100%)	**A** (14%)	**G** (100%)
**23,666,431**	3′UTR	**G**	**G** (100%)	**G** (100%)	**A** (13%)	**G** (100%)
**23,666,423**	3′UTR	**G**	**A** (3%)*	**G** (100%)	**A** (11%)	**G** (100%)
**23,666,395**	3′UTR	**C**	**C** (100%)	**T** (11%)	**C** (100%)	**C** (100%)

**23,666,368**	3′UTR	**G**	**A** (2%)*	**G** (95%)	**A** (26%)	**G** (100%)
**23,666,364**	3′UTR	**G**	**G** (100%)	**A** (14%)	**A** (15%)	**G** (100%)
**23,666,361**	3′UTR	**G**	**G** (100%)	**A** (5%)*	**A** (11%)	**G** (100%)
**23,666,359**	3′UTR	**G**	**G** (98%)	**G** (100%)	**A** (11%)	**G** (100%)
**23,666,331**	3′UTR	**T**	**G** (22%)	**G** (12%)	**G** (6%)*	**G** (27%)
**23,666,330**	3′UTR	**G**	**G** (100%)	**G** (100%)	**A** (18%)	**G** (100%)
**23,666,329**	3′UTR	**G**	**G** (100%)	**G** (100%)	**A** (17%)	**G** (100%)
**23,666,319**	3′UTR	**C**	**C** (100%)	**C** (100%)	**T** (14%)	**C** (100%)
**23,666,271**	Downstream regions	**G**	**G** (100%)	**A** (5%)*	**A** (13%)	**G** (100%)
**23,666,257**	Downstream regions	**C**	**C** (100%)	**T** (13%)	**T** (7%)*	**C** (100%)
**23,666,241**	Downstream regions	**T**	**C** (9%)*	**T** (100%)	**C** (4%)*	**C** (11%)
**23,666,196**	Downstream regions	**C**	**G** (89%)	**G** (80%)	**G** (86%)**	**G** (100%)
**23,666,181**	Downstream regions	**G**	**C** (97%)	**C** (90%)	**C** (100%)**	**G** (92%)
**23,666,158**	Downstream regions	**C**	**T** (100%)	**T** (100%)	**T** (100%)	**C** (100%)
**23,666,145**	Downstream regions	**G**	**G** (100%)	**A** (12%)	**G** (100%)	**G** (100%)
**23,666,144**	Downstream regions	**G**	**G** (100%)	**A** (12%)	**G** (100%)	**G** (100%)
**23,666,113**	Downstream regions	**G**	**G** (97%)	**A** (12%)	**G** (100%)	**G** (100%)
**23,666,111**	Downstream regions	**G**	**G** (100%)	**G** (100%)	**A** (14%)	**G** (100%)
**23,666,051**	Downstream regions	**C**	**C** (100%)	**T** (13%)	**C** (100%)	**C** (100%)
**23,666,026**	Downstream regions	**C**	**C** (100%)	**T** (13%)	**C** (100%)	**C** (100%)
**23,666,014**	Downstream regions	**C**	**C** (100%)	**T** (13%)	**T** (4%)*	**C** (100%)
**23,666,013**	Downstream regions	**C**	**C** (100%)	**T** (16%)	**T** (4%)*	**C** (100%)
**23,665,990**	Downstream regions	**C**	**C** (100%)	**C** (100%)	**T** (11%)	**C** (100%)
**23,665,955**	Downstream regions	**C**	**T** (100%)	**T** (100%)	**T** (100%)	**C** (100%)
**23,665,951**	Downstream regions	**C**	**C** (100%)	**C** (100%)	**T** (11%)	**T** (6%)*
**23,665,950**	Downstream regions	**C**	**C** (100%)	**T** (12%)	**T** (10%)*	**C** (100%)
**23,665,949**	Downstream regions	**C**	**C** (100%)	**T** (12%)	**T** (10%)*	**C** (100%)
**23,665,942**	Downstream regions	**C**	**C** (100%)	**C** (100%)	**T** (11%)	**C** (100%)
**23,665,940**	Downstream regions	**C**	**C** (100%)	**T** (7%)*	**T** (11%)	**C** (100%)
**23,665,933**	Downstream regions	**C**	**C** (100%)	**C** (100%)	**T** (15%)	**C** (100%)
**23,665,929**	Downstream regions	**C**	**C** (100%)	**T** (7%)*	**T** (11%)	**C** (100%)
**23,665,928**	Downstream regions	**C**	**C** (100%)	**T** (8%)*	**T** (11%)	**C** (100%)

**23,665,916**	Downstream regions	**C**	**C** (100%)	**T** (6%)*	**T** (13%)	**C** (100%)
**23,665,898**	Downstream regions	**C**	**C** (100%)	**T** (11%)	**C** (100%)	**C** (100%)
**23,665,862**	Downstream regions	**G**	**G** (100%)	**G** (100%)	**A** (11%)	**G** (100%)
**23,665,835**	Downstream regions	**G**	**G** (100%)	**G** (100%)	**A** (11%)	**G** (100%)
**23,665,834**	Downstream regions	**G**	**G** (100%)	**A** (14%)	**G** (100%)	**G** (100%)
**23,665,832**	Downstream regions	**G**	**G** (100%)	**A** (5%)*	**A** (11%)	**G** (100%)
**23,665,827**	Downstream regions	**G**	**G** (100%)	**A** (14%)	**G** (100%)	**G** (100%)
**23,665,790**	Downstream regions	**G**	**G** (100%)	**A** (4%)*	**A** (12%)	**G** (100%)
**23,665,772**	Downstream regions	**T**	**A** (4%)*	**A** (13%)	**T** (100%)	**A** (5%)*
**23,665,726**	Downstream regions	**C**	**A** (19%)	**A** (12%)	**A** (4%)*	**C** (100%)
**23,665,720**	Downstream regions	**C**	**T** (5%)*	**T** (6%)*	**T** (12%)	**C** (100%)
**23,665,718**	Downstream regions	**C**	**A** (73%)	**A** (68%)	**A** (59%)	**C** (100%)
**23,665,714**	Downstream regions	**C**	**C** (100%)	**T** (23%)	**T** (11%)	**C** (100%)
**23,665,712**	Downstream regions	**T**	**C** (5%)*	**T** (100%)	**T** (100%)	**C** (32%)
**23,665,711**	Downstream regions	**T**	**A** (5%)*	**T** (100%)	**T** (100%)	**A** (32%)
**23,665,710**	Downstream regions	**G**	**C** (3%)*	**G** (100%)	**G** (100%)	**C** (24%)
**23,665,708**	Downstream regions	**C**	**C** (100%)	**A** (15%)	**A** (4%)*	**C** (100%)
**23,665,707**	Downstream regions	**T**	**T** (100%)	**T** (100%)	**T** (100%)	**A** (14%)
**23,665,706**	Downstream regions	**A**	**A** (100%)	**A** (100%)	**A** (100%)	**C** (14%)

n.a.: not available; the variant frequency is referred to the percentage of the highlighted base in the sequenced ancient hominine genome, with* frequency≤10% and ** counts<10. In light orange are underlined the variants fixed at 100% in modern human compared to ancient hominines.
