# Supplementary material for: A Catalog of Coding Sequence Variations in Salivary Proteins’ Genes Occurring during Recent Human Evolution
Source: Int J Mol Sci. 2023 Oct 9;24(19):15010. doi: 10.3390/ijms241915010 (PMC10573131; doi:10.3390/ijms241915010)
Supplement: Supplementary file 1 [file ijms-24-15010-s001.zip › Table S15.pdf]

Table S15. Nucleotide Substitutions in *CST5* gene in introns and regulatory regions

Genomic Coordinates (hg19)	Gene Region	Modern human	Altai Neanderthal (Variant Frequency)	Chagyrskaya Neanderthal (Variant Frequency)	Vindija Neanderthal (Variant Frequency)	Denisovan (Variant Frequency)	Codon→ amino acid
**23,860,839**	Upstream regions	**C**	**C** (100%)	**C** (100%)	**T** (14%)	**C** (100%)	
**23,860,785**	Upstream regions	**G**	**G** (100%)	**A** (4%)*	**A** (11%)	**G** (100%)	
**23,860,782**	Upstream regions	**G**	**G** (100%)	**A** (4%)*	**A** (18%)	**G** (100%)	
**23,860,761**	Upstream regions	**G**	**A** (2%)*	**A** (4%)*	**A** (19%)	**G** (100%)	
**23,860,736**	Upstream regions	**G**	**G** (100%)	**A** (4%)*	**A** (11%)	**G** (100%)	
**23,860,710**	Upstream regions	**G**	**G** (100%)	**A** (12%)	**A** (7%)*	**G** (100%)	
**23,860,681**	Upstream regions	**C**	**C** (98%)	**C** (100%)	**T** (13%)	**C** (100%)	
**23,860,680**	Upstream regions	**C**	**C** (100%)	**T** (4%)*	**T** (13%)	**C** (100%)	
**23,860,677**	Upstream regions	**C**	**C** (96%)	**T** (4%)*	**T** (18%)	**C** (100%)	
**23,860,626**	Upstream regions	**C**	**C** (100%)	**C** (100%)	**T** (11%)	**C** (100%)	
**23,860,613**	Upstream regions	**C**	**C** (100%)	**T** (12%)	**T** (8%)*	**C** (100%)	
**23,860,587**	Upstream regions	**C**	**T** (3%)*	**C** (100%)	**T** (13%)	**C** (100%)	
**23,860,567**	Upstream regions	**C**	**C** (97%)	**T** (15%)	**T** (5%)*	**C** (100%)	
**23,860,550**	Upstream regions	**C**	**C** (97%)	**T** (9%)*	**T** (15%)	**C** (100%)	
**23,860,548**	Upstream regions	**C**	**C** (100%)	**C** (100%)	**T** (11%)	**C** (100%)	
**23,860,523**	Upstream regions	**C**	**C** (100%)	**T** (3%)*	**T** (19%)	**C** (100%)	
**23,860,513**	Upstream regions	**C**	**C** (100%)	**T** (9%)*	**T** (13%)	**C** (100%)	
**23,860,498**	Upstream regions	**C**	**G** (97%)	**G** (100%)	**G** (92%)	**C** (100%)	
**23,860,482**	Upstream regions	**C**	**C** (100%)	**T** (11%)**	**T** (13%)	**C** (100%)	
**23,860,481**	Upstream regions	**C**	**C** (100%)	**C** (100%)	**T** (14%)	**C** (100%)	
**23,860,468**	Upstream regions	**C**	**C** (100%)	**C** (100%)	**T** (14%)	**C** (100%)	
**23,860,466**	Upstream regions	**C**	**C** (100%)	**C** (100%)	**T** (14%)	**C** (100%)	
**23,860,435**	Upstream regions	**C**	**C** (100%)	**T** (7%)*	**T** (13%)	**C** (100%)	
**23,860,434**	Upstream regions	**C**	**C** (100%)	**T** (8%)*	**T** (13%)	**C** (100%)	
**23,860,289**	Exon 1 (Signal)	**G**AC	**G**AC (100%)	**A**AC (4%)*	**A**AC (17%)	**G**AC (100%)	CUG→$L_{9(sp)}$<br>UUG→$L_{9(sp)}$
**23,860,227**	Exon 1	TG**G**	TG**G** (100%)	TG**A** (17%)	TG**A** (6%)*	TG**G** (100%)	ACC→T_9<br>ACA→T_9
**23,860,212**	Exon 1	TA**G**	TA**G** (100%)	TA**G** (92%)	TA**A** (12%)	TA**G** (100%)	AUC→I_{14}<br>AUU→I_{14}
**23,860,158**	Exon 1	TA**G**	TA**G** (100%)	TA**G** (100%)	TA**A** (13%)	TA**G** (100%)	AUC→I_{32}<br>AUU→I_{32}

**23,860,152**	Exon 1	CT<u>**C**</u>	CT<u>**C**</u> (100%)	CT<u>**T**</u> (7%)*	CT<u>**T**</u> (14%)	CT<u>**C**</u> (100%)	GAG→E_{34}<br>GAA→E_{34}
**23,860,113**	Exon 1	GC<u>**G**</u>	GC<u>**G**</u> (100%)	GC<u>**A**</u> (17%)	GC<u>**A**</u> (9%)*	GC<u>**G**</u> (100%)	CGC→R_{47}<br>CGU→R_{47}
**23,860,083**	Exon 1	GT<u>**C**</u>	GT<u>**C**</u> (100%)	GT<u>**T**</u> (7%)*	GT<u>**T**</u> (12%)	GT<u>**C**</u> (100%)	CAG→Q_{57}<br>CAA→Q_{57}
**23,860,082**	Intron 1	**C**	**C** (100%)	**C** (100%)	**T** (13%)	**C** (100%)	
**23,860,079**	Intron 1	**G**	**G** (100%)	**A** (6%)*	**A** (13%)	**G** (100%)	
**23,860,046**	Intron 1	**T**	**T** (100%)	**T** (100%)	**T** (100%)	**G** (100%)	
**23,860,033**	Intron 1	**C**	**C** (100%)	**T** (8%)*	**T** (18%)	**C** (100%)	
**23,860,027**	Intron 1	**G**	**G** (100%)	**G** (100%)	**A** (14%)	**G** (100%)	
**23,859,994**	Intron 1	**G**	**G** (100%)	**A** (8%)*	**A** (13%)	**G** (100%)	
**23,859,956**	Intron 1	**G**	**G** (100%)	**A** (13%)	**A** (3%)*	**G** (100%)	
**23,859,950**	Intron 1	**C**	**C** (100%)	**T** (14%)	**T** (6%)*	**T** (3%)*	
**23,859,906**	Intron 1	**G**	**A** (94%)	**A** (88%)	**A** (82%)	**A** (94%)	
**23,859,861**	Intron 1	**G**	**G** (100%)	**G** (100%)	**A** (14%)	**G** (100%)	
**23,859,816**	Intron 1	**G**	**G** (100%)	**G** (100%)	**A** (17%)	**G** (100%)	
**23,859,798**	Intron 1	**C**	**C** (100%)	**C** (100%)	**T** (12%)	**C** (100%)	
**23,859,753**	Intron 1	**C**	**T** (100%)	**T** (100%)	**T** (100%)	**T** (100%)	
**23,859,750**	Intron 1	**C**	**C** (100%)	**T** (3%)*	**T** (11%)	**C** (100%)	
**23,859,700**	Intron 1	**G**	**G** (100%)	**A** (4%)*	**A** (18%)	**G** (100%)	
**23,859,653**	Intron 1	**C**	**C** (100%)	**T** (11%)	**C** (100%)	**C** (100%)	
**23,859,644**	Intron 1	**C**	**C** (100%)	**C** (100%)	**T** (11%)	**C** (100%)	
**23,859,604**	Intron 1	**G**	**G** (100%)	**A** (23%)	**G** (100%)	**G** (100%)	
**23,859,603**	Intron 1	**G**	**G** (100%)	**A** (15%)	**G** (100%)	**G** (100%)	
**23,859,586**	Intron 1	**C**	**C** (100%)	**C** (83%)	**T** (14%)	**C** (100%)	
**23,859,570**	Intron 1	**C**	**C** (100%)	**T** (15%)	**T** (10%)*	**C** (100%)	
**23,859,460**	Intron 1	**C**	**C** (100%)	**C** (100%)	**T** (11%)	**C** (100%)	
**23,859,440**	Intron 1	**G**	**G** (100%)	**G** (100%)	**A** (11%)	**G** (100%)	
**23,859,396**	Intron 1	**G**	**G** (100%)	**G** (100%)	**A** (18%)	**G** (100%)	
**23,859,394**	Intron 1	**C**	**C** (100%)	**C** (100%)	**T** (12%)	**C** (100%)	
**23,859,314**	Intron 1	**G**	**G** (100%)	**A** (11%)	**G** (100%)	**G** (100%)	
**23,859,282**	Intron 1	**G**	**G** (100%)	**G** (100%)	**A** (11%)	**G** (100%)	
**23,859,246**	Intron 1	**C**	**C** (100%)	**C** (100%)	**T** (11%)	**C** (100%)	
**23,859,213**	Intron 1	**C**	**C** (100%)	**C** (100%)	**T** (14%)	**C** (100%)	
**23,859,210**	Intron 1	**C**	**C** (100%)	**T** (6%)*	**T** (15%)	**C** (100%)	
**23,859,131**	Intron 1	**C**	**C** (100%)	**T** (5%)*	**T** (13%)	**C** (100%)	

**23,859,086**	Intron 1	**G**	**G** (100%)	**G** (100%)	**A** (11%)	**G** (100%)	
**23,859,050**	Intron 1	**G**	**A** (27%)	**A** (36%)	**A** (47%)	**G** (97%)	
**23,859,019**	Intron 1	**A**	**G** (48%)	**G** (50%)	**G** (33%)	**G** (45%)	
**23,859,017**	Intron 1	**A**	**C** (47%)	**C** (43%)	**C** (65%)	**C** (43%)	
**23,859,015**	Intron 1	**C**	**C** (100%)	**C** (100%)	**T** (11%)	**C** (100%)	
**23,858,984**	Intron 1	**G**	**T** (48%)	**T** (43%)	**T** (41%)	**T** (46%)	
**23,858,950**	Intron 1	**C**	**C** (100%)	**T** (21%)	**T** (7%)*	**C** (100%)	
**23,858,924**	Intron 1	**A**	**A** (100%)	**A** (100%)	**A** (100%)	**C** (25%)	
**23,858,901**	Intron 1	**C**	**C** (100%)	**T** (3%)*	**T** (13%)	**T** (2%)*	
**23,858,897**	Intron 1	**A**	**G** (62%)	**G** (67%)	**A** (100%)	**A** (97%)	
**23,858,775**	Intron 1	**A**	**G** (95%)	**G** (93%)	**G** (87%)	**A** (100%)	
**23,858,746**	Intron 1	**G**	**G** (100%)	**A** (12%)	**A** (4%)*	**G** (100%)	
**23,858,715**	Intron 1	**T**	**T** (100%)	**T** (100%)	**T** (100%)	**A** (100%)	
**23,858,705**	Intron 1	**G**	**G** (100%)	**A** (9%)*	**A** (20%)	**G** (100%)	
**23,858,701**	Intron 1	**G**	**G** (100%)	**A** (12%)	**G** (100%)	**G** (100%)	
**23,858,698**	Intron 1	**G**	**G** (100%)	**A** (11%)	**G** (100%)	**G** (100%)	
**23,858,689**	Intron 1	**C**	**C** (100%)	**C** (100%)	**T** (13%)	**C** (100%)	
**23,858,675**	Intron 1	**C**	**C** (100%)	**C** (100%)	**T** (14%)	**C** (100%)	
**23,858,614**	Intron 1	**C**	**G** (100%)	**G** (95%)	**G** (91%)	**C** (96%)	
**23,858,542**	Intron 1	**C**	**C** (100%)	**C** (100%)	**T** (13%)	**C** (100%)	
**23,858,526**	Intron 1	**G**	**G** (100%)	**G** (100%)	**A** (18%)	**G** (100%)	
**23,858,525**	Intron 1	**G**	**G** (100%)	**G** (100%)	**A** (12%)	**G** (100%)	
**23,858,521**	Intron 1	**G**	**G** (100%)	**A** (6%)*	**A** (11%)	**G** (100%)	
**23,858,517**	Intron 1	**G**	**G** (100%)	**G** (100%)	**A** (11%)	**G** (100%)	
**23,858,514**	Intron 1	**G**	**G** (100%)	**G** (100%)	**A** (22%)	**G** (100%)	
**23,858,509**	Intron 1	**G**	**G** (98%)	**A** (11%)	**G** (100%)	**G** (100%)	
**23,858,506**	Intron 1	**G**	**G** (100%)	**A** (11%)	**G** (100%)	**G** (100%)	
**23,858,428**	Intron 1	**A**	**G** (100%)	**G** (100%)	**G** (100%)	**G** (100%)	
**23,858,419**	Intron 1	**G**	**G** (100%)	**G** (100%)	**A** (15%)	**G** (100%)	
**23,858,324**	Intron 1	**C**	**C** (100%)	**T** (12%)	**T** (5%)*	**C** (100%)	
**23,858,263**	Intron 1	**C**	**C** (100%)	**C** (100%)	**T** (25%)	**C** (100%)	
**23,858,235**	Exon 2	AT<u>**G**</u>	AT<u>**G**</u> (100%)	AT<u>**A**</u> (12%)	AT<u>**G**</u> (98%)	AT<u>**G**</u> (100%)	UAC→Y_{64}<br>UAU →Y_{64}
**23,858,119**	Intron 2	**C**	**C** (100%)	**T** (11%)	**C** (100%)	**C** (100%)	
**23,858,110**	Intron 2	**C**	**C** (100%)	**T** (4%)*	**T** (11%)	**C** (100%)	
**23,858,107**	Intron 2	**G**	**G** (100%)	**G** (100%)	**A** (11%)	**G** (100%)	

**23,858,097**	Intron 2	**C**	**C** (100%)	**T** (13%)	**C** (100%)	**C** (100%)
**23,858,085**	Intron 2	**C**	**C** (100%)	**T** (4%)*	**T** (14%)	**C** (100%)
**23,858,067**	Intron 2	**C**	**C** (100%)	**C** (100%)	**T** (17%)	**C** (97%)
**23,858,062**	Intron 2	**G**	**A** (2%)*	**G** (100%)	**A** (11%)	**G** (100%)
**23,858,002**	Intron 2	**C**	**C** (100%)	**T** (11%)	**C** (100%)	**C** (100%)
**23,857,908**	Intron 2	**C**	**C** (100%)	**T** (12%)	**T** (3%)*	**C** (100%)
**23,857,868**	Intron 2	**C**	**C** (100%)	**T** (2%)*	**T** (12%)	**C** (100%)
**23,857,813**	Intron 2	**G**	**G** (100%)	**G** (100%)	**A** (12%)	**G** (100%)
**23,857,779**	Intron 2	**C**	**C** (100%)	**T** (7%)*	**T** (12%)	**C** (100%)
**23,857,772**	Intron 2	**C**	**C** (100%)	**C** (100%)	**T** (12%)	**C** (100%)
**23,857,767**	Intron 2	**C**	**C** (100%)	**T** (3%)*	**T** (17%)	**C** (100%)
**23,857,762**	Intron 2	**C**	**C** (100%)	**C** (100%)	**T** (19%)	**C** (100%)
**23,857,753**	Intron 2	**C**	**C** (100%)	**C** (100%)	**T** (23%)	**C** (100%)
**23,857,704**	Intron 2	**C**	**C** (100%)	**T** (11%)	**C** (100%)	**C** (100%)
**23,857,680**	Intron 2	**C**	**C** (100%)	**C** (100%)	**T** (14%)	**C** (100%)
**23,857,675**	Intron 2	**C**	**T** (2%)*	**T** (19%)	**T** (5%)*	**C** (100%)
**23,857,633**	Intron 2	**G**	**G** (100%)	**A** (4%)*	**A** (20%)	**G** (100%)
**23,857,624**	Intron 2	**C**	**C** (100%)	**C** (100%)	**T** (11%)	**C** (100%)
**23,857,615**	Intron 2	**C**	**C** (100%)	**C** (95%)	**T** (12%)	**C** (100%)
**23,857,603**	Intron 2	**C**	**T** (3%)*	**C** (100%)	**T** (11%)	**C** (100%)
**23,857,589**	Intron 2	**C**	**C** (100%)	**T** (5%)*	**T** (14%)	**C** (100%)
**23,857,530**	Intron 2	**G**	**G** (100%)	**A** (5%)*	**A** (11%)	**G** (100%)
**23,857,436**	Intron 2	**C**	**T** (100%)	**T** (100%)	**T** (100%)	**C** (100%)
**23,857,434**	Intron 2	**G**	**G** (100%)	**G** (100%)	**A** (14%)	**G** (100%)
**23,857,408**	Intron 2	**G**	**G** (100%)	**A** (3%)*	**A** (14%)	**G** (100%)
**23,857,366**	Intron 2	**C**	**C** (98%)	**T** (8%)*	**T** (11%)	**C** (100%)
**23,857,361**	Intron 2	**C**	**C** (100%)	**C** (100%)	**T** (15%)	**C** (100%)
**23,857,329**	Intron 2	**G**	**A** (35%)	**G** (100%)	**A** (4%)*	**G** (100%)
**23,857,261**	Intron 2	**C**	**C** (100%)	**T** (11%)	**T** (10%)*	**C** (100%)
**23,857,232**	Intron 2	**C**	**T** (2%)*	**C** (100%)	**T** (18%)	**C** (100%)
**23,857,230**	Intron 2	**C**	**C** (98%)	**C** (100%)	**T** (15%)	**C** (100%)
**23,857,228**	Intron 2	**C**	**C** (98%)	**T** (4%)*	**T** (16%)	**C** (100%)
**23,857,224**	Intron 2	**G**	**G** (100%)	**A** (5%)*	**A** (11%)	**G** (100%)
**23,857,208**	Intron 2	**G**	**G** (100%)	**A** (5%)*	**A** (11%)	**G** (100%)
**23,857,207**	Intron 2	**G**	**G** (100%)	**A** (15%)	**A** (5%)*	**G** (100%)

**23,857,206**	Intron 2	**G**	**G** (100%)	**A** (10%)*	**A** (15%)	**G** (100%)
**23,857,195**	Intron 2	**G**	**G** (100%)	**A** (4%)*	**A** (15%)	**G** (100%)
**23,857,183**	Intron 2	**G**	**G** (100%)	**A** (14%)	**A** (8%)*	**G** (100%)
**23,857,081**	Intron 2	**G**	**G** (100%)	**A** (4%)*	**A** (13%)	**G** (95%)
**23,857,075**	Intron 2	**G**	**G** (100%)	**A** (4%)*	**A** (14%)	**G** (95%)
**23,857,068**	Intron 2	**C**	**C** (98%)	**T** (4%)*	**T** (27%)	**C** (100%)
**23,857,062**	Intron 2	**G**	**G** (100%)	**G** (100%)	**A** (13%)	**G** (95%)
**23,857,058**	Intron 2	**C**	**C** (100%)	**C** (100%)	**T** (13%)	**C** (100%)
**23,857,044**	Intron 2	**C**	**C** (100%)	**C** (100%)	**T** (13%)	**T** (5%)*
**23,857,043**	Intron 2	**C**	**C** (100%)	**C** (100%)	**T** (12%)	**C** (100%)
**23,857,034**	Intron 2	**C**	**C** (100%)	**T** (7%)*	**T** (14%)	**C** (100%)
**23,857,027**	Intron 2	**C**	**C** (100%)	**T** (12%)	**T** (6%)*	**C** (100%)
**23,857,021**	Intron 2	**C**	**C** (100%)	**C** (100%)	**T** (13%)	**C** (100%)
**23,857,020**	Intron 2	**C**	**C** (100%)	**C** (100%)	**T** (11%)	**C** (100%)
**23,857,012**	Intron 2	**G**	**G** (100%)	**A** (11%)	**G** (100%)	**G** (100%)
**23,857,010**	Intron 2	**G**	**G** (100%)	**A** (12%)	**G** (100%)	**G** (100%)
**23,856,990**	Intron 2	**G**	**G** (100%)	**A** (13%)	**G** (100%)	**G** (100%)
**23,856,956**	Intron 2	**G**	**G** (100%)	**A** (13%)	**A** (3%)*	**G** (100%)
**23,856,822**	3′ UTR	**C**	**C** (100%)	**C** (100%)	**T** (11%)	**C** (100%)
**23,856,812**	3′ UTR	**C**	**C** (100%)	**T** (6%)*	**T** (14%)	**C** (100%)
**23,856,781**	3′ UTR	**G**	**G** (100%)	**G** (100%)	**A** (18%)	**G** (100%)
**23,856,763**	3′ UTR	**G**	**G** (100%)	**A** (9%)*	**A** (15%)	**G** (100%)
**23,856,748**	3′ UTR	**G**	**G** (100%)	**G** (100%)	**A** (11%)	**G** (100%)
**23,856,745**	3′ UTR	**G**	**G** (100%)	**A** (15%)	**A** (5%)*	**G** (100%)
**23,856,712**	3′ UTR	**G**	**G** (100%)	**G** (100%)	**A** (11%)	**G** (100%)
**23,856,711**	3′ UTR	**G**	**G** (100%)	**A** (9%)*	**A** (11%)	**G** (100%)
**23,856,709**	3′ UTR	**C**	**C** (100%)	**C** (95%)	**T** (11%)	**C** (100%)
**23,856,663**	3′ UTR	**T**	**C** (97%)	**C** (91%)	**C** (100%)	**C** (100%)
**23,856,661**	3′ UTR	**C**	**C** (100%)	**T** (11%)	**C** (100%)	**C** (100%)
**23,856,657**	3′ UTR	**A**	**A** (100%)	**G** (11%)	**A** (100%)	**A** (100%)
**23,856,655**	3′ UTR	**G**	**G** (100%)	**A** (11%)	**A** (8%)*	**G** (100%)
**23,856,654**	3′ UTR	**G**	**G** (100%)	**A** (6%)*	**A** (14%)	**G** (100%)
**23,856,639**	3′ UTR	**G**	**G** (100%)	**A** (5%)*	**A** (13%)	**G** (100%)
**23,856,624**	3′ UTR	**G**	**G** (100%)	**A** (12%)	**A** (12%)	**G** (100%)
**23,856,623**	3′ UTR	**G**	**G** (100%)	**G** (100%)	**A** (12%)	**G** (100%)

**23,856,601**	3′ UTR	**G**	**G** (100%)	**A** (12%)	**G** (100%)	**G** (100%)
**23,856,531**	Downstream region	**C**	**C** (98%)	**T** (11%)	**T** (4%)*	**C** (100%)
**23,856,478**	Downstream region	**C**	**C** (100%)	**T** (4%)*	**T** (13%)	**C** (100%)
**23,856,475**	Downstream region	**C**	**C** (100%)	**C** (100%)	**T** (14%)	**C** (95%)
**23,856,461**	Downstream region	**C**	**C** (100%)	**T** (4%)*	**T** (14%)	**C** (95%)
**23,856,444**	Downstream region	**C**	**C** (100%)	**C** (100%)	**T** (17%)	**C** (100%)
**23,856,432**	Downstream region	**C**	**C** (100%)	**T** (6%)*	**T** (12%)	**T** (4%)*
**23,856,407**	Downstream region	**C**	**C** (100%)	**T** (15%)	**T** (4%)*	**C** (100%)
**23,856,338**	Downstream region	**C**	**C** (100%)	**T** (11%)	**T** (2%)*	**C** (100%)
**23,856,305**	Downstream region	**G**	**G** (100%)	**A** (11%)	**A** (17%)	**G** (100%)
**23,856,263**	Downstream region	**G**	**G** (100%)	**A** (13%)	**G** (100%)	**G** (100%)
**23,856,223**	Downstream region	**C**	**C** (100%)	**T** (11%)	**T** (5%)*	**C** (100%)
**23,856,186**	Downstream region	**G**	**G** (100%)	**A** (15%)	**A** (2%)*	**G** (100%)
**23,856,165**	Downstream region	**G**	**G** (100%)	**A** (14%)	**G** (100%)	**G** (97%)
**23,856,108**	Downstream region	**C**	**C** (100%)	**C** (100%)	**T** (13%)	**C** (100%)
**23,856,092**	Downstream region	**G**	**G** (100%)	**A** (11%)	**G** (100%)	**G** (100%)
**23,856,083**	Downstream region	**G**	**G** (100%)	**A** (13%)	**G** (100%)	**G** (100%)
**23,856,029**	Downstream region	**C**	**C** (100%)	**T** (11%)	**T** (5%)*	**C** (100%)

n.a.: not available; the variant frequency is referred to the percentage of the highlighted base in the sequenced ancient hominine genome, with* frequency≤10% and ** counts<10. In light orange are underlined the variants fixed at 100% in modern human compared to ancient hominines.
