# Supplementary material for: A Catalog of Coding Sequence Variations in Salivary Proteins’ Genes Occurring during Recent Human Evolution
Source: Int J Mol Sci. 2023 Oct 9;24(19):15010. doi: 10.3390/ijms241915010 (PMC10573131; doi:10.3390/ijms241915010)
Supplement: Supplementary file 1 [file ijms-24-15010-s001.zip › Table S16 .pdf]

Table S16. Nucleotide Substitutions in *CSTA* gene in introns and regulatory regions

Genomic Coordinates (hg19)	Gene Region			Chagyrskaya (Variant Frequency)	Vindija (Variant Frequency)	Denisova (Variant Frequency)	Codon → amino acid
**122,043,603**	Upstream regions	**C**	**T** (100%)	**T** (100%)	**T** (100%)	**T** (100%)	
**122,043,745**	Upstream regions	**A**	**delA** (44%)	**A** (100%)	**A** (100%)	**A** (100%)	
**122,043,915**	Upstream regions	**C**	**C** (100%)	**C** (100%)	**T** (13%)	**C** (96%)	
**122,043,921**	Upstream regions	**C**	**C** (100%)	**T** (4%)*	**T** (19%)	**C** (100%)	
**122,043,922**	Upstream regions	**C**	**C** (100%)	**T** (9%)*	**T** (13%)	**C** (100%)	
**122,043,924**	Upstream regions	**C**	**T** (3%)*	**C** (100%)	**T** (13%)	**C** (100%)	
**122,043,947**	Upstream regions	**G**	**G** (100%)	**A** (4%)*	**A** (12%)	**G** (100%)	
**122,043,969**	Upstream regions	**G**	**G** (100%)	**A** (14%)	**G** (100%)	**G** (100%)	
**122,043,999**	Upstream regions	**C**	**C** (100%)	**T** (13%)	**C** (97%)	**C** (100%)	
**122,044,000**	Upstream regions	**C**	**C** (100%)	**T** (12%)	**C** (100%)	**C** (100%)	
**122,044,015**	5′ UTR	**G**	**G** (100%)	**G** (100%)	**A** (15%)	**G** (100%)	
**122,044,027**	5′ UTR	**T**	**C** (100%)	**C** (96%)	**C** (100%)	**T** (100%)	
**122,044,060**	5′ UTR	**T**	**T** (100%)	**T** (100%)	**T** (100%)	**C** (100%)	
**122,044,070**	5′ UTR	**C**	**C** (100%)	**T** (16%)	**T** (2%)*	**C** (100%)	
**122,044,134**	5′ UTR	**G**	**G** (100%)	**A** (12%)	**G** (100%)	**G** (100%)	
**122,044,205**	Exon 1	**AAG**	AAG (100%)	AAA (11%)	AAG (100%)	AAG (100%)	AAG→K_{22}<br>AAA→K_{22}
**122,044,505**	Intron 1	**C**	**C** (100%)	**T** (11%)	**T** (2%)*	**C** (100%)	
**122,044,573**	Intron 1	**G**	**G** (100%)	**A** (11%)	**A** (4%)*	**G** (100%)	
**122,044,641**	Intron 1	**G**	**G** (100%)	**A** (4%)*	**A** (13%)	**G** (100%)	
**122,044,660**	Intron 1	**G**	**G** (100%)	**A** (20%)	**A** (3%)*	**G** (100%)	
**122,044,705**	Intron 1	**G**	**G** (100%)	**G** (100%)	**A** (15%)	**G** (100%)	
**122,044,728**	Intron 1	**A**	**G** (100%)	**G** (100%)	**G** (100%)	**G** (100%)	
**122,044,745**	Intron 1	**G**	**G** (100%)	**A** (4%)*	**A** (12%)	**G** (100%)	
**122,044,779**	Intron 1	**G**	**A** (97%)	**A** (100%)	**A** (100%)	**G** (100%)	
**122,044,828**	Intron 1	**G**	**G** (100%)	**G** (97%)	**A** (11%)	**G** (100%)	
**122,044,848-122,044,850**	Intron 1	**CTT**	**CTT** (100%)	**CTT** (97%)	**CTT** (100%)	**delCTT** (97%)	
**122,044,889**	Intron 1	**G**	**G** (100%)	**G** (96%)	**A** (11%)	**G** (100%)	
**122,044,920**	Intron 1	**C**	**C** (100%)	**C** (100%)	**T** (11%)	**C** (100%)	
**122,044,969**	Intron 1	**G**	**A** (2%)*	**G** (100%)	**A** (14%)	**G** (97%)	

**122,044,991**	Intron 1	**G**	**G** (100%)	**A** (3%)*	**A** (14%)	**G** (100%)
**122,045,178**	Intron 1	**C**	**C** (100%)	**T** (13%)	**C** (100%)	**C** (100%)
**122,045,216**	Intron 1	**C**	**C** (98%)	**T** (11%)	**T** (5%)*	**C** (100%)
**122,045,274**	Intron 1	**C**	**T** (100%)	**T** (100%)	**T** (100%)	**T** (100%)
**122,045,618**	Intron 1	**C**	**T** (2%)*	**T** (15%)	**C** (100%)	**C** (100%)
**122,045,651**	Intron 1	**C**	**T** (100%)	**T** (100%)	**T** (100%)	**C** (100%)
**122,045,653**	Intron 1	**G**	**G** (100%)	**A** (3%)*	**A** (12%)	**G** (100%)
**122,045,716**	Intron 1	**C**	**C** (100%)	**T** (3%)*	**T** (13%)	**C** (100%)
**122,045,719**	Intron 1	**C**	**T** (2%)*	**T** (3%)*	**T** (11%)	**C** (100%)
**122,045,744**	Intron 1	**C**	**T** (3%)*	**T** (12%)	**C** (100%)	**C** (100%)
**122,045,800**	Intron 1	**G**	**G** (100%)	**A** (11%)	**G** (100%)	**G** (100%)
**122,045,943**	Intron 1	**A**	**G** (34%)	**G** (31%)	**G** (10%)*	**A** (100%)
**122,045,955**	Intron 1	**G**	**G** (100%)	**G** (100%)	**C** (11%)	**C** (3%)*
**122,046,021**	Intron 1	**G**	**G** (97%)	**A** (4%)*	**A** (11%)	**G** (100%)
**122,046,022**	Intron 1	**G**	**G** (100%)	**A** (11%)	**A** (5%)*	**G** (100%)
**122,046,049**	Intron 1	**C**	**C** (100%)	**T** (11%)	**T** (8%)*	**C** (100%)
**122,046,094**	Intron 1	**G**	**A** (9%)*	**A** (8%)*	**A** (15%)	**A** (16%)
**122,046,099**	Intron 1	**A**	**G** (60%)	**G** (35%)	**G** (32%)	**G** (6%)*
**122,046,103**	Intron 1	**G**	**A** (10%)*	**A** (18%)	**A** (10%)*	**A** (6%)*
**122,046,105**	Intron 1	**A**	**G** (2%)*	**G** (11%)	**G** (3%)*	**A** (100%)
**122,046,115**	Intron 1	**A**	**G** (38%)	**G** (23%)	**G** (19%)	**G** (26%)
**122,046,237**	Intron 1	**G**	**A** (34%)	**G** (100%)	**A** (3%)*	**G** (100%)
**122,046,375**	Intron 1	**C**	**C** (91%)	**C** (100%)**	**T** (24%)	**T** (6%)*
**122,046,376**	Intron 1	**A**	**C** (5%)*	**A** (87%)**	**C** (14%)	**C** (6%)*
**122,046,484**	Intron 1	**G**	**G** (100%)	**A** (11%)	**G** (100%)	**G** (100%)
**122,046,597**	Intron 1	**A**	**delA** (94%)	**delA** (68%)	**delA** (86%)	**delA** (95%)
**122,046,607**	Intron 1	**C**	**C** (100%)	**T** (13%)	**T** (3%)*	**C** (100%)
**122,046,627**	Intron 1	**C**	**C** (100%)	**T** (3%)*	**T** (11%)	**C** (100%)
**122,046,647**	Intron 1	**G**	**G** (100%)	**A** (4%)*	**A** (11%)	**G** (100%)
**122,046,850**	Intron 1	**C**	**C** (100%)	**T** (13%)	**C** (100%)	**C** (100%)
**122,046,860**	Intron 1	**C**	**C** (100%)	**C** (100%)	**T** (14%)	**C** (100%)
**122,046,888**	Intron 1	**G**	**G** (100%)	**A** (17%)	**A** (5%)*	**G** (100%)
**122,046,914**	Intron 1	**C**	**C** (94%)	**T** (8%)*	**T** (13%)	**T** (4%)*
**122,046,915**	Intron 1	**C**	**C** (100%)	**T** (8%)*	**T** (16%)	**C** (100%)
**122,046,950**	Intron 1	**C**	**C** (100%)	**T** (14%)	**T** (10%)*	**C** (100%)

**122,046,964**	Intron 1	**C**	**C** (100%)	**T** (5%)*	**T** (13%)	**C** (100%)
**122,047,111**	Intron 1	**G**	**G** (100%)	**G** (100%)	**A** (13%)	**G** (97%)
**122,047,205**	Intron 1	**C**	**C** (95%)	**C** (100%)	**C** (100%)	**G** (10%)*
**122,047,209**	Intron 1	**G**	**G** (100%)	**G** (100%)	**G** (100%)	**A** (53%)
**122,047,289**	Intron 1	**G**	**G** (98%)	**G** (100%)	**A** (11%)	**G** (100%)
**122,047,341**	Intron 1	**C**	**C** (100%)	**C** (92%)	**T** (12%)	**C** (100%)
**122,047,347**	Intron 1	**A**	**delA** (16%)	**delA** (9%)*	**delA** (8%)*	**delA** (30%)<br>**InsA** (30%)
**122,047,348**	Intron 1	**A**	**delA** (11%)	**delA** (10%)**	**delA** (3%)*	**delA** (10%)*
**122,047,367**	Intron 1	**G**	**A** (13%)	**G** (100%)**	**G** (100%)	**G** (100%)
**122,047,394**	Intron 1	**C**	**C** (100%)	**T** (12%)	**T** (10%)*	**C** (100%)
**122,047,419**	Intron 1	**C**	**C** (100%)	**T** (5%)*	**T** (12%)	**C** (100%)
**122,047,424**	Intron 1	**C**	**C** (100%)	**C** (100%)	**T** (18%)	**C** (100%)
**122,047,427**	Intron 1	**C**	**C** (100%)	**T** (4%)*	**T** (11%)	**C** (100%)
**122,047,528**	Intron 1	**G**	**G** (100%)	**A** (4%)*	**A** (11%)	**G** (100%)
**122,047,591**	Intron 1	**T**	**C** (86%)	**C** (68%)	**C** (61%)	**T** (100%)
**122,047,724**	Intron 1	**G**	**A** (3%)*	**A** (5%)*	**A** (13%)	**G** (100%)
**122,047,735**	Intron 1	**C**	**C** (100%)	**C** (95%)	**C** (100%)	**A** (90%)
**122,047,737**	Intron 1	**G**	**G** (100%)	**A** (52%)	**A** (33%)	**G** (100%)
**122,047,813**	Intron 1	**T**	**T** (100%)	**T** (100%)	**T** (100%)	**A** (92%)
**122,047,823**	Intron 1	**T**	**InsT** (78%)	**InsT** (34%)	**InsT** (65%)	**delT** (22%)
**122,047,833**	Intron 1	**G**	**G** (100%)	**T** (27%)	**G** (98%)	**G** (100%)
**122,047,834**	Intron 1	**A**	**A** (100%)	**G** (11%)	**A** (98%)	**A** (100%)
**122,047,869**	Intron 1	**G**	**G** (100%)	**A** (11%)	**A** (3%)*	**G** (100%)
**122,047,997**	Intron 1	**T**	**C** (2%)*	**T** (100%)	**T** (100%)	**C** (25%)
**122,048,070**	Intron 1	**C**	**C** (100%)	**T** (12%)	**T** (3%)*	**C** (100%)
**122,048,094**	Intron 1	**T**	**T** (100%)	**T** (100%)	**T** (100%)	**G** (23%)
**122,048,101**	Intron 1	**C**	**C** (100%)	**T** (13%)	**T** (2%)*	**C** (100%)
**122,048,102**	Intron 1	**C**	**C** (100%)	**T** (13%)	**C** (100%)	**T** (4%)*
**122,048,132**	Intron 1	**C**	**C** (100%)	**C** (100%)	**C** (100%)	**T** (100%)
**122,048,149**	Intron 1	**G**	**A** (5%)*	**G** (100%)	**A** (11%)	**A** (7%)*
**122,048,153**	Intron 1	**G**	**G** (100%)	**G** (100%)	**A** (13%)	**G** (100%)
**122,048,159**	Intron 1	**G**	**G** (100%)	**A** (15%)	**G** (100%)	**A** (3%)*
**122,048,180**	Intron 1	**G**	**G** (98%)	**A** (10%)*	**A** (12%)	**G** (100%)
**122,048,192**	Intron 1	**G**	**G** (100%)	**G** (100%)	**A** (12%)	**A** (3%)*

**122,048,253**	Intron 1	**T**	**T** (100%)	**C** (11%)	**T** (97%)	**T** (100%)
**122,048,254**	Intron 1	**C**	**C** (100%)	**T** (11%)	**T** (6%)*	**C** (100%)
**122,048,256**	Intron 1	**A**	**delA** (96%)	**delA** (89%)	**delA** (94%)	**A** (100%)
**122,048,285**	Intron 1	**C**	**T** (2%)*	**T** (11%)	**T** (4%)*	**C** (100%)
**122,048,321**	Intron 1	**G**	**G** (100%)	**A** (12%)	**G** (100%)	**G** (100%)
**122,048,333**	Intron 1	**C**	**C** (100%)	**T** (12%)	**T** (11%)	**C** (100%)
**122,048,526**	Intron 1	**G**	**A** (2%)*	**G** (100%)	**A** (14%)	**G** (100%)
**122,048,686**	Intron 1	**C**	**C** (100%)	**C** (97%)	**T** (11%)	**C** (100%)
**122,048,748**	Intron 1	**G**	**G** (100%)	**A** (16%)	**A** (10%)*	**G** (100%)
**122,048,788**	Intron 1	**G**	**G** (100%)	**A** (11%)	**A** (4%)*	**G** (100%)
**122,048,790**	Intron 1	**C**	**C** (100%)	**T** (11%)	**C** (100%)	**C** (100%)
**122,048,826**	Intron 1	**G**	**G** (100%)	**A** (13%)	**A** (12%)	**G** (100%)
**122,048,893**	Intron 1	**C**	**C** (100%)	**T** (6%)*	**T** (12%)	**C** (100%)
**122,048,909**	Intron 1	**G**	**G** (100%)	**A** (4%)*	**A** (13%)	**G** (100%)
**122,048,912**	Intron 1	**G**	**G** (100%)	**G** (100%)	**A** (14%)	**G** (100%)
**122,048,916**	Intron 1	**G**	**G** (100%)	**G** (100%)	**A** (17%)	**G** (100%)
**122,048,939**	Intron 1	**G**	**G** (99%)	**A** (6%)*	**A** (11%)	**G** (100%)
**122,048,976**	Intron 1	**G**	**G** (100%)	**G** (100%)	**A** (14%)	**G** (100%)
**122,048,998**	Intron 1	**G**	**G** (100%)	**A** (7%)*	**A** (11%)	**G** (100%)
**122,049,000**	Intron 1	**G**	**G** (100%)	**A** (3%)*	**A** (11%)	**G** (100%)
**122,049,018**	Intron 1	**G**	**G** (100%)	**A** (12%)	**A** (5%)*	**G** (100%)
**122,049,027**	Intron 1	**G**	**G** (100%)	**G** (100%)	**A** (13%)	**G** (100%)
**122,049,047**	Intron 1	**C**	**C** (100%)	**C** (100%)	**T** (22%)	**C** (100%)
**122,049,049**	Intron 1	**C**	**C** (100%)	**C** (100%)	**T** (11%)	**C** (100%)
**122,049,242**	Intron 1	**C**	**C** (100%)	**T** (12%)	**C** (100%)	**C** (100%)
**122,049,260**	Intron 1	**G**	**G** (100%)	**A** (3%)*	**A** (16%)	**G** (100%)
**122,049,409**	Intron 1	**C**	**C** (100%)	**C** (100%)	**T** (13%)	**C** (100%)
**122,049,602**	Intron 1	**G**	**G** (100%)	**A** (4%)*	**A** (11%)	**G** (100%)
**122,049,635**	Intron 1	**G**	**A** (3%)*	**A** (15%)	**A** (7%)*	**G** (100%)
**122,049,655**	Intron 1	**C**	**T** (2%)*	**T** (3%)*	**T** (13%)	**C** (100%)
**122,049,699**	Intron 1	**C**	**T** (2%)*	**C** (100%)	**T** (15%)	**C** (100%)
**122,049,712**	Intron 1	**A**	**T** (98%)	**T** (100%)	**T** (100%)	**A** (100%)
**122,049,917**	Intron 1	**G**	**G** (100%)	**A** (3%)*	**A** (11%)	**G** (100%)
**122,049,931**	Intron 1	**C**	**T** (2%)*	**C** (100%)	**T** (13%)	**C** (100%)

**122,049,952-122,049,955**	Intron 1	**ACAC**	**delAC** (6%)	**ACAC** (95%)	**ACAC** (95%)	**delACAC** (24%)
**122,049,957**	Intron 1	**C**	**C** (98%)	**A** (5%)*	**A** (5%)*	**A** (13%)
**122,050,067**	Intron 1	**C**	**C** (100%)	**T** (5%)*	**T** (11%)	**C** (100%)
**122,050,159**	Intron 1	**C**	**T** (2%)*	**T** (15%)	**C** (100%)	**C** (100%)
**122,050,228**	Intron 1	**G**	**G** (100%)	**G** (100%)	**A** (11%)	**G** (100%)
**122,050,264**	Intron 1	**C**	**C** (100%)	**C** (100%)	**T** (13%)	**C** (100%)
**122,050,267**	Intron 1	**C**	**C** (100%)	**C** (100%)	**T** (13%)	**C** (100%)
**122,050,297**	Intron 1	**C**	**C** (100%)	**C** (100%)	**T** (13%)	**C** (100%)
**122,050,299**	Intron 1	**C**	**C** (100%)	**T** (11%)	**T** (4%)*	**C** (100%)
**122,050,326**	Intron 1	**G**	**G** (100%)	**A** (14%)	**A** (4%)*	**G** (100%)
**122,050,342**	Intron 1	**C**	**C** (100%)	**C** (96%)	**T** (21%)	**C** (100%)
**122,050,343**	Intron 1	**C**	**C** (100%)	**C** (100%)	**T** (16%)	**C** (100%)
**122,050,404**	Intron 1	**G**	**A** (2%)*	**A** (13%)	**A** (4%)*	**G** (100%)
**122,050,530**	Intron 1	**C**	**C** (100%)	**T** (13%)	**T** (3%)*	**C** (100%)
**122,050,531**	Intron 1	**C**	**T** (2%)*	**T** (13%)	**T** (8%)*	**C** (100%)
**122,050,568**	Intron 1	**C**	**C** (100%)	**C** (100%)	**T** (11%)	**C** (100%)
**122,050,623**	Intron 1	**T**	**C** (100%)	**C** (97%)	**C** (100%)	**C** (100%)
**122,050,648**	Intron 1	**C**	**C** (100%)	**T** (5%)*	**T** (13%)	**T** (5%)*
**122,050,679**	Intron 1	**C**	**C** (100%)	**C** (100%)	**T** (13%)	**C** (100%)
**122,050,748**	Intron 1	**G**	**G** (100%)	**A** (13%)	**A** (11%)	**G** (100%)
**122,050,749**	Intron 1	**G**	**G** (100%)	**A** (23%)	**A** (6%)*	**G** (100%)
**122,050,750**	Intron 1	**G**	**G** (100%)	**A** (18%)	**A** (6%)*	**G** (100%)
**122,050,776**	Intron 1	**C**	**C** (100%)	**T** (4%)*	**T** (11%)	**C** (100%)
**122,050,777**	Intron 1	**C**	**C** (100%)	**T** (4%)*	**T** (11%)	**C** (100%)
**122,050,811**	Intron 1	**C**	**C** (100%)	**C** (100%)	**T** (13%)	**C** (100%)
**122,050,893**	Intron 1	**G**	**A** (3%)*	**A** (11%)	**G** (100%)	**G** (100%)
**122,050,925**	Intron 1	**C**	**C** (100%)	**T** (4%)*	**T** (11%)	**C** (100%)
**122,050,981**	Intron 1	**G**	**G** (100%)	**A** (3%)*	**A** (11%)	**G** (100%)
**122,050,995**	Intron 1	**G**	**G** (97%)	**G** (100%)	**A** (11%)	**G** (100%)
**122,050,996**	Intron 1	**G**	**G** (100%)	**G** (100%)	**A** (19%)	**G** (100%)
**122,051,080**	Intron 1	**C**	**C** (100%)	**T** (14%)	**C** (100%)	**C** (100%)
**122,051,082**	Intron 1	**C**	**C** (100%)	**C** (100%)	**T** (13%)	**C** (100%)
**122,051,084**	Intron 1	**C**	**T** (3%)*	**C** (100%)	**T** (15%)	**C** (100%)
**122,051,159**	Intron 1	**C**	**C** (100%)	**T** (5%)*	**T** (5%)*	**T** (40%)

**122,051,276**	Intron 1	**C**	**C** (100%)	**T** (4%)*	**T** (19%)	**C** (100%)
**122,051,278**	Intron 1	**C**	**C** (100%)	**C** (100%)	**T** (13%)	**C** (96%)
**122,051,288**	Intron 1	**C**	**C** (100%)	**T** (12%)	**T** (8%)*	**C** (100%)
**122,051,412**	Intron 1	**C**	**C** (98%)	**T** (5%)*	**T** (13%)	**C** (100%)
**122,051,449**	Intron 1	**C**	**C** (100%)	**C** (100%)	**T** (15%)	**C** (100%)
**122,051,450**	Intron 1	**C**	**C** (100%)	**T** (11%)	**T** (6%)*	**C** (100%)
**122,051,556**	Intron 1	**C**	**C** (100%)	**T** (4%)*	**T** (15%)	**C** (100%)
**122,051,595**	Intron 1	**C**	**C** (100%)	**T** (11%)	**C** (100%)	**C** (100%)
**122,051,603**	Intron 1	**C**	**T** (4%)*	**T** (11%)	**C** (100%)	**T** (3%)*
**122,051,623**	Intron 1	**G**	**G** (100%)	**G** (100%)	**A** (11%)	**A** (2%)*
**122,051,631**	Intron 1	**C**	**C** (100%)	**T** (5%)*	**T** (11%)	**C** (100%)
**122,051,667**	Intron 1	**C**	**C** (100%)	**T** (13%)	**C** (100%)	**C** (100%)
**122,051,681**	Intron 1	**C**	**T** (4%)*	**T** (12%)	**T** (9%)*	**T** (4%)*
**122,051,687**	Intron 1	**C**	**C** (100%)	**T** (11%)	**T** (4%)*	**C** (100%)
**122,051,797**	Intron 1	**C**	**C** (100%)	**T** (13%)	**C** (100%)	**C** (100%)
**122,051,805**	Intron 1	**C**	**C** (100%)	**T** (13%)	**C** (100%)	**C** (100%)
**122,051,821**	Intron 1	**C**	**C** (100%)	**T** (16%)	**C** (100%)	**C** (100%)
**122,051,918**	Intron 1	**G**	**G** (100%)	**A** (14%)	**A** (3%)*	**G** (100%)
**122,051,959**	Intron 1	**C**	**T** (2%)*	**T** (11%)	**T** (5%)*	**C** (100%)
**122,052,118**	Intron 1	**G**	**G** (100%)	**G** (96%)	**A** (12%)	**G** (100%)
**122,052,131**	Intron 1	**G**	**A** (2%)*	**A** (15%)	**A** (4%)*	**G** (100%)
**122,052,152**	Intron 1	**G**	**G** (100%)	**G** (100%)	**A** (18%)	**G** (100%)
**122,052,158**	Intron 1	**G**	**G** (98%)	**G** (100%)	**A** (11%)	**G** (100%)
**122,052,177**	Intron 1	**G**	**G** (100%)	**A** (3%)*	**A** (14%)	**G** (100%)
**122,052,228**	Intron 1	**G**	**G** (95%)	**A** (3%)*	**A** (16%)	**G** (100%)
**122,052,387**	Intron 1	**G**	**A** (2%)*	**A** (12%)	**A** (4%)*	**A** (4%)*
**122,052,426**	Intron 1	**G**	**G** (100%)	**A** (13%)	**A** (3%)*	**G** (100%)
**122,052,430**	Intron 1	**G**	**G** (100%)	**A** (12%)	**G** (100%)	**A** (5%)*
**122,052,441**	Intron 1	**G**	**A** (58%)	**A** (46%)	**A** (54%)	**G** (100%)
**122,052,442**	Intron 1	**A**	**G** (60%)	**G** (50%)	**G** (52%)	**A** (100%)
**122,052,475**	Intron 1	**G**	**G** (100%)	**A** (17%)	**A** (11%)	**G** (100%)
**122,052,515**	Intron 1	**G**	**A** (2%)*	**A** (19%)	**A** (6%)*	**G** (100%)
**122,052,529**	Intron 1	**G**	**G** (100%)	**A** (14%)	**G** (100%)	**G** (100%)
**122,052,541**	Intron 1	**G**	**A** (26%)	**A** (27%)	**A** (23%)	**A** (12%)
**122,052,543**	Intron 1	**G**	**A** (3%)*	**A** (14%)	**A** (7%)*	**A** (8%)*

**122,052,545**	Intron 1	**G**	**A** (2%)*	**G** (100%)	**A** (11%)	**G** (100%)
**122,052,567**	Intron 1	**G**	**A** (6%)*	**A** (21%)	**A** (8%)*	**G** (100%)
**122,052,577**	Intron 1	**G**	**G** (100%)	**A** (13%)	**A** (9%)*	**G** (100%)
**122,052,579**	Intron 1	**A**	**A** (100%)	**G** (13%)	**G** (76%)	**G** (75%)
**122,052,580**	Intron 1	**A**	**delA** (17%)	**delA** (5%)*	**A** (94%)	**A** (96%)
**122,052,581**	Intron 1	**G**	**delG** (20%)	**A** (19%)<br>**delG** (6%)*	**A** (33%)	**A** (87%)
**122,052,582**	Intron 1	**A**	**G** (31%)	**G** (6%)*	**G** (6%)*	**G** (8%)*
**122,052,583**	Intron 1	**G**	**A** (13%)	**A** (12%)	**A** (3%)*	**G** (100%)
**122,052,591**	Intron 1	**G**	**A** (5%)*	**G** (100%)	**A** (16%)	**G** (100%)
**122,052,594**	Intron 1	**A**	**G** (12%)	**A** (100%)	**A** (100%)	**A** (100%)
**122,052,596**	Intron 1	**G**	**A** (15%)	**G** (100%)	**A** (15%)	**A** (4%)*
**122,052,601**	Intron 1	**G**	**A** (8%)*	**G** (100%)	**A** (11%)	**G** (100%)
**122,052,606**	Intron 1	**G**	**G** (100%)	**C** (55%)	**C** (23%)	**G** (96%)
**122,052,658**	Intron 1	**G**	**G** (100%)	**A** (13%)	**G** (100%)	**A** (4%)*
**122,052,659**	Intron 1	**G**	**G** (100%)	**A** (22%)	**A** (5%)*	**G** (100%)
**122,052,706**	Intron 1	**C**	**C** (99%)	**T** (5%)*	**T** (14%)	**C** (100%)
**122,052,741**	Intron 1	**G**	**G** (100%)	**A** (4%)*	**A** (11%)	**G** (100%)
**122,052,759**	Intron 1	**C**	**G** (100%)	**G** (97%)	**G** (88%)	**G** (100%)
**122,052,781**	Intron 1	**G**	**G** (100%)	**A** (13%)	**A** (3%)*	**G** (100%)
**122,052,801**	Intron 1	**G**	**G** (97%)	**G** (100%)	**A** (11%)	**G** (100%)
**122,052,802**	Intron 1	**G**	**G** (100%)	**A** (12%)	**A** (4%)*	**G** (100%)
**122,052,889**	Intron 1	**G**	**G** (100%)	**A** (12%)	**G** (100%)	**G** (100%)
**122,052,901**	Intron 1	**G**	**G** (100%)	**A** (10%)*	**A** (11%)	**G** (100%)
**122,052,920**	Intron 1	**G**	**G** (100%)	**A** (11%)	**A** (6%)*	**G** (100%)
**122,052,931**	Intron 1	**C**	**T** (2%)*	**T** (7%)*	**T** (16%)	**C** (100%)
**122,053,120**	Intron 1	**C**	**C** (100%)	**T** (14%)	**T** (2%)*	**C** (97%)
**122,053,157**	Intron 1	**T**	**T** (100%)	**T** (100%)	**C** (36%)	**T** (100%)
**122,053,163**	Intron 1	**C**	**C** (100%)	**C** (97%)	**T** (15%)	**C** (100%)
**122,053,175**	Intron 1	**C**	**C** (100%)	**T** (11%)	**C** (100%)	**C** (100%)
**122,053,198**	Intron 1	**C**	**C** (100%)	**T** (11%)	**C** (100%)	**C** (100%)
**122,053,226**	Intron 1	**C**	**C** (100%)	**T** (18%)	**C** (100%)	**C** (100%)
**122,053,227**	Intron 1	**C**	**C** (100%)	**T** (11%)	**T** (3%)*	**C** (100%)
**122,053,249**	Intron 1	**G**	**A** (100%)	**A** (100%)	**A** (100%)	**A** (100%)
**122,053,285**	Intron 1	**C**	**C** (100%)	**T** (21%)	**C** (100%)	**C** (100%)

**122,053,354**	Intron 1	**C**	**C** (100%)	**T** (12%)	**T** (5%)*	**C** (100%)
**122,053,404**	Intron 1	**G**	**G** (100%)	**G** (96%)	**A** (3%)*	**A** (52%)
**122,053,435**	Intron 1	**C**	**C** (100%)	**T** (11%)	**C** (100%)	**C** (100%)
**122,053,575**	Intron 1	**G**	**A** (2%)*	**A** (11%)	**G** (100%)	**G** (100%)
**122,053,627**	Intron 1	**G**	**G** (99%)	**A** (11%)	**A** (7%)*	**G** (100%)
**122,053,676**	Intron 1	**G**	**G** (100%)	**A** (9%)*	**A** (11%)	**G** (100%)
**122,053,723**	Intron 1	**C**	**C** (100%)	**C** (100%)	**T** (11%)	**C** (100%)
**122,053,744**	Intron 1	**C**	**C** (100%)	**C** (100%)	**T** (17%)	**C** (100%)
**122,053,756**	Intron 1	**G**	**A** (3%)*	**G** (100%)	**A** (14%)	**G** (100%)
**122,053,766**	Intron 1	**G**	**G** (100%)	**A** (11%)	**G** (100%)	**G** (100%)
**122,053,815**	Intron 1	**G**	**G** (100%)	**A** (13%)	**A** (7%)*	**G** (100%)
**122,053,853**	Intron 1	**C**	**T** (3%)*	**T** (11%)	**C** (100%)	**C** (100%)
**122,053,892**	Intron 1	**G**	**G** (100%)	**A** (11%)	**A** (21%)	**G** (100%)
**122,053,948**	Intron 1	**G**	**G** (100%)	**G** (100%)	**A** (12%)	**G** (100%)
**122,054,004**	Intron 1	**G**	**G** (100%)	**A** (18%)	**A** (6%)*	**G** (100%)
**122,054,006**	Intron 1	**C**	**T** (2%)*	**T** (21%)	**C** (100%)	**C** (100%)
**122,054,008**	Intron 1	**C**	**C** (100%)	**T** (11%)	**C** (100%)	**C** (100%)
**122,054,131**	Intron 1	**C**	**T** (2%)*	**C** (100%)	**T** (13%)	**C** (100%)
**122,054,250**	Intron 1	**C**	**C** (100%)	**T** (12%)	**T** (11%)	**C** (100%)
**122,054,252**	Intron 1	**C**	**T** (100%)	**T** (100%)	**T** (100%)	**C** (100%)
**122,054,333**	Intron 1	**G**	**G** (100%)	**A** (12%)	**A** (8%)*	**G** (100%)
**122,054,350**	Intron 1	**G**	**A** (100%)	**A** (100%)	**A** (100%)	**A** (100%)
**122,054,461**	Intron 1	**T**	**T** (100%)	**T** (100%)	**T** (100%)	**InsTTAT** (57%)
**122,054,836**	Intron 1	**A**	**A** (100%)	**A** (100%)	**A** (97%)	**InsGT** (14%)
**122,054,839**	Intron 1	**C**	**C** (100%)	**C** (100%)	**T** (13%)	**C** (100%)
**122,054,929**	Intron 1	**A**	**A** (100%)	**G** (64%)	**G** (39%)	**A** (100%)
**122,054,943**	Intron 1	**G**	**G** (100%)	**A** (3%)*	**A** (12%)	**G** (100%)
**122,055,067**	Intron 1	**G**	**G** (100%)	**A** (6%)*	**A** (11%)	**G** (100%)
**122,055,124**	Intron 1	**C**	**C** (100%)	**T** (13%)	**C** (100%)	**C** (100%)
**122,055,346**	Intron 1	**G**	**G** (100%)	**A** (3%)*	**A** (13%)	**G** (100%)
**122,055,360**	Intron 1	**C**	**C** (100%)	**T** (11%)	**T** (3%)*	**C** (100%)
**122,055,429**	Intron 1	**C**	**C** (100%)	**T** (4%)*	**T** (11%)	**C** (100%)
**122,055,432**	Intron 1	**C**	**C** (100%)	**T** (13%)	**C** (100%)	**C** (100%)
**122,055,497**	Intron 1	**G**	**A** (100%)	**A** (100%)	**A** (100%)	**G** (100%)

**122,055,498**	Intron 1	**C**	**C** (98%)	**T** (4%)*	**T** (11%)	**C** (100%)
**122,055,541**	Intron 1	**G**	**G** (100%)	**A** (12%)	**A** (6%)*	**G** (100%)
**122,055,670**	Intron 1	**C**	**T** (2%)*	**C** (100%)	**T** (13%)	**C** (100%)
**122,055,689**	Intron 1	**C**	**C** (100%)	**T** (4%)*	**T** (14%)	**C** (100%)
**122,055,728**	Intron 1	**C**	**C** (100%)	**T** (11%)	**T** (20%)	**C** (100%)
**122,055,736**	Intron 1	**C**	**C** (100%)	**C** (100%)	**T** (11%)	**C** (100%)
**122,055,820**	Intron 1	**T**	**T** (100%)	**T** (100%)	**C** (56%)	**T** (100%)
**122,055,919**	Intron 1	**C**	**T** (3%)*	**T** (13%)	**T** (3%)*	**C** (100%)
**122,055,965**	Intron 1	**C**	**C** (100%)	**T** (6%)*	**T** (19%)	**C** (100%)
**122,056,120**	Intron 1	**C**	**C** (100%)	**T** (5%)*	**T** (11%)	**C** (100%)
**122,056,176**	Intron 1	**G**	**G** (100%)	**G** (100%)	**A** (12%)	**G** (100%)
**122,056,223**	Intron 1	**C**	**C** (99%)	**T** (11%)	**C** (100%)	**C** (100%)
**122,056,309**	Intron 1	**G**	**G** (100%)	**A** (4%)*	**A** (11%)	**G** (100%)
**122,056,330**	Intron 1	**G**	**G** (100%)	**A** (4%)*	**A** (11%)	**G** (100%)
**122,056,333**	Intron 1	**C**	**C** (100%)	**C** (100%)	**T** (11%)	**C** (100%)
**122,056,496**	Intron 2	**G**	**G** (100%)	**G** (100%)	**A** (28%)	**G** (100%)
**122,056,508**	Intron 2	**C**	**C** (100%)	**T** (8%)*	**T** (17%)	**C** (97%)
**122,056,510**	Intron 2	**C**	**C** (100%)	**T** (4%)*	**T** (17%)	**C** (100%)
**122,056,591**	Intron 2	**G**	**G** (100%)	**G** (100%)	**A** (16%)	**G** (100%)
**122,056,748**	Intron 2	**C**	**C** (98%)	**T** (11%)	**C** (100%)	**C** (100%)
**122,056,824**	Intron 2	**G**	**A** (3%)*	**A** (12%)	**A** (6%)*	**G** (100%)
**122,056,825**	Intron 2	**G**	**G** (100%)	**A** (13%)	**A** (6%)*	**G** (100%)
**122,056,836**	Intron 2	**G**	**G** (100%)	**A** (4%)*	**A** (11%)	**G** (100%)
**122,056,839**	Intron 2	**C**	**C** (100%)	**T** (11%)	**T** (6%)*	**C** (100%)
**122,056,948**	Intron 2	**C**	**C** (100%)	**T** (13%)	**T** (3%)*	**C** (97%)
**122,056,982**	Intron 2	**C**	**C** (100%)	**T** (12%)	**T** (7%)*	**T** (3%)*
**122,057,024**	Intron 2	**G**	**G** (100%)	**A** (12%)	**A** (3%)*	**G** (100%)
**122,057,084**	Intron 2	**A**	**InsA** (93%)	**InsA** (80%)	**InsA** (84%)	**A** (100%)
**122,057,090**	Intron 2	**T**	**T** (100%)	**A** (11%)	**T** (97%)	**T** (100%)
**122,057,110**	Intron 2	**G**	**G** (100%)	**G** (100%)	**A** (11%)	**G** (100%)
**122,057,257**	Intron 2	**C**	**C** (100%)	**C** (96%)	**T** (15%)	**C** (100%)
**122,057,343**	Intron 2	**C**	**T** (4%)*	**T** (14%)	**C** (100%)	**C** (100%)
**122,057,347**	Intron 2	**A**	**delA** (51%)	**delA** (83%)	**delA** (50%)	**delA** (47%)
**122,057,348**	Intron 2	**A**	**delA** (19%)	**delA** (38%)	**delA** (30%)	**delA** (30%)
**122,057,349**	Intron 2	**A**	**delA** (7%)*	**delA** (15%)	**delA** (6%)*	**delA** (13%)

**122,057,381**	Intron 2	**G**	**G** (100%)	**G** (100%)	**A** (14%)	**G** (100%)
**122,057,408**	Intron 2	**C**	**C** (100%)	**C** (95%)	**T** (14%)	**C** (100%)
**122,057,483**	Intron 2	**G**	**G** (95%)	**A** (14%)	**A** (5%)*	**G** (100%)
**122,057,624**	Intron 2	**C**	**C** (99%)	**T** (4%)*	**T** (14%)	**C** (100%)
**122,057,682**	Intron 2	**C**	**C** (98%)	**T** (12%)	**T** (9%)*	**C** (100%)
**122,057,690**	Intron 2	**C**	**C** (100%)	**T** (12%)	**C** (100%)	**C** (100%)
**122,057,705**	Intron 2	**G**	**A** (2%)*	**A** (12%)	**A** (8%)*	**G** (100%)
**122,057,748**	Intron 2	**T**	**T** (100%)	**T** (100%)	**A** (65%)	**T** (100%)
**122,057,767**	Intron 2	**G**	**G** (100%)	**G** (100%)	**A** (11%)	**G** (95%)
**122,057,769**	Intron 2	**C**	**C** (100%)	**C** (100%)	**T** (18%)	**C** (100%)
**122,057,787**	Intron 2	**C**	**C** (100%)	**C** (96%)	**T** (12%)	**delC** (18%)
**122,057,788**	Intron 2	**T**	**T** (94%)	**T** (96%)	**T** (98%)	**delT** (18%)
**122,057,789**	Intron 2	**C**	**C** (98%)	**T** (4%)*	**T** (11%)	**C** (100%)
**122,057,793**	Intron 2	**C**	**C** (100%)	**C** (100%)	**T** (14%)	**C** (100%)
**122,057,795**	Intron 2	**C**	**C** (100%)	**C** (100%)	**T** (14%)	**C** (100%)
**122,057,810**	Intron 2	**C**	**C** (100%)	**T** (13%)	**C** (100%)	**C** (100%)
**122,057,918**	Intron 2	**T**	**C** (97%)	**C** (97%)	**C** (100%)	**T** (100%)
**122,058,016**	Intron 2	**G**	**G** (100%)	**A** (3%)*	**A** (11%)	**G** (100%)
**122,058,076**	Intron 2	**C**	**C** (100%)	**T** (12%)	**C** (100%)	**C** (100%)
**122,058,122**	Intron 2	**C**	**T** (3%)*	**T** (12%)	**T** (3%)*	**C** (100%)
**122,058,194**	Intron 2	**G**	**G** (100%)	**A** (12%)	**G** (100%)	**G** (100%)
**122,058,195**	Intron 2	**G**	**G** (97%)	**A** (12%)	**A** (3%)*	**G** (100%)
**122,058,264**	Intron 2	**G**	**G** (100%)	**G** (100%)	**A** (11%)	**G** (100%)
**122,058,285**	Intron 2	**G**	**G** (100%)	**A** (11%)	**A** (4%)*	**G** (100%)
**122,058,318**	Intron 2	**G**	**G** (100%)	**G** (100%)	**A** (13%)	**G** (100%)
**122,058,389**	Intron 2	**C**	**C** (100%)	**C** (100%)	**T** (12%)	**C** (100%)
**122,058,481**	Intron 2	**C**	**T** (2%)*	**T** (4%)*	**T** (11%)	**C** (100%)
**122,058,579**	Intron 2	**G**	**G** (100%)	**G** (100%)	**A** (11%)	**G** (100%)
**122,058,624**	Intron 2	**C**	**C** (100%)	**T** (12%)	**C** (100%)	**C** (100%)
**122,058,637**	Intron 2	**G**	**G** (100%)	**A** (7%)*	**A** (14%)	**G** (100%)
**122,058,638**	Intron 2	**G**	**G** (100%)	**G** (100%)	**A** (20%)	**G** (100%)
**122,058,646**	Intron 2	**G**	**G** (100%)	**G** (100%)	**A** (12%)	**G** (100%)
**122,058,658**	Intron 2	**C**	**T** (3%)*	**C** (100%)	**T** (12%)	**C** (100%)
**122,058,660**	Intron 2	**C**	**C** (100%)	**T** (14%)	**T** (5%)*	**C** (100%)
**122,058,724**	Intron 2	**G**	**G** (100%)	**A** (11%)	**A** (4%)*	**G** (100%)

**122,059,006**	Intron 2	**G**	**G** (100%)	**G** (100%)	**A** (11%)	**G** (100%)	
**122,059,070**	Intron 2	**C**	**C** (98%)	**T** (11%)	**T** (2%)*	**C** (100%)	
**122,059,175**	Intron 2	**G**	**A** (2%)*	**A** (42%)	**A** (3%)*	**G** (100%)	
**122,059,239**	Intron 2	**C**	**C** (98%)	**T** (3%)*	**T** (11%)	**C** (100%)	
**122,059,445**	Intron 2	**C**	**C** (100%)	**C** (100%)	**T** (13%)	**C** (100%)	
**122,059,447**	Intron 2	**G**	**G** (100%)	**G** (100%)	**A** (13%)	**G** (100%)	
**122,059,456**	Intron 2	**C**	**C** (100%)	**T** (4%)*	**T** (13%)	**C** (100%)	
**122,059,474**	Intron 2	**G**	**G** (97%)	**A** (24%)	**G** (100%)	**G** (100%)	
**122,059,475**	Intron 2	**G**	**A** (3%)*	**A** (11%)	**G** (100%)	**G** (100%)	
**122,059,491**	Intron 2	**C**	**C** (100%)	**T** (18%)	**C** (100%)	**C** (100%)	
**122,059,495**	Intron 2	**G**	**A** (3%)*	**G** (95%)	**A** (14%)	**G** (100%)	
**122,059,664**	Intron 2	**C**	**C** (100%)	**C** (100%)	**T** (13%)	**C** (100%)	
**122,059,761**	Intron 2	**C**	**T** (2%)*	**T** (11%)	**T** (12%)	**C** (100%)	
**122,059,792**	Intron 2	**T**	**delT** (63%)	**delT** (44%)	**delT** (54%)	**delT** (61%)	
**122,059,807**	Intron 2	**A**	**A** (100%)	**T** (13%)	**T** (3%)*	**A** (100%)	
**122,059,809**	Intron 2	**G**	**G** (100%)	**A** (18%)	**G** (100%)	**A** (6%)*	
**122,059,815**	Intron 2	**C**	**C** (100%)	**A** (6%)*	**A** (13%)	**C** (97%)	
**122,059,911**	Intron 2	**C**	**C** (99%)	**T** (3%)*	**T** (14%)	**C** (100%)	
**122,060,031**	Intron 2	**C**	**C** (100%)	**T** (11%)	**C** (100%)	**C** (100%)	
**122,060,032**	Intron 2	**C**	**T** (3%)*	**T** (11%)	**C** (100%)	**C** (100%)	
**122,060,063**	Intron 2	**C**	**C** (100%)	**T** (15%)	**C** (100%)	**C** (100%)	
**122,060,085**	Intron 2	**G**	**G** (100%)	**G** (100%)	**A** (13%)	**G** (100%)	
**122,060,173**	Intron 2	**G**	**G** (100%)	**A** (3%)*	**A** (11%)	**G** (100%)	
**122,060,372**	Exon 3	**TAC**	TAC (100%)	TAC (100%)	TAT (12%)	TAC (100%)	UAC→Y_{85}<br>UAU→Y_{85}
**122,060,639**	3′ UTR	**T**	**T** (100%)	**T** (100%)	**T** (100%)	**C** (48%)	
**122,060,653**	3′ UTR	**G**	**A** (96%)	**A** (100%)	**A** (100%)	**G** (100%)	
**122,060,654**	3′ UTR	**C**	**C** (100%)	**T** (4%)*	**T** (14%)	**C** (100%)	
**122,060,678**	3′ UTR	**G**	**G** (100%)	**A** (13%)	**G** (100%)	**G** (100%)	
**122,060,686**	3′ UTR	**C**	**C** (100%)	**C** (100%)	**T** (17%)	**C** (100%)	
**122,060,690**	3′ UTR	**C**	**C** (100%)	**T** (12%)	**T** (6%)*	**C** (100%)	
**122,060,692**	3′ UTR	**C**	**C** (100%)	**T** (6%)*	**T** (14%)	**C** (100%)	
**122,060,697**	3′ UTR	**C**	**C** (100%)	**C** (100%)	**T** (13%)	**C** (100%)	
**122,060,706**	3′ UTR	**G**	**G** (100%)	**A** (13%)	**A** (4%)*	**G** (100%)	
**122,060,741**	3′ UTR	**G**	**G** (100%)	**G** (96%)	**A** (13%)	**G** (96%)	

**122,060,823**	Downstream regions	**G**	**G** (100%)	**A** (11%)	**A** (6%)*	**G** (100%)
**122,060,825**	Downstream regions	**G**	**G** (100%)	**A** (11%)	**A** (6%)*	**G** (100%)
**122,060,827**	Downstream regions	**G**	**G** (100%)	**G** (100%)	**A** (13%)	**G** (100%)
**122,060,831**	Downstream regions	**G**	**G** (100%)	**A** (13%)	**G** (100%)	**G** (100%)
**122,060,839**	Downstream regions	**G**	**A** (32%)	**A** (38%)	**A** (43%)	**G** (100%)
**122,060,848**	Downstream regions	**T**	**C** (22%)	**C** (13%)**	**C** (13%)	**T** (100%)
**122,060,849**	Downstream regions	**A**	**T** (28%)	**T** (13%)**	**T** (22%)	**A** (100%)
**122,060,850**	Downstream regions	**C**	**T** (28%)	**T** (25%)**	**T** (10%)*	**T** (27%)
**122,060,870**	Downstream regions	**T**	**T** (91%)	**C** (20%)	**T** (100%)	**T** (100%)
**122,060,874**	Downstream regions	**G**	**A** (5%)*	**G** (100%)**	**G** (100%)	**A** (18%)
**122,060,895**	Downstream regions	**C**	**T** (2%)*	**T** (11%)	**T** (3%)*	**C** (100%)
**122,060,974**	Downstream regions	**C**	**C** (100%)	**C** (100%)	**T** (7%)*	**T** (18%)
**122,060,978**	Downstream regions	**C**	**C** (100%)	**T** (3%)*	**T** (4%)*	**T** (13%)
**122,061,011**	Downstream regions	**G**	**A** (35%)	**A** (4%)*	**G** (100%)	**G** (100%)
**122,061,152**	Downstream regions	**T**	**C** (98%)	**C** (90%)	**C** (88%)	**C** (100%)
**122,061,182**	Downstream regions	**C**	**T** (1%)*	**T** (13%)	**T** (3%)*	**C** (100%)
**122,061,198**	Downstream regions	**A**	**delA** (6%)*	**delA** (16%)	**InsA** (26%) **delA** (6%)*	**InsA** (7%)* **delA** (2%)*

n.a.: not available; the variant frequency is referred to the percentage of the highlighted base in the sequenced ancient hominine genome, with* frequency≤10% and ** counts<10. In light orange are underlined the variants fixed at 100% in modern human compared to ancient hominines.
