# Supplementary material for: A Catalog of Coding Sequence Variations in Salivary Proteins’ Genes Occurring during Recent Human Evolution
Source: Int J Mol Sci. 2023 Oct 9;24(19):15010. doi: 10.3390/ijms241915010 (PMC10573131; doi:10.3390/ijms241915010)
Supplement: Supplementary file 1 [file ijms-24-15010-s001.zip › Table S17 .pdf]

Table S17. Nucleotide Substitutions in *CSTB* gene in introns and regulatory regions

Genomic Coordinates (hg19)	Gene Region	Modern human	Altai Neanderthal (Variant Frequency)	Chagyrskaya Neanderthal (Variant Frequency)	Vindija Neanderthal (Variant Frequency)	Denisovan (Variant Frequency)	Codon → amino acid
**45,196,797**	Upstream regions	**G**	**G** (100%)	**A** (11%)	**G** (100%)	**G** (100%)	
**45,196,795**	Upstream regions	**G**	**G** (100%)	**A** (5%)*	**A** (11%)	**G** (100%)	
**45,196,777**	Upstream regions	**G**	**G** (100%)	**A** (11%)	**A** (8%)*	**G** (100%)	
**45,196,773**	Upstream regions	**G**	**G** (100%)	**A** (12%)	**G** (100%)	**G** (100%)	
**45,196,771**	Upstream regions	**G**	**G** (100%)	**A** (18%)	**G** (100%)	**G** (100%)	
**45,196,766**	Upstream regions	**G**	**G** (100%)	**G** (100%)	**A** (15%)	**G** (100%)	
**45,196,739**	Upstream regions	**G**	**G** (100%)	**A** (20%)	**G** (100%)	**G** (100%)**	
**45,196,724**	Upstream regions	**G**	**G** (100%)	**A** (14%)**	**A** (13%)	**G** (100%)**	
**45,196,712**	Upstream regions	**G**	**G** (100%)	**G** (98%)	**A** (17%)	**G** (100%)	
**45,196,691**	Upstream regions	**G**	**G** (100%)	**G** (100%)	**A** (11%)	**G** (100%)	
**45,196,667**	Upstream regions	**C**	**T** (4%)*	**T** (13%)	**C** (100%)	**C** (100%)	
**45,196,641**	Upstream regions	**C**	**C** (100%)	**C** (100%)	**T** (13%)	**C** (100%)	
**45,196,634**	Upstream regions	**G**	**G** (100%)	**G** (100%)	**A** (29%)	**G** (100%)**	
**45,196,633**	Upstream regions	**G**	**G** (100%)	**A** (18%)	**A** (7%)*	**G** (100%)	
**45,196,614**	Upstream regions	**G**	**G** (100%)	**G** (100%)	**A** (17%)	**G** (100%)	
**45,196,603**	Upstream regions	**G**	**G** (100%)	**G** (100%)	**A** (17%)	**G** (100%)	
**45,196,599**	Upstream regions	**G**	**G** (100%)	**G** (100%)	**A** (15%)	**G** (100%)	
**45,196,567**	Upstream regions	**G**	**G** (100%)	**G** (100%)	**A** (18%)	**G** (100%)	
**45,196,566**	Upstream regions	**G**	**G** (95%)	**G** (100%)	**A** (17%)	**G** (100%)	
**45,196,494**	Upstream regions	**G**	**G** (100%)	**A** (23%)	**G** (100%)	**G** (100%)**	
**45,196,374**	Upstream regions	**C**	**C** (100%)	**C** (86%)**	**T** (15%)	**C** (100%)**	
**45,196,372**	Upstream regions	**C**	**C** (100%)	**T** (14%)**	**T** (17%)	**C** (100%)**	
**45,196,362**	Upstream regions	**A**	**G** (100%)	**G** (100%)**	**G** (89%)**	**G** (100%)**	
**45,196,341**	Upstream regions	**G**	**G** (100%)	**A** (10%)*	**A** (13%)	**G** (100%)**	
**45,196,329**	Upstream regions	**G**	**G** (100%)	**G** (67%)**	**A** (11%)	**G** (100%)**	
**45,196,303**	Upstream regions	**G**	**G** (100%)	**G** (100%)	**A** (15%)	**G** (100%)	
**45,196,267**	Upstream regions	**G**	**G** (100%)	**G** (100%)	**A** (20%)	**G** (100%)	
**45,196,210**	5′ UTR	**C**	**C** (100%)	**C** (100%)	**T** (18%)	**C** (100%)	

**45,196,198**	5′ UTR	**G**	**G** (100%)	**G** (100%)	**A** (20%)	**G** (100%)	
**45,196,182**	5′ UTR	**G**	**G** (100%)	**G** (100%)	**A** (27%)	**G** (100%)	
**45,196,181**	5′ UTR	**G**	**G** (100%)	**G** (100%)	**A** (23%)	**G** (100%)	
**45,196,180**	5′ UTR	**G**	**G** (100%)	**G** (100%)	**A** (13%)	**G** (100%)	
**45,196,139**	Exon 1	**CCC**	CC**C** (100%)	CC**C** (100%)	CC**T** (14%)	CC**C** (100%)	GGG→G_4<br>GGA→G_4
**45,196,078**	Intron 1	**C**	**C** (100%)	**T** (8%)*	**T** (11%)	**C** (100%)**	
**45,196,040**	Intron 1	**C**	**C** (100%)	**T** (8%)*	**T** (12%)	**C** (100%)**	
**45,196,034**	Intron 1	**C**	**C** (100%)	**T** (14%)	**C** (100%)	**C** (100%)**	
**45,196,032**	Intron 1	**C**	**C** (100%)	**T** (15%)	**C** (100%)	**C** (100%)**	
**45,196,012**	Intron 1	**C**	**C** (100%)	**C** (100%)	**T** (13%)	**C** (100%)**	
**45,195,994**	Intron 1	**C**	**C** (100%)	**C** (100%)	**T** (14%)	**C** (86%)**	
**45,195,979**	Intron 1	**G**	**G** (100%)	**A** (7%)*	**A** (11%)	**G** (100%)**	
**45,195,975**	Intron 1	**C**	**C** (100%)	**C** (100%)	**T** (11%)	**C** (100%)**	
**45,195,970**	Intron 1	**C**	**C** (100%)	**C** (100%)	**T** (11%)	**C** (100%)**	
**45,195,955**	Intron 1	**C**	**C** (100%)	**T** (7%)*	**T** (12%)	**C** (100%)**	
**45,195,915**	Intron 1	**C**	**C** (100%)	**T** (12%)	**C** (100%)	**C** (100%)	
**45,195,911**	Intron 1	**G**	**G** (100%)	**G** (100%)	**A** (11%)	**G** (100%)	
**45,195,873**	Intron 1	**C**	**T** (100%)	**T** (95%)	**T** (95%)	**C** (100%)	
**45,195,867**	Intron 1	**C**	**T** (3%)*	**T** (4%)*	**T** (13%)	**C** (100%)	
**45,195,845**	Intron 1	**C**	**C** (100%)	**C** (100%)	**T** (18%)	**C** (100%)	
**45,195,792**	Intron 1	**C**	**C** (100%)	**C** (100%)	**T** (17%)	**C** (100%)	
**45,195,772**	Intron 1	**C**	**C** (100%)	**C** (100%)	**T** (13%)	**C** (100%)	
**45,195,758**	Intron 1	**C**	**C** (100%)	**T** (14%)**	**T** (15%)	**C** (100%)	
**45,195,732**	Intron 1	**C**	**T** (3%)*	**T** (8%)*	**T** (15%)	**C** (100%)	
**45,195,721**	Intron 1	**C**	**C** (100%)	**C** (100%)	**T** (11%)	**C** (100%)	
**45,195,632**	Intron 1	**T**	**C** (62%)	**C** (94%)	**C** (93%)	**T** (100%)	
**45,195,499**	Intron 1	**C**	**C** (100%)	**T** (14%)	**T** (6%)*	**C** (100%)	
**45,195,498**	Intron 1	**C**	**C** (100%)	**T** (12%)	**T** (6%)*	**T** (4%)*	
**45,195,483**	Intron 1	**C**	**C** (100%)	**T** (4%)*	**T** (13%)	**C** (100%)	
**45,195,445**	Intron 1	**C**	**C** (100%)	**T** (11%)	**C** (100%)	**C** (100%)	
**45,195,343**	Intron 1	**C**	**C** (100%)	**T** (16%)	**C** (100%)	**C** (100%)	
**45,195,119**	Intron 1	**A**	**C** (100%)	**C** (95%)	**C** (100%)	**C** (96%)	
**45,195,111**	Intron 1	**A**	**C** (100%)	**C** (100%)	**C** (90%)	**C** (96%)	
**45,195,096**	Intron 1	**A**	**C** (100%)	**C** (95%)	**C** (100%)	**C** (100%)	

**45,194,940**	Intron 1	**C**	**C** (100%)	**T** (13%)	**T** (2%)*	**C** (100%)	
**45,194,932**	Intron 1	**C**	**T** (13%)	**T** (6%)*	**T** (11%)	**C** (100%)	
**45,194,913**	Intron 1	**T**	**C** (13%)	**T** (100%)	**C** (6%)*	**T** (100%)	
**45,194,871**	Intron 1	**G**	**A** (39%)	**A** (4%)*	**A** (22%)	**G** (100%)	
**45,194,844**	Intron 1	**G**	**G** (100%)	**A** (4%)*	**A** (17%)	**G** (100%)	
**45,194,843**	Intron 1	**G**	**G** (100%)	**A** (9%)*	**A** (11%)	**G** (100%)	
**45,194,723**	Intron 1	**C**	**C** (100%)	**T** (11%)	**T** (7%)*	**C** (100%)	
**45,194,713**	Intron 1	**C**	**C** (100%)	**C** (100%)	**T** (14%)	**C** (100%)	
**45,194,711**	Intron 1	**C**	**C** (100%)	**C** (100%)	**T** (24%)	**C** (100%)	
**45,194,705**	Intron 1	**C**	**C** (100%)	**C** (100%)	**T** (14%)	**C** (100%)	
**45,194,680**	Intron 1	**C**	**C** (100%)	**T** (14%)	**T** (3%)*	**C** (100%)	
**45,194,677**	Intron 1	**C**	**C** (100%)	**T** (9%)*	**T** (11%)	**C** (100%)	
**45,194,655**	Intron 1	**C**	**C** (100%)	**T** (4%)*	**T** (14%)	**C** (100%)	
**45,194,652**	Intron 1	**G**	**G** (100%)	**A** (12%)	**G** (100%)	**G** (96%)	
**45,194,635**	Exon 2	**TCC**	TC**C** (100%)	TC**T** (19%)	TC**T** (3%)*	TC**C** (100%)	AGG→R_{24}<br>AGA→R_{24}
**45,194,538**	Intron 2	**C**	**C** (100%)	**C** (100%)	**T** (12%)	**C** (100%)	
**45,194,530**	Intron 2	**A**	**A** (100%)	**T** (12%)	**A** (100%)	**A** (100%)	
**45,194,501**	Intron 2	**C**	**C** (100%)	**C** (100%)	**T** (14%)	**C** (100%)	
**45,194,484**	Intron 2	**G**	**G** (100%)	**A** (18%)	**G** (100%)	**G** (100%)	
**45,194,468**	Intron 2	**G**	**A** (4%)*	**A** (14%)	**G** (100%)	**G** (100%)	
**45,194,464**	Intron 2	**C**	**C** (98%)	**C** (100%)	**T** (18%)	**C** (100%)	
**45,194,429**	Intron 2	**G**	**G** (100%)	**A** (20%)	**A** (7%)*	**G** (100%)	
**45,194,428**	Intron 2	**G**	**G** (100%)	**A** (17%)	**G** (100%)	**G** (100%)	
**45,194,376**	Intron 2	**C**	**C** (100%)	**C** (100%)	**T** (12%)	**C** (100%)	
**45,194,255**	Intron 2	**C**	**C** (100%)	**C** (100%)	**T** (13%)	**C** (100%)	
**45,194,232**	Intron 2	**G**	**G** (100%)	**G** (100%)	**A** (12%)	**G** (100%)	
**45,194,224**	Intron 2	**C**	**C** (100%)	**T** (5%)*	**T** (11%)	**C** (100%)	
**45,194,188**	Exon 3	AA**G**	AA**A** (2%)*	AA**G** (95%)	AA**A** (11%)	AA**G** (100%)	UUC→F_{64}<br>UUU→F_{64}
**45,194,181**	Exon 3	**G**AC	**G**AC (100%)	**G**AC (100%)	**A**AC (11%)	**G**AC (100%)	CUG→L_{67}<br>UUG→L_{67}
**45,194,140**	Exon 3	AA**C**	AA**C** (100%)	AA**T** (6%)*	AA**T** (13%)	AA**C** (100%)	UUG→L_{80}<br>UUA→L_{80}
**45,194,137**	Exon 3	TG**G**	TG**G** (100%)	TG**A** (6%)*	TG**A** (11%)	TG**G** (100%)	ACC→T_{81}<br>ACU→T_{81}
**45,194,062**	3′ UTR	**G**	**G** (96%)	**A** (12%)	**A** (4%)*	**G** (100%)	
**45,194,031**	3′ UTR		**-**	**-**	**InsG** (40%)	**-**	
**45,193,981**	3′ UTR	**G**	**G** (98%)	**G** (100%)	**A** (12%)	**G** (100%)	

**45,193,964**	3′ UTR	**C**	**C** (100%)	**T** (11%)	**T** (3%)*	**C** (97%)
**45,193,851**	3′ UTR	**C**	**C** (100%)	**C** (100%)	**T** (11%)	**C** (100%)
**45,193,823**	3′ UTR	**G**	**G** (100%)	**A** (11%)	**G** (100%)	**G** (100%)
**45,193,789**	3′ UTR	**G**	**G** (100%)	**A** (5%)*	**A** (13%)	**G** (100%)
**45,193,783**	3′ UTR	**G**	**G** (100%)	**G** (100%)	**A** (13%)	**A** (4%)*
**45,193,777**	3′ UTR	**C**	**C** (100%)	**C** (100%)	**T** (13%)	**C** (100%)
**45,193,776**	3′ UTR	**C**	**C** (100%)	**T** (5%)*	**T** (19%)	**C** (100%)
**45,193,753**	3′ UTR	**C**	**T** (3%)*	**T** (11%)	**C** (100%)	**C** (100%)
**45,193,752**	3′ UTR	**C**	**C** (100%)	**T** (12%)	**T** (8%)*	**C** (100%)
**45,193,751**	3′ UTR	**C**	**C** (100%)	**T** (6%)*	**T** (17%)	**C** (100%)
**45,193,742**	3′ UTR	**G**	**G** (100%)	**A** (13%)	**G** (100%)	**G** (100%)
**45,193,728**	3′ UTR	**G**	**G** (100%)	**C** (86%)	**C** (60%)	**G** (100%)
**45,193,512**	Downstream regions	**C**	**C** (100%)	**C** (100%)	**T** (12%)	**C** (100%)
**45,193,507**	Downstream regions	**G**	**G** (98%)	**G** (100%)	**A** (12%)	**G** (100%)
**45,193,503**	Downstream regions	**G**	**A** (2%)*	**A** (6%)*	**A** (11%)	**G** (100%)
**45,193,502**	Downstream regions	**T**	**T** (100%)	**T** (100%)	**T** (100%)	**C** (65%)
**45,193,490**	Downstream regions	**C**	**C** (100%)	**T** (11%)	**T** (3%)*	**T** (6%)*
**45,193,458**	Downstream regions	**G**	**G** (100%)	**A** (6%)*	**A** (11%)	**G** (100%)
**45,193,414**	Downstream regions	**C**	**C** (100%)	**C** (100%)	**T** (15%)	**C** (100%)
**45,193,412**	Downstream regions	**C**	**C** (100%)	**T** (11%)	**C** (100%)	**C** (100%)
**45,193,410**	Downstream regions	**C**	**C** (100%)	**C** (100%)	**T** (17%)	**C** (100%)
**45,193,401**	Downstream regions	**C**	**C** (100%)	**T** (5%)*	**T** (21%)	**C** (100%)
**45,193,400**	Downstream regions	**C**	**C** (100%)	**T** (5%)*	**T** (14%)	**C** (100%)
**45,193,398**	Downstream regions	**C**	**C** (100%)	**T** (4%)*	**T** (15%)	**C** (100%)
**45,193,324**	Downstream regions	**G**	**G** (100%)	**A** (16%)	**G** (100%)	**G** (100%)
**45,193,248**	Downstream regions	**C**	**C** (100%)	**T** (11%)	**C** (100%)	**C** (100%)
**45,193,231**	Downstream regions	**C**	**C** (100%)	**C** (100%)	**T** (11%)	**T** (3%)*
**45,193,229**	Downstream regions	**C**	**T** (2%)*	**T** (5%)*	**T** (17%)	**C** (100%)
**45,193,228**	Downstream regions	**C**	**C** (100%)	**T** (14%)	**C** (100%)	**T** (3%)*
**45,193,178**	Downstream regions	**G**	**G** (100%)	**G** (100%)	**A** (12%)	**A** (5%)*
**45,193,177**	Downstream regions	**C**	**C** (100%)	**T** (100%)	**T** (46%)	**C** (100%)
**45,193,173**	Downstream regions	**G**	**A** (2%)*	**A** (13%)	**A** (4%)*	**A** (6%)*
**45,193,172**	Downstream regions	**C**	**C** (100%)	**C** (100%)	**T** (12%)	**C** (100%)

**45,193,163**	Downstream regions	**G**	**G** (100%)	**A** (11%)	**G** (100%)	**G** (100%)
**45,193,151**	Downstream regions	**G**	**G** (100%)	**A** (16%)	**A** (3%)*	**G** (100%)

n.a.: not available; the variant frequency is referred to the percentage of the highlighted base in the sequenced ancient hominine genome, with* frequency≤10% and ** counts<10. In light orange are underlined the variants fixed at 100% in modern human compared to ancient hominines.
