# Supplementary material for: A Catalog of Coding Sequence Variations in Salivary Proteins’ Genes Occurring during Recent Human Evolution
Source: Int J Mol Sci. 2023 Oct 9;24(19):15010. doi: 10.3390/ijms241915010 (PMC10573131; doi:10.3390/ijms241915010)
Supplement: Supplementary file 1 [file ijms-24-15010-s001.zip › Table S2 .pdf]

Table S2. Nucleotide Substitutions in *PRB2* gene in introns and regulatory regions

Genomic Coordinates (hg19)	Gene Region	Modern human	Altai Neanderthal (Variant Frequency)	Chagyrskaya Neanderthal (Variant Frequency)	Vindija Neanderthal (Variant Frequency)	Denisovan (Variant Frequency)	Codon → Amino acid
**11,548,942**	Upstream regions	**G**	**G** (100%)	**G** (100%)	**A** (13%)	**G** (100%)	
**11,548,866**	Upstream regions	**G**	**G** (100%)	**G** (100%)	**A** (13%)	**G** (100%)	
**11,548,855**	Upstream regions	**G**	**C** (70%)	**C** (71%)	**C** (61%)	**C** (76%)	
**11,548,817**	Upstream regions	**C**	**C** (99%)	**T** (3%)*	**T** (12%)	**C** (100%)	
**11,548,807**	Upstream regions	**G**	**G** (100%)	**A** (6%)*	**A** (20%)	**A** (2%)*	
**11,548,679**	Upstream regions	**G**	**G** (100%)	**A** (5%)*	**A** (12%)	**G** (100%)	
**11,548,613**	Upstream regions	**C**	**C** (100%)	**T** (18%)	**C** (100%)	**C** (100%)	
**11,548,610**	Upstream regions	**C**	**C** (100%)	**T** (15%)	**C** (100%)	**C** (100%)	
**11,548,583**	Upstream regions	**C**	**C** (100%)	**C** (100%)	**T** (20%)	**C** (100%)	
**11,548,522**	Upstream regions	**C**	**C** (100%)	**T** (11%)	**T** (7%)*	**C** (100%)	
**11,548,436**	Exon 1 (Signal)	**A**AC	**G**AC (69%)	**G**AC (58%)	**G**AC (60%)	**G**AC (67%)	UUG→$L_{9(sp)}$ CUG→$L_{9(sp)}$
**11,548,358**	Intron 1	**C**	**C** (100%)	**C** (100%)	**T** (15%)	**C** (100%)	
**11,548,273**	Intron 1	**C**	**T** (2%)*	**T** (3%)*	**T** (13%)	**C** (100%)	
**11,548,178**	Intron 1	**C**	**C** (100%)	**T** (11%)	**C** (100%)	**C** (100%)	
**11,548,055**	Intron 1	**C**	**C** (100%)	**T** (16%)	**T** (5%)*	**C** (100%)	
**11,548,051**	Intron 1	**C**	**C** (100%)	**T** (17%)	**T** (7%)*	**C** (100%)	
**11,547,994**	Intron 1	**C**	**C** (100%)	**T** (9%)*	**T** (11%)	**C** (100%)	
**11,547,988**	Intron 1	**C**	**C** (100%)	**T** (15%)	**T** (4%)*	**C** (100%)	
**11,547,770**	Intron 1	**C**	**C** (100%)	**C** (100%)	**T** (11%)	**C** (100%)	
**11,547,743**	Intron 1	**G**	**G** (100%)	**A** (7%)*	**A** (14%)	**G** (100%)	
**11,547,734**	Intron 1	**C**	**C** (100%)	**T** (12%)	**C** (100%)	**C** (100%)	
**11,547,684**	Intron 1	**C**	**C** (100%)	**T** (12%)	**T** (8%)*	**C** (100%)	
**11,547,682**	Intron 1	**C**	**C** (100%)	**T** (12%)	**T** (4%)*	**C** (100%)	
**11,547,651**	Intron 1	**G**	**G** (100%)	**A** (4%)*	**A** (12%)	**G** (100%)	
**11,547,642**	Intron 1	**G**	**G** (100%)	**A** (8%)*	**A** (13%)	**G** (100%)	
**11,547,639**	Intron 1	**G**	**G** (100%)	**A** (4%)*	**A** (15%)	**G** (100%)	
**11,547,624**	Intron 1	**G**	**A** (2%)*	**A** (5%)*	**A** (11%)	**G** (100%)	
**11,547,595**	Intron 1	**C**	**C** (100%)	**T** (14%)	**T** (3%)*	**C** (100%)	
**11,547,570**	Intron 1	**C**	**C** (100%)	**T** (7%)*	**T** (11%)	**C** (100%)	

**11,547,569**	Intron 1	**C**	**T** (2%)*	**T** (11%)	**T** (6%)*	**C** (100%)	
**11,547,568**	Intron 1	**C**	**C** (100%)	**T** (7%)*	**T** (16%)	**C** (100%)	
**11,547,565**	Intron 1	**C**	**C** (100%)	**T** (4%)*	**T** (12%)	**C** (100%)	
**11,547,493**	Intron 1	**C**	**A** (97%)	**A** (100%)	**A** (100%)	**A** (94%)	
**11,547,428**	Intron 2	**C**	**T** (2%)*	**T** (13%)	**T** (3%)*	**C** (100%)	
**11,547,411**	Intron 2	**G**	**G** (100%)	**A** (13%)	**A** (3%)*	**G** (100%)	
**11,547,319**	Intron 2	**C**	**C** (100%)	**T** (12%)	**T** (6%)*	**C** (100%)	
**11,547,282**	Intron 2	**G**	**A** (3%)*	**A** (6%)*	**A** (15%)	**G** (100%)	
**11,547,238**	Intron 2	**C**	**C** (100%)	**T** (3%)*	**T** (15%)	**C** (100%)	
**11,547,210**	Intron 2	**C**	**C** (100%)	**T** (12%)	**T** (8%)*	**C** (100%)	
**11,547,206**	Intron 2	**C**	**C** (100%)	**T** (4%)*	**T** (15%)	**C** (100%)	
**11,547,196**	Intron 2	**G**	**G** (100%)	**A** (100%)	**A** (13%)	**G** (100%)	
**11,547,194**	Intron 2	**G**	**G** (100%)	**A** (8%)*	**A** (20%)	**G** (100%)	
**11,547,185**	Intron 2	**C**	**C** (100%)	**C** (100%)	**T** (13%)	**C** (100%)	
**11,547,160**	Intron 2	**G**	**A** (100%)	**A** (100%)	**G** (100%)	**G** (100%)	
**11,547,133**	Intron 2	**G**	**A** (3%)*	**A** (11%)	**G** (100%)	**G** (100%)	
**11,547,130**	Intron 2	**G**	**G** (100%)	**A** (11%)	**G** (100%)	**G** (100%)	
**11,547,117**	Intron 2	**C**	**C** (100%)	**C** (100%)	**T** (11%)	**C** (97%)	
**11,547,581**	Intron 2	**C**	**C** (100%)	**C** (100%)	**T** (11%)	**C** (100%)	
**11,546,742**	Exon 3<br>(IB-1)	GT**T**	GT**C** (15%)	GT**C** (30%)	GT**C** (9%)*	GT**C** (35%)	CAA→Q_{74}<br>CAG→Q_{74}
**11,546,634**	Exon 3<br>(P-J)	CC**G**	CC**A** (3%)*	CC**A** (15%)	CC**A** (16%)	CC**A** (6%)*	GGC→G_{14}<br>GGU→G_{14}
**11,546,559**	Exon 3<br>(P-J)	GT**T**	GT**C** (8%)*	GT**C** (18%)	GT**C** (22%)	GT**C** (19%)	CAA→Q_{39}<br>CAG→Q_{39}
**11,546,553**	Exon 3<br>(P-J)	GG**G**	GG**G** (100%)	GG**G** (100%)	GG**A** (11%)	GG**G** (100%)	CCC→P_{42}<br>CCU→P_{42}
**11,546,523**	Exon 3<br>(P-J)	GG**G**	GG**G** (100%)	GG**G** (100%)	GG**A** (13%)	GG**G** (100%)	CCC→P_{52}<br>CCU→P_{52}
**11,546,373**	Exon 3<br>(IB-8a)	GT**T**	GT**C** (10%)	GT**T** (100%)	GT**C** (27%)	GT**C** (5%)*	CAA→Q_{39}<br>CAG→Q_{39}
**11,546,367**	Exon 3<br>(IB-8a)	GG**G**	GG**G** (100%)	GG**A** (11%)	GG**G** (100%)	GG**G** (100%)	CCC→P_{41}<br>CCU→P_{41}
**11,546,262**	Exon 3<br>(IB-8a)	CC**G**	CC**A** (11%)	CC**A** (8%)*	CC**A** (5%)*	CC**A** (5%)*	GGC→G_{76}<br>GGU→G_{76}
**11,546,259**	Exon 3<br>(IB-8a)	TT**G**	TT**G** (100%)	TT**A** (27%)	TT**G** (100%)	TT**G** (100%)	AAC→N_{77}<br>AAC→N_{77}
**11,546,088**	Exon 3<br>(P-F)	GG**G**	GG**G** (100%)	GG**G** (100%)	GG**A** (17%)	GG**G** (100%)	CCC→P_{10}<br>CCU→P_{10}
**11,546,013**	Exon 3<br>(P-F)	CC**G**	CC**A** (3%)*	CC**A** (15%)	CC**G** (100%)	CC**G** (100%)	GGC→G_{35}<br>GGU→G_{35}
**11,546,007**	Exon 3<br>(P-F)	TT**T**	TT**T** (100%)	TT**T** (100%)	TT**T** (100%)	TT**C** (15%)	AAA→K_{37}<br>AAG→K_{37}
**11,546,001**	Exon 3<br>(P-F)	GT**T**	GT**C** (11%)	GT**C** (100%)	GT**C** (13%)	GT**C** (11%)	CAA→Q_{39}<br>CAG→Q_{39}
**11,545,965**	Exon 3<br>(P-F)	GG**G**	GG**G** (100%)	GG**A** (14%)	GG**G** (100%)	GG**G** (100%)	CCC→P_{51}<br>CCU→P_{51}

**11,545,790**	Exon 3 (P-H)	CC**C**	CC**C** (100%)	CC**C** (100%)	CC**T** (14%)	CC**C** (100%)	GGG→G_{48}<br>GGA→G_{48}
**11,544,480**	3′ UTR	**G**	**A** (2%)*	**A** (11%)	**G** (100%)	**G** (97%)	
**11,544,389**	Downstream regions	**G**	**T** (100%)	**T** (100%)	**G** (96%)	**G** (100%)	
**11,544,353**	Downstream regions	**G**	**G** (98%)	**A** (68%)	**A** (38%)	**G** (100%)	
**11,544,289**	Downstream regions	**G**	**G** (100%)	**A** (13%)	**A** (6%)*	**G** (100%)	
**11,544,278**	Downstream regions	**G**	**G** (100%)	**A** (15%)	**A** (6%)*	**G** (100%)	
**11,544,213**	Downstream regions	**G**	**G** (100%)	**A** (19%)	**A** (7%)*	**G** (100%)	
**11,544,172**	Downstream regions	**G**	**G** (100%)	**A** (3%)*	**A** (14%)	**G** (100%)	
**11,544,043**	Downstream regions	**C**	**C** (100%)	**T** (6%)*	**T** (11%)	**C** (100%)	
**11,544,014**	Downstream regions	**G**	**A** (100%)	**A** (100%)	**A** (100%)	**A** (100%)	

n.a.: not available; the variant frequency is referred to the percentage of the highlighted base in the sequenced ancient hominine genome, with* frequency≤10%. In light orange are underlined the variants fixed at 100% in modern human compared to ancient hominines.
