# Supplementary material for: A Catalog of Coding Sequence Variations in Salivary Proteins’ Genes Occurring during Recent Human Evolution
Source: Int J Mol Sci. 2023 Oct 9;24(19):15010. doi: 10.3390/ijms241915010 (PMC10573131; doi:10.3390/ijms241915010)
Supplement: Supplementary file 1 [file ijms-24-15010-s001.zip › Table S3 .pdf]

Table S3. Nucleotide Substitutions in *PRB3* gene in introns and regulatory regions

Genomic Coordinates (hg19)	Gene Region	Modern human	Altai Neanderthal (Variant Frequency)	Chagyrskaya Neanderthal (Variant Frequency)	Vindija Neanderthal (Variant Frequency)	Denisovan (Variant Frequency)	Codon → amino acid
**11,422,912**	Upstream regions	**A**	**A** (100%)	**G** (4%)*	**A** (100%)	**G** (100%)	
**11,422,899**	Upstream regions	**G**	**G** (100%)	**A** (13%)	**A** (19%)	**G** (97%)	
**11,422,898**	Upstream regions	**G**	**G** (100%)	**A** (4%)*	**A** (16%)	**A** (3%)*	
**11,422,897**	Upstream regions	**C**	**C** (100%)	**T** (8%)*	**T** (13%)	**T** (3%)*	
**11,422,894**	Upstream regions	**G**	**G** (100%)	**A** (12%)	**G** (100%)	**G** (100%)	
**11,422,892**	Upstream regions	**C**	**C** (100%)	**C** (100%)	**T** (11%)	**C** (100%)	
**11,422,827**	Upstream regions	**C**	**C** (98%)	**C** (100%)	**T** (11%)	**C** (100%)	
**11,422,768**	Upstream regions	**C**	**C** (100%)	**C** (100%)	**T** (15%)	**C** (100%)	
**11,422,763**	Upstream regions	**G**	**G** (100%)	**A** (11%)	**A** (6%)*	**G** (100%)	
**11,422,756**	Upstream regions	**G**	**T** (95%)	**T** (96%)	**T** (100%)	**T** (94%)	
**11,422,745**	Upstream regions	**C**	**C** (100%)	**C** (100%)	**T** (11%)	**C** (100%)	
**11,422,701**	Upstream regions	**C**	**C** (98%)	**C** (100%)	**T** (15%)	**C** (100%)	
**11,422,661**	Upstream regions	**C**	**C** (100%)	**T** (4%)*	**T** (11%)	**C** (100%)	
**11,422,660**	Upstream regions	**C**	**C** (100%)	**C** (100%)	**T** (11%)	**C** (100%)	
**11,422,598**	Exon 1 (Signal)	GAT	GAC (2%)*	GAC (8%)*	GAC (18%)	GAC (11%)	CUA→$L_{9(sp)}$ CUG→$L_{9(sp)}$
**11,422,513**	Intron 1	**C**	**C** (100%)	**T** (18%)	**C** (100%)	**C** (100%)	
**11,422,471**	Intron 1	**C**	**C** (100%)	**T** (13%)	**C** (100%)	**C** (100%)	
**11,422,316**	Intron 1	**C**	**C** (100%)	**T** (12%)	**T** (6%)*	**C** (100%)	
**11,422,294**	Intron 1	**C**	**C** (100%)	**T** (17%)	**T** (7%)*	**C** (98%)	
**11,422,265**	Intron 1	**C**	**C** (100%)	**T** (12%)	**T** (11%)	**C** (100%)	
**11,422,251**	Intron 1	**C**	**C** (100%)	**T** (4%)*	**T** (15%)	**C** (100%)	
**11,422,190**	Intron 1	**A**	**C** (100%)	**C** (100%)	**C** (91%)	**C** (100%)	
**11,422,174**	Intron 1	**G**	**G** (100%)	**G** (96%)	**A** (15%)	**G** (100%)	
**11,422,040**	Intron 1	**G**	**G** (100%)	**A** (14%)	**A** (4%)*	**G** (100%)	
**11,422,039**	Intron 1	**G**	**G** (97%)	**A** (11%)	**G** (100%)	**G** (100%)	
**11,422,020**	Intron 1	**C**	**C** (100%)	**T** (17%)	**T** (7%)*	**C** (100%)	
**11,422,013**	Intron 1	**C**	**T** (2%)*	**T** (12%)	**T** (4%)*	**C** (100%)	
**11,422,003**	Intron 1	**C**	**C** (100%)	**T** (11%)	**T** (12%)	**C** (100%)	
**11,422,002**	Intron 1	**C**	**C** (100%)	**C** (100%)	**T** (12%)	**C** (100%)	

**11,421,951**	Intron 1	**C**	**C** (100%)	**T** (11%)	**C** (100%)	**C** (100%)	
**11,421,787**	Intron 1	**C**	**T** (4%)*	**C** (100%)	**T** (11%)	**C** (100%)	
**11,421,775**	Intron 1	**G**	**G** (97%)	**A** (13%)	**A** (6%)*	**G** (100%)	
**11,421,729**	Intron 1	**C**	**C** (100%)	**C** (100%)	**T** (13%)	**C** (100%)	
**11,421,721**	Intron 1	**A**	**G** (94%)	**G** (100%)	**G** (100%)	**G** (96%)	
**11,421,713**	Intron 1	**G**	**G** (100%)	**A** (17%)	**G** (100%)	**G** (100%)	
**11,421,668**	Intron 1	**C**	**C** (100%)	**C** (100%)	**T** (16%)	**C** (100%)	
**11,421,611**	Intron 1	**G**	**G** (100%)	**A** (6%)*	**A** (12%)	**G** (100%)	
**11,421,577**	Exon 2<br>(Gl-5)	AG**G**	AG**G** (100%)	AG**A** (11%)	AG**G** (100%)	AG**G** (100%)	UCC→S_{14}<br>UCU→S_{14}
**11,421,454**	Intron 1	**C**	**T** (2%)*	**T** (6%)*	**T** (16%)	**C** (100%)	
**11,421,371**	Intron 1	**C**	**C** (100%)	**C** (100%)	**A** (56%)	**A** (3%)*	
**11,421,352**	Intron 1	**C**	**C** (100%)	**C** (100%)	**T** (18%)	**C** (100%)	
**11,421,344**	Intron 1	**C**	**C** (100%)	**C** (100%)	**T** (18%)	**C** (100%)	
**11,421,329**	Intron 1	**C**	**C** (100%)	**C** (100%)	**T** (17%)	**C** (100%)	
**11,421,325**	Intron 1	**C**	**C** (100%)	**C** (100%)	**T** (14%)	**C** (100%)	
**11,421,322**	Intron 1	**C**	**T** (2%)*	**C** (100%)	**T** (13%)	**C** (100%)	
**11,421,318**	Intron 2	**G**	**G** (100%)	**A** (18%)	**A** (6%)*	**G** (100%)	
**11,421,295**	Intron 2	**C**	**C** (100%)	**T** (13%)	**T** (4%)*	**C** (100%)	
**11,421,294**	Intron 2	**C**	**C** (100%)	**T** (27%)	**C** (100%)	**C** (100%)	
**11,421,286**	Intron 2	**G**	**G** (100%)	**A** (13%)	**G** (100%)	**G** (100%)	
**11,421,265**	Intron 2	**C**	**C** (100%)	**A** (8%) *	**C** (97%)	**A** (13%)	
**11,421,102**	Intron 2	**G**	**G** (100%)	**G** (100%)	**A** (16%)	**G** (100%)	
**11,421,099**	Intron 2	**C**	**C** (100%)	**C** (100%)	**T** (13%)	**C** (100%)	
**11,420,967**	Exon 3<br>(Gl-5)	GG**T**	GG**T** (100%)	GG**C** (36%)	GG**T** (100%)	GG**T** (100%)	CCA→P_{56}<br>CCG→P_{56}
**11,420,841**	Exon 3<br>(Gl-5)	GG**T**	GG**C** (27%)	GG**T** (100%)	GG**T** (100%)	GG**T** (100%)	CCA→P_{98}<br>CCG→P_{98}
**11,420,799**	Exon 3<br>(Gl-5)	CC**T**	CC**T** (100%)	CC**T** (100%)	CC**G** (20%)	CC**T** (100%)	GGA→G_{112}<br>GGC→G_{112}
**11,420,781**	Exon 3<br>(Gl-5)	GG**G**	GG**G** (100%)	GG**A** (11%)	GG**A** (5%)*	GG**G** (100%)	CCC→P_{118}<br>CCU→P_{118}
**11,420,736**	Exon 3<br>(Gl-5)	CC**T**	CC**T** (100%)	CC**G** (27%)	CC**G** (14%)	CC**T** (100%)	GGA→G_{133}<br>GGC→G_{133}
**11,420,715**	Exon 3<br>(Gl-5)	GG**C**	GG**T** (2%)*	GG**T** (24%)	GG**T** (4%)*	GG**C** (100%)	CCG→P_{140}<br>CCA→P_{140}
**11,420,601**	Exon 3<br>(Gl-2)	AG**G**	AG**G** (100%)	AG**G** (100%)	AG**A** (11%)	AG**G** (100%)	UCC→S_{178}<br>UCU→S_{178}
**11,420,496**	Exon 3<br>(Gl-2)	GG**G**	GG**A** (12%)	GG**G** (97%)	GG**A** (3%)*	GG**A** (8%)*	CCC→ P_{213}<br>CCU→P_{213}
**11,420,458**	Intron 3	**C**	**T** (16%)	**T** (4%) *	**T** (33%)	**T** (15%)	
**11,420,454**	Intron 3	**T**	**T** (100%)	**C** (37%)	**C** (18%)	**T** (100%)	
**11,420,440**	Intron 3	**C**	**C** (100%)	**T** (4%) *	**T** (14%)	**C** (100%)	

**11,420,432**	Intron 3	**G**	**G** (100%)	**G** (100%)	**A** (11%)	**G** (100%)	
**11,420,407**	Intron 3	**C**	**C** (100%)	**T** (16%)	**C** (100%)	**C** (100%)	
**11,420,403**	Intron 3	**G**	**A** (2%)*	**A** (14%)	**G** (100%)	**G** (100%)	
**11,420,399**	Intron 3	**G**	**A** (9%)*	**A** (13%)	**G** (98%)	**G** (100%)	
**11,420,395**	Intron 3	**C**	**T** (6%)*	**C** (100%)	**T** (15%)	**C** (100%)	
**11,420,372**	Intron 3	**G**	**G** (100%)	**A** (8%)*	**A** (13%)	**G** (100%)	
**11,420,337**	Intron 3	**T**	**T** (100%)	**G** (18%)	**G** (17%)**	**T** (100%)	
**11,420,336**	Intron 3	**G**	**G** (100%)	**T** (24%)	**T** (17%)**	**G** (100%)	
**11,420,334**	Intron 3	**G**	**delG** (91%)	**delG** (77%)	**delG** (50%)**<br>**A** (50%)**	**delG** (89%)	
**11,420,306**	Exon 4<br>(Gl-2)	GG**G**	GG**G** (100%)	GG**A** (12%)	GG**A** (5%)*	GG**G** (100%)	CCC→P_{234}<br>CCU→P_{234}
**11,420,246**	Exon 4<br>(Gl-2)	GG**G**	GG**G** (100%)	GG**G** (100%)	GG**A** (14%)	GG**G** (100%)	CCC→P_{254}<br>CCU→P_{254}
**11,420,225**	Exon 4<br>(Gl-2)	GT**C**	GT**C** (100%)	GT**C** (100%)	GT**T** (12%)	GT**C** (100%)	CAG→Q_{261}<br>CAA→Q_{261}
**11,420,218**	Exon 4<br><br>(Gl-2)	**G**AC	**G**AC (100%)	**G**AC (100%)	**A**AC (15%)	**G**AC (100%)	CUG→L_{264}<br><br>UUG→L_{264}
**11,420,109**	3′UTR	**C**	**C** (100%)	**T** (4%)*	**T** (11%)	**C** (100%)	
**11,420,006**	Intron 4	**G**	**delG** (95%)	**delG** (88%)	**delG** (97%)	**delG** (100%)	
**11,419,970**	Intron 4	**G**	**G** (100%)	**A** (12%)	**A** (5%)*	**G** (100%)	
**11,419,953**	Intron 4	**C**	**C** (100%)	**C** (100%)	**T** (11%)	**C** (100%)	
**11,419,836**	Intron 4	**G**	**G** (100%)	**A** (6%)*	**A** (13%)	**G** (100%)	
**11,419,782**	Intron 4	**G**	**G** (100%)	**A** (12%)	**G** (100%)	**G** (100%)	
**11,419,678**	Intron 4	**G**	**G** (100%)	**A** (15%)	**A** (3%)*	**G** (100%)	
**11,419,650**	Intron 4	**G**	**A** (2%)*	**A** (11%)	**A** (4%)*	**G** (100%)	
**11,419,630**	Intron 4	**C**	**T** (100%)	**T** (100%)	**T** (98%)	**T** (100%)	
**11,419,573**	Intron 4	**G**	**G** (100%)	**A** (17%)	**A** (8%)*	**G** (100%)	
**11,419,509**	Intron 4	**C**	**C** (100%)	**T** (7%)*	**T** (16%)	**C** (100%)	
**11,419,513**	Intron 4	**G**	**G** (100%)	**A** (12%)	**G** (100%)	**G** (100%)	
**11,419,505**	Intron 4	**A**	**G** (100%)	**A** (100%)	**A** (100%)	**A** (100%)	
**11,419,379**	Intron 4	**C**	**C** (100%)	**C** (100%)	**T** (11%)	**C** (100%)	
**11,419,295**	Intron 4	**G**	**G** (100%)	**G** (100%)	**A** (12%)	**G** (100%)	
**11,419,132**	Intron 4	**G**	**G** (100%)	**G** (100%)	**A** (12%)	**G** (100%)	
**11,419,080**	Intron 4	**C**	**C** (100%)	**T** (17%)	**C** (100%)	**C** (100%)	
**11,419,079**	Intron 4	**C**	**C** (100%)	**T** (13%)	**T** (8%)*	**C** (100%)	
**11,419,072**	Intron 4	**C**	**T** (3%)*	**T** (10%)*	**T** (11%)	**C** (100%)	
**11,419,071**	Intron 4	**C**	**C** (100%)	**T** (14%)	**T** (5%)*	**C** (100%)	

**11,419,031**	Intron 4	**G**	**G** (100%)	**G** (100%)	**A** (14%)	**G** (100%)
**11,419,011**	Intron 4	**G**	**G** (100%)	**G** (100%)	**A** (13%)	**G** (100%)
**11,419,001**	Intron 4	**G**	**G** (100%)	**A** (14%)	**A** (4%)*	**G** (100%)
**11,418,969**	Intron 4	**C**	**C** (100%)	**T** (13%)	**C** (100%)	**C** (100%)
**11,418,876**	3′UTR	**G**	**G** (100%)	**A** (5%)*	**A** (27%)	**G** (100%)
**11,418,771**	Downstream regions	**G**	**A** (2%)*	**G** (100%)	**A** (11%)	**G** (100%)
**11,418,769**	Downstream regions	**G**	**G** (100%)	**A** (13%)	**A** (4%)*	**G** (100%)
**11,418,741**	Downstream regions	**G**	**G** (100%)	**A** (11%)	**A** (8%)*	**G** (100%)
**11,418,740**	Downstream regions	**G**	**G** (98%)	**G** (100%)	**A** (15%)	**G** (100%)
**11,418,645**	Downstream regions	**C**	**C** (100%)	**C** (100%)	**T** (14%)	**C** (100%)
**11,418,639**	Downstream regions	**G**	**G** (98%)	**A** (11%)	**G** (100%)	**G** (100%)
**11,418,631**	Downstream regions	**G**	**A** (4%)*	**A** (11%)	**A** (14%)	**G** (100%)
**11,418,630**	Downstream regions	**G**	**G** (98%)	**A** (17%)	**G** (100%)	**A** (4%)*
**11,418,627**	Downstream regions	**C**	**C** (100%)	**C** (100%)	**T** (13%)	**C** (100%)
**11,418,626**	Downstream regions	**C**	**C** (100%)	**C** (100%)	**T** (17%)	**C** (100%)
**11,418,570**	Downstream regions	**C**	**C** (100%)	**T** (11%)	**T** (11%)	**C** (100%)
**11,418,557**	Downstream regions	**G**	**A** (3%)*	**A** (3%)*	**A** (12%)	**G** (100%)
**11,418,555**	Downstream regions	**G**	**G** (98%)	**G** (100%)	**A** (12%)	**G** (100%)
**11,418,546**	Downstream regions	**G**	**A** (3%)*	**G** (100%)	**A** (15%)	**G** (100%)
**11,418,508**	Downstream regions	**C**	**C** (100%)	**C** (100%)	**T** (11%)	**C** (100%)
**11,418,414**	Downstream regions	**G**	**G** (100%)	**G** (100%)	**A** (15%)	**G** (100%)
**11,418,410**	Downstream regions	**T**	**C** (98%)	**C** (95%)	**C** (90%)	**C** (100%)
**11,418,406**	Downstream regions	**C**	**C** (100%)	**C** (100%)	**T** (11%)	**C** (100%)
**11,418,394**	Downstream regions	**C**	**T** (6%)*	**C** (100%)	**T** (17%)	**C** (100%)
**11,418,393**	Downstream regions	**C**	**C** (100%)	**T** (13%)	**T** (6%)*	**C** (100%)
**11,418,386**	Downstream regions	**G**	**G** (100%)	**G** (100%)	**A** (14%)	**G** (100%)
**11,418,372**	Downstream regions	**G**	**G** (100%)	**A** (18%)	**A** (7%)*	**G** (100%)
**11,418,368**	Downstream regions	**G**	**G** (100%)	**G** (100%)	**A** (14%)	**G** (100%)

n.a.: not available; the variant frequency is referred to the percentage of the highlighted base in the sequenced ancient hominine genome, with* frequency≤10% and ** counts<10. In light orange are underlined the variants fixed at 100% in modern human compared to ancient hominines.
