# Supplementary material for: A Catalog of Coding Sequence Variations in Salivary Proteins’ Genes Occurring during Recent Human Evolution
Source: Int J Mol Sci. 2023 Oct 9;24(19):15010. doi: 10.3390/ijms241915010 (PMC10573131; doi:10.3390/ijms241915010)
Supplement: Supplementary file 1 [file ijms-24-15010-s001.zip › Table S4 .pdf]

Table S4. Nucleotide Substitutions in *PRB4* gene in introns and regulatory regions

Genomic Coordinates (hg19)	Gene Region	Modern human	Altai Neanderthal (Variant Frequency)	Chagyrskaya Neanderthal (Variant Frequency)	Vindija Neanderthal (Variant Frequency)	Denisovan (Variant Frequency)	Codon → amino acid
**11,463,890**	Upstream regions	**C**	**C** (100%)	**T** (13%)	**T** (9%)*	**C** (100%)	
**11,463,868**	Upstream regions	**G**	**T** (100%)	**T** (100%)	**T** (100%)	**T** (100%)	
**11,463,814**	Upstream regions	**A**	**T** (97%)	**T** (100%)	**T** (100%)	**T** (100%)	
**11,463,813**	Upstream regions	**G**	**A** (100%)	**A** (100%)	**A** (100%)	**A** (97%)	
**11,463,806**	Upstream regions	**G**	**G** (100%)	**G** (100%)	**A** (13%)	**G** (100%)	
**11,463,780**	Upstream regions	**G**	**G** (100%)	**A** (4%)*	**A** (13%)	**G** (100%)	
**11,463,693**	Upstream regions	**C**	**C** (100%)	**T** (3%)*	**T** (13%)	**C** (100%)	
**11,463,675**	Upstream regions	**C**	**T** (3%)*	**C** (100%)	**T** (11%)	**C** (100%)	
**11,463,623**	Upstream regions	**G**	**G** (100%)	**A** (4%)*	**A** (11%)	**G** (100%)	
**11,463,613**	Upstream regions	**T**	**T** (100%)	**T** (100%)	**T** (100%)	**A** (100%)	
**11,463,513**	Upstream regions	**T**	**T** (100%)	**T** (100%)	**T** (100%)	**C** (56%)	
**11,463,491**	Upstream regions	**C**	**C** (100%)	**T** (5%)*	**T** (11%)	**C** (100%)	
**11,463,480**	Upstream regions	**G**	**T** (3%)*	**T** (4%)*	**T** (11%)<br>**A** (11%)	**T** (4%)*	
**11,463,443**	Upstream regions	**C**	**C** (100%)	**C** (100%)	**T** (11%)	**C** (100%)	
**11,463,391**	Upstream regions	**C**	**C** (100%)	**T** (13%)	**C** (100%)	**C** (100%)	
**11,463,375**	Upstream regions	**G**	**G** (100%)	**G** (100%)	**A** (21%)	**A** (5%)*	
**11,463,335**	5′UTR	**C**	**T** (100%)	**T** (100%)	**T** (100%)	**T** (100%)	
**11,463,302**	Exon 1 (Signal)	<u>G</u>AC	<u>G</u>AC (100%)	<u>A</u>AC (13%)	<u>A</u>AC (6%)*	<u>G</u>AC (100%)	CUG→L_{10}(sp)<br>UUG→L_{10}(sp)
**11,463,193**	Intron 1	**C**	**C** (100%)	**T** (4%)*	**T** (12%)	**C** (100%)	
**11,463,190**	Intron 1	**C**	**C** (100%)	**C** (100%)	**T** (11%)	**C** (100%)	
**11,463,130**	Intron 1	**G**	**G** (100%)	**A** (24%)	**A** (8%)*	**G** (100%)	
**11,463,113**	Intron 1	**C**	**C** (100%)	**C** (100%)	**T** (13%)	**T** (2%)*	
**11,463,051**	Intron 1	**T**	**A** (100%)	**T** (100%)	**T** (100%)	**T** (100%)	
**11,463,030**	Intron 1	**C**	**C** (100%)	**C** (100%)	**T** (11%)	**C** (97%)	
**11,463,028**	Intron 1	**C**	**C** (100%)	**C** (100%)	**T** (16%)	**C** (100%)	
**11,463,000**	Intron 1	**C**	**C** (100%)	**C** (100%)	**T** (11%)	**C** (100%)	
**11,462,979**	Intron 1	**C**	**A** (98%)	**A** (96%)	**A** (96%)	**A** (100%)	
**11,462,919**	Intron 1	**G**	**G** (100%)	**G** (100%)	**A** (11%)	**G** (100%)	
**11,462,900**	Intron 1	**C**	**C** (100%)	**T** (9%)*	**T** (11%)	**C** (100%)	

**11,462,894**	Intron 1	**C**	**C** (98%)	**C** (100%)	**T** (11%)	**C** (100%)	
**11,462,827**	Intron 1	**G**	**G** (98%)	**G** (100%)	**A** (20%)	**G** (100%)	
**11,462,558**	Intron 1	**C**	**C** (100%)	**T** (14%)	**C** (100%)	**C** (100%)	
**11,462,521**	Intron 1	**C**	**C** (100%)	**C** (100%)	**T** (21%)	**C** (100%)	
**11,462,507**	Intron 1	**G**	**G** (100%)	**A** (16%)	**A** (7%)*	**G** (100%)	
**11,462,505**	Intron 1	**G**	**G** (100%)	**A** (11%)	**G** (100%)	**G** (100%)	
**11,462,504**	Intron 1	**G**	**G** (100%)	**A** (15%)	**G** (94%)	**G** (100%)	
**11,462,487**	Intron 1	**G**	**G** (100%)	**A** (14%)	**A** (7%)*	**G** (100%)	
**11,462,486**	Intron 1	**G**	**G** (100%)	**A** (14%)	**A** (6%)*	**G** (100%)	
**11,462,477**	Intron 1	**G**	**G** (100%)	**A** (12%)	**G** (100%)	**G** (100%)	
**11,462,432**	Intron 1	**C**	**C** (100%)	**T** (4%)*	**T** (11%)	**C** (100%)	
**11,462,430**	Intron 1	**C**	**C** (100%)	**T** (3%)*	**T** (12%)	**C** (100%)	
**11,462,392**	Intron 1	**C**	**T** (2%)*	**C** (100%)	**T** (11%)	**C** (100%)	
**11,462,317**	Exon 2 (PGA)	GA**G**	GA**G** (100%)	GA**A** (17%)	GA**G** (100%)	GA**T** (4%)*	CUC→L_{13} CUU→L_{13}
**11,462,269**	Intron 2	**G**	**G** (100%)	**A** (14%)	**A** (4%)*	**G** (100%)	
**11,462,185**	Intron 2	**G**	**G** (100%)	**A** (11%)	**G** (100%)	**G** (100%)	
**11,462,175**	Intron 2	**A**	**A** (99%)	**A** (100%)	**A** (100%)	**G** (100%)	
**11,462,142**	Intron 2	**G**	**G** (98%)	**G** (100%)	**A** (15%)	**G** (100%)	
**11,462,082**	Intron 2	**C**	**C** (100%)	**C** (100%)	**T** (19%)	**C** (100%)	
**11,462,081**	Intron 2	**C**	**C** (100%)	**C** (100%)	**T** (27%)	**C** (100%)	
**11,462,076**	Intron 2	**G**	**G** (100%)	**A** (13%)	**G** (100%)	**G** (100%)	
**11,462,027**	Intron 2	**C**	**C** (100%)	**T** (14%)	**C** (100%)	**C** (100%)	
**11,462,002**	Intron 2	**C**	**A** (93%)	**A** (95%)	**A** (80%)	**A** (95%)	
**11,461,980**	Intron 2	**G**	**A** (2%)*	**A** (13%)	**A** (3%)*	**G** (100%)	
**11,461,978**	Intron 2	**G**	**G** (100%)	**A** (12%)	**A** (13%)	**G** (100%)	
**11,461,860**	Intron 2	**A**	**G** (14%)	**G** (13%)	**G** (15%)	**G** (33%)	
**11,461,836**	Intron 2	**T**	**C** (100%)	**C** (100%)	**C** (100%)	**C** (100%)	
**11,461,470**	Exon 3 (PGA)	CC**A**	CC**T** (5%)*	CC**T** (15%)	CC**T** (17%)	CC**G** (11%)	GGU→G_{133} GGA→G_{133}
**11,461,452**	Exon 3 (PGA)	GG**G**	GG**G** (100%)	GG**A** (11%)	GG**A** (4%)*	GG**G** (100%)	CCC→P_{139} CCU→P_{139}
**11,461,155**	3′UTR	**C**	**C** (100%)	**T** (16%)	**C** (100%)	**C** (100%)	
**11,461,009**	Intron 4	**C**	**C** (100%)	**T** (4%)*	**T** (21%)	**C** (100%)	
**11,461,004**	Intron 4	**C**	**C** (100%)	**T** (6%)*	**T** (11%)	**C** (100%)	
**11,461,002**	Intron 4	**C**	**C** (100%)	**C** (96%)	**T** (16%)	**C** (100%)	
**11,460,987**	Intron 4	**T**	**T** (100%)	**A** (35%)	**T** (100%)	**T** (100%)	

**11,460,919**	Intron 4	**C**	**C** (100%)	**T** (14%)	**C** (100%)	**C** (100%)
**11,460,891**	Intron 4	**G**	**G** (100%)	**G** (100%)	**T** (11%)	**G** (100%)
**11,460,868**	Intron 4	**G**	**G** (100%)	**A** (4%)*	**A** (14%)	**G** (100%)
**11,460,860**	Intron 4	**C**	**C** (100%)	**C** (100%)	**T** (12%)	**C** (100%)
**11,460,821**	Intron 4	**G**	**G** (99%)	**A** (13%)	**A** (6%)*	**G** (100%)
**11,460,819**	Intron 4	**G**	**A** (3%)*	**A** (8%)*	**A** (11%)	**G** (100%)
**11,460,683**	Intron 4	**G**	**G** (100%)	**A** (19%)	**G** (100%)	**G** (100%)
**11,460,647**	Intron 4	**C**	**C** (100%)	**T** (15%)	**T** (13%)	**C** (100%)
**11,460,636**	Intron 4	**G**	**G** (98%)	**A** (13%)	**A** (6%)*	**G** (100%)
**11,460,532**	Intron 4	**G**	**G** (100%)	**A** (8%)*	**A** (22%)	**G** (100%)
**11,460,531**	Intron 4	**G**	**G** (100%)	**G** (100%)	**A** (13%)	**G** (100%)
**11,460,498**	Intron 4	**C**	**C** (99%)	**T** (15%)	**T** (12%)	**C** (100%)
**11,460,445**	Intron 4	**C**	**C** (100%)	**T** (5%)*	**T** (13%)	**C** (100%)
**11,460,394**	Intron 4	**G**	**G** (100%)	**A** (11%)	**A** (10%)*	**G** (100%)
**11,460,245**	Intron 4	**C**	**C** (100%)	**T** (14%)	**C** (100%)	**C** (100%)
**11,460,244**	Intron 4	**G**	**G** (98%)	**A** (5%)*	**A** (13%)	**G** (100%)
**11,460,231**	Intron 4	**C**	**T** (98%)	**T** (100%)	**T** (100%)	**T** (100%)
**11,460,224**	Intron 4	**G**	**A** (11%)	**G** (98%)	**G** (100%)	**G** (100%)
**11,460,070**	3′UTR	**C**	**C** (98%)	**C** (100%)	**T** (13%)	**C** (100%)
**11,460,054**	3′UTR	**C**	**C** (100%)	**T** (9%)*	**T** (17%)	**C** (100%)
**11,459,932**	Downstream regions	**G**	**G** (100%)	**A** (14%)	**A** (5%)*	**G** (100%)
**11,459,865**	Downstream regions	**T**	**C** (99%)	**C** (97%)	**C** (93%)	**C** (100%)
**11,459,793**	Downstream regions	**G**	**A** (2%)*	**A** (5%)*	**A** (11%)	**G** (100%)
**11,459,750**	Downstream regions	**G**	**G** (98%)	**A** (12%)	**G** (100%)	**G** (100%)
**11,459,722**	Downstream regions	**G**	**G** (100%)	**G** (100%)	**A** (11%)	**A** (4%)*
**11,459,713**	Downstream regions	**G**	**G** (100%)	**A** (21%)	**A** (6%)*	**G** (100%)
**11,459,706**	Downstream regions	**G**	**G** (100%)	**A** (4%)*	**A** (11%)	**G** (100%)
**11,459,689**	Downstream regions	**C**	**C** (100%)	**T** (9%)*	**T** (18%)	**C** (97%)
**11,459,685**	Downstream regions	**G**	**G** (98%)	**G** (100%)	**A** (14%)	**G** (100%)
**11,459,684**	Downstream regions	**G**	**G** (100%)	**A** (3%)*	**A** (14%)	**G** (100%)
**11,459,683**	Downstream regions	**G**	**A** (2%)*	**G** (100%)	**A** (13%)	**G** (100%)
**11,459,645**	Downstream regions	**C**	**C** (100%)	**C** (100%)	**T** (11%)	**C** (100%)
**11,459,633**	Downstream regions	**G**	**G** (100%)	**A** (11%)	**A** (5%)*	**G** (100%)

**11,459,625**	Downstream regions	**C**	**C** (100%)	**C** (100%)	**T** (12%)	**C** (100%)
**11,459,615**	Downstream regions	**C**	**C** (100%)	**C** (100%)	**T** (14%)	**C** (100%)
**11,459,596**	Downstream regions	**C**	**C** (100%)	**C** (100%)	**T** (11%)	**C** (100%)
**11,459,588**	Downstream regions	**C**	**G** (18%)	**C** (100%)	**C** (100%)	**G** (5%)*
**11,459,568**	Downstream regions	**C**	**A** (25%)	**C** (100%)	**A** (4%)*	**A** (6%)*
**11,459,566**	Downstream regions	**C**	**C** (100%)	**C** (100%)	**T** (11%)	**C** (100%)
**11,459,565**	Downstream regions	**A**	**G** (20%)	**A** (100%)	**A** (3%)*	**A** (100%)
**11,459,556**	Downstream regions	**G**	**G** (100%)	**A** (38%)	**A** (7%)*	**G** (100%)
**11,459,551**	Downstream regions	**T**	**G** (22%)	**G** (6%)*	**T** (100%)	**G** (3%)*
**11,459,531**	Downstream regions	**G**	**G** (100%)	**G** (100%)	**A** (13%)	**G** (97%)

n.a.: not available; the variant frequency is referred to the percentage of the highlighted base in the sequenced ancient hominine genome, with* frequency≤10%. In light orange are underlined the variants fixed at 100% in modern human compared to ancient hominines.
