# Supplementary material for: A Catalog of Coding Sequence Variations in Salivary Proteins’ Genes Occurring during Recent Human Evolution
Source: Int J Mol Sci. 2023 Oct 9;24(19):15010. doi: 10.3390/ijms241915010 (PMC10573131; doi:10.3390/ijms241915010)
Supplement: Supplementary file 1 [file ijms-24-15010-s001.zip › Table S5.pdf]

Table S5. Nucleotide Substitutions in *PRH2* gene in introns and regulatory regions

Genomic Coordinates (hg19)	Gene Region	Modern human	Altai Neanderthal (Variant Frequency)	Chagyrskaya Neanderthal (Variant Frequency)	Vindija Neanderthal (Variant Frequency)	Denisovan (Variant Frequency)	Codon → amino acid
**11,081,651**	Upstream regions	**C**	**C** (100%)	**C** (100%)	**T** (16%)	**C** (100%)	
**11,081,653**	Upstream regions	**C**	**C** (100%)	**T** (4%)*	**T** (15%)	**C** (100%)	
**11,081,672**	Upstream regions	**C**	**A** (4%)*	**T** (11%)	**C** (100%)	**C** (100%)	
**11,081,681**	Upstream regions	**C**	**C** (100%)	**T** (15%)	**C** (100%)	**C** (100%)	
**11,081,759**	Upstream regions	**G**	**G** (100%)	**A** (15%)	**A** (13%)**	**G** (100%)	
**11,081,788**	Upstream regions	**G**	**A** (4%)*	**A** (100%)	**G** (100%)	**G** (100%)	
**11,081,803**	Upstream regions	**G**	**G** (100%)	**G** (100%)	**A** (17%)	**G** (96%)	
**11,081,840**	5′UTR	**A**	**G** (98%)	**A** (100%)	**A** (100%)	**A** (100%)	
**11,081,842**	5′UTR	**A**	**A** (100%)	**A** (100%)	**A** (100%)	**T** (100%)	
**11,081,856**	5′UTR	**C**	**C** (100%)	**C** (100%)	**T** (13%)	**C** (100%)	
**11,081,997**	Intron 1	**G**	**G** (100%)	**A** (4%)*	**A** (11%)	**G** (100%)	
**11,082,013**	Intron 1	**G**	**G** (100%)	**A** (14%)	**G** (100%)	**G** (100%)	
**11,082,167**	Intron 1	**G**	**G** (100%)	**A** (11%)	**A** (6%)*	**G** (100%)	
**11,082,201**	Intron 1	**G**	**G** (100%)	**A** (3%)*	**A** (11%)	**G** (100%)	
**11,082,210**	Intron 1	**A**	**A** (100%)	**G** (89%)	**G** (100%)	**A** (100%)	
**11,082,254**	Intron 1	**C**	**C** (100%)	**A** (100%)	**A** (100%)	**C** (100%)	
**11,082,310**	Intron 1	**T**	**T** (100%)	**C** (100%)	**C** (97%)	**T** (100%)	
**11,082,355**	Intron 1	**G**	**G** (97%)	**A** (15%)	**A** (3%)*	**G** (100%)	
**11,082,462**	Intron 1	**C**	**T** (100%)	**T** (4%)*	**T** (7%)*	**T** (3%)*	
**11,082,479**	Intron 1	**G**	**G** (100%)	**G** (100%)	**A** (14%)	**G** (100%)	
**11,082,514**	Intron 1	**G**	**A** (3%)*	**G** (100%)	**A** (11%)	**G** (100%)	
**11,082,598**	Intron 1	**C**	**C** (100%)	**T** (12%)	**T** (10%)*	**C** (100%)	
**11,082,599**	Intron 1	**C**	**C** (100%)	**T** (16%)	**C** (97%)	**C** (100%)	
**11,082,615**	Intron 1	**A**	**A** (100%)	**A** (100%)	**A** (100%)	**G** (97%)	
**11,082,650**	Intron 1	**G**	**G** (100%)	**A** (11%)	**A** (3%)*	**G** (100%)	
**11,082,651**	Intron 1	**G**	**G** (100%)	**A** (11%)	**G** (100%)	**G** (100%)	
**11,082,652**	Intron 1	**C**	**C** (100%)	**T** (3%)*	**T** (22%)	**C** (100%)	
**11,082,653**	Intron 1	**C**	**C** (100%)	**T** (6%)*	**T** (11%)	**C** (100%)	
**11,082,654**	Intron 1	**C**	**C** (100%)	**T** (3%)*	**T** (12%)	**C** (100%)	
**11,082,661**	Intron 1	**G**	**G** (100%)	**A** (3%)*	**A** (13%)	**G** (100%)	

**11,082,689**	Intron 1	**G**	**G** (100%)	**G** (100%)	**A** (16%)	**G** (100%)	
**11,082,705**	Intron 1	**C**	**C** (100%)	**T** (12%)	**T** (4%)*	**C** (100%)	
**11,082,740**	Intron 1	**G**	**A** (2%)*	**A** (3%)*	**A** (16%)	**G** (100%)	
**11,082,774**	Intron 1	**G**	**G** (100%)	**G** (94%)	**A** (11%)	**G** (100%)	
**11,082,775**	Intron 1	**G**	**G** (100%)	**A** (7%)*	**A** (16%)	**G** (100%)	
**11,082,787**	Intron 1	**G**	**G** (100%)	**G** (100%)	**A** (17%)	**G** (100%)	
**11,082,831**	Intron 1	**C**	**C** (100%)	**A** (100%)	**A** (100%)	**C** (100%)	
**11,082,869**	Exon 2 (PRP-1)	GA**T**	GA**C** (100%)	GA**T** (100%)	GA**T** (100%)	GA**T** (100%)	GAU→D_{6}<br>GAC→D_{6}
**11,082,884**	Exon 2 (PRP-1)	GA**C**	GA**C** (100%)	GA**T** (11%)	GA**T** (8%)*	GA**C** (100%)	GAC→D_{11}<br>GAU→D_{11}
**11,082,932**	Intron 2	**C**	**C** (100%)	**T** (3%)*	**T** (11%)	**C** (100%)	
**11,082,971**	Intron 2	**G**	**G** (100%)	**A** (11%)	**A** (3%)*	**G** (100%)	
**11,083,014**	Intron 2	**C**	**C** (97%)	**T** (14%)	**C** (100%)	**C** (100%)	
**11,083,015**	Intron 2	**C**	**C** (100%)	**T** (14%)	**C** (100%)	**C** (97%)	
**11,083,054**	Intron 2	**G**	**G** (100%)	**G** (100%)	**A** (13%)	**G** (100%)	
**11,083,088**	Intron 2	**C**	**C** (100%)	**T** (6%)*	**T** (12%)	**C** (100%)	
**11,083,089**	Intron 2	**C**	**C** (98%)	**C** (100%)	**T** (11%)	**C** (100%)	
**11,083,090**	Intron 2	**C**	**C** (100%)	**C** (100%)	**T** (13%)	**C** (100%)	
**11,083,134**	Intron 2	**G**	**G** (100%)	**T** (93%)	**T** (83%)	**C** (79%)	
**11,083,177**	Intron 2	**C**	**C** (100%)	**T** (6%)*	**T** (13%)	**C** (100%)	
**11,083,180**	Intron 2	**C**	**C** (100%)	**C** (100%)	**T** (12%)	**C** (100%)	
**11,083,313**	Exon 3 (PRP-1)	TT**G**	TT**G** (98%)	TT**A** (12%)	TT**A** (5%)*	TT**G** (94%)	UUG→L_{35}<br>UUA→L_{35}
**11,083,445**	Exon 3 (PRP-1)	CC**C**	CC**C** (100%)	CC**T** (11%)	CC**C** (100%)	CC**C** (100%)	CCC→P_{79}<br>CCU→P_{79}
**11,083,502**	Exon 3 (PRP-1)	CA**G**	CA**G** (98%)	CA**G** (100%)	CA**A** (20%)	CA**G** (100%)	CAG→Q_{98}<br>CAA→Q_{98}
**11,083,613**	Exon 3 (PRP-1)	CC**C**	CC**C** (100%)	CC**C** (100%)	CC**C** (100%)	CC**T** (60%)	CCC→P_{135}<br>CCU→P_{135}
**11,083,622**	Exon 3 (PRP-1)	GG**C**	GG**C** (100%)	GG**C** (100%)	GG**T** (11%)	GG**C** (100%)	GGC→G_{138}<br>GGU→G_{138}
**11,083,671**	3′UTR	**C**	**C** (100%)	**C** (100%)	**T** (12%)	**C** (100%)	
**11,083,677**	3′UTR	**T**	**C** (100%)	**C** (100%)	**C** (85%)	**C** (93%)	
**11,083,679**	3′UTR	**G**	**G** (100%)	**G** (100%)	**A** (12%)	**G** (100%)	
**11,083,689**	Intron 3	**C**	**C** (100%)	**T** (14%)	**T** (4%)*	**C** (100%)	
**11,083,700**	Intron 3	**C**	**C** (100%)	**C** (100%)	**T** (3%)*	**T** (86%)	
**11,083,709**	Intron 3	**G**	**G** (98%)	**A** (13%)	**A** (3%)*	**G** (100%)	
**11,083,819**	Intron 3	**T**	**T** (100%)	**T** (100%)	**T** (100%)	**C** (39%)	
**11,083,828**	Intron 3	**G**	**G** (100%)	**A** (11%)	**A** (5%)*	**G** (100%)	
**11,083,870**	Intron 3	**C**	**C** (100%)	**C** (100%)	**T** (12%)	**C** (100%)	

**11,083,899**	Intron 3	**C**	**C** (100%)	**T** (4%)*	**T** (13%)	**C** (100%)
**11,083,900**	Intron 3	**C**	**C** (100%)	**T** (3%)*	**T** (13%)	**C** (100%)
**11,083,951**	Intron 3	**G**	**G** (100%)	**A** (13%)	**G** (100%)	**G** (100%)
**11,083,962**	Intron 3	**C**	**C** (100%)	**C** (100%)	**T** (12%)	**C** (100%)
**11,083,967**	Intron 3	**C**	**C** (100%)	**T** (5%)*	**T** (11%)	**C** (100%)
**11,084,096**	Intron 3	**C**	**C** (98%)	**T** (13%)	**T** (4%)*	**C** (100%)
**11,084,126**	Intron 3	**C**	**C** (100%)	**T** (12%)	**C** (100%)	**C** (100%)
**11,084,167**	Intron 3	**C**	**C** (100%)	**T** (11%)	**C** (100%)	**C** (100%)
**11,084,228**	Intron 3	**C**	**C** (100%)	**T** (13%)	**T** (6%)*	**C** (100%)
**11,084,229**	Intron 3	**C**	**C** (100%)	**T** (13%)	**T** (5%)*	**C** (100%)
**11,084,419**	Intron 3	**T**	**InsT** (2%)*<br>**delT** (7%)*	**InsT** (16%)	**InsT** (8%)*<br>**delT** (6%)*	**delT** (14%)
**11,084,464**	Intron 3	**A**	**G** (96%)	**G** (96%)	**G** (98%)	**G** (100%)
**11,084,646**	Intron 3	**C**	**C** (100%)	**C** (100%)	**T** (16%)	**C** (100%)
**11,084,671**	Intron 3	**G**	**G** (100%)	**A** (3%)*	**A** (3%)*	**G** (100%)
**11,084,698**	Intron 3	**G**	**G** (100%)	**A** (7%)*	**A** (11%)	**G** (100%)
**11,084,701**	Intron 3	**G**	**G** (100%)	**A** (3%)*	**A** (18%)	**G** (100%)
**11,084,702**	Intron 3	**G**	**G** (100%)	**G** (100%)	**A** (13%)	**G** (100%)
**11,084,711**	Intron 3	**G**	**A** (2%)*	**A** (13%)	**G** (100%)	**G** (100%)
**11,084,750**	Intron 3	**C**	**C** (98%)	**C** (95%)	**A** (17%)	**C** (97%)
**11,084,798**	Intron 3	**G**	**G** (100%)	**G** (100%)	**A** (13%)	**G** (100%)
**11,084,799**	Intron 3	**C**	**C** (100%)	**T** (4%)*	**T** (13%)	**C** (100%)
**11,084,807**	Intron 3	**C**	**C** (100%)	**T** (11%)	**C** (100%)	**C** (100%)
**11,084,825**	3′UTR	**G**	**G** (100%)	**A** (4%)*	**A** (11%)	**G** (100%)
**11,084,960**	3′UTR	**G**	**G** (100%)	**G** (100%)	**A** (13%)	**G** (100%)
**11,084,999**	3′UTR	**G**	**G** (100%)	**A** (3%)*	**A** (14%)	**G** (100%)
**11,085,183**	3′UTR	**G**	**G** (100%)	**A** (100%)	**G** (100%)	**G** (100%)
**11,085,272**	3′UTR	**C**	**C** (100%)	**C** (100%)	**T** (11%)	**C** (100%)
**11,085,302**	3′UTR	**C**	**T** (2%)*	**C** (100%)	**T** (13%)	**C** (100%)
**11,085,303**	3′UTR	**C**	**T** (2%)*	**C** (100%)	**T** (13%)	**C** (100%)
**11,085,331**	3′UTR	**G**	**G** (100%)	**G** (100%)	**A** (11%)	**G** (97%)
**11,085,392**	3′UTR	**C**	**C** (100%)	**T** (7%)*	**T** (11%)	**C** (100%)
**11,085,499**	3′UTR	**G**	**G** (100%)	**G** (97%)	**A** (15%)	**G** (100%)
**11,085,555**	3′UTR	**C**	**C** (98%)	**T** (3%)*	**T** (17%)	**C** (100%)
**11,085,557**	3′UTR	**C**	**T** (100%)	**T** (2%)*	**T** (13%)	**T** (100%)

**11,085,704**	3′UTR	**G**	**G** (100%)	**A** (17%)	**A** (6%)*	**G** (97%)
**11,085,705**	3′UTR	**G**	**G** (100%)	**A** (13%)	**G** (100%)	**G** (100%)
**11,085,739**	3′UTR	**T**	**T** (100%)	**C** (100%)	**C** (100%)	**T** (100%)
**11,085,747**	3′UTR	**G**	**A** (1%)*	**G** (100%)	**A** (13%)	**G** (100%)
**11,085,925**	3′UTR	**C**	**T** (100%)	**T** (6%)*	**T** (13%)	**T** (100%)
**11,085,964**	3′UTR	**C**	**T** (100%)	**T** (3%)*	**T** (13%)	**T** (100%)
**11,086,132**	3′UTR	**G**	**A** (2%)*	**A** (13%)	**A** (3%)*	**G** (100%)
**11,086,145**	3′UTR	**C**	**T** (100%)	**T** (97%)	**T** (97%)	**T** (100%)
**11,086,323**	3′UTR	**G**	**A** (3%)*	**G** (100%)	**A** (14%)	**G** (100%)
**11,086,491**	3′UTR	**G**	**G** (100%)	**G** (100%)	**A** (13%)	**G** (100%)
**11,086,570**	3′UTR	**G**	**G** (100%)	**C** (100%)	**C** (100%)	**G** (100%)
**11,086,646**	3′UTR	**C**	**C** (100%)	**T** (3%)*	**T** (11%)	**T** (3%)*
**11,086,828**	3′UTR	**C**	**C** (100%)	**T** (14%)	**T** (4%)*	**C** (100%)
**11,086,844**	3′UTR	**C**	**C** (100%)	**T** (11%)	**C** (100%)	**C** (100%)
**11,086,849**	3′UTR	**C**	**C** (100%)	**T** (11%)	**T** (3%)*	**T** (5%)*
**11,086,882**	3′UTR	**C**	**C** (100%)	**T** (11%)	**T** (5%)*	**C** (100%)
**11,086,908**	3′UTR	**C**	**C** (100%)	**T** (16%)	**T** (8%)*	**C** (100%)
**11,086,910**	3′UTR	**C**	**C** (100%)	**T** (4%)*	**T** (11%)	**C** (100%)
**11,086,966**	3′UTR	**G**	**G** (100%)	**G** (100%)	**A** (13%)	**G** (100%)
**11,086,987**	3′UTR	**C**	**G** (100%)	**T** (4%)*	**C** (100%)	**C** (100%)
**11,087,001**	3′UTR	**C**	**C** (100%)	**T** (12%)	**T** (12%)	**C** (100%)
**11,087,003**	3′UTR	**C**	**C** (100%)	**C** (100%)	**T** (11%)	**C** (100%)
**11,087,039**	3′UTR	**C**	**C** (100%)	**T** (13%)	**T** (3%)*	**C** (100%)
**11,087,080**	3′UTR	**G**	**G** (100%)	**A** (15%)	**A** (8%)*	**G** (100%)
**11,087,107**	3′UTR	**G**	**G** (100%)	**G** (100%)	**A** (13%)	**G** (100%)
**11,087,118**	3′UTR	**G**	**T** (3%)*	**A** (11%)	**A** (7%)*	**G** (100%)
**11,087,172**	3′UTR	**C**	**A** (2%)*	**T** (16%)	**C** (100%)	**A** (3%)*
**11,087,173**	3′UTR	**C**	**T** (100%)	**T** (11%)	**T** (16%)	**T** (100%)
**11,087,199**	3′UTR	**G**	**G** (100%)	**G** (100%)	**A** (13%)	**G** (100%)
**11,087,215**	3′UTR	**C**	**C** (99%)	**T** (11%)	**C** (100%)	**C** (100%)
**11,087,216**	3′UTR	**C**	**C** (100%)	**T** (17%)	**T** (5%)*	**C** (97%)
**11,087,220**	3′UTR	**A**	**delA** (91%)	**T** (29%)<br>**delA** (45%)	**delA** (82%)	**delA** (82%)
**11,087,260**	3′UTR	**T**	**T** (100%)	**C** (100%)	**C** (100%)	**T** (100%)
**11,087,348**	3′UTR	**G**	**G** (100%)	**C** (100%)	**C** (88%)<br>**T** (12%)	**G** (100%)

**11,087,467**	Downstream regions	**C**	**C** (100%)	**T** (3%)*	**T** (11%)	**C** (100%)
**11,087,570**	Downstream regions	**G**	**G** (100%)	**A** (14%)	**G** (100%)	**G** (100%)
**11,087,615**	Downstream regions	**G**	**G** (100%)	**A** (12%)	**G** (100%)	**G** (100%)
**11,087,625**	Downstream regions	**G**	**G** (100%)	**A** (13%)	**A** (8%)*	**G** (100%)
**11,087,626**	Downstream regions	**G**	**G** (98%)	**A** (12%)	**A** (7%)*	**G** (100%)
**11,087,765**	Downstream regions	**C**	**T** (2%)*	**C** (100%)	**T** (11%)	**C** (100%)
**11,087,766**	Downstream regions	**G**	**A** (2%)*	**G** (100%)	**A** (11%)	**G** (100%)
**11,087,811**	Downstream regions	**G**	**G** (100%)	**A** (12%)	**G** (100%)	**G** (100%)

n.a.: not available; the variant frequency is referred to the percentage of the highlighted base in the sequenced ancient hominine genome, with* frequency≤10% and ** counts<10. In light orange are underlined the variants fixed at 100% in modern human compared to ancient hominines.
