# Supplementary material for: A Catalog of Coding Sequence Variations in Salivary Proteins’ Genes Occurring during Recent Human Evolution
Source: Int J Mol Sci. 2023 Oct 9;24(19):15010. doi: 10.3390/ijms241915010 (PMC10573131; doi:10.3390/ijms241915010)
Supplement: Supplementary file 1 [file ijms-24-15010-s001.zip › Table S6 .pdf]

Table S6. Nucleotide Substitutions in *HTN1* gene in introns and regulatory regions

Genomic Coordinates (hg19)	Gene Region	Modern human	Altai Neanderthal (Variant Frequency)	Chagyrskaya Neanderthal (Variant Frequency)	Vindija Neanderthal (Variant Frequency)	Denisovan (Variant Frequency)	Codon → Amino acid
**70,915,835**	Upstream regions	**G**	**G** (100%)	**A** (6%)*	**A** (12%)	**A** (3%)*	
**70,915,946**	Upstream regions	**A**	**A** (100%)	**A** (100%)	**G** (14%)	**A** (100%)	
**70,915,954**	Upstream regions	**G**	**G** (100%)	**G** (100%)	**A** (12%)	**C** (4%)*	
**70,915,975**	Upstream regions	**C**	**C** (100%)	**T** (12%)	**T** (8%)*	**C** (100%)	
**70,915,993**	Upstream regions	**C**	**C** (100%)	**C** (100%)	**T** (16%)	**C** (100%)	
**70,916,067**	Upstream regions	**A**	**G** (12%)	**G** (4%)*	**G** (10%)*	**A** (100%)	
**70,916,151**	5′UTR	**G**	**G** (98%)	**G** (100%)	**A** (11%)	**G** (100%)	
**70,916,157**	5′UTR	**C**	**T** (4%)*	**C** (100%)	**T** (12%)	**C** (100%)	
**70,916,201**	5′UTR	**C**	**C** (100%)	**T** (14%)	**C** (100%)	**C** (100%)	
**70,916,229**	Intron 1	**C**	**C** (99%)	**C** (100%)	**T** (12%)	**C** (100%)	
**70,916,433**	Intron 1	**T**	**G** (93%)	**G** (86%) ** **A** (14%) **	**G** (67%) ** **A** (33%) **	**T** (100%)	
**70,916,454**	Intron 1	**C**	**C** (100%)	**C** (100%)	**T** (15%)	**C** (100%)	
**70,916,507**	Intron 1	**G**	**G** (97%)	**A** (5%)*	**A** (15%)	**G** (100%)	
**70,916,513**	Intron 1	**G**	**G** (100%)	**A** (13%)	**G** (100%)	**G** (100%)	
**70,916,527**	Intron 1	**G**	**A** (100%)	**A** (100%)	**A** (100%)**	**A** (100%)**	
**70,916,549**	Intron 1	**G**	**A** (7%)*	**A** (7%)*	**A** (15%)	**T** (8%)*	
**70,916,629**	Intron 1	**C**	**C** (100%)	**T** (4%)*	**T** (11%)	**C** (100%)	
**70,916,745**	Intron 1	**C**	**C** (100%)	**T** (11%)	**C** (100%)	**C** (100%)	
**70,916,801**	Intron 1	**G**	**G** (100%)	**G** (100%)	**A** (13%)	**G** (100%)	
**70,916,810**	Intron 1	**C**	**T** (2%)*	**C** (100%)	**T** (10%)*	**T** (100%)	
**70,916,855**	Intron 1	**C**	**G** (100%)	**G** (100%)	**G** (100%)	**G** (100%)	
**70,916,970**	Intron 1	**C**	**C** (100%)	**T** (13%)	**T** (3%)*	**C** (100%)	
**70,917,037**	Intron 1	**G**	**G** (100%)	**A** (3%)*	**A** (13%)	**G** (100%)	
**70,917,063**	Intron 1	**G**	**G** (100%)	**A** (13%)	**A** (3%)*	**G** (100%)	
**70,917,193**	Intron 1	**G**	**A** (100%)	**A** (100%)	**A** (100%)	**A** (100%)	
**70,917,298**	Intron 1	**C**	**C** (100%)	**C** (94%)	**T** (14%)	**C** (100%)	
**70,917,390**	Intron 1	**C**	**C** (100%)	**T** (11%)	**C** (100%)	**C** (100%)	
**70,917,413**	Intron 1	**T**	**A** (98%)	**A** (100%)	**A** (100%)	**A** (100%)	
**70,917,415**	Intron 1	**G**	**G** (100%)	**A** (11%)	**A** (3%)*	**G** (100%)	
**70,917,429**	Intron 1	**A**	**G** (98%)	**G** (97%)	**G** (100%)	**G** (100%)	

**70,917,485**	Intron 1	**G**	**A** (2%)*	**G** (100%)	**A** (11%)	**G** (100%)
**70,917,487**	Intron 1	**G**	**G** (100%)	**G** (100%)	**A** (16%)	**G** (100%)
**70,917,494**	Intron 1	**G**	**G** (100%)	**A** (11%)	**A** (6%)*	**G** (100%)
**70,917,498**	Intron 1	**G**	**A** (2%)*	**A** (5%)*	**A** (19%)	**G** (100%)
**70,917,669**	Intron 1	**A**	**C** (100%)	**C** (100%)	**C** (100%)	**A** (100%)
**70,917,801**	Intron 1	**C**	**C** (100%)	**C** (100%)	**T** (13%)	**C** (100%)
**70,917,152**	Intron 1	**C**	**C** (100%)	**T** (3%)*	**T** (11%)	**C** (100%)
**70,917,227**	Intron 1	**C**	**C** (100%)	**C** (100%)	**T** (17%)	**C** (100%)
**70,917,230**	Intron 1	**C**	**C** (100%)	**T** (11%)	**C** (100%)	**C** (100%)
**70,917,401**	Intron 1	**C**	**C** (100%)	**T** (12%)	**C** (100%)	**C** (100%)
**70,917,452**	Intron 1	**G**	**G** (100%)	**A** (15%)	**A** (2%)*	**G** (100%)
**70,917,509**	Intron 1	**C**	**A** (2%)*	**T** (11%)	**T** (9%)*	T (3%)*
**70,918,511**	Intron 1	**A**	**delA** (15%)	**delA** (48%)	**delA** (32%)	**delA** (21%)
**70,918,512**	Intron 1	**A**	**delA** (5%)*	**delA** (14%)	**delA** (11%)	**delA** (3%)*
**70,918,551**	Intron 1	**G**	**G** (100%)	**A** (4%)*	**A** (15%)	**G** (100%)
**70,918,555**	Intron 1	**C**	**C** (100%)	**T** (10%)*	**T** (15%)	**C** (100%)
**70,918,557**	Intron 1	**C**	**C** (100%)	**C** (100%)	**T** (15%)	**C** (100%)
**70,918,559**	Intron 1	**C**	**T** (2%)*	**C** (100%)	**T** (17%)	**C** (100%)
**70,918,620**	Intron 1	**G**	**A** (2%)*	**A** (14%)	**A** (5%)*	**G** (100%)
**70,918,780**	Intron 1	**G**	**G** (100%)	**A** (3%)*	**A** (11%)	**G** (100%)
**70,918,901**	Intron 2	**C**	**T** (2%)*	**T** (6%)*	**T** (13%)	**C** (100%)
**70,918,978**	Intron 2	**G**	**G** (100%)	**A** (8%)*	**A** (12%)	**G** (100%)
**70,918,980**	Intron 2	**G**	**G** (100%)	**A** (8%)*	**A** (12%)	**G** (100%)
**70,918,984**	Intron 2	**C**	**C** (100%)	**T** (3%)*	**T** (13%)	**C** (97%)
**70,919,320**	Intron 2	**A**	**C** (100%)	**C** (100%)	**C** (100%)	**A** (100%)
**70,919,342**	Intron 2	**A**	**A** (100%)	**A** (100%)	**A** (100%)	**G** (100%)
**70,919,353**	Intron 2	**G**	**A** (2%)*	**A** (14%)	**A** (8%)*	**G** (100%)
**70,919,355**	Intron 2	**C**	**C** (100%)	**T** (3%)*	**T** (13%)	**C** (100%)
**70,919,413**	Intron 2	**C**	**C** (100%)	**T** (4%)*	**T** (11%)	**C** (100%)
**70,919,418**	Intron 2	**T**	**C** (100%)	**C** (100%)	**C** (100%)	**T** (100%)
**70,919,448**	Intron 2	**C**	**C** (100%)	**T** (11%)	**C** (100%)	**C** (100%)
**70,919,615**	Intron 2	**G**	**G** (100%)	**A** (11%)	**G** (100%)	**G** (100%)
**70,919,739**	Intron 2	**A**	**G** (95%)	**G** (100%)	**G** (97%)	**A** (100%)
**70,919,775**	Intron 2	**C**	**C** (100%)	**T** (13%)	**T** (5%)*	**C** (100%)
**70,919,777**	Intron 2	**G**	**G** (100%)	**A** (4%)*	**A** (14%)	**G** (100%)

**70,919,796**	Intron 2	**T**	**G** (95%)	**G** (100%)	**G** (92%)	**T** (100%)	
**70,919,872**	Intron 2	**C**	**C** (100%)	**C** (100%)	**T** (11%)	**C** (100%)	
**70,920,060**	Intron 3	**G**	**G** (98%)	**A** (11%)	**A** (7%)*	**G** (100%)	
**70,920,149**	Exon 4	GG**G**	GG**A** (2%)*	GG**A** (5%)*	GG**A** (17%)	GG**G** (100%)	GGG→G_9<br>GGA→G_9
**70,920,408**	Intron 4	**A**	**A** (100%)	**A** (97%)	**T** (60%)	**A** (100%)	
**70,920,473**	Intron 4	**G**	**G** (100%)	**A** (10%)*	**A** (14%)	**G** (100%)	
**70,920,499**	Intron 4	**G**	**A** (5%)*	**A** (3%)*	**A** (12%)	**G** (100%)	
**70,920,608**	Intron 4	**A**	**G** (95%)	**G** (100%)	**G** (100%)	**A** (100%)	
**70,920,715**	Intron 4	**G**	**G** (100%)	**A** (17%)	**G** (100%)	**G** (100%)	
**70,920,879**	Intron 4	**C**	**C** (100%)	**C** (100%)	**T** (11%)	**C** (100%)	
**70,920,880**	Intron 4	**C**	**C** (100%)	**C** (100%)	**T** (11%)	**C** (100%)	
**70,920,916**	Intron 4	**G**	**G** (100%)	**G** (100%)	**A** (11%)	**G** (100%)	
**70,920,989**	Intron 4	**G**	**A** (2%)*	**G** (96%)	**A** (13%)	**G** (100%)	
**70,921,018**	Intron 4	**C**	**C** (100%)	**C** (100%)	**T** (12%)	**C** (100%)	
**70,921,122**	Intron 4	**G**	**A** (98%)	**A** (100%)	**A** (100%)	**G** (100%)	
**70,921,164**	Intron 4	**T**	**C** (99%)	**C** (90%)	**C** (97%)	**C** (100%)	
**70,921,174**	Intron 4	**C**	**C** (99%)	**T** (8%)*	**T** (15%)	**C** (100%)	
**70,921,295**	3′UTR	**G**	**G** (100%)	**A** (14%)	**A** (5%)*	**G** (100%)	
**70,921,306**	3′UTR	**G**	**G** (100%)	**G** (96%)	**A** (11%)	**G** (100%)	
**70,921,319**	3′UTR	**G**	**G** (100%)	**G** (100%)	**A** (11%)	**G** (100%)	
**70,921,329**	Intron 5	**C**	**C** (100%)	**C** (100%)	**T** (11%)	**C** (100%)	
**70,921,331**	Intron 5	**C**	**C** (100%)	**C** (100%)	**T** (11%)	**C** (100%)	
**70,921,337**	Intron 5	**G**	**A** (98%)	**A** (100%)	**A** (100%)	**A** (100%)	
**70,921,357**	Intron 5	**C**	**C** (100%)	**T** (11%)	**T** (10%)*	**C** (100%)	
**70,921,393**	Intron 5	**C**	**T** (2%)*	**T** (16%)	**C** (100%)	**T** (4%)*	
**70,921,419**	Intron 5	**C**	**C** (98%)	**C** (100%)	**C** (96%)	**A** (100%)	
**70,921,450**	Intron 5	**G**	**A** (98%)	**A** (100%)	**A** (100%)	**A** (100%)	
**70,921,525**	Intron 5		**InsT** (92%)	**InsC** (45%)	**InsC** (44%)	**InsT** (100%)	
**70,921,537**	Intron 5	**G**	**G** (100%)	**G** (100%)	**A** (20%)	**G** (100%)	
**70,921,564**	Intron 5	**C**	**C** (100%)	**T** (20%)	**T** (15%)	**C** (100%)	
**70,921,603**	Intron 5	**C**	**C** (100%)	**T** (5%)*	**T** (11%)	**C** (96%)	
**70,921,704**	Intron 5	**A**	**G** (93%)	**G** (96%)	**G** (67%)	**A** (100%)	
**70,921,706**	Intron 5	**G**	**G** (98%)	**A** (4%)*	**A** (14%)	**G** (100%)	
**70,921,707**	Intron 5	**G**	**A** (2%)*	**A** (4%)*	**A** (13%)	**G** (100%)	

**70,921,710**	Intron 5	**A**	**G** (89%)	**G** (89%)	**G** (72%)	**A** (100%)
**70,921,724**	Intron 5	**G**	**G** (100%)	**G** (96%)	**A** (11%)	**G** (100%)
**70,921,748**	Intron 5	**A**	**G** (93%)	**G** (81%)	**G** (59%)	**A** (100%)
**70,921,788**	Intron 5	**A**	**T** (92%)	**T** (81%)	**T** (72%)	**A** (100%)
**70,921,911**	Intron 5	**T**	**C** (52%)	**C** (49%)	**C** (26%)	**T** (100%)
**70,921,969**	Intron 5	**G**	**A** (3%)*	**G** (100%)	**A** (13%)	**G** (100%)
**70,922,002**	Intron 5	**A**	**G** (100%)	**G** (97%)	**G** (93%)	**A** (100%)
**70,922,084**	Intron 5	**C**	**C** (100%)	**T** (14%)	**C** (100%)	**C** (97%)
**70,922,163**	Intron 5	**C**	**C** (100%)	**C** (100%)	**T** (17%)	**C** (100%)
**70,922,167**	Intron 5	**G**	**G** (100%)	**G** (100%)	**A** (14%)	**G** (100%)
**70,922,174**	Intron 5	**C**	**C** (100%)	**T** (11%)	**T** (27%)	**T** (4%)*
**70,922,175**	Intron 5	**C**	**T** (2%)*	**T** (5%)*	**T** (27%)	**C** (100%)
**70,922,194**	Intron 5	**C**	**C** (100%)	**C** (100%)	**T** (13%)	**C** (100%)
**70,922,298**	Intron 5	**A**	**A** (100%)	**A** (100%)	**A** (100%)	**G** (43%)
**70,922,383**	Intron 5	**C**	**T** (2%)*	**T** (3%)*	**T** (11%)	**C** (100%)
**70,922,567**	Intron 5	**C**	**C** (100%)	**C** (100%)	**T** (12%)	**C** (100%)
**70,922,656**	Intron 5	**G**	**G** (100%)	**A** (11%)	**A** (7%)*	**A** (3%)*
**70,922,713**	Intron 5	**G**	**G** (100%)	**A** (12%)	**G** (100%)	**G** (100%)
**70,922,717**	Intron 5	**C**	**T** (2%)*	**C** (100%)	**T** (14%)	**C** (100%)
**70,922,892**	Intron 5	**G**	**A** (2%)*	**A** (13%)	**G** (100%)	**G** (100%)
**70,923,008**	Intron 5	**A**	**T** (5%)*	**A** (100%)	**T** (12%)	**A** (100%)
**70,923,009**	Intron 5	**A**	**C** (5%)*	**A** (100%)	**C** (12%)	**A** (100%)
**70,923,015**	Intron 5	**C**	**T** (4%)*	**C** (100%)	**T** (12%)	**C** (100%)
**70,923,066**	Intron 5	**G**	**G** (100%)	**A** (4%)*	**A** (11%)	**G** (100%)
**70,923,077**	Intron 5	**G**	**G** (100%)	**A** (5%)*	**A** (13%)	**G** (100%)
**70,923,088**	Intron 5	**C**	**C** (100%)	**C** (100%)	**T** (14%)	**C** (100%)
**70,923,101**	Intron 5	**G**	**G** (100%)	**A** (13%)	**G** (100%)	**G** (100%)
**70,923,105**	Intron 5	**G**	**G** (100%)	**A** (5%)*	**A** (15%)	**G** (100%)
**70,923,108**	Intron 5	**G**	**G** (97%)	**A** (4%)*	**A** (17%)	**G** (100%)
**70,923,123**	Intron 5		**InsTT** (100%)	**InsTT** (46%)	**InsTT** (50%)	-
**70,923,154**	Intron 5	**C**	**T** (93%)	**T** (90%)	**T** (50%)	**C** (100%)
**70,923,180**	Intron 5	**G**	**G** (100%)	**G** (100%)	**A** (11%)	**G** (100%)
**70,923,254**	Intron 5	**C**	**C** (100%)	**T** (5%)*	**T** (13%)	**C** (100%)
**70,923,276**	Intron 5	**C**	**C** (100%)	**C** (100%)	**T** (12%)	**C** (100%)
**70,923,278**	Intron 5	**C**	**C** (100%)	**C** (96%)	**T** (12%)	**C** (100%)

**70,923,315**	Intron 5	**G**	**A** (2%)*	**A** (10%)*	**A** (12%)	**G** (100%)
**70,923,371**	Intron 5	**C**	**T** (4%)*	**C** (100%)	**T** (13%)	**T** (6%)*
**70,923,380**	Intron 5	**C**	**T** (100%)	**T** (100%)	**T** (100%)	**C** (100%)
**70,923,451**	Intron 5	**G**	**A** (2%)*	**G** (100%)	**A** (11%)	**G** (100%)
**70,923,457**	Intron 5	**G**	**G** (100%)	**A** (5%)*	**A** (15%)	**G** (100%)
**70,923,461**	Intron 5	**G**	**G** (100%)	**G** (100%)	**A** (11%)	**G** (100%)
**70,923,493**	Intron 5	**C**	**C** (100%)	**C** (100%)	**T** (12%)	**C** (100%)
**70,923,498**	Intron 5	**A**	**T** (97%)	**T** (100%)	**T** (100%)	**A** (100%)
**70,923,630**	Intron 5	**G**	**G** (100%)	**G** (100%)	**A** (15%)	**G** (100%)
**70,923,635**	Intron 5	**G**	**G** (98%)	**A** (3%)*	**A** (11%)	**G** (100%)
**70,923,826**	Intron 5	**C**	**C** (99%)	**T** (2%)*	**T** (11%)	**C** (100%)
**70,923,913**	Intron 5	**C**	**C** (97%)	**T** (7%)*	**T** (12%)	**C** (100%)
**70,923,922**	Intron 5	**C**	**C** (100%)	**C** (100%)	**T** (11%)	**C** (100%)
**70,923,931**	Intron 5	**T**	**G** (98%)	**G** (100%)	**G** (100%)	**G** (100%)
**70,923,932**	Intron 5	**A**	**C** (98%)	**C** (100%)	**C** (94%)	**A** (100%)
**70,923,940**	Intron 5	**G**	**G** (100%)	**A** (44%)	**A** (13%)	**G** (100%)
**70,923,943**	Intron 5	**G**	**G** (100%)	**G** (100%)	**A** (17%)	**G** (100%)
**70,924,022**	Intron 5	**G**	**G** (100%)	**G** (100%)	**A** (16%)	**G** (100%)
**70,924,034**	Intron 5	**G**	**G** (100%)	**A** (4%)*	**A** (11%)	**G** (100%)
**70,924,036**	Intron 5	**G**	**G** (98%)	**G** (100%)	**A** (14%)	**G** (100%)
**70,924,091**	Intron 5	**G**	**G** (99%)	**A** (11%)	**G** (100%)	**G** (100%)
**70,924,131**	Intron 5	**C**	**C** (100%)	**T** (11%)	**C** (100%)	**C** (100%)
**70,924,200**	Intron 5	**C**	**C** (100%)	**T** (12%)	**C** (100%)	**C** (100%)
**70,924,204**	Intron 5	**C**	**C** (100%)	**T** (8%)*	**T** (15%)	**C** (100%)
**70,924,250**	Intron 5	**G**	**A** (2%)*	**A** (11%)	**G** (100%)	**G** (100%)
**70,924,345**	3′UTR	**C**	**C** (100%)	**T** (3%)*	**T** (11%)	**C** (100%)
**70,924,346**	3′UTR	**C**	**C** (100%)	**T** (3%)*	**T** (26%)	**T** (3%)*
**70,924,358**	3′UTR	**C**	**C** (100%)	**T** (14%)	**C** (100%)	**C** (100%)
**70,924,359**	3′UTR	**C**	**C** (100%)	**T** (15%)	**C** (100%)	**A** (3%)*
**70,924,419**	3′UTR	**T**	**T** (100%)	**T** (100%)	**T** (100%)	**C** (98%)
**70,924,467**	3′UTR	**G**	**G** (100%)	**A** (14%)	**A** (6%)*	**G** (100%)
**70,924,568**	Downstream regions	**T**	**C** (98%)	**C** (100%)	**C** (89%)	**T** (100%)
**70,924,569**	Downstream regions	**G**	**A** (5%)*	**A** (10%)*	**A** (12%)	**G** (100%)
**70,924,574**	Downstream regions	**C**	**C** (100%)	**T** (11%)	**T** (13%)	**C** (100%)

**70,924,576**	Downstream regions	**C**	**C** (100%)	**C** (100%)	**T** (12%)	**C** (100%)
**70,924,584**	Downstream regions	**C**	**C** (100%)	**T** (13%)	**C** (100%)	**C** (100%)
**70,924,585-70,924,587**	Downstream regions	**TCT**	**delTCT** (98%)	**delTCT** (74%)	**delTCT** (93%)	**TCT** (100%)
**70,924,658**	Downstream regions	**G**	**G** (100%)	**A** (13%)	**A** (3%)*	**G** (100%)
**70,924,661**	Downstream regions	**C**	**C** (100%)	**C** (100%)	**T** (12%)	**C** (100%)
**70,924,669**	Downstream regions	**C**	**T** (80%)	**T** (96%)	**T** (76%)	**T** (3%)*
**70,924,671**	Downstream regions	**T**	**C** (76%)	**C** (90%)	**C** (73%)	**T** (100%)
**70,924,735**	Downstream regions	**G**	**G** (100%)	**G** (100%)	**A** (16%)	**G** (100%)
**70,924,736**	Downstream regions	**G**	**G** (100%)	**A** (13%)	**A** (11%)	**G** (100%)
**70,924,747**	Downstream regions	**C**	**C** (100%)	**C** (100%)	**T** (11%)	**C** (100%)
**70,924,758**	Downstream regions	**C**	**C** (100%)	**T** (13%)	**C** (100%)	**C** (100%)
**70,924,767**	Downstream regions	**C**	**C** (100%)	**C** (100%)	**T** (12%)	**C** (100%)
**70,924,794**	Downstream regions	**G**	**G** (96%)	**A** (11%)	**A** (7%)*	**G** (100%)
**70,924,833**	Downstream regions	**C**	**delC** (97%)	**delC** (79%)	**delC** (95%)	**C** (100%)
**70,924,852**	Downstream regions	**G**	**G** (100%)	**A** (11%)	**A** (2%)*	**G** (100%)
**70,924,873**	Downstream regions	**C**	**C** (100%)	**T** (6%)*	**T** (18%)	**C** (96%)
**70,924,894**	Downstream regions	**G**	**G** (100%)	**G** (100%)	**A** (12%)	**G** (100%)

n.a.: not available; the variant frequency is referred to the percentage of the highlighted base in the sequenced ancient hominine genome, with* frequency≤10% and ** counts<10. In light orange are underlined the variants fixed at 100% in modern human compared to ancient hominines.
