# Supplementary material for: A Catalog of Coding Sequence Variations in Salivary Proteins’ Genes Occurring during Recent Human Evolution
Source: Int J Mol Sci. 2023 Oct 9;24(19):15010. doi: 10.3390/ijms241915010 (PMC10573131; doi:10.3390/ijms241915010)
Supplement: Supplementary file 1 [file ijms-24-15010-s001.zip › Table S7.pdf]

Table S7. Nucleotide Substitutions in *HTN3* gene in introns and regulatory regions

Genomic Coordinates (hg19)	Gene Region	Modern human	Altai Neanderthal (Variant Frequency)	Chagyrskaya Neanderthal (Variant Frequency)	Vindija Neanderthal (Variant Frequency)	Denisovan (Variant Frequency)	Codon → amino acid
**70,893,747**	Upstream regions	**C**	**C** (100%)	**T** (4%)*	**T** (13%)	**C** (100%)	
**70,893,792**	Upstream regions	**C**	**C** (100%)	**C** (100%)	**T** (12%)	**T** (3%)*	
**70,893,878**	Upstream regions	**C**	**C** (100%)	**T** (12%)	**T** (2%)*	**C** (100%)	
**70,893,946**	Upstream regions	**A**	**A** (100%)	**A** (100%)	**G** (27%)	**A** (100%)	
**70,893,955**	Upstream regions	**G**	**G** (100%)	**G** (100%)	**A** (19%)	**G** (100%)	
**70,893,964**	Upstream regions	**C**	**C** (98%)	**C** (100%)	**T** (17%)	**C** (100%)	
**70,893,976**	Upstream regions	**C**	**C** (100%)	**C** (100%)	**T** (17%)	**C** (100%)	
**70,894,056**	Upstream regions	**C**	**C** (100%)	**T** (13%)	**C** (100%)	**C** (100%)	
**70,894,081**	Upstream regions	**A**	**C** (100%)	**C** (93%)	**C** (100%)	**A** (100%)	
**70,894,130**	5′UTR	**G**	**G** (100%)	**A** (12%)	**A** (8%)*	**G** (100%)	
**70,894,131**	5′UTR	**G**	**G** (100%)	**G** (100%)	**A** (13%)	**A** (3%)*	
**70,894,159**	5′UTR	**G**	**G** (100%)	**A** (5%)*	**A** (17%)	**G** (100%)	
**70,894,234**	Intron 1	**G**	**A** (2%)*	**A** (11%)	**A** (8%)*	**G** (100%)	
**70,894,357**	Intron 1	**C**	**C** (100%)	**T** (11%)	**T** (3%)*	**C** (100%)	
**70,894,468**	Intron 1	**C**	**C** (100%)	**T** (4%)*	**T** (12%)	**C** (100%)	
**70,894,547**	Intron 1	**A**	**A** (100%)	**A** (100%)	**A** (100%)	**C** (100%)	
**70,894,581**	Intron 1	**C**	**C** (100%)	**C** (100%)	**T** (11%)	**C** (100%)	
**70,894,640**	Intron 1	**C**	**C** (97%)	**C** (100%)	**T** (13%)	**C** (100%)	
**70,894,751**	Intron 1	**C**	**C** (100%)	**T** (11%)	**C** (100%)	**C** (100%)	
**70,894,799**	Intron 1	**C**	**C** (100%)	**C** (100%)	**T** (11%)	**C** (100%)	
**70,894,878**	Intron 1	**C**	**C** (100%)	**C** (100%)	**T** (15%)	**C** (100%)	
**70,894,931**	Intron 1	**C**	**T** (2%)*	**T** (13%)	**T** (5%)*	**C** (100%)	
**70,895,173**	Intron 1	**G**	**G** (98%)	**A** (3%)*	**A** (14%)	**G** (100%)	
**70,895,228**	Intron 1	**C**	**C** (100%)	**T** (10%)*	**T** (18%)	**C** (100%)	
**70,895,232**	Intron 1	**C**	**T** (2%)*	**C** (100%)	**T** (19%)	**C** (100%)	
**70,895,241**	Intron 1	**C**	**C** (100%)	**C** (100%)	**T** (12%)	**C** (100%)	
**70,895,243**	Intron 1	**G**	**G** (100%)	**A** (13%)	**G** (100%)	**G** (100%)	
**70,895,244**	Intron 1	**G**	**G** (100%)	**A** (13%)	**G** (100%)	**G** (100%)	
**70,895,361**	Intron 1	**A**	**T** (63%)	**A** (100%)	**A** (100%)	**A** (100%)	

**70,895,411**	Intron 1	**C**	**C** (100%)	**T** (12%)	**C** (100%)	**C** (100%)
**70,895,437-70,895,438**	Intron 1	**AC**	**delAC** (72%)	**delAC** (72%)**	**delAC** (83%)**	**AC** (100%)
**70,895,440**	Intron 1	**G**	**A** (100%)	**A** (100%)**	**A** (100%)**	**G** (100%)
**70,895,441**	Intron 1	**G**	**T** (100%)	**T** (100%)**	**T** (100%)**	**G** (100%)
**70,895,444**	Intron 1	**T**	**G** (100%)	**G** (80%)**	**G** (100%)**	**T** (100%)
**70,895,466-70,895,471**	Intron 1	**AAAAAC**	**AAAAAC** (100%)	**AAAAAC** (100%)	**delAAAAAC** (12%)	**AAAAAC** (100%)
**70,895,478**	Intron 1	**G**	**G** (100%)	**G** (100%)	**A** (14%)	**G** (100%)
**70,895,494**	Intron 1	**T**	**A** (4%)*	**A** (11%)	**T** (100%)	**T** (100%)
**70,895,495**	Intron 1		**InsTTG** (82%)	**InsTTG** (64%)	**InsTTG** (95%)	-
**70,895,572**	Intron 1	**G**	**G** (100%)	**A** (11%)	**G** (97%)	**G** (100%)
**70,895,628**	Intron 1	**G**	**G** (98%)	**A** (8%)*	**A** (15%)	**G** (100%)
**70,895,643**	Intron 1	**C**	**C** (100%)	**C** (100%)	**T** (13%)	**C** (100%)
**70,895,697**	Intron 1	**C**	**C** (100%)	**C** (100%)	**T** (16%)	**C** (100%)
**70,895,736**	Intron 1	**G**	**G** (98%)	**A** (7%)*	**A** (12%)	**G** (100%)
**70,895,787**	Intron 1	**G**	**G** (98%)	**A** (3%)*	**A** (11%)	**G** (100%)
**70,895,846**	Intron 1	**G**	**A** (2%)*	**A** (11%)	**G** (100%)	**G** (100%)
**70,895,900**	Intron 1	**C**	**C** (100%)	**T** (14%)	**T** (5%)*	**T** (3%)*
**70,895,902**	Intron 1	**C**	**C** (100%)	**T** (14%)	**T** (12%)	**C** (100%)
**70,895,905**	Intron 1	**C**	**C** (100%)	**T** (5%)*	**T** (13%)	**C** (100%)
**70,895,912**	Intron 1	**C**	**C** (100%)	**T** (14%)	**T** (13%)	**C** (100%)
**70,895,962**	Intron 1	**C**	**C** (100%)	**T** (5%)*	**T** (11%)	**C** (100%)
**70,896,213**	Intron 1	**G**	**G** (100%)	**A** (15%)	**A** (5%)*	**G** (100%)
**70,896,255**	Intron 1	**G**	**G** (100%)	**A** (11%)	**G** (100%)	**G** (100%)
**70,896,257**	Intron 1	**C**	**C** (100%)	**C** (100%)	**T** (14%)	**C** (100%)
**70,896,285**	Intron 1	**G**	**G** (100%)	**G** (100%)	**A** (16%)	**G** (100%)
**70,896,365**	Intron 1	**G**	**G** (100%)	**G** (100%)	**A** (11%)	**G** (100%)
**70,896,509**	Intron 2	**G**	**G** (100%)	**G** (100%)	**A** (11%)	**G** (100%)
**70,896,812**	Intron 2	**C**	**C** (100%)	**T** (18%)	**T** (4%)*	**C** (100%)
**70,896,813**	Intron 2	**C**	**C** (100%)	**T** (17%)	**T** (5%)*	**C** (100%)
**70,896,839**	Intron 2	**G**	**G** (100%)	**A** (11%)	**A** (4%)*	**G** (100%)
**70,896,875**	Intron 2	**G**	**G** (100%)	**G** (100%)	**A** (11%)	**G** (100%)
**70,896,876**	Intron 2	**G**	**G** (100%)	**A** (6%)*	**A** (11%)	**G** (100%)
**70,896,877**	Intron 2	**G**	**G** (100%)	**G** (100%)	**A** (11%)	**G** (100%)
**70,896,879**	Intron 2	**G**	**G** (100%)	**G** (100%)	**A** (13%)	**G** (100%)

**70,897,046**	Intron 2	**G**	**G** (98%)	**G** (100%)	**A** (13%)	**G** (100%)	
**70,897,113**	Intron 2	**C**	**C** (100%)	**T** (14%)	**C** (100%)	**C** (100%)	
**70,897,179**	Intron 2	**C**	**T** (2%)*	**T** (6%)*	**T** (14%)	**C** (100%)	
**70,897,184**	Intron 2	**C**	**C** (100%)	**C** (100%)	**T** (18%)	**C** (100%)	
**70,897,340**	Intron 2	**G**	**G** (100%)	**G** (100%)	**A** (12%)	**G** (100%)	
**70,897,444**	Intron 2	**C**	**C** (100%)	**T** (3%)*	**T** (11%)	**C** (100%)	
**70,897,522**	Intron 2	**C**	**C** (100%)	**C** (100%)	**T** (11%)	**C** (97%)	
**70,897,523**	Intron 2	**C**	**C** (98%)	**C** (100%)	**T** (11%)	**C** (100%)	
**70,897,571**	Intron 2	**A**	**G** (98%)	**G** (100%)	**G** (96%)	**A** (100%)	
**70,897,695**	Intron 2	**G**	**G** (100%)	**A** (4%)*	**A** (11%)	**G** (100%)	
**70,897,717**	Intron 3	**G**	**G** (100%)	**A** (10%)*	**A** (12%)	**G** (100%)	
**70,897,806**	Exon 4	GG**G**	GG**G** (100%)	GG**A** (12%)	GG**A** (5%)*	GG**G** (100%)	GGG→G_9<br>GGA→G_9
**70,897,829**	Intron 4	**G**	**G** (100%)	**A** (5%)*	**A** (13%)	**G** (100%)	
**70,898,014**	Intron 4	**C**	**G** (100%)	**G** (96%)*	**G** (95%)	**C** (100%)	
**70,898,016**	Intron 4	**G**	**A** (2%)*	**A** (17%)	**A** (5%)*	**G** (100%)	
**70,898,027**	Intron 4	**A**	**C** (100%)	**C** (100%)	**C** (98%)	**A** (100%)	
**70,898,115**	Intron 4	**C**	**C** (100%)	**C** (100%)	**T** (11%)	**C** (97%)	
**70,898,122**	Intron 4	**C**	**C** (98%)	**T** (13%)	**T** (3%)*	**C** (100%)	
**70,898,177**	Intron 4	**G**	**A** (2%)*	**A** (11%)	**A** (10%)*	**G** (100%)	
**70,898,289**	Intron 4	**G**	**G** (100%)	**G** (100%)	**A** (11%)	**G** (100%)	
**70,898,340**	Intron 4	**G**	**A** (98%)	**A** (100%)	**A** (100%)	**G** (100%)	
**70,898,459**	Intron 4	**G**	**G** (100%)	**A** (3%)*	**A** (11%)	**G** (100%)	
**70,898,488**	Intron 4	**T**	**T** (100%)	**T** (100%)	**T** (100%)	**C** (100%)	
**70,898,561**	Intron 4	**G**	**G** (100%)	**G** (100%)	**A** (11%)	**G** (100%)	
**70,898,562**	Intron 4	**G**	**G** (100%)	**A** (3%)*	**A** (11%)	**G** (100%)	
**70,898,563**	Intron 4	**G**	**G** (100%)	**A** (6%)*	**A** (11%)	**G** (100%)	
**70,898,617**	Intron 4	**G**	**G** (100%)	**G** (97%)	**A** (11%)	**G** (100%)	
**70,898,619**	Intron 4	**G**	**G** (100%)	**G** (100%)	**A** (12%)	**G** (100%)	
**70,898,620**	Intron 4	**G**	**G** (100%)	**A** (10%)*	**A** (16%)	**G** (100%)	
**70,898,623**	Intron 4	**G**	**G** (100%)	**G** (100%)	**A** (13%)	**G** (100%)	
**70,898,722**	Intron 4	**G**	**A** (99%)	**A** (100%)	**A** (100%)	**G** (100%)	
**70,898,811**	Intron 4	**G**	**G** (100%)	**A** (9%)*	**A** (11%)	**G** (100%)	
**70,898,954**	3′UTR	**G**	**A** (100%)	**A** (100%)	**A** (100%)	**G** (100%)	
**70,899,036**	Intron 5	**T**	**T** (100%)	**delT** (12%)	**T** (97%)	**T** (100%)	

**70,899,037**	Intron 5	**T**	**A** (100%)	**A** (88%)	**A** (100%)	**A** (95%)
**70,899,067**	Intron 5	**C**	**T** (1%)*	**T** (6%)*	**T** (12%)	**C** (100%)
**70,899,203**	Intron 5	**C**	**T** (9%)*	**T** (18%)	**C** (100%)	**T** (7%)*
**70,899,225**	Intron 5	**C**	**C** (96%)	**C** (100%)	**T** (19%)	**C** (100%)
**70,899,239**	Intron 5	**G**	**G** (100%)	**A** (4%)*	**A** (11%)	**G** (100%)
**70,899,368**	Intron 5	**T**	**C** (98%)	**C** (91%)	**C** (85%)	**T** (100%)
**70,899,602**	Intron 5	**G**	**G** (100%)	**G** (100%)	**A** (11%)	**G** (100%)
**70,899,668**	Intron 5	**G**	**G** (98%)	**A** (3%)*	**A** (11%)	**G** (100%)
**70,899,676**	Intron 5	**G**	**G** (98%)	**A** (3%)*	**A** (12%)	**G** (100%)
**70,899,819**	Intron 5	**C**	**C** (100%)	**T** (17%)	**T** (3%)*	**C** (100%)
**70,899,836**	Intron 5	**G**	**G** (97%)	**A** (6%)*	**A** (16%)	**G** (100%)
**70,899,837**	Intron 5	**G**	**G** (100%)	**G** (100%)	**A** (14%)	**G** (100%)
**70,900,035**	Intron 5	**G**	**G** (100%)	**A** (13%)	**G** (100%)	**G** (100%)
**70,900,037**	Intron 5	**G**	**G** (100%)	**G** (100%)	**A** (18%)	**G** (100%)
**70,900,040**	Intron 5	**G**	**G** (100%)	**A** (3%)*	**A** (11%)	**G** (100%)
**70,900,108**	Intron 5	**A**	**G** (96%)	**G** (88%)	**G** (86%)	**G** (90%)
**70,900,280**	Intron 5	**G**	**G** (100%)	**A** (3%)*	**A** (11%)	**G** (100%)
**70,900,305**	Intron 5	**C**	**T** (2%)*	**T** (3%)*	**T** (15%)	**C** (100%)
**70,900,315**	Intron 5	**G**	**G** (100%)	**G** (100%)	**A** (11%)	**G** (100%)
**70,900,319**	Intron 5	**G**	**G** (100%)	**A** (3%)*	**A** (21%)	**G** (100%)
**70,900,396**	Intron 5	**C**	**C** (100%)	**C** (100%)	**T** (11%)	**C** (100%)
**70,900,408**	Intron 5	**G**	**A** (90%)	**A** (85%)	**A** (63%)	**G** (100%)
**70,900,563**	Intron 5	**C**	**C** (98%)	**T** (3%)*	**T** (13%)	**C** (97%)
**70,900,602**	Intron 5	**C**	**C** (98%)	**C** (100%)	**T** (4%)*	**T** (100%)
**70,900,603**	Intron 5	**C**	**C** (100%)	**A** (100%)	**A** (41%)	**C** (100%)
**70,900,622**	Intron 5	**G**	**G** (100%)	**G** (100%)	**A** (15%)	**G** (100%)
**70,900,681**	Intron 5	**C**	**C** (100%)	**T** (11%)	**C** (100%)	**C** (100%)
**70,900,683**	Intron 5	**C**	**C** (100%)	**C** (100%)	**T** (11%)	**C** (100%)
**70,900,728**	Intron 5	**G**	**G** (100%)	**A** (11%)	**G** (100%)	**G** (100%)
**70,900,729**	Intron 5	**G**	**G** (100%)	**A** (5%)*	**A** (27%)	**G** (100%)
**70,900,731**	Intron 5	**G**	**G** (100%)	**A** (14%)	**G** (100%)	**G** (100%)
**70,900,734**	Intron 5	**G**	**G** (100%)	**A** (5%)*	**A** (20%)	**G** (100%)
**70,900,751**	Intron 5	**G**	**G** (100%)	**A** (12%)	**G** (100%)	**G** (100%)
**70,900,766**	Intron 5	**G**	**G** (100%)	**A** (10%)*	**A** (17%)	**G** (100%)
**70,900,770**	Intron 5	**C**	**C** (100%)	**C** (100%)	**T** (13%)	**C** (100%)

**70,900,775**	Intron 5	**G**	**G** (100%)	**G** (100%)	**A** (19%)	**G** (100%)
**70,900,776**	Intron 5	**G**	**G** (100%)	**A** (5%)*	**A** (13%)	**G** (100%)
**70,900,799**	Intron 5	**G**	**A** (3%)*	**A** (7%)*	**A** (12%)	**A** (4%)*
**70,900,856**	Intron 5	**C**	**C** (100%)	**T** (3%)*	**T** (12%)	**C** (100%)
**70,900,966**	Intron 5	**C**	**C** (98%)	**T** (4%)*	**T** (14%)	**C** (100%)
**70,901,006**	Intron 5	**C**	**C** (100%)	**T** (11%)	**T** (3%)*	**C** (100%)
**70,901,013**	Intron 5	**G**	**G** (100%)	**A** (11%)	**A** (3%)*	**G** (100%)
**70,901,058**	Intron 5	**C**	**C** (100%)	**T** (5%)*	**T** (15%)	**C** (100%)
**70,901,107**	Intron 5	**G**	**G** (100%)	**A** (14%)	**G** (100%)	**G** (100%)
**70,901,208**	Intron 5	**C**	**C** (100%)	**T** (12%)	**C** (100%)	**C** (100%)
**70,901,275**	Intron 5	**A**	**G** (95%)	**G** (89%)	**G** (98%)	**A** (100%)
**70,901,313**	Intron 5	**G**	**G** (100%)	**A** (14%)	**G** (100%)	**G** (100%)
**70,901,362**	Intron 5	**A**	**G** (93%)	**G** (100%)	**G** (97%)	**A** (100%)
**70,901,432**	Intron 5	**C**	**C** (100%)	**T** (12%)	**T** (2%)*	**C** (100%)
**70,901,488**	Intron 5	**G**	**G** (100%)	**A** (14%)	**G** (100%)	**G** (100%)
**70,901,496**	Intron 5	**C**	**T** (1%)*	**T** (11%)	**T** (6%)*	**C** (100%)
**70,901,552**	Intron 5	**G**	**G** (100%)	**G** (100%)	**A** (14%)	**G** (100%)
**70,901,583**	Intron 5	**C**	**T** (100%)	**T** (100%)	**T** (100%)	**T** (100%)
**70,901,598**	Intron 5	**G**	**G** (100%)	**A** (3%)*	**A** (13%)	**A** (3%)*
**70,901,610**	Intron 5	**A**	**C** (8%)*	**C** (8%)*	**C** (30%)	**A** (100%)
**70,901,729-70,901,731**	Intron 5	**GAT**	**delGAT** (88%)	**delGAT** (72%)	**delGAT** (85%)	**GAT** (100%)
**70,901,736**	Intron 5	**A**	**A** (100%)	**G** (13%)	**A** (100%)	**A** (100%)
**70,901,737**	Intron 5	**T**	**T** (100%)	**A** (13%)	**T** (100%)	**T** (100%)
**70,901,738**	Intron 5	**G**	**G** (98%)	**A** (13%)	**G** (100%)	**G** (100%)
**70,901,739**	Intron 5	**G**	**G** (100%)	**A** (11%)	**G** (100%)	**G** (100%)
**70,901,925**	Intron 5	**A**	**T** (100%)	**T** (100%)	**T** (100%)	**A** (100%)
**70,901,940**	Intron 5	**A**	**G** (100%)	**G** (96%)	**G** (92%)	**A** (100%)
**70,902,051**	3′UTR	**C**	**C** (100%)	**T** (11%)	**T** (4%)*	**C** (100%)
**70,902,079**	3′UTR	**C**	**A** (99%)	**A** (100%)	**A** (100%)	**C** (100%)
**70,902,127**	3′UTR	**C**	**C** (100%)	**T** (6%)*	**T** (14%)	**C** (100%)
**70,902,228**	3′UTR	**C**	**C** (100%)	**T** (2%)*	**T** (11%)	**C** (100%)
**70,902,290**	Downstream region	**C**	**C** (100%)	**T** (2%)*	**T** (13%)	**C** (100%)
**70,902,355**	Downstream region	**C**	**T** (9%)*	**T** (12%)	**T** (35%)	**C** (100%)
**70,902,357**	Downstream region	**T**	**C** (8%)*	**C** (4%)*	**C** (29%)	**T** (100%)

**70,902,601**	Downstream region	**C**	**C** (100%)	**C** (100%)	**T** (12%)	**C** (100%)
**70,902,634**	Downstream region	**G**	**G** (100%)	**G** (100%)	**A** (11%)	**G** (100%)
**70,902,651**	Downstream region	**C**	**T** (2%)*	**T** (7%)*	**T** (18%)	**C** (100%)
**70,902,661**	Downstream region	**C**	**C** (100%)	**T** (12%)	**C** (100%)	**C** (100%)
**70,902,690**	Downstream region	**C**	**C** (100%)	**T** (4%)*	**T** (12%)	**C** (100%)
**70,902,692**	Downstream region	**C**	**C** (100%)	**T** (4%)*	**T** (13%)	**C** (100%)
**70,902,694**	Downstream region	**C**	**C** (100%)	**C** (100%)	**T** (17%)	**C** (100%)

n.a.: not available; the variant frequency is referred to the percentage of the highlighted base in the sequenced ancient hominine genome, with* frequency≤10% and ** counts<10. In light orange are underlined the variants fixed at 100% in modern human compared to ancient hominines.
