# Supplementary material for: A Catalog of Coding Sequence Variations in Salivary Proteins’ Genes Occurring during Recent Human Evolution
Source: Int J Mol Sci. 2023 Oct 9;24(19):15010. doi: 10.3390/ijms241915010 (PMC10573131; doi:10.3390/ijms241915010)
Supplement: Supplementary file 1 [file ijms-24-15010-s001.zip › Table S8 .pdf]

Table S8. Nucleotide Substitutions in *AMY1A* gene in introns and regulatory regions

Genomic Coordinates (hg19)	Gene Region	Modern human	Altai Neanderthal (Variant Frequency)	Chagyrskaya Neanderthal (Variant Frequency)	Vindija Neanderthal (Variant Frequency)	Denisovan (Variant Frequency)	Codon → Amino acid
**104,239,478**	Upstream region	**G**	**G** (100%)	**G** (100%)	**A** (17%)	**G** (100%)	
**104,239,460**	Upstream region	**C**	**C** (100%)	**C** (100%)	**T** (14%)	**C** (100%)	
**104,239,450**	Upstream region	**G**	**G** (100%)	**G** (100%)	**A** (13%)	**G** (100%)	
**104,239,175**	Upstream region	**C**	**C** (100%)	**C** (100%)	**T** (14%)	**C** (100%)	
**104,239,005**	5′UTR	**G**	**G** (100%)	**G** (100%)	**A** (27%)	**G** (100%)	
**104,238,932**	5′UTR	**C**	**C** (100%)	**T** (18%)	**C** (100%)	**C** (100%)	
**104,238,918**	5′UTR	**G**	**G** (100%)	**G** (100%)	**A** (11%)	**G** (100%)	
**104,238,905**	5′UTR	**C**	**C** (100%)	**C** (100%)	**T** (13%)	**C** (100%)	
**104,238,850**	5′UTR	**G**	**G** (100%)	**G** (100%)	**A** (15%)	**G** (100%)	
**104,238,820**	5′UTR	**C**	**C** (100%)	**C** (100%)	**T** (18%)	**C** (100%)	
**104,238,815**	5′UTR	**C**	**C** (100%)	**C** (100%)	**T** (17%)	**C** (100%)	
**104,238,794**	5′UTR	**G**	**A** (100%)	**A** (100%)	**A** (90%)	**A** (100%)**	
**104,238,676**	5′UTR	**G**	**G** (100%)	**G** (100%)	**A** (20%)	**G** (100%)	
**104,238,649**	Intron 1	**G**	**G** (98%)	**A** (8%)*	**A** (18%)	**G** (100%)	
**104,238,648**	Intron 1	**G**	**A** (3%)*	**G** (100%)	**A** (20%)	**G** (100%)	
**104,238,610**	Intron 1	**C**	**T** (3%)*	**C** (100%)	**C** (100%)	**T** (27%)	
**104,238,487**	Intron 1	**T**	**delT** (23%)	**T** (100%)	**delT** (30%)	**delT** (29%)	
**104,238,486**	Intron 1	**C**	**T** (14%)	**C** (100%)	**T** (17%)	**T** (21%)	
**104,238,481**	Intron 1	**C**	**T** (7%)*	**C** (100%)	**C** (100%)	**T** (14%)	
**104,238,366**	Intron 1	**C**	**C** (100%)	**T** (14%)	**C** (100%)	**C** (100%)	
**104,238,362**	Intron 1	**C**	**C** (100%)	**C** (100%)	**T** (11%)	**C** (100%)	
**104,238,354**	Intron 1	**C**	**C** (100%)	**C** (100%)	**T** (11%)	**C** (100%)	
**104,238,317**	Intron 1	**G**	**G** (100%)	**G** (91%)	**A** (11%)	**G** (100%)	
**104,238,094**	Exon 2	GT<u>C</u>	GT<u>C</u> (100%)	GT<u>C</u> (100%)	GT<u>T</u> (17%)	GT<u>C</u> (100%)	CAG→Q_{41}<br>CAA→Q_{41}
**104,238,054**	Intron 2	**C**	**G** (100%)	**G** (100%)	**G** (100%)	**G** (100%)**	
**104,238,050**	Intron 2	**C**	**C** (100%)	**T** (6%)*	**T** (13%)	**C** (100%)	
**104,238,047**	Intron 2	**C**	**C** (100%)	**T** (12%)	**T** (15%)	**C** (100%)	
**104,238,025**	Intron 2	**T**	**T** (100%)	**T** (100%)	**C** (94%)	**T** (100%)	
**104,238,017**	Intron 2	**G**	**G** (100%)	**A** (8%)*	**A** (12%)	**G** (100%)	
**104,237,948**	Intron 2	**C**	**C** (100%)	**C** (100%)	**T** (18%)	**C** (100%)	

**104,237,938**	Intron 2	**G**	**G** (100%)	**G** (100%)	**A** (13%)	**G** (100%)	
**104,237,897**	Intron 2	**C**	**C** (100%)	**T** (8%)*	**T** (13%)	**C** (100%)	
**104,237,850**	Intron 2	**G**	**G** (100%)	**G** (100%)	**A** (15%)	**G** (100%)	
**104,237,816**	Intron 2	**T**	**C** (87%)	**C** (89%)	**C** (100%)	**C** (100%)	
**104,237,595**	Intron 3	**C**	**C** (100%)	**C** (100%)	**T** (14%)	**C** (100%)	
**104,237,546**	Intron 3	**G**	**G** (100%)	**A** (11%)	**A** (8%)*	**G** (100%)	
**104,237,512**	Intron 3	**G**	**G** (100%)	**A** (18%)	**G** (100%)	**G** (100%)	
**104,237,472**	Intron 3	**T**	**C** (100%)	**C** (100%)	**T** (100%)	**T** (100%)	
**104,237,461**	Intron 3	**G**	**G** (100%)	**A** (25%)	**G** (100%)	**G** (100%)	
**104,237,384**	Intron 3	**T**	**G** (100%)	**G** (100%)	**G** (100%)	**G** (100%)	
**104,237,332**	Intron 3	**C**	**C** (100%)	**C** (100%)	**T** (11%)	**C** (100%)	
**104,237,331**	Intron 3	**C**	**C** (100%)	**C** (100%)	**T** (11%)	**C** (100%)	
**104,237,328**	Intron 3	**C**	**C** (100%)	**C** (100%)	**T** (11%)	**C** (100%)	
**104,237,319**	Intron 3	**C**	**C** (100%)	**T** (12%)	**C** (100%)	**C** (100%)	
**104,237,314**	Intron 3	**C**	**C** (100%)	**C** (100%)	**T** (12%)	**C** (100%)	
**104,237,254**	Intron 3	**G**	**G** (100%)	**G** (100%)	**G** (100%)	**A** (93%)	
**104,237,248**	Intron 3	**G**	**G** (100%)	**A** (5%)*	**G** (100%)	**A** (13%)	
**104,237,245**	Intron 3	**C**	**C** (100%)	**C** (100%)	**C** (100%)	**A** (13%)	
**104,237,229**	Intron 3	**C**	**C** (100%)	**T** (12%)	**C** (100%)	**C** (100%)	
**104,237,220**	Intron 3	**C**	**C** (100%)	**T** (11%)	**C** (100%)	**C** (100%)	
**104,237,214**	Intron 3	**G**	**G** (100%)	**A** (14%)	**A** (7%)*	**G** (100%)	
**104,237,205**	Intron 3	**C**	**C** (100%)	**T** (8%)*	**T** (13%)	**C** (100%)	
**104,237,078**	Intron 3	**C**	**A** (100%)	**A** (100%)	**A** (100%)	**A** (100%)**	
**104,236,994**	Intron 3	**C**	**T** (100%)	**T** (100%)	**T** (100%)	**T** (100%)**	
**104,236,974**	Intron 3	**C**	**C** (100%)	**T** (7%)*	**T** (20%)	**C** (100%)	
**104,236,963**	Intron 3	**C**	**C** (100%)	**C** (100%)	**T** (20%)	**C** (100%)	
**104,236,949**	Intron 3	**G**	**G** (100%)	**A** (14%)	**G** (100%)	**G** (100%)	
**104,236,905**	Intron 3	**C**	**C** (100%)	**C** (100%)	**C** (100%)	**T** (79%)	
**104,236,901**	Intron 3	**C**	**C** (100%)	**T** (8%)*	**T** (11%)	**C** (100%)	
**104,236,715**	Exon 4	ATG	ATG (100%)	ATG (100%)	ATA (14%)	ATG (100%)	UAC→Y_{118}<br>UAU→Y_{118}
**104,236,425**	Intron 4	**A**	**A** (100%)	**A** (100%)	**A** (100%)	**C** (100%)	
**104,236,399**	Intron 4	**C**	**C** (100%)	**C** (100%)	**T** (13%)	**C** (100%)	
**104,236,396**	Intron 4	**G**	**G** (100%)	**A** (13%)	**A** (6%)*	**G** (100%)	
**104,236,381**	Intron 4	**G**	**G** (100%)	**G** (100%)	**A** (11%)	**G** (100%)	

**104,236,379**	Intron 4	**G**	**G** (100%)	**G** (100%)	**A** (11%)	**G** (100%)	
**104,236,340**	Intron 4	**G**	**G** (100%)	**A** (14%)	**A** (10%)*	**G** (100%)	
**104,236,262**	Intron 4	**C**	**C** (100%)	**C** (100%)	**T** (13%)	**C** (100%)	
**104,236,261**	Intron 4	**A**	**A** (100%)	**T** (100%)	**A** (100%)	**A** (100%)	
**104,236,192**	Intron 4	**C**	**T** (3%)*	**C** (100%)	**T** (14%)	**C** (100%)	
**104,236,153**	Intron 4	**C**	**C** (100%)	**C** (100%)	**T** (13%)	**C** (100%)	
**104,236,135**	Exon 5	GA**C**	GA**C** (100%)	GA**C** (100%)	GA**T** (11%)	GA**C** (100%)	CUG→L_{162}<br>CUA→L_{162}
**104,236,108**	Exon 5	CC**C**	CC**C** (100%)	CC**T** (11%)**	CC**T** (13%)	CC**C** (100%)	GGG→G_{171}<br>GGA→G_{171}
**104,235,967**	Exon 5	TT**G**	TT**G** (100%)	TT**A** (14%)	TT**G** (100%)	TT**G** (100%)	AAC→N_{218}<br>AAU→N_{218}
**104,235,656**	Intron 5	**T**	**delT** (97%)	**delT** (78%)	**delT** (73%)	**delT** (91%)	
**104,235,449**	Intron 5	**C**	**C** (100%)	**C** (100%)	**T** (19%)	**T** (11%)**	
**104,235,370**	Intron 5	**G**	**G** (100%)	**G** (100%)	**A** (13%)	**G** (100%)	
**104,235,337**	Intron 5	**C**	**C** (100%)	**C** (100%)	**T** (15%)	**C** (100%)	
**104,235,186**	Intron 5	**G**	**G** (100%)	**G** (100%)	**A** (14%)	**G** (100%)	
**104,235,171**	Exon 6	GA**C**	GA**C** (100%)	GA**T** (8%)*	GA**T** (11%)	GA**C** (100%)	CUG→L_{237}<br>CUA→L_{237}
**104,235,144**	Exon 6	CT**G**	CT**G** (100%)	CT**G** (100%)	CT**A** (15%)	CT**G** (100%)	GAC→D_{246}<br>GAU→D_{246}
**104,235,096**	Exon 6	GA**G**	GA**A** (3%)*	GA**A** (19%)	GA**A** (52%)	GA**G** (100%)	CUC→L_{262}<br>CUU→L_{262}
**104,234,993**	Intron 6	**G**	**G** (100%)	**A** (20%)	**A** (10%)*	**G** (100%)	
**104,234,937**	Intron 6	**C**	**C** (100%)	**C** (100%)	**T** (27%)	**C** (100%)	
**104,234,877**	Intron 6	**C**	**C** (100%)	**C** (100%)	**T** (20%)	**C** (100%)	
**104,234,553**	Intron 6	**G**	**G** (100%)	**G** (100%)	**A** (11%)	**A** (11%)**	
**104,234,436**	Intron 6	**C**	**C** (100%)	**T** (20%)	**C** (100%)	**C** (100%)	
**104,234,328**	Intron 6	**G**	**G** (100%)	**G** (100%)	**A** (14%)	**G** (100%)	
**104,234,256**	Intron 6	**C**	**T** (28%)	**T** (33%)	**T** (10%)*	**C** (100%)**	
**104,234,233**	Intron 6	**C**	**T** (3%)*	**C** (100%)	**T** (15%)	**C** (100%)	
**104,234,009**	Exon 8	AT**G**	AT**A** (7%)*	AT**A** (14%)	AT**A** (14%)	AT**A** (13%)	UAC→Y_{321}<br>UAU→Y_{321}
**104,233,908**	Intron 8	**A**	**A** (100%)	**A** (100%)	**A** (100%)	**C** (17%)	
**104,233,903**	Intron 8	**C**	**C** (100%)	**C** (100%)	**T** (18%)	**C** (100%)	
**104,233,860**	Intron 8	**G**	**G** (100%)	**A** (14%)	**A** (8%)*	**G** (100%)	
**104,233,857**	Intron 8	**G**	**G** (100%)	**G** (100%)	**A** (15%)	**G** (100%)	
**104,233,840**	Intron 8	**G**	**G** (100%)	**G** (100%)	**A** (13%)	**G** (100%)	
**104,233,833**	Intron 8	**C**	**C** (100%)	**T** (11%)	**T** (6%)*	**C** (100%)	
**104,233,831**	Intron 8	**C**	**C** (100%)	**C** (100%)	**T** (17%)	**C** (100%)	
**104,233,787**	Intron 8	**C**	**C** (100%)	**T** (9%)*	**T** (11%)	**C** (100%)	

**104,233,775**	Intron 8	**T**	**T** (100%)	**T** (100%)	**T** (100%)	**C** (10%)*
**104,233,766**	Intron 8	**C**	**C** (100%)	**T** (13%)	**T** (20%)	**C** (100%)
**104,233,765**	Intron 8	**C**	**C** (100%)	**T** (13%)	**C** (92%)	**C** (100%)
**104,233,718**	Intron 8	**G**	**G** (100%)	**A** (15%)	**G** (100%)	**G** (100%)
**104,233,648**	Intron 8	**G**	**G** (100%)	**A** (18%)	**G** (100%)	**G** (100%)
**104,233,645**	Intron 8	**G**	**G** (100%)	**A** (18%)	**G** (100%)	**G** (100%)
**104,233,598**	Intron 8	**G**	**G** (100%)	**G** (100%)	**A** (20%)	**G** (100%)
**104,233,330**	Intron 8	**G**	**G** (100%)	**G** (100%)	**A** (18%)	**G** (100%)
**104,233,260**	Intron 8	**C**	**C** (100%)	**T** (17%)	**C** (100%)	**C** (100%)
**104,233,193**	Intron 8	**G**	**G** (100%)	**G** (100%)	**A** (12%)	**G** (100%)
**104,233,189**	Intron 8	**G**	**G** (100%)	**G** (100%)	**A** (13%)	**G** (100%)
**104,233,186**	Intron 8	**G**	**G** (100%)	**G** (100%)	**A** (12%)	**G** (100%)
**104,233,127**	Intron 8	**G**	**T** (97%)	**G** (100%)	**G** (95%)	**G** (100%)**
**104,233,126**	Intron 8	**G**	**G** (100%)	**G** (100%)	**A** (11%)	**G** (100%)
**104,232,949**	Intron 8	**G**	**G** (100%)	**A** (11%)	**G** (100%)	**G** (100%)
**104,232,911**	Intron 8	**G**	**G** (100%)	**A** (11%)	**G** (100%)	**G** (100%)
**104,232,902**	Intron 8	**C**	**C** (100%)	**C** (100%)	**T** (11%)	**C** (100%)
**104,232,892**	Intron 8	**C**	**C** (100%)	**T** (20%)	**T** (12%)	**C** (100%)
**104,232,891**	Intron 8	**C**	**C** (100%)	**T** (20%)	**T** (6%)*	**C** (100%)
**104,232,886**	Intron 8	**G**	**G** (100%)	**A** (9%)*	**A** (11%)	**G** (100%)
**104,232,884**	Intron 8	**G**	**G** (100%)	**G** (100%)	**A** (12%)	**G** (100%)
**104,232,882**	Intron 8	**C**	**C** (100%)	**C** (100%)	**T** (11%)	**C** (100%)
**104,232,646**	Intron 8	**A**	**G** (97%)	**G** (86%)	**G** (100%)	**G** (100%)
**104,232,616**	Intron 8	**C**	**C** (100%)	**C** (100%)	**C** (100%)	**T** (10%)*
**104,232,532**	Intron 8	**G**	**G** (100%)	**G** (100%)	**A** (11%)	**G** (100%)
**104,232,489**	Intron 8	**C**	**C** (100%)	**C** (100%)	**T** (13%)	**C** (100%)
**104,232,186**	Intron 8	**G**	**G** (100%)	**A** (13%)	**A** (10%)*	**G** (100%)
**104,232,184**	Intron 8	**C**	**C** (100%)	**T** (13%)	**T** (20%)	**C** (100%)
**104,232,161**	Intron 8	**C**	**C** (100%)	**C** (100%)	**T** (17%)	**C** (100%)
**104,232,130**	Intron 8	**C**	**C** (100%)	**T** (25%)	**C** (100%)	**C** (100%)
**104,232,117**	Intron 8	**G**	**G** (100%)	**G** (100%)	**G** (100%)	**T** (10%)*
**104,232,025**	Intron 8	**G**	**G** (100%)	**A** (18%)	**G** (100%)	**G** (100%)
**104,231,993**	Intron 8	**C**	**C** (100%)	**C** (100%)	**T** (14%)	**C** (100%)
**104,231,798**	Intron 9	**C**	**C** (100%)	**T** (11%)	**C** (100%)	**C** (100%)
**104,231,749**	Intron 9	**C**	**C** (100%)	**C** (100%)	**T** (13%)	**C** (100%)

**104,231,731**	Intron 9	**G**	**G** (100%)	**A** (15%)	**G** (100%)	**G** (100%)	
**104,231,642**	Exon 10	CC**C**	CC**T** (4%)*	CC**C** (100%)	CC**T** (17%)	CC**T** (5%)*	GGG→G_{413}<br>GGA→G_{413}
**104,231,621**	Exon 10	CC**C**	CC**C** (100%)	CC**C** (100%)	CC**T** (13%)	CC**C** (100%)	GGG→G_{420}<br>GGA→G_{420}
**104,231,542**	Intron 10	**C**	**C** (100%)	**C** (100%)	**T** (13%)	**C** (100%)	
**104,231,514**	Intron 10	**A**	**A** (100%)	**A** (100%)	**InsA** (14%)	**DelA** (14%)**	
**104,231,438**	Intron 10	**C**	**C** (100%)	**T** (7%)*	**T** (14%)	**C** (100%)	
**104,231,411**	Intron 10	**G**	**G** (100%)	**A** (19%)	**G** (100%)	**G** (100%)	
**104,231,404**	Intron 10	**C**	**T** (4%)*	**C** (100%)	**T** (12%)	**T** (13%)**	
**104,231,298**	Intron 10	**G**	**G** (100%)	**G** (100%)	**A** (15%)	**G** (100%)	
**104,231,278**	Intron 10	**C**	**C** (97%)	**C** (100%)	**T** (12%)	**C** (100%)	
**104,231,242**	Intron 10	**G**	**G** (100%)	**A** (27%)	**G** (100%)	**G** (100%)	
**104,231,201**	Intron 10	**C**	**C** (100%)	**C** (94%)	**T** (14%)	**C** (100%)	
**104,231,200**	Intron 10	**G**	**G** (100%)	**A** (6%)*	**A** (14%)	**G** (100%)	
**104,231,175**	Intron 10	**G**	**G** (100%)	**A** (18%)	**G** (100%)	**G** (100%)	
**104,231,134**	Intron 10	**G**	**G** (100%)	**A** (17%)	**A** (17%)	**G** (100%)	
**104,231,089**	Intron 10	**C**	**C** (100%)	**C** (100%)	**T** (13%)	**C** (100%)	
**104,231,088**	Intron 10	**G**	**G** (100%)	**G** (100%)	**A** (14%)	**G** (100%)	
**104,231,043**	Intron 10	**C**	**C** (100%)	**T** (16%)	**C** (100%)	**C** (100%)	
**104,231,040**	Intron 10	**C**	**C** (100%)	**C** (100%)	**T** (15%)	**C** (100%)	
**104,231,033**	Intron 10	**C**	**C** (100%)	**T** (6%)*	**T** (15%)	**C** (100%)	
**104,231,005**	Intron 10	**C**	**C** (100%)	**T** (14%)	**C** (100%)	**C** (100%)	
**104,230,805**	Intron 10	**G**	**G** (100%)	**G** (100%)	**A** (13%)	**G** (100%)	
**104,230,786**	Intron 10	**G**	**G** (100%)	**G** (100%)	**A** (15%)	**G** (100%)	
**104,230,664**	Intron 10	**G**	**G** (100%)	**A** (20%)	**G** (100%)	**G** (100%)	
**104,230,649**	Intron 10	**C**	**C** (100%)	**T** (17%)	**C** (100%)	**C** (100%)	
**104,230,635**	Intron 10	**A**	**T** (90%)	**T** (100%)	**T** (100%)	**T** (100%)**	
**104,230,594**	Intron 10	**C**	**C** (100%)	**T** (18%)	**C** (100%)	**C** (100%)	
**104,230,577**	Intron 10	**G**	**A** (3%)*	**G** (100%)	**A** (14%)	**G** (100%)	
**104,230,548**	Intron 10	**C**	**C** (95%)	**C** (100%)	**T** (17%)	**C** (100%)	
**104,230,496**	Intron 10	**G**	**G** (100%)	**A** (14%)	**G** (100%)	**G** (100%)	
**104,230,422**	Intron 10	**G**	**G** (100%)	**A** (18%)	**G** (100%)	**A** (8%)*	
**104,230,397**	Intron 10	**C**	**C** (100%)	**T** (20%)	**C** (100%)	**C** (100%)	
**104,230,384**	Intron 10	**C**	**C** (100%)	**C** (100%)	**T** (17%)	**C** (100%)	
**104,230,352**	Intron 10	**C**	**C** (100%)	**C** (100%)	**T** (12%)	**C** (100%)	

**104,230,319**	Intron 10	**C**	**C** (100%)	**T** (12%)	**C** (100%)	**C** (100%)
**104,230,014**	Downstream region	**G**	**G** (100%)	**G** (100%)	**A** (30%)	**G** (100%)
**104,229,887**	Downstream region	**C**	**C** (100%)	**T** (14%)	**C** (100%)	**C** (100%)
**104,229,778**	Downstream region	**G**	**G** (100%)	**A** (8%)*	**A** (15%)	**G** (100%)
**104,229,768**	Downstream region	**C**	**C** (100%)	**T** (12%)	**T** (7%)*	**C** (100%)
**104,229,767**	Downstream region	**C**	**C** (100%)	**C** (100%)	**T** (13%)	**C** (100%)
**104,229,761**	Downstream region	**G**	**G** (100%)	**G** (100%)	**A** (18%)	**G** (100%)
**104,229,742**	Downstream region	**G**	**G** (100%)	**A** (13%)	**G** (100%)	**G** (100%)
**104,229,617**	Downstream region	**G**	**G** (100%)	**A** (17%)	**G** (100%)	**G** (100%)
**104,229,602**	Downstream region	**C**	**C** (100%)	**T** (25%)	**C** (100%)	**C** (100%)
**104,229,591**	Downstream region	**G**	**G** (100%)	**A** (13%)	**G** (100%)	**G** (100%)
**104,229,568**	Downstream region	**C**	**C** (100%)	**T** (5%)*	**T** (18%)	**C** (100%)
**104,229,563**	Downstream region	**G**	**G** (100%)	**A** (12%)	**G** (100%)	**G** (100%)
**104,229,544**	Downstream region	**C**	**C** (100%)	**T** (11%)	**T** (10%)*	**C** (100%)
**104,229,543**	Downstream region	**C**	**C** (100%)	**T** (11%)	**C** (100%)	**C** (100%)
**104,229,502**	Downstream region	**C**	**C** (100%)	**T** (17%)	**T** (8%)*	**C** (100%)

n.a.: not available; the variant frequency is referred to the percentage of the highlighted base in the sequenced ancient hominine genome, with* frequency≤10% and ** counts<10. In light orange are underlined the variants fixed at 100% in modern human compared to ancient hominines.
