# Supplementary material for: A Catalog of Coding Sequence Variations in Salivary Proteins’ Genes Occurring during Recent Human Evolution
Source: Int J Mol Sci. 2023 Oct 9;24(19):15010. doi: 10.3390/ijms241915010 (PMC10573131; doi:10.3390/ijms241915010)
Supplement: Supplementary file 1 [file ijms-24-15010-s001.zip › Table S9 .pdf]

Table S9. Nucleotide Substitutions in *STATH* gene in introns and regulatory regions

Genomic Coordinates (hg19)	Gene Region	Modern human	Altai Neanderthal (Variant Frequency)	Chagyrskaya Neanderthal (Variant Frequency)	Vindija Neanderthal (Variant Frequency)	Denisovan (Variant Frequency)	Codon → Amino acid
**70,861,286**	Upstream region	**G**	**A** (7%)*	**G** (100%)	**A** (14%)	**A** (3%)*	
**70,861,321**	Upstream region	**G**	**G** (98%)	**G** (100%)	**A** (17%)	**G** (100%)	
**70,861,322**	Upstream region	**G**	**G** (100%)	**G** (100%)	**A** (11%)	**G** (100%)	
**70,861,326**	Upstream region	**G**	**G** (100%)	**A** (3%)*	**A** (16%)	**G** (100%)	
**70,861,343**	Upstream region	**G**	**G** (100%)	**G** (100%)	**A** (14%)	**G** (100%)	
**70,861,345**	Upstream region	**G**	**G** (100%)	**G** (100%)	**A** (21%)	**G** (100%)	
**70,861,354**	Upstream region	**C**	**C** (100%)	**C** (100%)	**T** (11%)	**C** (100%)	
**70,861,408**	Upstream region	**G**	**G** (100%)	**G** (96%)	**A** (11%)	**G** (100%)	
**70,861,449**	Upstream region	**C**	**C** (100%)	**C** (100%)	**T** (12%)	**C** (100%)	
**70,861,482**	Upstream region	**G**	**G** (100%)	**A** (19%)	**A** (4%)*	**G** (100%)	
**70,861,575**	Upstream region	**G**	**G** (100%)	**A** (9%)*	**A** (13%)	**G** (100%)	
**70,861,643**	Upstream region	**C**	**C** (100%)	**C** (100%)	**T** (11%)	**C** (100%)	
**70,861,650**	5′UTR	**G**	**G** (100%)	**A** (17%)	**A** (4%)*	**G** (100%)	
**70,861,675**	5′UTR	**G**	**G** (100%)	**G** (100%)	**A** (11%)	**G** (100%)	
**70,861,702**	5′UTR	**C**	**C** (100%)	**T** (15%)	**T** (5%)*	**C** (100%)	
**70,861,720**	5′UTR	**T**	**C** (55%)	**T** (100%)	**T** (100%)	**T** (100%)	
**70,861,728**	5′UTR	**C**	**C** (100%)	**C** (100%)	**T** (12%)	**C** (100%)	
**70,861,779**	Intron 1	**A**	**G** (100%)	**G** (96%)	**G** (96%)	**A** (100%)	
**70,861,786**	Intron 1	**G**	**G** (100%)	**G** (100%)	**A** (11%)	**G** (97%)	
**70,861,796**	Intron 1	**C**	**C** (99%)	**T** (4%)*	**T** (12%)	**C** (100%)	
**70,861,974**	Intron 1	**A**	**G** (100%)	**G** (97%)	**G** (96%)	**G** (100%)	
**70,862,105**	Intron 1	**G**	**A** (8%)*	**A** (9%)*	**A** (13%)	**G** (100%)	
**70,862,192**	Intron 1	**C**	**C** (100%)	**T** (21%)	**C** (100%)	**C** (100%)	
**70,862,294**	Intron 1	**C**	**T** (97%)	**T** (100%)	**T** (100%)	**C** (100%)	
**70,862,374**	Intron 1	**G**	**A** (1%)*	**A** (2%)*	**A** (13%)	**G** (100%)	
**70,862,380**	Intron 1	**T**	**T** (100%)	**T** (100%)	**delT** (41%)	**T** (97%)	
**70,862,448**	Intron 1	**G**	**G** (100%)	**A** (8%)*	**A** (25%)	**G** (100%)	
**70,862,458**	Intron 1	**C**	**C** (99%)	**C** (100%)	**T** (13%)	**C** (100%)	
**70,862,463**	Intron 1	**C**	**T** (1%)*	**T** (13%)	**T** (4%)*	**C** (100%)	
**70,862,526**	Intron 1	**C**	**C** (100%)	**T** (3%)*	**T** (11%)	**C** (100%)	

**70,862,547**	Intron 1	**C**	**T** (99%)	**T** (100%)	**T** (97%)	**C** (100%)		
**70,862,645**	Intron 1	**T**	**delT** (44%)	**delT** (20%)**	**delT** (25%)	**delT** (33%)		
**70,862,646**	Intron 1	**T**	**delT** (11%)	**delT** (20%)**	**delT** (10%)*	**delT** (17%)		
**70,862,666**	Intron 1	**T**	**A** (18%)	**A** (50%)**	**A** (19%)	**A** (7%)*		
**70,862,668**	Intron 1	**A**	**T** (19%)	**A** (100%)	**T** (4%)*	**A** (100%)		
**70,862,677**	Intron 1	**A**	**G** (22%)	**G** (17%)**	**G** (20%)	**A** (100%)		
**70,862,684**	Intron 1	**C**	**C** (96%)	**C** (100%)	**T** (20%)	**C** (100%)		
**70,862,700**	Intron 1	**G**	**G** (100%)	**G** (100%)	**A** (15%)	**G** (100%)		
**70,862,715**	Intron 1	**T**	**C** (45%)	**C** (47%)	**C** (13%)	**T** (100%)		
**70,862,730**	Intron 1	**C**	**T** (3%)*	**T** (11%)	**T** (6%)*	**T** (4%)*		
**70,862,732**	Intron 1	**G**	**G** (100%)	**A** (11%)	**G** (100%)	**G** (100%)		
**70,862,736**	Intron 1	**C**	**T** (3%)*	**C** (100%)	**T** (13%)	**C** (100%)		
**70,862,797**	Intron 1	**G**	**G** (100%)	**G** (100%)	**A** (13%)	**G** (100%)		
**70,862,917**	Intron 1	**C**	**C** (100%)	**C** (100%)	**T** (13%)	**C** (100%)		
**70,863,157**	Intron 1	**C**	**C** (100%)	**T** (3%)*	**T** (12%)	**C** (100%)		
**70,863,163**	Intron 1	**C**	**C** (99%)	**T** (11%)	**T** (12%)	**T** (4%)*		
**70,863,200**	Intron 1	**G**	**G** (100%)	**A** (13%)	**A** (5%)*	**G** (100%)		
**70,863,375**	Intron 1	**G**	**G** (100%)	**A** (3%)*	**A** (11%)	**G** (100%)		
**70,863,442**	Intron 1	**G**	**A** (2%)*	**G** (100%)	**A** (11%)	**G** (100%)		
**70,863,443**	Intron 1	**G**	**G** (98%)	**A** (8%)*	**A** (15%)	**G** (100%)		
**70,863,668**	Intron 1	**C**	**C** (100%)	**T** (12%)	**C** (100%)	**C** (100%)		
**70,863,751**	Intron 1	**C**	**T** (2%)*	**C** (100%)	**T** (12%)	**C** (100%)		
**70,863,822**	Intron 1	**C**	**C** (100%)	**T** (13%)	**C** (100%)	**C** (100%)		
**70,863,839**	Intron 1	**T**	**C** (100%)	**C** (92%)	**C** (86%)	**C** (97%)		
**70,863,883**	Intron 1	**C**	**C** (100%)	**T** (12%)	**T** (15%)	**C** (100%)		
**70,863,924**	Intron 1	**A**	**T** (100%)	**T** (100%)	**T** (100%)	**A** (100%)		
**70,863,952**	Intron 1	**A**	**G** (100%)	**G** (92%)	**G** (100%)	**A** (100%)		
**70,863,957**	Intron 1	**C**	**T** (100%)	**T** (100%)	**T** (100%)	**C** (100%)		
**70,864,053**	Intron 1	**G**	**G** (100%)	**A** (13%)	**G** (100%)	**G** (100%)		
**70,864,178**	Exon 2 (Signal)	TT<u>C</u>	TT<u>C</u> (100%)	TT<u>C</u> (100%)	TT<u>**T**</u> (11%)	TT<u>C</u> (100%)	UUC→$F_{7(sp)}$ UUU→$F_{7(sp)}$	
**70,864,261**	Intron 2	**C**	**C** (98%)	**T** (11%)	**T** (3%)*	**C** (100%)		
**70,864,274**	Intron 2	**G**	**G** (100%)	**A** (15%)	**G** (100%)	**G** (100%)		
**70,864,349**	Intron 2	**C**	**C** (100%)	**T** (12%)	**T** (8%)*	**C** (100%)		
**70,864,353**	Intron 2	**C**	**C** (100%)	**C** (100%)	**T** (11%)	**C** (100%)		

**70,864,509**	Intron 2	**C**	**C** (100%)	**T** (13%)	**T** (3%)*	**C** (100%)
**70,864,733**	Intron 2	**T**	**G** (98%)	**G** (100%)	**G** (100%)	**T** (100%)
**70,864,751**	Intron 2	**A**	**G** (97%)	**G** (100%)	**G** (89%)	**A** (100%)
**70,864,762**	Intron 2	**G**	**A** (2%)*	**G** (100%)	**A** (13%)	**G** (100%)
**70,864,768**	Intron 2	**G**	**G** (100%)	**A** (11%)	**A** (8%)*	**G** (100%)
**70,864,822**	Intron 2	**G**	**G** (100%)	**G** (100%)	**A** (13%)	**G** (100%)
**70,864,970**	Intron 2	**C**	**C** (100%)	**T** (5%)*	**T** (12%)	**C** (100%)
**70,865,133**	Intron 2	**C**	**T** (1%)*	**C** (100%)	**T** (19%)	**C** (100%)
**70,865,136**	Intron 2	**C**	**C** (100%)	**T** (10%)*	**T** (12%)	**C** (100%)
**70,865,137**	Intron 2	**C**	**C** (100%)	**T** (13%)	**T** (4%)*	**C** (97%)
**70,865,152**	Intron 2	**C**	**C** (99%)	**C** (100%)	**T** (11%)	**C** (100%)
**70,865,189**	Intron 2	**C**	**C** (98%)	**C** (100%)	**T** (14%)	**C** (100%)
**70,865,193**	Intron 2	**C**	**C** (100%)	**C** (100%)	**T** (11%)	**C** (100%)
**70,865,261**	Intron 2	**G**	**G** (100%)	**A** (11%)	**A** (3%)*	**G** (100%)
**70,865,284**	Intron 2	**C**	**C** (100%)	**C** (100%)	**T** (11%)	**C** (100%)
**70,865,285**	Intron 2	**C**	**C** (100%)	**C** (100%)	**T** (13%)	**C** (100%)
**70,865,405**	Intron 3	**G**	**A** (3%)*	**A** (2%)*	**A** (13%)	**G** (100%)
**70,865,533**	Intron 4	**G**	**A** (100%)	**A** (100%)	**A** (100%)	**G** (100%)
**70,865,633**	Intron 4	**C**	**T** (1%)*	**T** (11%)	**C** (100%)	**C** (100%)
**70,865,638**	Intron 4	**G**	**G** (100%)	**A** (13%)	**G** (100%)	**G** (100%)
**70,865,677**	Intron 4	**A**	**C** (100%)	**C** (100%)	**C** (81%)	**C** (100%)
**70,865,748**	Intron 4	**C**	**T** (2%)*	**T** (14%)	**T** (5%)*	**C** (100%)
**70,865,768**	Intron 4	**C**	**C** (100%)	**T** (4%)*	**T** (14%)	**C** (100%)
**70,865,853**	Intron 4	**G**	**G** (100%)	**A** (11%)	**G** (100%)	**G** (100%)
**70,866,032**	Intron 4	**G**	**G** (100%)	**G** (100%)	**A** (13%)	**G** (100%)
**70,866,033**	Intron 4	**G**	**G** (98%)	**G** (100%)	**A** (13%)	**G** (100%)
**70,866,034**	Intron 4	**G**	**G** (100%)	**G** (100%)	**A** (19%)	**G** (100%)
**70,866,046**	Intron 4	**C**	**C** (100%)	**C** (100%)	**T** (13%)	**C** (100%)
**70,866,061**	Intron 4	**G**	**G** (100%)	**G** (100%)	**A** (14%)	**G** (100%)
**70,866,109**	Intron 4	**G**	**G** (98%)	**A** (4%)*	**A** (15%)	**G** (100%)
**70,866,118**	Intron 4	**T**	**C** (96%)	**C** (95%)	**C** (94%)	**T** (100%)
**70,866,221**	Intron 4	**T**	**A** (97%)	**A** (100%)	**A** (100%)	**A** (100%)
**70,866,267**	Intron 4	**G**	**G** (100%)	**G** (100%)	**A** (13%)	**G** (100%)
**70,866,278**	Intron 4	**G**	**A** (2%)*	**A** (6%)*	**A** (11%)	**G** (100%)
**70,866,503**	Intron 4	**C**	**C** (100%)	**T** (12%)	**C** (100%)	**C** (100%)

**70,866,529**	Intron 4	**C**	**C** (100%)	**T** (11%)	**T** (5%)*	**C** (100%)	
**70,866,660**	Exon 5	AC<u>**C**</u>	AC<u>**C**</u> (98%)	AC<u>**T**</u> (11%)	AC<u>**C**</u> (100%)	AC<u>**C**</u> (100%)	ACC→T_{42}<br>ACU→T_{42}
**70,866,707**	Intron 5	**G**	**G** (100%)	**G** (100%)	**A** (12%)	**G** (100%)	
**70,866,776**	Intron 5	**A**	**C** (100%)	**C** (100%)	**C** (98%)	**A** (100%)	
**70,866,862**	Intron 5	**G**	**G** (100%)	**T** (11%)	**G** (100%)	**G** (100%)	
**70,866,864**	Intron 5	**T**	**T** (100%)	**A** (11%)	**T** (100%)	**T** (100%)	
**70,866,865**	Intron 5		**InsAA** (92%)	**InsAA** (55%)	**InsAA** (100%)	**InsAA** (100%)	
**70,866,866**	Intron 5	**G**	**G** (100%)	**A** (28%)	**G** (100%)	**G** (100%)	
**70,866,867**	Intron 5	**T**	**T** (100%)	**A** (28%)	**T** (100%)	**T** (100%)	
**70,866,868**	Intron 5	**C**	**C** (100%)	**C** (100%)	**G** (18%)	**C** (100%)	
**70,866,893**	Intron 5	**A**	**T** (100%)	**T** (100%)	**T** (100%)	**A** (100%)	
**70,866,917**	Intron 5	**G**	**A** (3%)*	**G** (100%)	**A** (15%)	**G** (100%)	
**70,866,944**	Intron 5	**C**	**C** (100%)	**T** (7%)*	**T** (17%)	**C** (100%)	
**70,866,954**	Intron 5	**G**	**G** (100%)	**G** (100%)	**A** (15%)	**G** (100%)	
**70,866,963**	Intron 5	**C**	**C** (100%)	**C** (100%)	**T** (11%)	**C** (100%)	
**70,866,979**	Intron 5	**T**	**C** (98%)	**C** (100%)	**C** (100%)	**T** (100%)	
**70,866,980**	Intron 5	**G**	**A** (2%)*	**G** (100%)	**A** (11%)	**G** (100%)	
**70,866,996**	Intron 5	**G**	**A** (98%)	**A** (100%)	**A** (100%)	**G** (100%)	
**70,867,021**	Intron 5	**C**	**C** (100%)	**T** (7%)*	**T** (13%)	**C** (100%)	
**70,867,092**	Intron 5	**G**	**G** (100%)	**A** (13%)	**A** (6%)*	**G** (100%)	
**70,867,137**	Intron 5	**C**	**C** (100%)	**T** (4%)*	**T** (14%)	**C** (100%)	
**70,867,142**	Intron 5	**G**	**G** (100%)	**A** (14%)	**A** (5%)*	**G** (100%)	
**70,867,237**	Intron 5	**C**	**C** (100%)	**T** (13%)	**T** (4%)*	**C** (100%)	
**70,867,238**	Intron 5	**C**	**C** (100%)	**C** (100%)	**T** (14%)	**C** (100%)	
**70,867,289**	Intron 5	**C**	**T** (1%)*	**T** (6%)*	**T** (12%)	**T** (3%)*	
**70,867,343**	Intron 5	**C**	**C** (100%)	**T** (3%)*	**T** (11%)	**C** (100%)	
**70,867,424**	Intron 5	**G**	**A** (1%)*	**A** (11%)	**A** (3%)*	**G** (100%)	
**70,867,426**	Intron 5	**G**	**G** (100%)	**A** (11%)	**A** (3%)*	**G** (100%)	
**70,867,431**	Intron 5	**G**	**G** (100%)	**A** (10%)*	**A** (12%)	**G** (100%)	
**70,867,457**	Intron 5	**G**	**G** (100%)	**G** (100%)	**A** (11%)	**G** (100%)	
**70,867,522**	Intron 5	**G**	**A** (100%)	**A** (100%)	**A** (100%)	**G** (100%)	
**70,867,531**	Intron 5	**G**	**A** (2%)*	**A** (12%)	**A** (2%)*	**G** (100%)	
**70,867,535**	Intron 5	**G**	**G** (100%)	**A** (11%)	**A** (2%)*	**G** (100%)	
**70,867,537**	Intron 5	**G**	**G** (97%)	**A** (11%)	**A** (5%)*	**G** (100%)	

**70,867,623**	Intron 5	**G**	**G** (100%)	**G** (100%)	**A** (13%)	**G** (100%)
**70,867,656**	Intron 5	**T**	**C** (98%)	**C** (100%)	**C** (97%)	**T** (100%)
**70,867,787**	Intron 5	**C**	**C** (100%)	**T** (6%)*	**T** (12%)	**C** (100%)
**70,867,856**	Intron 5	**C**	**C** (100%)	**T** (3%)*	**T** (13%)	**C** (100%)
**70,867,859**	Intron 5	**G**	**G** (100%)	**A** (6%)*	**A** (17%)	**G** (100%)
**70,867,886**	Intron 5		**-**	**-**	**InsT** (23%)	**-**
**70,867,898**	Intron 5	**C**	**C** (98%)	**C** (100%)	**T** (12%)	**T** (2%)*
**70,867,971**	3′UTR	**C**	**C** (100%)	**C** (100%)	**T** (13%)	**C** (100%)
**70,868,014**	3′UTR	**C**	**C** (100%)	**T** (7%)*	**T** (11%)	**C** (100%)
**70,868,162**	3′UTR	**A**	**G** (98%)	**G** (97%)	**G** (94%)	**A** (100%)
**70,868,247**	Downstream regions	**C**	**C** (100%)	**T** (9%)*	**T** (11%)	**C** (100%)
**70,868,250**	Downstream regions	**G**	**G** (100%)	**G** (100%)	**A** (16%)	**G** (100%)
**70,868,280**	Downstream regions	**C**	**C** (100%)	**T** (12%)	**T** (4%)*	**C** (100%)
**70,868,378**	Downstream regions	**C**	**C** (100%)	**T** (12%)	**C** (97%)	**C** (100%)
**70,868,397**	Downstream regions	**C**	**C** (100%)	**C** (100%)	**T** (12%)	**C** (100%)
**70,868,398**	Downstream regions	**C**	**C** (100%)	**T** (11%)	**T** (6%)*	**C** (100%)
**70,868,410**	Downstream regions	**G**	**A** (2%)*	**A** (3%)*	**A** (11%)	**G** (100%)
**70,868,420**	Downstream regions	**C**	**T** (2%)*	**T** (6%)*	**T** (11%)	**C** (100%)
**70,868,471**	Downstream regions	**C**	**C** (100%)	**T** (11%)	**C** (100%)	**C** (100%)
**70,868,546**	Downstream regions	**T**	**C** (99%)	**C** (100%)	**C** (96%)	**T** (100%)
**70,868,550**	Downstream regions	**C**	**T** (99%)	**T** (100%)	**T** (100%)	**C** (100%)
**70,868,585**	Downstream regions	**T**	**G** (98%)	**G** (91%)	**G** (100%)	**T** (100%)

n.a.: not available; the variant frequency is referred to the percentage of the highlighted base in the sequenced ancient hominine genome, with* frequency≤10% and ** counts<10. In light orange are underlined the variants fixed at 100% in modern human compared to ancient hominines.
